# Gene Mining and Flavour Metabolism Analyses of *Wickerhamomyces anomalus* Y-1 Isolated From a Chinese Liquor Fermentation Starter

**DOI:** 10.3389/fmicb.2022.891387

**Published:** 2022-05-02

**Authors:** Xin Shi, Xin Wang, Xiaoge Hou, Qing Tian, Ming Hui

**Affiliations:** ^1^College of Biological Engineering, Henan University of Technology, Zhengzhou, China; ^2^Industrial Microorganism Preservation and Breeding Henan Engineering Laboratory, Zhengzhou, China; ^3^School of Food and Bioengineering, Henan College of Animal Husbandry Economics, Zhengzhou, China

**Keywords:** Luzhou-flavoured liquor, *Wickerhamomyces anomalus*, ethyl hexanoate biosynthesis pathway, medium-chain fatty acid ethyl ester, whole genome sequencing

## Abstract

Luzhou-flavoured liquor is one of Chinese most popular distilled liquors. Hundreds of flavoured components have been detected from this liquor, with esters as its primary flavouring substance. Among these esters, ethyl hexanoate was the main component. As an essential functional microbe that produces ethyl hexanoate, yeast is an important functional microorganism that produces ethyl hexanoate. The synthesis of ethyl hexanoate in yeast mainly involves the lipase/esterase synthesis pathway, alcohol transferase pathway and alcohol dehydrogenase pathway. In this study, whole-genome sequencing of *W. anomalus* Y-1 isolated from a Chinese liquor fermentation starter, a fermented wheat starter containing brewing microorganisms, was carried out using the Illumina HiSeq X Ten platform. The sequence had a length of 15,127,803 bp with 34.56% GC content, encoding 7,024 CDS sequences, 69 tRNAs and 1 rRNA. Then, genome annotation was performed using three high-quality databases, namely, COG, KEGG and GO databases. The annotation results showed that the ko7019 pathway of gene 6,340 contained the Eht1p enzyme, which was considered a putative acyltransferase similar to Eeb1p and had 51.57% homology with two known medium-chain fatty acid ethyl ester synthases, namely, Eht1 and Eeb1. Ethyl hexanoate in *W. anomalus* was found to be synthesised through the alcohol acyltransferase pathway, while acyl-coenzyme A and alcohol were synthesised under the catalytic action of Eht1p. The results of this study are beneficial to the exploration of key genes of ester synthesis and provide reference for the improvement of liquor flavoured.

## Introduction

Luzhou-flavoured liquor, with thousands of years of history, is a typical traditional distilled liquor in China (Zheng et al., 2015a,b; Jin et al., 2017). The fermentation of this liquor involves the use of sorghum as raw material, a mixed steaming process and medium-temperature Daqu as saccharifying and fermentation agent. This liquor has a rich cellar, sweet, mellow and harmonious fragrance (Qiu et al., 2016). Its main flavour component is ethyl hexanoate, and the right amount of ethyl butyrate, ethyl acetate and ethyl lactate constitute the compound aroma (Wei et al., 2020). These aromas are usually inferred to be formed by the exceptional microbial environment during brewing (Zou et al., 2018). The production of Luzhou-flavoured liquor usually consists of four stages (Liu and Sun, 2018). The first stage is the cultivation of the pit mud. An excellent pit mud is an essential condition for the production of high-quality Luzhou-flavoured liquor (Lu et al., 2020). The second stage is the production of Daqu, as the liquor saccharifying and fermentation agent (Li et al., 2019). Daqu, which resembles a brick in appearance, is a spontaneous fermentation product in an open environment and is an important fermenting agent in the fermentation of liquor (Jin et al., 2017). Daqu contains a variety of microorganisms and a variety of hydrolytic enzymes (Ling et al., 2020). It is generally accepted that the fermentation of liquor is initiated by the interaction of these enzymes, and the activity of these enzymes is an important test indicator in the production process (Zhang et al., 2019). The next stage is brewing, cooking pulverised raw materials, cooling and then the addition of Daqu for saccharification leading to fermentation. Finally, the original liquor is stored and blended to reduce the pungency and irritation of the new liquor. Blending can adjust the proportion of each component in the liquor. The addition of Daqu during the fermentation of Chinese liquor and the influence of different pit mud result in liquor with different flavour substances (He et al., 2019b; Yang et al., 2020). Due to differences in the natural environment, the flavour of Baijiu varies significantly from region to region and the specific action of different microorganisms affects the composition of the enzyme system in the fermentation grains and thus the formation of esters. Daqu provides an important source of microorganisms for the fermentation process and is a key factor in the quality and style of China liquor (He et al., 2019a).

According to the characteristics of liquor fermentation, it can be divided into three stages (Cheng et al., 2017; Yin et al., 2020). The first stage is the primary fermentation stage, which is the production of ethanol. The second stage is the biogenic acid stage. In addition to the generation of alcohol and sugar, a large number of organic acids are produced during this stage. The third stage is the production of aromas, which are produced by the interaction of microorganisms in the pit mud and daqu. The flavour analysis of China liquor revealed that ethanol and water accounted for 98% of the total mass and flavour substances accounted for 2% (Liu and Sun, 2018). Flavour substances mainly include alcohols, esters, aldehydes, ketones, acids, pyrazines, etc. (Hong et al., 2020). Esters account for more than 60% of the total flavour substances, and most esters have a low odour threshold but contribute more to the odour. In addition to this, these esters can affect the taste of China liquor together with acids and alcohols. Different types and concentrations of esters therefore have a crucial influence on the flavour of baijiu. The ester-producing yeast has been identified as one of the important microbe affecting the content of esters in liquor (Xu et al., 2017; Wang et al., 2019).

Studies have shown that esters are synthesised by the lipase/esterase pathway (Wang et al., 2008), alcohol transferase pathway (Wolter et al., 1966) and alcohol dehydrogenase pathway (Kusano et al., 1998). The lipase/esterase synthesis pathway, namely, the esterification reaction pathway, is the most common pathway used to generate esters in liquor (Papamichael, 2013). This process refers to the slow reaction of organic acids and alcohols to form esters. The acyltransferase pathway is the main synthesis pathway of ethyl hexanoate in *Saccharomyces cerevisiae* (Furukawa et al., 2003). The alcohol dehydrogenase pathway is the oxidation of hemiacetals to esters. At present, *Zygosaccharomyces rouxii* (Rojo et al., 2017), *Pichia anomala* (Zhang et al., 2014), *Kluyveromyces marxianus* (Reyes-Sánchez et al., 2020), *Saccharomycopsis fibuligera* (Yang et al., 2020), *Wickerhamomyces anomalus* (Wang et al., 2020) and other yeasts have been reported to have high esterification capacity, but the esterification mechanism of some yeasts has not been revealed. Nowadays, whole-genome sequencing has been a reliable tool for gene annotation and analysis to understand microbial metabolism, and many important results have been achieved (Wang et al., 2018).

In this study, a strain of *Wickerhamomyces anomalus* (named Y-1) with a high yield of ethyl hexanoate was isolated from a Chinese liquor fermentation starter. Then, whole-genome sequencing was performed using the second-generation sequencer, Illumina HiSeq X Ten platform. The obtained genome sequence was assembled and annotated. Genome annotation was performed based on three high-quality databases, namely, the Clusters of Orthologous Groups of proteins (COG), Kyoto Encyclopaedia of Genes and Genomes (KEGG) and Gene Ontology (GO) databases. Finally, the ethyl hexanoate biosynthesis pathway of the *W. anomalus* Y-1 was analysed, facilitating the design and construction of the industrial strains.

## Materials and Methods

### Raw Materials and Culture Medium

The Chinese liquor fermentation starter was provided by Songhe Liquor Co., Ltd. (Zhoukou City, Henan Province, China), the starter is a medium-temperature Daqu and the maximum temperature during the manufacture of Daqu is less than 60°C.

The yeast screening medium consisted of glucose (20 g/l), peptone (5 g/l), agar (20 g/l) and amoxicillin (1 g/l).

The yeast extract peptone dextrose (YEPD) medium contained peptone (20 g/l), yeast extract (10 g/l), glucose (20 g/l) and agar (20 g/l).

The ester production medium consisted of yeast extract (1 g/l), glucose (10 g/l), peptone (20 g/l), (NH_4_) _2_SO_4_ (0.1 g/l), KH_2_PO_4_ (0.1 g/l), MgSO_4_ (0.1 g/l), CaCl_2_ (0.1 g/l) and NH_4_Cl (0.2 g/l).

The identification medium for esterase production contained yeast extract (3 g/l), peptone (5 g/l), agar (20 g/l) and 0.1% tributyrin.

### Screening of Ester-Producing Yeast

Firstly, yeast was isolated from the Chinese liquor fermentation starter. Add daqu (10 g) to 90 ml sterile water. After shaking well, transfer 1 ml mixture to 9 ml pure water, in a dilution ratio of 10^−2^. According to this method, the solution was diluted to 10^−3^, 10^−4^ and 10^−5^ in turn. Then, 0.2 ml of the diluted solution was absorbed and spread in the yeast screening medium for each gradient and cultured at 28°C for 2 days. Single colonies were picked and purified on the YEPD plate to screen the ester-producing yeast. The YEPD medium activated the strains preserved on the oblique plane. After activation, the seed solution was inoculated in 100 ml of the ester-producing medium at 10% inoculum size and cultured at 28°C and 120 rpm for 2 days. After yeast culture, the content of total ester in the fermentation broth was determined by the saponification reflux method (Zheng et al., 2015). The screened ester-producing yeast was prepared into the seed liquid, and 2 μl of seed liquid was absorbed and coated on the esterase identification medium with triglyceride and cultured at 28°C for 2 days. Tributyrin is commonly used to screen lipase-producing and esterase-producing strains, and lipase activity is measured by observing the hydrolytic zone on nutrient Agar containing tributyrin (Farooq et al., 2021). In this experiment, by measuring the diameter of yeast colony (d) and the diameter of surrounding transparent ring (D), and calculating their ratio (D/d), to judge the size of yeast ester production capacity.

### Identification of Ester-Producing Yeast

The screened strains were streaked on a YPD medium and cultured at 28°C for 2 days. The colony and microscopic morphologies were observed. The DNA was extracted using the Omega Fungal DNA Kit D3390-02 according to the manufacturer’s instructions, and high-quality genomic 18SrDNA was obtained for amplification. Electrophoresis and sequencing of the amplified products were performed. The obtained sequences were compared in the NCBI online software BLAST to determine the strain type. The phylogenetic tree was constructed using the N-J method in MEGA 7.0 software.

### Strain Culture and Detection of the Flavoured Components

The strain was cultured in the ester-producing medium. For liquid fermentation, 2% seed liquor was inoculated in the medium and complexed at 28°C for 2 days. The fermentation broth was centrifuged at the end of the culture, and the supernatant was taken to detect the flavoured components.

The flavoured components were extracted using a solid-phase microextraction (SPME) method, with divinylbenzene/carboxy on polydimethylsiloxane (50/30 μm) fibre. The fibre was preconditioned in the gas chromatograph (GC) injection port at 250°C for 30 min, as indicated by the manufacturer. For headspace sampling, each sample (5 ml) was placed in a 20 ml vial and determined using solid-phase microextraction (SPME) coupled with GC-mass spectrometry (MS). The SPME fibre was exposed to the headspace at 50°C for 50 min. After sampling, the SPME fibre was introduced into the GC injector and left for 5 min for the thermal desorption of the analytes (Fan et al., 2011).

GC–MS was carried out using a Thermo Scientific Trace 1,300 Gas Chromatograph coupled to a Thermo Scientific ISO 7000 Single Quadrupole Mass Spectrometer selective detector operating in electron impact mode (ionisation voltage of 70 eV; Thermo Fisher Scientific, Waltham, MA, United States). A DB-WAX capillary column (30 m × 0.25 mm i.d., 0.25 μm film thickness, J&W Scientific, USA) was equipped. Ultra-high-purity helium was used as the carrier gas at a constant flow of 1 ml/min. The split ratio was 20:1. The oven temperature was programmed as follows: column temperature starting temperature from 30°C, constant temperature for 1 min; increased rapidly by 4°C to 120°C, constant temperature for 2 min. Then, the SPME fibre volatiles was heated to 210°C at 7°C/min for 6 min. The MS conditions were as follows: ion source temperature, 280°C; transmission line temperature, 215°C; electron ionisation mode, 70 eV; and mass-to-charge (m/z) range, 33–450 in full scan acquisition mode (Ding et al., 2015). The MS spectral database library of the National Institute for Standards and Technology was used to identify the compounds. Tentatively identified compounds also had to fit logically the retention time in the chromatograms. Quantification analysis was performed using 2% n-butyl acetate as an internal standard.

### Genome Sequencing

Firstly, the Y-1 strain was cultured in a liquid ester-producing medium to OD_600_ = 0.7, and the cell genome was extracted by centrifugation. The DNA was extracted using the Omega Fungal DNA Kit D3390-02 according to the manufacturer’s instructions, and NanoDrop was employed to determine the purity and concentration of the samples. The absorbance values at 260 nm and 280 nm were measured. The A_260_/A_280_ ratio was more significant than 1.8, indicating that the nucleic acid was more excellent than 90%, which can be used for subsequent sequencing. Qualified samples were prepared for database construction and quality inspection. The eligible gene library was sequenced, and the final data were subjected to quality control procedures.

DNA degradation and contamination were detected using 1% agarose gel. A nanophotometer was used to determine DNA purity. DNA concentration was determined by TBS-380 fluorometer (Turner BioSystems Inc., Sunnyvale, CA), and the ultrasonic processor lysed the qualified DNA. The inserted fragment was~350 bp in length. Then, end repair, base addition, sequence adapter addition, purification and PCR amplification were performed to complete the preparation of the 350 bp library. The concentration was diluted to 1 ng/μL for the initial quantification with TBS-380 fluorometer. Then, Agilent 2,100 electrophoresis bioanalyzer was used to verify the size of the inserted fragment of the library, and qPCR was performed to measure the effective quantitative concentration of the library. For the identified libraries, sequencing was performed using Illumina HiSeq X Ten, PE150 (Illumina, San Diego, CA, United States).

### Genome Assembly and Annotation

The genome sequence (scaffold) was assembled from clean data that met the requirements after quality control. The assembled sequence was predicted, and the gene information of each sample was obtained (Steinberg et al., 2017). The short sequence assembly software SOAPdenovo v2.04^1^ was used to splice multiple k-mer parameters of the optimised sequence, and the optimal assembly result was obtained. After comparing reads to contig, the assembly results were locally assembled and optimised according to paired-end and overlap relationships. The sequencing reads were cut into smaller units, namely, k-mer. Heterozygosity, repeatability, size, presence of plasmids and genome contamination were assessed by calculating the k-mer depth and the ratio of each depth. The coding sequence (CDS) of the genome was predicted to obtain the nucleic acid sequence and amino acid sequence of the functional genes for subsequent functional and phylogenetic analysis (Zhao et al., 2008).

Basic functional annotations were performed on the predicted encoding genes, and functional annotations were performed with three databases (COG, GO and KEGG databases). Gene annotation was mainly based on the protein sequence alignment. The gene sequence was compared with each database to obtain the corresponding functional annotation information. COG is constructed based on the protein sequences of the completed genome sequencing species (Tatusov, 2001). COG databases can be compared for functional annotation, classification and protein evolution analysis of the predicted proteins. GO standardises the biological terms with different language descriptions and facilitates direct communication among scholars (Vesztrocy and Dessimoz, 2017). GO annotation includes the cellular component, molecular function and biological process. The rich pathway information in the KEGG database facilitates a systematic understanding of the biological functions of genes (e.g. metabolic pathways), genetic information and complex biological processes (e.g. cellular processes; Kanehisa, 2017).

## Results and Discussion

### Separation Results of Ester-Producing Yeast

**Figure 1:**
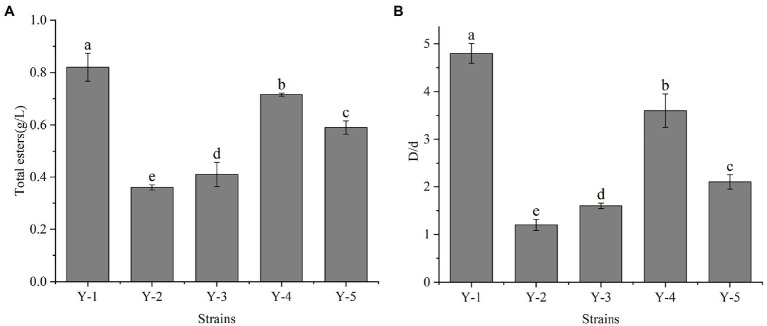
Determination of total ester content **(A)** and esterification ability of yeast **(B)**. Different letters indicate significant differences (*p* < 0.05).

Five yeast strains were isolated and purified from the Chinese liquor fermentation starter, and the strains were denoted as Y-1, Y-2, Y-3, Y-4 and Y-5. The strains were activated to prepare the seed solution. The seed solution was inoculated in the ester-producing medium at 10% inoculation and cultured at 28°C for 2 days. Among the five yeast strains, Y-1 had the highest total ester content, which was significantly higher than the other four strains (Figure 1A). According to the ratio between the diameter of the transparent circle (D) and the diameter of the colony (d), the esterification capacity of yeast was analysed. The greater the ratio, the greater the esterification capacity (Figure 1B). Among the five strains, the Y-1 strain had the most significant ratio of the fine circle diameter to the strain diameter, indicating that the strain had the strongest esterification decomposition and synthesis ability; therefore, the Y-1 strain was selected for follow-up tests.

### Morphological Observation and Molecular Identification of *Wickerhamomyces anomalus* Y-1

**Figure 2:**
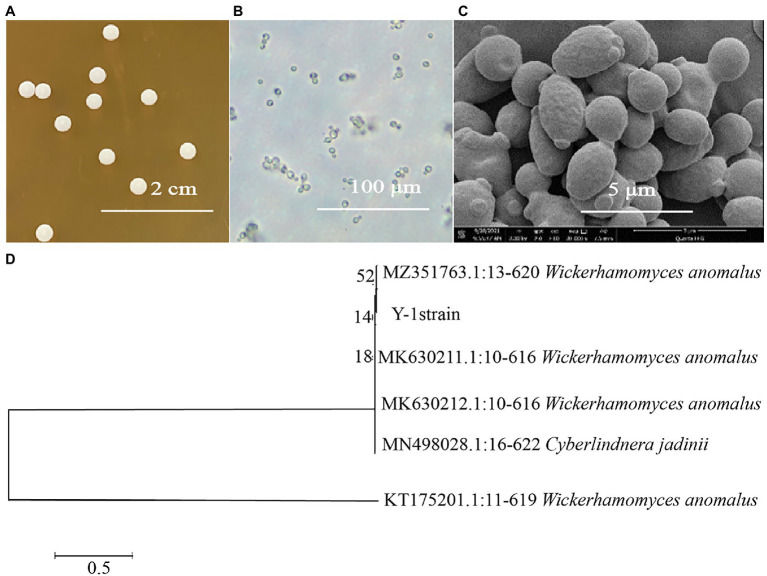
**(A)** Y-1 colony morphology; **(B)** Y-1 morphology under the microscope; **(C)** Y-1 morphology under the scanning electron microscope; and **(D)** A neighbour phylogenetic tree of Y-1 and 5 strains with high homology was established based on the 18S rDNA fragment of Y-1.

The colonies of *W. Anomalus* Y-1 were round, indicating a moist, smooth and typical yeast morphology (Figure 2A). A bud protruding from one end of the cell was observed under a 40× microscope and gradually divided into single cells (Figure 2B).

Scanning electron microscopy shows a bulge like a bud at one end of the cell, and some buds separated from the mother cell to form independent cells (Figure 2C). The obtained sequences were submitted to the Gene Bank for homology analysis. The results showed that strain Y-1 was most closely related to *W. anomalus*, so this yeast was *W. anomalus*. The phylogenetic tree was constructed using MEGA7.0 software (Figure 2D).

(Represents 1,000 bootstrap copied values calculated by MEGA7. The percentage of replicate trees in which the associated taxa clustered together in the bootstrap test (1,000 replicates) are shown above the branches, 0.5 represents the unit length of the value of the difference between organisms.)

### Flavoured Components of *Wickerhamomyces anomalus* Y-1

**Figure 3:**
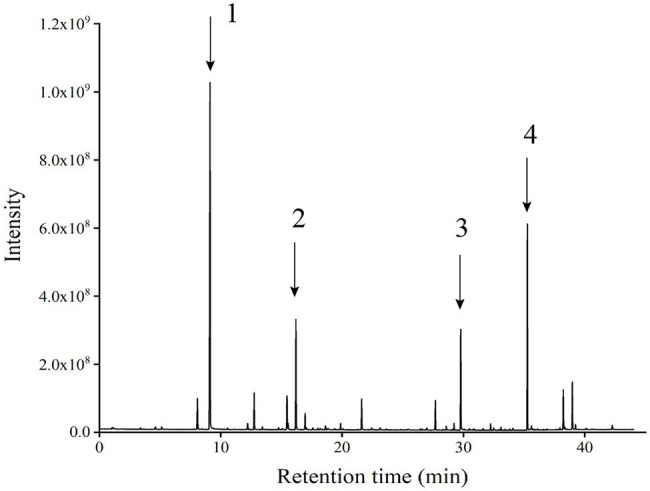
Chromatographic patterns of flavoured components in the liquid fermentation of strain Y-1 (Numbers 1, 2, 3 and 4 represent ethyl hexanoate, ethyl caprylate, phenyl ethanol and ethyl palmitate, respectively).

GC–MS analysis showed that strain Y-1 produced more than 40 flavoured components, including esters, alcohols, acids and aldehydes, of which 86% were esters. The chromatogram of flavoured components from liquid fermentation of strain Y-1 was shown in Figure 3. The content of ethyl hexanoate was the highest, accounting for 49.526% of the total flavour components, and the content was 381.75 μg/l, followed by ethyl palmitic acid, ethyl valerate and ethyl caprylic acid, the total volatile substance is 100% (Supplementary Table S1). In previous GC–MS analysis, some yeasts also produced esters, but more strains produced ethyl acetate than those that produced ethyl hexanoate (Christian et al., 2014; Zhang et al., 2020). Therefore, Y-1 can produce ethyl hexanoate, which is of great significance to improve the quality of Luzhou-flavoured liquor. In addition to more esters, the Y-1 strain could also produce alcohols, among which phenyl ethanol had the highest relative content, followed by isoamyl alcohol. This yeast could also create a small amount of 1-propanol, isobutanol and other higher alcohols. These alcohols could enhance the distinctive aroma of liquor in the appropriate range.

### Y-1 Genome Characteristics

The critical genes related to the metabolic pathway of Y-1 ethyl hexanoate were further studied by whole-genome prediction. Firstly, PE sequencing reads were used to select the intermediate high-quality sequencing region, and a certain length of K-mer base was taken to assess the genome size (Zhao et al., 2018). After quality control, the assembly results of the samples were analysed with the reads. Several genomic structure prediction software has been developed, such as Maker2 (Holt and Yandell, 2011), Barrnap 0.4.2^2^ and tRNAs can-SE V1.3.1.^3^ Maker2 software was used to predict the fungal genes. According to the assembly and prediction results, the genome sequence of strain Y-1 was composed of a chromosome with a total length of 15,127,803 bp (including chromosomes and plasmids). The average GC content of the whole sequence was 34.56%. Using the eukaryotic genome annotation analysis tool (MAKER2 + V2.31.9), 7,024 CDS were annotated, including 69 tRNAs and one rRNA in the whole-genome sequence. Across the whole genome, 3,981 genes had specific pathways compared with the KEGG database, and 5,966 genes had annotated COG information compared with the EggNOG database (Table 1). The data of this sequence were submitted to the GeneBank database of the National Biotechnology Information Centre (NCBI) under the name *W. anomalus* Y-1, and the obtained biological project ID was PRJNA770424. These genomic features were similar to other members of the genus *Wickerhamomyces*, such as *Wickerhamomy anomalus* NRRL Y-366\u20138 (GenBank accession number: GCA_001661255.1, genome size 14.15Mbp, GC content 35%, 6,421 CDSs and 154 tRNAs).

**Table 1:** Genome features of strain *Wickerhamomyces anomalus* Y-1.

Sample name	Y-1
Genome size (bp)	15,127,803
Scaffold No.	1,471
GC content (%)	34.56
CDS No.	7,024
Gene total length (bp)	11,118,812
Gene average length (bp)	1,582.97
Genes of KEGG	3,981
Genes of COG	5,966
tRNA No.	69
rRNA No.	1

### Genome Annotation Analysis

The genome needed to be annotated on the database to determine the identified gene function and its descriptive information. Genome annotation can reflect the functional classification of the filtered gene set, which is of great significance for the subsequent study of cell growth and metabolism. For genome annotation comparison, three high-quality databases, namely, COG, GO and KEGG, were selected.

**Figure 4:**
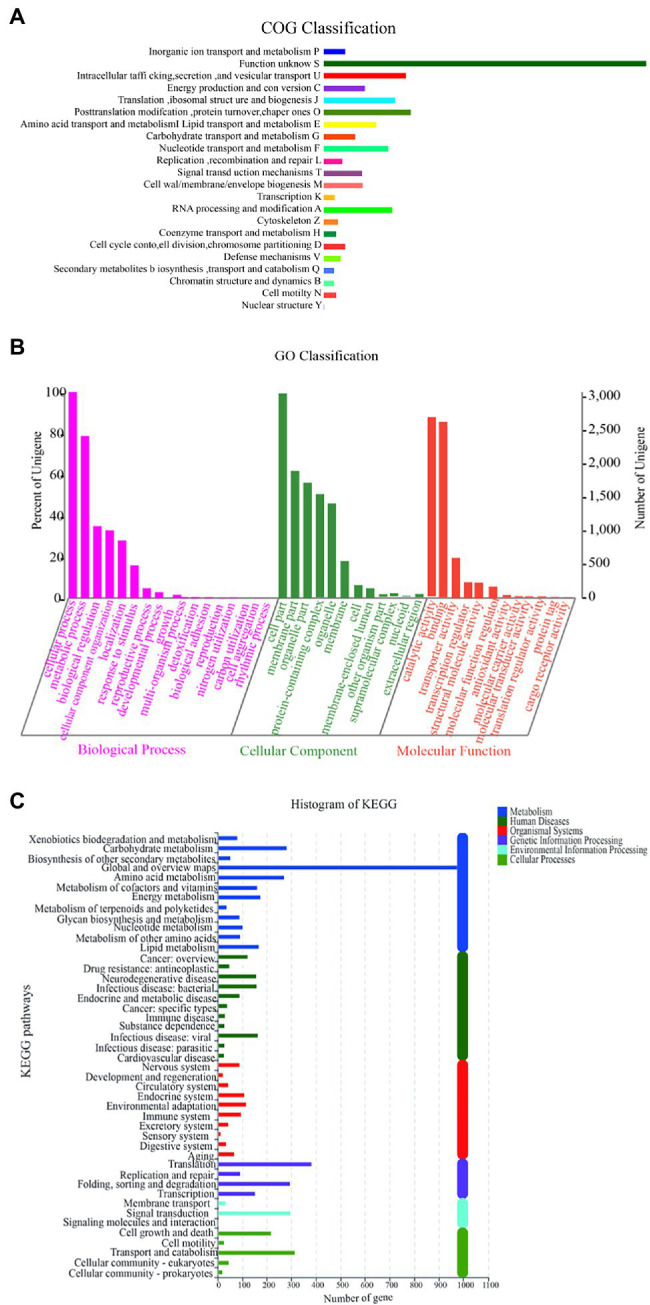
Diagram of COG function annotation analysis **(A)**; Diagram of GO/IPR function annotation analysis **(B)**; and Diagram of KEGG function annotation analysis **(C)**.

**Figure 5:**
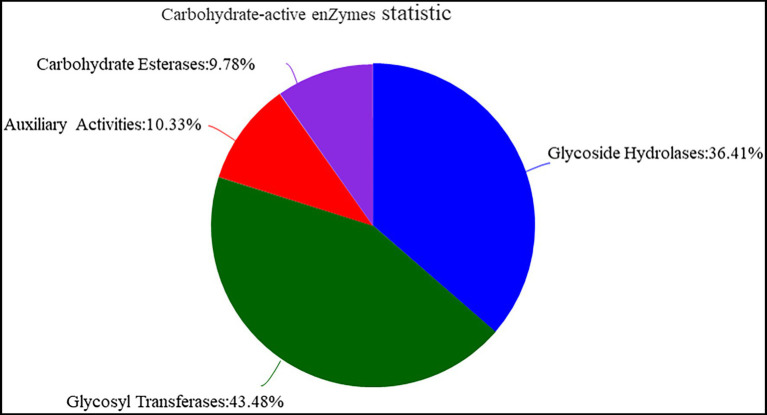
Analysis of carbohydrate-active enzyme annotation.

The function of Y-1 was studied by annotating the whole genome. Functional analysis was performed using COG (Figure 4A; Supplementary Tables S2 and S3), GO (Figure 4B; Supplementary Tables S4 and S5) and KEGG (Figure 4C; Supplementary Table S6). Based on the analysis of three annotated databases, genes with high abundance in the Y-1 genome included amino acid transport and metabolism, carbohydrate transport and metabolism. In the fermentation of Luzhou-flavoured liquor, grains rich in carbohydrates and proteins, such as sorghum and wheat, were used as raw materials. The annotation results showed that the strain could degrade starch, indicating that the strain could adapt to the particular environment of liquor fermentation. Genome annotation is of great significance for future research, especially in exploring the biosynthesis of flavoured compounds in the genome. In addition to the annotations from these three databases, the annotations were based on the Carbohydrate-Active Enzyme Database (Vincent et al., 2014).^4^ This database annotates enzymes that can synthesise or decompose complex carbohydrates. According to the amino acid sequence similarity of a protein domain, carbohydrate-active enzymes are divided into different protein families. The classification and related information of the synthesis, metabolism and transport of the carbon compound and other enzymes are provided. The annotated results showed that glycosyltransferases accounted for 43.48%, glycosidases accounted for 36.41%, carbohydrate esterases accounted for 9.78% and coenzymes accounted for 10.33% (Figure 5; Supplementary Table S7). Among these compounds, carbohydrate esterases were mostly related to the synthesis of esters, such as isoamyl acetate hydrolase (EC3.1.1.-) in the CE12 family annotated by gene 1962 and carboxylesterase (EC3.1.1.1) in the CE1 family annotated by gene 4,180, which played a crucial role in the synthesis and hydrolysis of esters.

## Discussion

Two kinds of esters are used in liquor making, namely, acetate and medium-chain fatty acid ethyl ester (MCFA). Acetate mainly includes ethyl acetate, isoamyl acetate and phenyl ethyl acetate. Enzymes related to acetate synthesis have been reported, for example: using AATaseII by purifying acetate synthase led to the identification of three acyltransferases, namely, AATaseI encoded by *ATF1,* LG-AATASei encoded by *lgATF1* and AATaseII encoded by *ATF2* (Verstrepen et al., 2003; Lilly et al., 2006). Studies have shown that *ATF1* and *ATF2* exist in the *S. cerevisiae* cells, while *lgATF1* is only found in *Streptomyces*. Homology search of the *S. cerevisiae* genome did not show other homology genes with *ATF1* or *ATF2* (Yoshimoto et al., 1999; Van et al., 2008).

**Figure 6:**
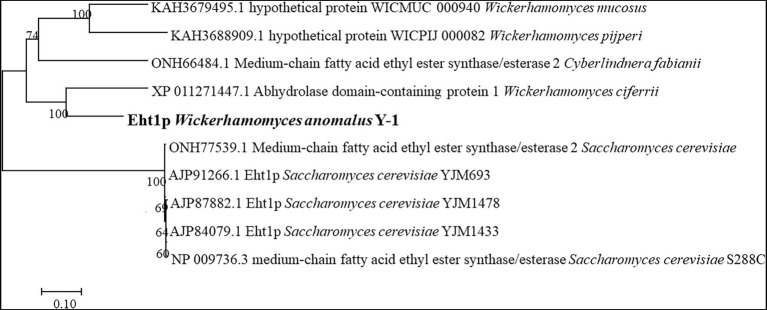
Phylogenetic tree of Eht1p homologous fungi established by MEGA7.

MCFA mainly includes ethyl hexanoate, ethyl capryate, the synthesis and regulation of MCFA are rarely studied. Malcorps and Dufour (2010) speculated that in addition to Atf1p, LG-ATF1P and Atf2p, other alcoholyltransferases should be present in the yeast group (Mason and Dufour, 2000; Malcorps and Dufour, 2010). One possible alcohol transferase was hypothesised to be alcohol acetyltransferase (Eht1p; ko7019). As a putative acyltransferase similar to Eeb1p, this enzyme is involved in synthesising MCFA esters containing ethyl hexanoate. Ethyl hexanoate is the most important ester in Luzhou-flavoured liquor, which is mainly synthesised by the alcohol acyltransferase pathway in *S. cerevisiae*, catalysing ethyl hexanoate from ethanol and hexanoyl-coenzyme A under the catalytic action of alcohol acyltransferase. The synthesis rate depended on the acyltransferase activity level and the acyl-CoA content of the substrate. Saerens (2006) reported that *EHT1* and *EEB1* of *S. cerevisiae* could encode acyltransferase, which is a key gene for the ethyl ester synthesis of MCFAs in yeast (Van et al., 2008). The acyl transferases encoded by *EHT1* and *EEB1* genes catalyse the formation of ethyl hexanoate from hexanoyl-CoA and alcohols. Sofie et al. reported that the expression of the *EHT1* gene can improve the synthesis ability of ethyl hexanoate and ethyl capryate (Saerens, 2006). In this study, according to the whole-genome study of Y-1, it was found that the pathway ko7019 of gene 6,340 contained Eht1p. Homology analysis of the amino acid sequence of Eht1p showed that Eht1p was similar to the homology sequences of different yeasts in the known database. The phylogenetic correlation between Eth1p sequences of this species was estimated by the adjacency programme (Figure 6).

(The percentage of replicate trees in which the associated taxa clustered together in the bootstrap test (1,000 replicates) are shown above the branches. The branch lengths are in the same units as those of the evolutionary distances used to infer the phylogenetic tree. A 0.10 represents the unit length of the difference value between sequences. The analysis involved 10 amino acid sequences.)

**Figure 7:**
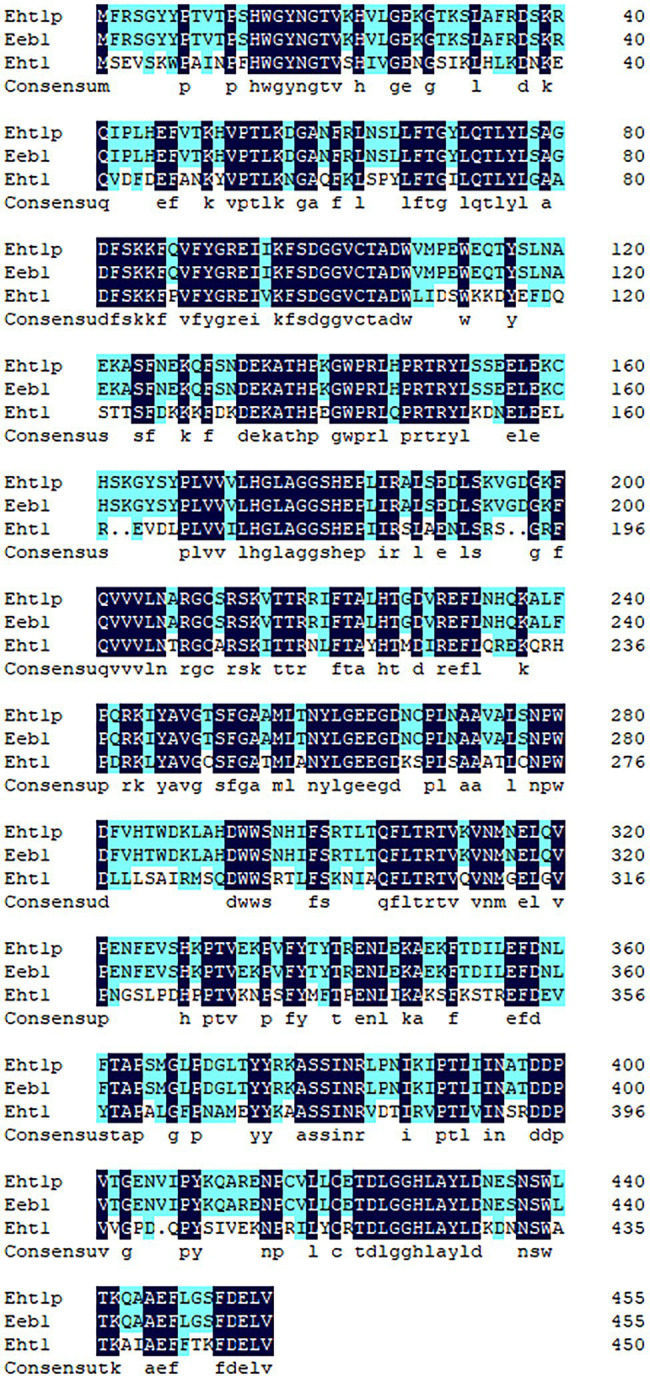
Multiple comparisons of Eht1p, Eeb1 and Eht1 amino acid sequences of *Wickerhamomyces anomalus* Y-1. Non-conservative residues are black text on a white background. Conservative same residue as white text on dark blue background. A similar residue is a black text on a light blue background.

The amino acid sequences of Eht1p were compared with those of Eht1 and Eeb1. To find the conserved areas of homologous sequences and possible vital sequences, the same amino acid residue base was 51.75% in the multiple alignment sequence (Figure 7). This result confirmed the conjecture of Malcorps and Dufour (2010) that ethyl hexanoate is synthesised by catalysing ethanol and hexanoyl-CoA in the presence of alcohol hexacyltransferase. The synthesis of ethyl hexanoate of *W. anomalus* Y-1 in liquor brewing could be summarised as follows: starch and other macromolecules were converted into glucose by highly active degradation enzymes into cells. The glucose was absorbed and utilised by yeast cells to generate primary metabolites and intermediates. Ethanol and organic acids were produced through the citric acid cycle. Hexanoic acid mainly produced by hexanoic acid bacteria produced the hexanoyl coenzyme under the action of alcohol hexanoyl transferase (Yan and Dong, 2018). Then, hexanoyl coenzyme and ethanol produced ethyl hexanoate under the action of esterase. Ethyl hexanoate was the prominent aroma of Luzhou-flavoured liquor, and its content determined the quality of Luzhou-flavoured liquor.

In addition to the discovery of Eht1p, the full-length functional annotation of Y-1 enabled the identification of other key metabolic enzyme-encoding genes, such as genes related to carbohydrate metabolism; for example, 6-phosphate glucuronidase (EC3.1.1.31) encoding pgi (gene ID: 25796, protein sequence number: NP_036220), which acts on the ester bond belonging to carboxylesterase hydrolase and participates in the pentose phosphate and carbon metabolism pathways. The gloB-encoded hydrolase (EC3.1.2.6) also acts on the ester bond, which belongs to this hydrolase and participates in pyruvate metabolism. Inositol phosphate esterase encoding such (gene ID: 947285, protein sequence number NP_417028) belongs to phosphate monoester hydrolase, which acts on the ester bond and participates in the synthesis of secondary metabolites and carbohydrate metabolism. These enzymes are key enzymes for the synthesis of esters. Functional annotations had also identified some genes in amino acid metabolism (e.g. *argE*, *ydfG*, *metB* and *ansA*), cofactors and vitamin metabolism (e.g. *gltX*, *phoD*, *wrbA* and *phoA*), terpenes and polyketides metabolism (e.g. *mvaD*, *tktA*, *mvaK2* and *FCLY*) and lipid metabolism (e.g. *aslA*, *pgsA*, *ERG1* and *adhP*). The enzymes expressed by these genes are often involved in the degradation of macromolecular substances, such as proteins and peptides, some of which have good acid and sugar resistance and can adapt to the unique environment of liquor brewing.

*Wickerhamomyces anomalus* Y-1 with strong esterification ability was selected from the Daqu. To study the synthesis of mechanism for ethyl hexanoate in the yeast, whole-genome sequencing and assembly were performed. Functional annotation was conducted using the sequencing results. Annotation results showed closely related mechanism of ethyl hexanoate to synthetic alcohol acyltransferase, and MCFA ethyl ester with known transferase was used for sequence alignment, with the same base of amino acid residues of 51.75%. In this study, the key genes for ester synthesis were identified by whole-genome sequencing of *W. anomalus* Y-1 strain. In order to prove the promoting effect of this gene on liquor flavour during liquor brewing, some validation experiments are needed.

## Data Availability Statement

The datasets presented in this study can be found in online repositories. The names of the repository/repositories and accession number(s) can be found in the article/Supplementary Material.

## Author Contributions

XS and XW: data curation, conceptualization, writing—review and editing, supervision, and methodology. XH and QT: conceptualization and writing—review and editing. MH: writing—review and editing, funding acquisition, project administration, and supervision. All authors contributed to the article and approved the submitted version.

## Funding

This work was financially supported by the research and application of microflora of yujiu, a sub-project of major science and technology projects of Henan province, China (181100211400–8). Henan University of Technology (no. 31401184).

## Conflict of Interest

The authors declare that the research was conducted in the absence of any commercial or financial relationships that could be construed as a potential conflict of interest.

## Publisher’s Note

All claims expressed in this article are solely those of the authors and do not necessarily represent those of their affiliated organizations, or those of the publisher, the editors and the reviewers. Any product that may be evaluated in this article, or claim that may be made by its manufacturer, is not guaranteed or endorsed by the publisher.

## Footnotes

^1^ https://github.com/topics/soapdenovo

^2^ https://github.com/tseemann/barrnap

^3^ http://lowelab.Ucsc.edu/tRNAsc

^4^ http://www.cazy.org/

## References

[ref1] Cheng N. Koda K. Tamai Y. Yamamoto Y. Takasuka T. E. Uraki Y. (2017). Optimization of simultaneous saccharification and fermentation conditions with amphipathic lignin derivatives for concentrated bioethanol production. Bioresour. Technol. 232, 126–132. doi: 10.1016/j.biortech.2017.02.018, PMID: 28214699

[ref2] Christian L. Thanet U. Thomas B. (2014). Perspectives for the biotechnological production of ethyl acetate by yeasts. Appl. Microbiol. Biotechnol. 98, 5397–5415. doi: 10.1007/s00253-014-5765-9, PMID: 24788328

[ref3] Ding X. F. Wu C. D. Huang J. Zhou R. Q. (2015). Changes in volatile compounds of chinese Luzhou-flavor liquor during the fermentation and distillation process. J. Food Sci. 80, C2373–C2381. doi: 10.1111/1750-3841.13072, PMID: 26444440

[ref4] Fan W. L. Shen H. Y. Xu Y. (2011). Quantification of volatile compounds in Chinese soy sauce aroma type liquor by stir bar sorptive extraction and gas chromatography-mass spectrometry. J. Sci. Food Agric. 91, 1187–1198. doi: 10.1002/jsfa.4294, PMID: 21384368

[ref5] Farooq S. Ganai S. A. Ganai B. A. Mohan S. Uqab B. Nazir R. (2021). Molecular characterization of lipase from a psychrotrophic bacterium pseudomonas sp. CRBC14. Curr. Genet. 68, 243–251. doi: 10.1007/S00294-021-01224-W 34837516

[ref6] Furukawa K. Yamada T. Mizoguchi H. Hara S. (2003). Increased ethyl caproate production by inositol limitation in Saccharomyces cerevisiae. J. Biosci. Bioeng. 95, 448–454. doi: 10.1016/S1389-1723(03)80043-9, PMID: 16233438

[ref7] He G. Q. Huang J. Wu C. D. Jin Y. Zhou R. Q. (2019a). Bioturbation effect of fortified Daqu on microbial community and flavor metabolite in Chinese strong-flavor liquor brewing microecosystem. Food Res. Int. 129:108851. doi: 10.1016/j.foodres.2019.108851 32036891

[ref8] He G. Q. Huang J. Zhou R. Q. Wu C. D. Jin Y. (2019b). Effect of fortified daqu on the microbial community and flavor in chinese strong-flavor liquor brewing process. Front. Microbiol. 10:56. doi: 10.3389/fmicb.2019.00056, PMID: 30761106 PMC6361764

[ref9] Holt C. Yandell M. (2011). MAKER2: An annotation pipeline and genome-database management tool for second-generation genome projects. BMC Bioinform. 12, 1–14. doi: 10.1186/1471-2105-12-491 PMC3280279 22192575

[ref10] Hong J. X. Tian W. J. Zhao D. R. (2020). Research progress of trace components in sesame-aroma type of baijiu. Food Res. Int. 137:109695. doi: 10.1016/j.foodres.2020.109695, PMID: 33233269

[ref11] Jin G. Y. Zhu Y. Xu Y. (2017). Mystery behind Chinese liquor fermentation. Trends Food Sci. Tech. 63, 18–28. doi: 10.1016/j.tifs.2017.02.016

[ref12] Kanehisa M. (2017). Enzyme annotation and metabolic reconstruction using KEGG. Methods Mol. Biol. 1611, 135–145. doi: 10.1007/978-1-4939-7015-5_11, PMID: 28451977

[ref13] Kusano M. Sakai Y. Kato N. Yoshimoto H. Tamai Y. (1998). Hemiacetal dehydrogenation activity of alcohol dehydrogenases in Saccharomyces cerevisiae. Biosci. Biotech. Bioch. 62, 1956–1961. doi: 10.1271/bbb.62.1956 9836432

[ref14] Li W. W. Fan G. S. Fu Z. L. Wang W. H. Li X. T. (2019). Effects of fortification of daqu with various yeasts on microbial community structure and flavor metabolism. Food Res. Int. 129:108837. doi: 10.1016/j.foodres.2019.108837, PMID: 32036879

[ref15] Lilly M. Bauer F. F. Lambrechts M. G. Swiegers J. H. Cozzolino D. Pretorius I. S. (2006). The effect of increased yeast alcohol acetyltransferase and esterase activity on the flavour profiles of wine and distillates. Yeast 23, 641–659. doi: 10.1002/yea.1382, PMID: 16845703

[ref16] Ling Y. X. Li W. Y. Tong T. Li Z. M. Wang Y. G. (2020). Assessing the microbial communities in four different daqus by using PCR-DGGE, PLFA, and biology analyses. Pol. J. Microbiol. 69, 1–11. doi: 10.33073/pjm-2020-004, PMID: 32067441 PMC7256838

[ref17] Liu H. L. Sun B. G. (2018). Effect of fermentation processing on the flavor of Baijiu. J. Agr. Food Chem. 66, 5425–5432. doi: 10.1021/acs.jafc.8b00692, PMID: 29751730

[ref18] Lu M. M. Zhou W. C. Ji F. (2020). Profiling prokaryotic community in pit mud of Chinese strong-aroma type liquor by using oligotrophic culturing. Int. J. Food Microbiol. 337:108951. doi: 10.1016/j.ijfoodmicro.2020.108951 33202299

[ref19] Malcorps P. Dufour J. P. (2010). Short-chain and medium-chain aliphatic-ester synthesis in Saccharomyces cerevisiae. FEBS J. 210, 1015–1022. doi: 10.1111/j.1432-1033.1992.tb17507.x, PMID: 1483449

[ref20] Mason A. B. Dufour J. I. (2000). Alcohol acetyltransferases and the significance of ester synthesis in yeast. Yeast 16, 1287–1298. 11015726 10.1002/1097-0061(200010)16:14<1287::AID-YEA613>3.0.CO;2-I

[ref21] Papamichael P. S. A. A. (2013). Advances in lipase-catalyzed esterification reactions. Biotechnol. Adv. 31, 1846–1859. doi: 10.1016/j.biotechadv.2013.08.006, PMID: 23954307

[ref22] Qiu Y. Fang F. Zhou X. Zhang L. (2016). Characterization of arginine utilization strains from fermented grains and evaluation of their contribution to citrulline accumulation in Chinese Luzhou-flavor spirits. Acta Microbiol Sin. 56, 1638–1646. doi: 10.13343/j.cnki.wsxb.20160007, PMID: 29741826

[ref23] Reyes-Sánchez F. J. Páez-Lerma J. Rojas-Contreras J. A. López-Miranda J. Reinhart-Kirchmayr M. (2020). Study of the enzymatic capacity of kluyveromyces marxianus for the synthesis of esters. J. Mol. Microb. Biotech 29, 1–9. doi: 10.1159/000507551 32325454

[ref24] Rojo M. C. Palazzolo C. T. Cuello R. González M. Guevara F. Ponsone M. L.. (2017). Incidence of osmophilic yeasts and Zygosaccharomyces rouxii during the production of concentrate grape juices. Food Microbiol. 64, 7–14. doi: 10.1016/j.fm.2016.11.017, PMID: 28213037

[ref25] Saerens S. (2006). The saccharomyces cerevisiae EHT1 and EEB1 genes encode novel enzymes with medium-chain fatty acid ethyl ester synthesis and hydrolysis capacity. J. Biol. Chem. 281, 4446–4456. doi: 10.1074/jbc.M512028200, PMID: 16361250

[ref26] Steinberg K. M. Schneider V. A. Alkan C. Montague M. J. Warren W. C. Church D. M.. (2017). Building and improving reference genome assemblies. P. IEEE. PP(3), 1–14. doi: 10.1109/JPROC.2016.2645402

[ref27] Tatusov R. L. (2001). The COG database: new developments in phylogenetic classification of proteins from complete genomes. Nucleic Acids Res. 29, 22–28. doi: 10.1093/nar/29.1.22, PMID: 11125040 PMC29819

[ref28] Van D. M. Saerens M. G. Verstrepen K. J. (2008). Flavour formation in fungi: characterisation of KlAtf, the *Kluyveromyces lactis* orthologue of the *Saccharomyces cerevisiae* alcohol acetyltransferases Atf1 and Atf2. Appl. Microbiol. Biotechnol. 78, 783–792. doi: 10.1007/s00253-008-1366-9, PMID: 18309479

[ref29] Verstrepen K. J. Laere S. V. Vanderhaegen B. Derdelinckx G. Dufour J. P. Pretorius I. S.. (2003). Expression levels of the yeast alcohol acetyltransferase genes ATF1, Lg-ATF1, and ATF2 control the formation of a broad range of volatile esters. Appl. Environ. Microbiol. 69, 5228–5237. doi: 10.1016/j.cej.2007.03.005, PMID: 12957907 PMC194970

[ref30] Vesztrocy A. W. Dessimoz C. (2017). A gene ontology tutorial in python. Methods Mol. Biol. 1446, 221–229. doi: 10.1007/978-1-4939-3743-1_16 27812946

[ref31] Vincent L. Hemalatha G. R. Elodie D. Coutinho P. M. Bernard H. (2014). The carbohydrate-active enzymes database (CAZy) in 2013. Nucleic Acids Res. 42, D490–D495. doi: 10.1093/nar/gkt1178, PMID: 24270786 PMC3965031

[ref32] Wang D. Q. Chen L. Q. Yang F. Wang H. Y. Wang L. (2019). Yeasts and their importance to the flavour of traditional Chinese liquor: A review. J. Brewing. 125, 214–221. doi: 10.1002/jib.552

[ref33] Wang W. H. Fan G. S. Li X. T. Fu Z. L. Liang X. Sun B. G. (2020). Application of Wickerhamomyces anomalus in simulated solid-state fermentation for baijiu production: changes of microbial community structure and flavor metabolism. Front. Microbiol. 11:2994. doi: 10.3389/fmicb.2020.598758 PMC7728721 33329488

[ref34] Wang Y. S. Li B. Dong H. Huang X. D. Chen R. Y. Chen X. J.. (2018). Complete genome sequence of clostridium kluyveri JZZ applied in chinese strong-flavor liquor production. Curr. Microbiol. 75, 1429–1433. doi: 10.1007/s00284-018-1539-4, PMID: 30030563

[ref35] Wang H. Y. Yan X. U. Fan L. I. Jin Y. G. (2008). Enzymatic characteristics of alcohol acetyltransferase from hanseniaspora valbyensis, a non-saccharomyces yeast. J. Food Biochem. 32, 506–520. doi: 10.1111/j.1745-4514.2008.00180.x

[ref36] Wei Y. Zou W. Shen C. H. Yang J. G. (2020). Basic flavor types and component characteristics of Chinese traditional liquors: A review. J. Food Sci. 85, 4096–4107. doi: 10.1111/1750-3841.15536, PMID: 33190291

[ref37] Wolter H. Lietz P. Beubler A. (1966). Influence of temperature and yeast strain on the formation of fermentation amyl alcohol, isobutanol and ethyl acetate in fermenting malt wort. Folia Microbiol. 11, 210–214. doi: 10.1007/BF02901434, PMID: 5912715

[ref38] Xu Y. Q. Sun B. G. Fan G. S. Teng C. Li X. T. (2017). The brewing process and microbial diversity of strong flavour Chinese spirits: A review. J. Inst. Brewing. 123, 5–12. doi: 10.1002/jib.404

[ref39] Yan S. Dong D. (2018). Improvement of caproic acid production in a clostridium kluyveri H068 and methanogen 166 co-culture fermentation system. AMB Express 8:175. doi: 10.1186/s13568-018-0705-1, PMID: 30361817 PMC6202304

[ref41] Yang Y. R. Zhong H. Y. Yang T. Lan C. H. (2020). Characterization of the key aroma compounds of a sweet rice alcoholic beverage fermented with *Saccharomycopsis fibuligera*. J Food Sci Technol. 58, 1–13. doi: 10.1007/s13197-020-04833-4 PMC8357862 34471299

[ref42] Yin X. Yoshizaki Y. Ikenaga M. Han X. L. Okutsu K. Futagami T.. (2020). Manufactural impact of the solid-state saccharification process in rice-flavor baijiu production – ScienceDirect. J. Biosci. Bioeng. 129, 315–321. doi: 10.1016/j.jbiosc.2019.09.017, PMID: 31718882

[ref43] Yoshimoto H. Fujiwara D. Momma T. Tanaka K. Sone H. Nagasawa N.. (1999). Isolation and characterization of the ATF2 gene encoding alcohol acetyltransferase II in the bottom fermenting yeast saccharomyces pastorianus. Yeast 15, 409–417. doi: 10.1002/(SICI)1097-0061(19990330)15:53.0.CO;2-Q, PMID: 10219999

[ref44] Zhang S. J. Guo F. Yan W. Dong W. L. Jiang M. (2020). Perspectives for the microbial production of ethyl acetate. Appl. Microbiol. Biot. 104, 7239–7245. doi: 10.1007/s00253-020-10756-z, PMID: 32656615

[ref45] Zhang G. Q. Lin Y. P. He P. Li L. Wang Q. H. Ma Y. (2014). Characterization of the sugar alcohol-producing yeast *Pichia anomala*. J. Ind. Microbiol. Biot. 41, 41–48. doi: 10.1007/s10295-013-1364-5, PMID: 24170383

[ref46] Zhang H. M. Meng Y. J. Wang Y. L. Zhou Q. W. Xing X. H. (2019). Prokaryotic communities in multidimensional bottom-pit-mud from old and young pits used for the production of Chinese strong-flavor Baijiu – science direct. Food Chem. 312:126084. doi: 10.1016/j.foodchem.2019.126084 31901820

[ref47] Zhao X. M. Wang Y. Chen L. Aihara K. (2008). Gene function prediction using labeled and unlabeled data. BMC Bioinform. 9:57. doi: 10.1186/1471-2105-9-57, PMID: 18221567 PMC2275242

[ref48] Zhao L. Xie J. Bai L. Chen W. Wang M. Zhang Z.. (2018). Mining statistically-solid k-mers for accurate NGS error correction. BMC Genomics 19:912. doi: 10.1186/s12864-018-5272-y, PMID: 30598110 PMC6311904

[ref49] Zheng J. Wu C. D. Huang J. Zhou R. Q. Liao X. P. (2015). Spatial distribution of bacterial communities and related biochemical properties in Luzhou-flavor liquor-fermented grains. J. Food Sci. 79, M2491–M2498. doi: 10.1111/1750-3841.12697, PMID: 25393984

[ref50] Zou W. Ye G. B. Zhang K. Z. (2018). Diversity, function, and application of clostridium in Chinese strong flavor baijiu ecosystem: A review. J. Food Sci. 83, 1193–1199. doi: 10.1111/1750-3841.14134, PMID: 29660763

