# Supplementary material for: Gene Mining and Flavour Metabolism Analyses of Wickerhamomyces anomalus Y-1 Isolated From a Chinese Liquor Fermentation Starter
Source: Front Microbiol. 2022 May 2;13:891387. doi: 10.3389/fmicb.2022.891387 (PMC9108772; doi:10.3389/fmicb.2022.891387)
Supplement: Supplementary file 8 [file Table_2.DOC]

The raw dateset of figure3

gc-ms

Chromatogram Data Information:

Time Min. (min) 0.034052

Time Max. (min) 44.010488

Data Points 12931

Detector MSDevice

Generating Data System Chromeleon 7.2.10 Build 23925 (cfd1e7af542fd56c07b8e0c2791055c459cfcf14)

Exporting Data System Chromeleon 7.2.10.0

Operator Instrument Controller

Signal Quantity Intensity

Signal Unit counts

Signal Min. 6,091,234.900846

Signal Max. 1,027,531,999.339830

Channel TIC

Driver Name Thermo.MassSpectrometer

Channel Type Evaluation

Min. Step (s) 0.204

Max. Step (s) 0.2041

Average Step (s) 0.20406699

Signal Parameter Information:

Signal Info

Chromatogram Data:

Time (min) Step (s) Value (counts)

0.034052 n.a. 10,088,160.085053

0.037453 0.2041 9,937,966.924854

0.040855 0.2041 10,142,749.992415

0.044255 0.204 10,296,870.994191

0.047657 0.2041 10,091,266.419466

0.051058 0.2041 10,376,790.770671

0.054458 0.204 10,458,894.519630

0.057860 0.2041 10,320,149.186601

0.061262 0.2041 10,459,405.829545

0.064662 0.204 10,528,997.841935

0.068063 0.2041 10,474,018.499255

0.071465 0.2041 10,489,063.604521

0.074865 0.204 10,536,328.037164

0.078267 0.2041 10,374,081.969225

0.081668 0.2041 10,393,837.410149

0.085068 0.204 10,610,872.870087

0.088470 0.2041 10,551,570.917567

0.091872 0.2041 10,402,248.529428

0.095273 0.2041 10,356,136.042507

0.098673 0.204 10,307,378.593856

0.102075 0.2041 10,293,542.990593

0.105477 0.2041 10,518,486.411038

0.108877 0.204 10,689,449.406635

0.112278 0.2041 10,438,068.474330

0.115680 0.2041 10,172,983.696998

0.119080 0.204 10,381,667.342433

0.122482 0.2041 10,513,901.509638

0.125883 0.2041 10,308,317.696406

0.129283 0.204 10,419,650.850500

0.132685 0.2041 10,433,198.611848

0.136087 0.2041 10,206,025.593238

0.139487 0.204 10,076,543.943077

0.142888 0.2041 9,985,607.558107

0.146290 0.2041 10,081,956.135605

0.149690 0.204 10,387,474.361262

0.153092 0.2041 10,432,903.024940

0.156493 0.2041 10,268,804.639042

0.159893 0.204 10,334,573.105429

0.163295 0.2041 10,272,916.861183

0.166697 0.2041 10,236,409.966326

0.170097 0.204 10,292,551.422249

0.173498 0.2041 10,188,884.888727

0.176900 0.2041 10,347,430.664157

0.180300 0.204 10,499,595.635564

0.183702 0.2041 10,507,635.520090

0.187103 0.2041 10,477,164.976330

0.190503 0.204 10,110,415.079951

0.193905 0.2041 10,130,824.995547

0.197307 0.2041 10,381,309.153247

0.200707 0.204 10,372,249.522059

0.204108 0.2041 10,411,924.557005

0.207510 0.2041 10,430,214.696259

0.210910 0.204 10,506,352.329547

0.214312 0.2041 10,658,525.443658

0.217713 0.2041 10,639,989.972960

0.221113 0.204 10,452,331.411072

0.224515 0.2041 10,385,392.575055

0.227917 0.2041 10,336,193.380604

0.231317 0.204 10,317,169.015929

0.234718 0.2041 10,378,736.811577

0.238120 0.2041 10,303,157.835876

0.241520 0.204 10,154,624.425871

0.244922 0.2041 10,236,026.547757

0.248323 0.2041 10,521,299.343435

0.251723 0.204 10,501,549.226215

0.255125 0.2041 10,272,977.431568

0.258527 0.2041 10,470,017.340417

0.261927 0.204 10,432,737.902011

0.265328 0.2041 10,141,601.800258

0.268730 0.2041 10,407,920.018012

0.272130 0.204 10,320,961.899388

0.275532 0.2041 10,195,824.868820

0.278933 0.2041 10,360,131.212218

0.282333 0.204 10,269,544.556687

0.285735 0.2041 10,171,905.107795

0.289137 0.2041 10,102,820.670554

0.292537 0.204 10,404,940.372509

0.295938 0.2041 10,658,518.961595

0.299340 0.2041 10,418,286.222391

0.302740 0.204 10,265,037.734309

0.306142 0.2041 10,311,038.475162

0.309543 0.2041 10,379,115.703066

0.312943 0.204 10,325,201.832537

0.316345 0.2041 10,145,656.115053

0.319747 0.2041 10,149,677.137708

0.323147 0.204 10,002,098.120229

0.326548 0.2041 10,005,110.766767

0.329950 0.2041 10,125,509.064821

0.333350 0.204 10,060,372.858751

0.336752 0.2041 10,269,178.726318

0.340153 0.2041 10,272,257.512053

0.343553 0.204 10,310,135.082340

0.346955 0.2041 10,386,349.414479

0.350357 0.2041 10,333,474.693992

0.353757 0.204 10,559,723.957598

0.357158 0.2041 10,587,603.739641

0.360560 0.2041 10,181,274.462279

0.363960 0.204 9,919,373.786813

0.367362 0.2041 10,211,368.893863

0.370763 0.2041 10,397,608.046980

0.374163 0.204 10,269,149.269618

0.377565 0.2041 10,264,921.492491

0.380967 0.2041 10,204,762.347274

0.384367 0.204 10,266,860.142286

0.387768 0.2041 10,481,426.675908

0.391170 0.2041 10,110,931.771704

0.394570 0.204 9,891,286.384096

0.397972 0.2041 10,121,106.944310

0.401373 0.2041 10,353,707.191101

0.404773 0.204 10,400,769.197589

0.408175 0.2041 10,115,217.634962

0.411577 0.2041 10,148,407.454978

0.414977 0.204 10,244,405.023771

0.418378 0.2041 9,975,204.952421

0.421780 0.2041 9,866,174.554831

0.425180 0.204 10,126,226.733616

0.428582 0.2041 10,085,064.017667

0.431983 0.2041 10,069,795.760427

0.435383 0.204 10,220,073.494533

0.438785 0.2041 10,033,824.041869

0.442187 0.2041 10,049,497.351666

0.445587 0.204 10,025,820.228580

0.448988 0.2041 9,876,439.576372

0.452390 0.2041 10,056,702.419731

0.455792 0.2041 9,926,945.459201

0.459192 0.204 9,776,931.872546

0.462593 0.2041 10,140,546.751038

0.465995 0.2041 10,205,193.267769

0.469395 0.204 10,134,221.286758

0.472797 0.2041 10,131,992.155158

0.476198 0.2041 10,020,816.319903

0.479598 0.204 10,406,857.914486

0.483000 0.2041 10,520,785.790124

0.486402 0.2041 10,204,394.421864

0.489802 0.204 10,323,400.132648

0.493203 0.2041 10,166,969.387714

0.496605 0.2041 9,977,431.802378

0.500005 0.204 10,184,610.390348

0.503407 0.2041 10,201,043.494760

0.506808 0.2041 10,201,523.457990

0.510208 0.204 10,041,374.605760

0.513610 0.2041 10,070,825.320988

0.517012 0.2041 10,295,232.459391

0.520412 0.204 10,137,978.853642

0.523813 0.2041 10,128,748.488862

0.527215 0.2041 10,254,210.393593

0.530615 0.204 10,079,774.848779

0.534017 0.2041 10,076,348.165011

0.537418 0.2041 10,260,868.301514

0.540818 0.204 10,171,948.945027

0.544220 0.2041 10,111,564.578619

0.547622 0.2041 10,116,535.714009

0.551022 0.204 10,260,255.857173

0.554423 0.2041 10,090,526.647015

0.557825 0.2041 9,964,193.719046

0.561225 0.204 10,214,194.188466

0.564627 0.2041 10,162,924.120441

0.568028 0.2041 10,122,112.996743

0.571428 0.204 9,961,253.445221

0.574830 0.2041 9,984,755.448844

0.578232 0.2041 10,310,386.796068

0.581632 0.204 10,309,288.837503

0.585033 0.2041 10,256,773.509046

0.588435 0.2041 10,193,564.538846

0.591835 0.204 10,122,163.135621

0.595237 0.2041 10,165,099.070129

0.598638 0.2041 10,217,816.799809

0.602038 0.204 10,143,840.406727

0.605440 0.2041 10,114,210.005056

0.608842 0.2041 10,037,000.935720

0.612242 0.204 10,058,441.412970

0.615643 0.2041 10,350,617.055630

0.619045 0.2041 10,216,225.433569

0.622445 0.204 10,103,718.738477

0.625847 0.2041 10,274,770.478065

0.629248 0.2041 10,107,168.925032

0.632648 0.204 9,954,126.536177

0.636050 0.2041 10,075,660.119478

0.639452 0.2041 10,163,109.631911

0.642852 0.204 10,154,501.210994

0.646253 0.2041 10,066,199.535087

0.649655 0.2041 10,029,019.511233

0.653055 0.204 10,196,664.606938

0.656457 0.2041 10,194,786.255258

0.659858 0.2041 9,841,143.662869

0.663258 0.204 9,893,706.737122

0.666660 0.2041 10,206,419.373923

0.670062 0.2041 10,207,070.513625

0.673462 0.204 10,102,472.433602

0.676863 0.2041 10,001,922.957398

0.680265 0.2041 10,182,031.756351

0.683665 0.204 10,304,466.488583

0.687067 0.2041 10,221,280.649527

0.690468 0.2041 9,987,515.919654

0.693868 0.204 9,928,721.983343

0.697270 0.2041 10,378,281.592734

0.700672 0.2041 10,445,995.205925

0.704072 0.204 10,233,501.374972

0.707473 0.2041 10,319,199.758201

0.710875 0.2041 10,505,822.811732

0.714275 0.204 10,558,073.256995

0.717677 0.2041 10,442,972.983040

0.721078 0.2041 10,448,492.654202

0.724478 0.204 10,395,584.431155

0.727880 0.2041 10,214,225.132264

0.731282 0.2041 10,387,646.984680

0.734682 0.204 10,271,166.636508

0.738083 0.2041 10,098,560.322884

0.741485 0.2041 10,351,872.107132

0.744885 0.204 10,182,731.084802

0.748287 0.2041 10,214,229.057439

0.751688 0.2041 10,422,801.358641

0.755088 0.204 10,316,305.766952

0.758490 0.2041 10,286,488.254116

0.761892 0.2041 10,288,626.168208

0.765292 0.204 10,218,625.607503

0.768693 0.2041 10,362,048.967176

0.772095 0.2041 10,461,895.238434

0.775495 0.204 10,106,172.009462

0.778897 0.2041 10,280,952.050962

0.782298 0.2041 10,480,329.438437

0.785698 0.204 10,311,411.200853

0.789100 0.2041 10,301,350.430724

0.792502 0.2041 10,358,711.947179

0.795902 0.204 10,341,184.470888

0.799303 0.2041 10,184,823.414610

0.802705 0.2041 10,046,700.706077

0.806107 0.2041 10,047,308.232557

0.809507 0.204 10,205,618.386716

0.812908 0.2041 10,178,060.450257

0.816310 0.2041 10,241,719.298372

0.819710 0.204 10,120,842.059046

0.823112 0.2041 9,857,848.320773

0.826513 0.2041 10,097,841.299788

0.829913 0.204 10,254,053.385635

0.833315 0.2041 10,086,637.223939

0.836717 0.2041 10,328,155.912039

0.840117 0.204 10,516,566.187353

0.843518 0.2041 10,036,972.364677

0.846920 0.2041 10,007,673.307277

0.850320 0.204 10,325,418.821783

0.853722 0.2041 10,000,120.303869

0.857123 0.2041 10,034,195.997709

0.860523 0.204 10,174,821.204804

0.863925 0.2041 9,817,017.135052

0.867327 0.2041 9,840,079.105252

0.870727 0.204 10,084,496.973594

0.874128 0.2041 10,246,188.955945

0.877530 0.2041 10,219,542.017360

0.880930 0.204 10,180,972.521523

0.884332 0.2041 9,982,286.917546

0.887733 0.2041 10,067,484.924374

0.891133 0.204 10,343,781.082935

0.894535 0.2041 10,170,933.209055

0.897937 0.2041 10,151,465.106837

0.901337 0.204 10,099,190.975800

0.904738 0.2041 9,840,250.962227

0.908140 0.2041 10,022,110.926072

0.911540 0.204 10,472,407.749586

0.914942 0.2041 10,287,994.769607

0.918343 0.2041 9,960,061.963199

0.921743 0.204 10,174,541.526370

0.925145 0.2041 10,048,818.337691

0.928547 0.2041 9,756,342.745761

0.931947 0.204 9,961,437.645543

0.935348 0.2041 10,026,554.033598

0.938750 0.2041 10,102,945.696072

0.942150 0.204 10,140,289.789361

0.945552 0.2041 10,101,903.004285

0.948953 0.2041 10,267,240.780432

0.952353 0.204 10,191,679.550105

0.955755 0.2041 10,014,678.477042

0.959157 0.2041 10,150,783.157144

0.962557 0.204 10,219,692.135690

0.965958 0.2041 9,938,260.882943

0.969360 0.2041 10,048,366.180865

0.972760 0.204 10,362,773.451388

0.976162 0.2041 10,276,804.479085

0.979563 0.2041 10,293,739.597316

0.982963 0.204 10,226,103.216243

0.986365 0.2041 9,923,852.031333

0.989767 0.2041 9,856,692.321433

0.993167 0.204 9,978,100.931430

0.996568 0.2041 10,378,570.660083

0.999970 0.2041 10,554,787.762209

1.003370 0.204 10,270,806.704193

1.006772 0.2041 10,396,426.603398

1.010173 0.2041 10,402,918.769642

1.013573 0.204 10,422,746.722922

1.016975 0.2041 10,642,747.810933

1.020377 0.2041 10,575,964.717939

1.023777 0.204 10,877,507.493073

1.027178 0.2041 11,139,769.928571

1.030580 0.2041 11,320,955.442137

1.033980 0.204 11,677,033.706224

1.037382 0.2041 12,039,240.724786

1.040783 0.2041 12,306,337.228453

1.044183 0.204 12,443,525.433794

1.047585 0.2041 12,846,886.480657

1.050987 0.2041 13,662,992.142814

1.054387 0.204 14,152,720.088264

1.057788 0.2041 14,076,956.391063

1.061190 0.2041 14,306,597.332179

1.064590 0.204 14,572,629.745413

1.067992 0.2041 14,663,884.847298

1.071393 0.2041 14,688,073.064770

1.074793 0.204 14,608,680.598796

1.078195 0.2041 14,488,784.788220

1.081597 0.2041 14,196,687.648242

1.084997 0.204 14,099,975.111482

1.088398 0.2041 13,944,821.835939

1.091800 0.2041 13,636,206.637335

1.095200 0.204 13,320,972.524774

1.098602 0.2041 12,745,195.479585

1.102003 0.2041 12,361,284.646569

1.105403 0.204 12,150,903.418909

1.108805 0.2041 11,847,587.538920

1.112207 0.2041 11,864,906.056391

1.115607 0.204 11,970,899.537441

1.119008 0.2041 11,829,998.567995

1.122410 0.2041 11,532,762.969495

1.125810 0.204 11,336,360.504087

1.129212 0.2041 11,322,624.454380

1.132613 0.2041 11,180,179.397262

1.136013 0.204 11,103,696.998616

1.139415 0.2041 10,768,237.209915

1.142817 0.2041 10,583,106.558337

1.146218 0.2041 11,047,903.625238

1.149618 0.204 11,108,131.335102

1.153020 0.2041 10,852,372.399303

1.156422 0.2041 11,057,156.092342

1.159822 0.204 11,286,236.727893

1.163223 0.2041 11,098,455.060867

1.166625 0.2041 10,728,731.785677

1.170025 0.204 10,912,701.751245

1.173427 0.2041 11,087,264.724110

1.176828 0.2041 11,010,337.931942

1.180228 0.204 11,248,194.110423

1.183630 0.2041 11,165,263.254315

1.187032 0.2041 11,114,984.900302

1.190432 0.204 11,272,755.177618

1.193833 0.2041 11,453,387.738586

1.197235 0.2041 11,504,881.425075

1.200635 0.204 11,436,727.540837

1.204037 0.2041 11,516,632.330561

1.207438 0.2041 11,575,124.011286

1.210838 0.204 11,596,235.929423

1.214240 0.2041 11,731,498.317905

1.217642 0.2041 11,721,479.631264

1.221042 0.204 11,627,506.774777

1.224443 0.2041 11,582,719.324421

1.227845 0.2041 11,332,583.411702

1.231245 0.204 11,291,085.016873

1.234647 0.2041 11,282,677.500764

1.238048 0.2041 11,201,705.735256

1.241448 0.204 11,231,772.066931

1.244850 0.2041 11,026,153.981011

1.248252 0.2041 11,013,619.863040

1.251652 0.204 11,155,577.059055

1.255053 0.2041 10,771,250.179254

1.258455 0.2041 10,663,947.831056

1.261855 0.204 10,916,999.285239

1.265257 0.2041 10,744,723.177255

1.268658 0.2041 10,555,169.238791

1.272058 0.204 10,520,423.635607

1.275460 0.2041 10,417,388.667378

1.278862 0.2041 10,182,500.041749

1.282262 0.204 10,221,849.129223

1.285663 0.2041 10,669,887.553208

1.289065 0.2041 10,637,498.829019

1.292465 0.204 10,442,853.163243

1.295867 0.2041 10,593,062.757500

1.299268 0.2041 10,352,821.215695

1.302668 0.204 10,171,084.458466

1.306070 0.2041 10,420,508.775079

1.309472 0.2041 10,372,952.437937

1.312872 0.204 10,195,194.855264

1.316273 0.2041 10,165,865.818484

1.319675 0.2041 10,126,779.249669

1.323075 0.204 10,130,092.901595

1.326477 0.2041 10,213,107.856741

1.329878 0.2041 10,258,695.897130

1.333278 0.204 9,897,512.816645

1.336680 0.2041 9,788,091.417208

1.340082 0.2041 10,166,739.773241

1.343482 0.204 10,071,189.608852

1.346883 0.2041 9,949,171.038143

1.350285 0.2041 10,185,103.533427

1.353685 0.204 10,249,806.711938

1.357087 0.2041 10,226,843.263431

1.360488 0.2041 10,303,590.899442

1.363888 0.204 10,303,007.411010

1.367290 0.2041 10,205,681.950583

1.370692 0.2041 10,248,419.943482

1.374092 0.204 10,216,003.337037

1.377493 0.2041 10,102,152.420999

1.380895 0.2041 10,030,733.314766

1.384295 0.204 9,872,700.773632

1.387697 0.2041 9,925,722.266865

1.391098 0.2041 10,090,768.061548

1.394498 0.204 10,096,655.131375

1.397900 0.2041 10,065,382.484342

1.401302 0.2041 10,170,093.444377

1.404702 0.204 10,041,498.036761

1.408103 0.2041 9,881,024.972875

1.411505 0.2041 9,869,409.444855

1.414905 0.204 9,716,156.979955

1.418307 0.2041 9,590,595.689167

1.421708 0.2041 9,610,734.456367

1.425108 0.204 9,866,081.222149

1.428510 0.2041 9,713,378.931482

1.431912 0.2041 9,598,319.058000

1.435312 0.204 9,556,979.779061

1.438713 0.2041 9,482,042.858190

1.442115 0.2041 9,789,060.559055

1.445515 0.204 9,653,206.613077

1.448917 0.2041 9,640,556.875206

1.452318 0.2041 9,849,812.577706

1.455718 0.204 9,810,442.149859

1.459120 0.2041 9,837,045.401315

1.462522 0.2041 9,590,081.730738

1.465922 0.204 9,468,513.705757

1.469323 0.2041 9,742,733.390970

1.472725 0.2041 9,758,343.985058

1.476125 0.204 9,640,044.575665

1.479527 0.2041 9,695,989.840852

1.482928 0.2041 9,680,623.197938

1.486328 0.204 9,593,103.992908

1.489730 0.2041 9,479,088.123977

1.493132 0.2041 9,575,115.352758

1.496532 0.204 9,763,987.147649

1.499933 0.2041 9,447,158.075376

1.503335 0.2041 9,499,069.852329

1.506735 0.204 9,641,905.339332

1.510137 0.2041 9,553,421.531839

1.513538 0.2041 9,810,871.113321

1.516938 0.204 9,646,392.673091

1.520340 0.2041 9,458,451.047067

1.523742 0.2041 9,604,480.729090

1.527143 0.2041 9,597,326.822209

1.530543 0.204 9,797,335.623217

1.533945 0.2041 9,783,661.632523

1.537347 0.2041 9,341,627.421558

1.540747 0.204 9,417,030.249356

1.544148 0.2041 9,410,383.676148

1.547550 0.2041 9,247,428.552371

1.550950 0.204 9,457,542.138735

1.554352 0.2041 9,600,876.427869

1.557753 0.2041 9,554,031.213422

1.561153 0.204 9,412,515.829788

1.564555 0.2041 9,582,172.161164

1.567957 0.2041 9,582,586.834414

1.571357 0.204 9,562,892.533892

1.574758 0.2041 9,643,502.288128

1.578160 0.2041 9,438,894.072735

1.581560 0.204 9,542,298.292674

1.584962 0.2041 9,553,969.125719

1.588363 0.2041 9,685,851.442888

1.591763 0.204 9,857,171.038804

1.595165 0.2041 9,633,986.330675

1.598567 0.2041 9,484,951.870750

1.601967 0.204 9,679,424.925928

1.605368 0.2041 9,817,652.020068

1.608770 0.2041 9,436,749.830790

1.612170 0.204 9,397,751.942603

1.615572 0.2041 9,526,203.307793

1.618973 0.2041 9,534,079.596250

1.622373 0.204 9,656,801.974139

1.625775 0.2041 9,471,319.131068

1.629177 0.2041 9,581,559.948893

1.632577 0.204 9,690,842.742996

1.635978 0.2041 9,446,022.018573

1.639380 0.2041 9,539,738.615330

1.642780 0.204 9,558,009.710817

1.646182 0.2041 9,581,199.687366

1.649583 0.2041 9,684,980.941693

1.652983 0.204 9,587,446.437751

1.656385 0.2041 9,584,116.135291

1.659787 0.2041 9,655,945.296452

1.663187 0.204 9,634,914.611111

1.666588 0.2041 9,807,159.951417

1.669990 0.2041 9,969,691.993434

1.673390 0.204 9,699,148.612656

1.676792 0.2041 9,415,087.796294

1.680193 0.2041 9,586,954.904025

1.683593 0.204 9,677,044.429455

1.686995 0.2041 9,693,171.435331

1.690397 0.2041 9,713,812.399614

1.693797 0.204 9,520,255.544461

1.697198 0.2041 9,687,755.409896

1.700600 0.2041 9,677,929.691460

1.704000 0.204 9,522,528.532736

1.707402 0.2041 9,652,328.457073

1.710803 0.2041 9,838,180.587043

1.714203 0.204 9,760,003.945024

1.717605 0.2041 9,613,648.177975

1.721007 0.2041 9,817,865.156706

1.724407 0.204 9,898,215.527448

1.727808 0.2041 9,636,847.003197

1.731210 0.2041 9,474,820.234155

1.734610 0.204 9,740,298.525968

1.738012 0.2041 9,724,592.147542

1.741413 0.2041 9,649,425.453875

1.744813 0.204 10,013,099.036957

1.748215 0.2041 9,895,607.807125

1.751617 0.2041 9,589,373.192982

1.755017 0.204 9,591,121.475890

1.758418 0.2041 9,731,900.665269

1.761820 0.2041 9,903,601.925513

1.765220 0.204 9,957,949.858393

1.768622 0.2041 10,064,808.376167

1.772023 0.2041 9,995,410.937643

1.775423 0.204 9,746,865.323847

1.778825 0.2041 9,730,920.126799

1.782227 0.2041 9,897,415.408347

1.785627 0.204 9,989,286.639059

1.789028 0.2041 9,986,027.685257

1.792430 0.2041 9,868,586.869296

1.795830 0.204 9,792,283.380013

1.799232 0.2041 9,914,947.684556

1.802633 0.2041 10,021,214.161930

1.806033 0.204 10,138,196.977965

1.809435 0.2041 9,956,297.756254

1.812837 0.2041 9,449,632.389442

1.816237 0.204 9,474,368.478096

1.819638 0.2041 9,790,236.129125

1.823040 0.2041 9,778,482.233901

1.826440 0.204 9,837,435.474616

1.829842 0.2041 9,926,766.903839

1.833243 0.2041 9,899,245.104468

1.836643 0.204 9,967,909.227737

1.840045 0.2041 9,873,698.036803

1.843447 0.2041 9,654,283.205513

1.846847 0.204 9,601,630.447595

1.850248 0.2041 9,675,291.468241

1.853650 0.2041 9,814,979.868911

1.857050 0.204 9,677,540.388860

1.860452 0.2041 9,487,934.637509

1.863853 0.2041 9,720,101.713144

1.867253 0.204 9,715,640.342948

1.870655 0.2041 9,580,042.915319

1.874057 0.2041 9,905,219.181730

1.877457 0.204 10,047,552.170459

1.880858 0.2041 9,689,499.342235

1.884260 0.2041 9,844,565.326940

1.887660 0.204 10,312,997.980015

1.891062 0.2041 9,966,988.243839

1.894463 0.2041 9,609,675.305420

1.897863 0.204 9,721,078.763674

1.901265 0.2041 9,854,296.527892

1.904667 0.2041 10,014,989.119843

1.908067 0.204 10,027,249.285350

1.911468 0.2041 9,891,787.455157

1.914870 0.2041 9,879,842.764730

1.918272 0.2041 9,900,998.826349

1.921672 0.204 9,861,113.455956

1.925073 0.2041 9,915,503.460730

1.928475 0.2041 9,711,741.215365

1.931875 0.204 9,668,867.597792

1.935277 0.2041 10,010,415.167279

1.938678 0.2041 9,947,044.272641

1.942078 0.204 9,760,633.707373

1.945480 0.2041 9,753,598.840125

1.948882 0.2041 9,696,693.784510

1.952282 0.204 9,939,325.522993

1.955683 0.2041 9,869,579.163886

1.959085 0.2041 9,622,398.066608

1.962485 0.204 9,736,587.857644

1.965887 0.2041 9,687,848.286141

1.969288 0.2041 9,834,954.750780

1.972688 0.204 9,819,420.595488

1.976090 0.2041 9,770,125.787032

1.979492 0.2041 9,939,414.038325

1.982892 0.204 9,848,127.007460

1.986293 0.2041 9,838,066.285324

1.989695 0.2041 9,874,140.574283

1.993095 0.204 9,797,134.769325

1.996497 0.2041 9,922,840.223962

1.999898 0.2041 9,997,333.630998

2.003298 0.204 10,117,804.796979

2.006700 0.2041 10,125,561.160108

2.010102 0.2041 9,956,536.664812

2.013502 0.204 9,893,710.499525

2.016903 0.2041 9,712,805.079800

2.020305 0.2041 9,725,791.201803

2.023705 0.204 9,788,436.712416

2.027107 0.2041 9,939,213.223485

2.030508 0.2041 9,864,145.486468

2.033908 0.204 9,818,260.188249

2.037310 0.2041 10,132,460.330460

2.040712 0.2041 10,285,106.644274

2.044112 0.204 10,059,416.932295

2.047513 0.2041 9,815,163.823028

2.050915 0.2041 10,196,793.143881

2.054315 0.204 10,121,529.798511

2.057717 0.2041 9,704,487.079629

2.061118 0.2041 9,869,423.459372

2.064518 0.204 9,903,330.055341

2.067920 0.2041 9,698,324.032781

2.071322 0.2041 9,804,142.757759

2.074722 0.204 10,179,648.807828

2.078123 0.2041 10,080,981.877484

2.081525 0.2041 9,786,800.234120

2.084925 0.204 9,878,875.085387

2.088327 0.2041 9,977,578.317098

2.091728 0.2041 10,078,424.688099

2.095128 0.204 10,033,628.861707

2.098530 0.2041 9,618,689.613889

2.101932 0.2041 9,448,892.409975

2.105332 0.204 9,620,759.789967

2.108733 0.2041 9,643,120.603085

2.112135 0.2041 9,659,573.169744

2.115535 0.204 9,405,679.440787

2.118937 0.2041 9,333,548.115476

2.122338 0.2041 9,531,697.590419

2.125738 0.204 9,412,625.333291

2.129140 0.2041 9,647,701.245936

2.132542 0.2041 9,634,252.627052

2.135942 0.204 9,560,129.693817

2.139343 0.2041 9,760,653.185132

2.142745 0.2041 9,647,296.813187

2.146145 0.204 9,502,292.016675

2.149547 0.2041 9,392,757.534635

2.152948 0.2041 9,346,670.516374

2.156348 0.204 9,378,296.530620

2.159750 0.2041 9,659,611.597492

2.163152 0.2041 9,667,751.005981

2.166552 0.204 9,454,904.055200

2.169953 0.2041 9,395,199.957128

2.173355 0.2041 9,424,840.384331

2.176755 0.204 9,358,920.686004

2.180157 0.2041 9,268,812.705576

2.183558 0.2041 9,591,980.776754

2.186958 0.204 9,547,523.486514

2.190360 0.2041 9,300,153.206719

2.193762 0.2041 9,335,273.002432

2.197162 0.204 9,448,769.339366

2.200563 0.2041 9,687,765.403093

2.203965 0.2041 9,429,421.899122

2.207365 0.204 9,275,416.364789

2.210767 0.2041 9,624,765.907352

2.214168 0.2041 9,478,628.682977

2.217568 0.204 9,275,387.947604

2.220970 0.2041 9,477,232.458518

2.224372 0.2041 9,641,438.011115

2.227772 0.204 9,571,381.716209

2.231173 0.2041 9,499,261.050374

2.234575 0.2041 9,431,733.910015

2.237975 0.204 9,399,118.449041

2.241377 0.2041 9,453,910.009177

2.244778 0.2041 9,360,999.663240

2.248178 0.204 9,442,511.600566

2.251580 0.2041 9,495,597.708792

2.254982 0.2041 9,556,859.889874

2.258383 0.2041 9,585,350.735976

2.261783 0.204 9,460,388.079245

2.265185 0.2041 9,546,589.090221

2.268587 0.2041 9,550,866.527651

2.271987 0.204 9,494,018.024817

2.275388 0.2041 9,537,082.282103

2.278790 0.2041 9,360,003.442045

2.282190 0.204 9,281,999.629253

2.285592 0.2041 9,561,417.465785

2.288993 0.2041 9,542,109.973316

2.292393 0.204 9,393,273.858117

2.295795 0.2041 9,414,762.448187

2.299197 0.2041 9,413,738.585895

2.302597 0.204 9,419,818.547411

2.305998 0.2041 9,514,740.068512

2.309400 0.2041 9,650,252.069432

2.312800 0.204 9,630,677.545023

2.316202 0.2041 9,428,391.684576

2.319603 0.2041 9,513,114.138165

2.323003 0.204 9,714,262.350881

2.326405 0.2041 9,696,638.798086

2.329807 0.2041 9,670,229.903938

2.333207 0.204 9,501,696.092522

2.336608 0.2041 9,366,949.212102

2.340010 0.2041 9,417,286.642267

2.343410 0.204 9,463,766.573307

2.346812 0.2041 9,496,298.330135

2.350213 0.2041 9,654,066.476030

2.353613 0.204 9,497,749.046487

2.357015 0.2041 9,257,485.284144

2.360417 0.2041 9,486,833.943379

2.363817 0.204 9,506,835.249433

2.367218 0.2041 9,377,012.979319

2.370620 0.2041 9,675,807.706941

2.374020 0.204 9,681,611.751267

2.377422 0.2041 9,455,133.932608

2.380823 0.2041 9,404,284.423487

2.384223 0.204 9,440,773.195250

2.387625 0.2041 9,766,277.777822

2.391027 0.2041 9,672,477.294414

2.394427 0.204 9,499,968.123784

2.397828 0.2041 9,748,585.799361

2.401230 0.2041 9,566,379.265405

2.404630 0.204 9,274,775.871938

2.408032 0.2041 9,171,393.481163

2.411433 0.2041 9,147,216.732448

2.414833 0.204 9,415,827.788120

2.418235 0.2041 9,436,405.182208

2.421637 0.2041 9,389,183.284712

2.425037 0.204 9,514,598.724641

2.428438 0.2041 9,442,098.763734

2.431840 0.2041 9,483,163.324608

2.435240 0.204 9,376,386.881778

2.438642 0.2041 9,354,383.945806

2.442043 0.2041 9,599,760.813398

2.445443 0.204 9,351,379.232393

2.448845 0.2041 9,204,476.588847

2.452247 0.2041 9,291,004.802423

2.455647 0.204 9,264,584.401158

2.459048 0.2041 9,311,410.612640

2.462450 0.2041 9,407,253.408429

2.465850 0.204 9,383,875.237045

2.469252 0.2041 9,231,434.124350

2.472653 0.2041 9,236,622.799553

2.476053 0.204 9,115,890.840730

2.479455 0.2041 9,226,205.478746

2.482857 0.2041 9,321,176.210590

2.486257 0.204 9,282,423.356276

2.489658 0.2041 9,568,837.890483

2.493060 0.2041 9,414,258.629863

2.496460 0.204 9,272,629.865997

2.499862 0.2041 9,511,999.563834

2.503263 0.2041 9,548,171.736563

2.506663 0.204 9,361,549.389012

2.510065 0.2041 9,347,884.240226

2.513467 0.2041 9,596,408.481939

2.516867 0.204 9,753,200.825268

2.520268 0.2041 9,446,943.609233

2.523670 0.2041 9,363,824.844736

2.527070 0.204 9,783,638.813022

2.530472 0.2041 9,796,876.824047

2.533873 0.2041 9,930,570.752291

2.537273 0.204 10,154,786.325995

2.540675 0.2041 9,995,091.107194

2.544077 0.2041 9,926,349.828287

2.547477 0.204 10,014,883.966908

2.550878 0.2041 10,293,762.841244

2.554280 0.2041 10,366,197.486563

2.557680 0.204 10,266,359.699305

2.561082 0.2041 10,110,367.248042

2.564483 0.2041 10,060,181.527133

2.567883 0.204 10,341,656.476285

2.571285 0.2041 10,271,651.325515

2.574687 0.2041 10,074,475.004394

2.578087 0.204 9,959,134.797231

2.581488 0.2041 9,891,711.006425

2.584890 0.2041 9,941,850.527628

2.588290 0.204 9,912,938.506592

2.591692 0.2041 9,752,497.145587

2.595093 0.2041 9,574,422.476499

2.598493 0.204 9,659,809.148533

2.601895 0.2041 9,785,143.855077

2.605297 0.2041 9,525,862.441358

2.608698 0.2041 9,354,396.112690

2.612098 0.204 9,478,441.266803

2.615500 0.2041 9,444,193.754347

2.618902 0.2041 9,529,247.515470

2.622302 0.204 9,537,454.689355

2.625703 0.2041 9,225,951.982741

2.629105 0.2041 9,181,611.894017

2.632505 0.204 9,394,055.540145

2.635907 0.2041 9,497,108.144220

2.639308 0.2041 9,328,170.053113

2.642708 0.204 9,261,773.089294

2.646110 0.2041 9,446,163.689584

2.649512 0.2041 9,472,533.787509

2.652912 0.204 9,679,139.439436

2.656313 0.2041 9,674,173.794363

2.659715 0.2041 9,437,274.173990

2.663115 0.204 9,214,141.115507

2.666517 0.2041 9,017,205.541812

2.669918 0.2041 9,163,061.410466

2.673318 0.204 9,432,006.883913

2.676720 0.2041 9,753,914.569683

2.680122 0.2041 9,581,267.363958

2.683522 0.204 9,342,092.780579

2.686923 0.2041 9,235,672.494780

2.690325 0.2041 9,181,105.513526

2.693725 0.204 9,398,995.569870

2.697127 0.2041 9,492,613.178488

2.700528 0.2041 9,496,652.592799

2.703928 0.204 9,292,904.681971

2.707330 0.2041 9,365,611.749686

2.710732 0.2041 9,567,114.893755

2.714132 0.204 9,435,307.286830

2.717533 0.2041 9,392,878.781588

2.720935 0.2041 9,374,166.416940

2.724335 0.204 9,341,755.166621

2.727737 0.2041 9,407,610.461686

2.731138 0.2041 9,424,675.266394

2.734538 0.204 9,407,834.327454

2.737940 0.2041 9,224,080.342391

2.741342 0.2041 9,106,458.940985

2.744742 0.204 9,285,466.790176

2.748143 0.2041 9,305,822.386528

2.751545 0.2041 9,196,073.510544

2.754945 0.204 9,214,120.143613

2.758347 0.2041 9,509,610.003482

2.761748 0.2041 9,485,089.297245

2.765148 0.204 9,280,728.909227

2.768550 0.2041 9,414,101.660971

2.771952 0.2041 9,279,631.102168

2.775352 0.204 9,080,608.491979

2.778753 0.2041 9,246,618.184573

2.782155 0.2041 9,459,406.558471

2.785555 0.204 9,383,536.935845

2.788957 0.2041 9,232,752.785476

2.792358 0.2041 9,299,372.749682

2.795758 0.204 9,459,146.079907

2.799160 0.2041 9,362,141.507777

2.802562 0.2041 9,421,362.833658

2.805962 0.204 9,313,376.954482

2.809363 0.2041 9,140,432.907557

2.812765 0.2041 9,361,570.604904

2.816165 0.204 9,217,516.340897

2.819567 0.2041 9,271,048.119365

2.822968 0.2041 9,425,013.494547

2.826368 0.204 9,397,734.003624

2.829770 0.2041 9,380,181.012064

2.833172 0.2041 9,291,764.833621

2.836572 0.204 9,537,753.263956

2.839973 0.2041 9,695,684.336130

2.843375 0.2041 9,537,090.215921

2.846775 0.204 9,551,029.472202

2.850177 0.2041 9,439,822.176470

2.853578 0.2041 9,354,933.919866

2.856978 0.204 9,559,760.767883

2.860380 0.2041 9,451,766.790064

2.863782 0.2041 9,364,779.938429

2.867182 0.204 9,485,496.405980

2.870583 0.2041 9,478,424.507685

2.873985 0.2041 9,478,680.058981

2.877385 0.204 9,468,088.403091

2.880787 0.2041 9,396,692.690043

2.884188 0.2041 9,321,898.733768

2.887588 0.204 9,334,714.848743

2.890990 0.2041 9,387,786.208523

2.894392 0.2041 9,416,338.729992

2.897792 0.204 9,259,555.526204

2.901193 0.2041 9,048,506.891877

2.904595 0.2041 9,290,072.270750

2.907995 0.204 9,343,676.945553

2.911397 0.2041 9,151,787.512879

2.914798 0.2041 9,350,917.303601

2.918198 0.204 9,398,026.712820

2.921600 0.2041 9,285,228.674539

2.925002 0.2041 9,241,030.448358

2.928402 0.204 9,218,004.962624

2.931803 0.2041 9,418,821.356907

2.935205 0.2041 9,356,340.186721

2.938605 0.204 9,177,768.750196

2.942007 0.2041 9,250,741.451274

2.945408 0.2041 9,245,878.670035

2.948810 0.2041 9,188,139.682361

2.952210 0.204 9,071,534.090536

2.955612 0.2041 9,050,324.319682

2.959013 0.2041 9,242,679.808451

2.962413 0.204 9,296,421.952575

2.965815 0.2041 9,272,727.228478

2.969217 0.2041 9,176,818.729479

2.972617 0.204 9,202,801.421519

2.976018 0.2041 9,454,647.691382

2.979420 0.2041 9,327,132.151297

2.982820 0.204 9,252,509.000948

2.986222 0.2041 9,343,795.752554

2.989623 0.2041 9,301,953.304542

2.993023 0.204 9,249,520.125595

2.996425 0.2041 9,176,759.185878

2.999827 0.2041 9,054,907.447920

3.003227 0.204 9,180,382.439671

3.006628 0.2041 9,468,065.218593

3.010030 0.2041 9,258,506.309262

3.013430 0.204 9,134,514.330298

3.016832 0.2041 9,277,112.066373

3.020233 0.2041 9,253,484.373083

3.023633 0.204 9,359,579.180970

3.027035 0.2041 9,482,693.211660

3.030437 0.2041 9,413,806.061761

3.033837 0.204 9,397,366.708527

3.037238 0.2041 9,096,832.584957

3.040640 0.2041 8,868,956.448176

3.044040 0.204 9,172,567.664823

3.047442 0.2041 9,370,596.513102

3.050843 0.2041 9,298,627.528342

3.054243 0.204 9,078,113.821630

3.057645 0.2041 9,099,172.732730

3.061047 0.2041 9,288,844.758814

3.064447 0.204 9,302,276.355123

3.067848 0.2041 9,239,460.960017

3.071250 0.2041 9,117,249.837620

3.074650 0.204 9,030,095.514246

3.078052 0.2041 9,067,862.742784

3.081453 0.2041 9,289,059.229728

3.084853 0.204 9,319,361.998829

3.088255 0.2041 9,083,036.416490

3.091657 0.2041 9,213,124.723805

3.095057 0.204 9,317,574.551527

3.098458 0.2041 9,168,681.405999

3.101860 0.2041 9,205,069.578979

3.105260 0.204 9,244,224.008986

3.108662 0.2041 9,322,258.415407

3.112063 0.2041 9,214,101.646960

3.115463 0.204 8,964,726.171034

3.118865 0.2041 9,138,520.834008

3.122267 0.2041 9,205,676.381710

3.125667 0.204 9,145,887.482999

3.129068 0.2041 9,370,609.236194

3.132470 0.2041 9,240,157.349774

3.135870 0.204 8,952,906.256961

3.139272 0.2041 9,186,011.377772

3.142673 0.2041 9,391,411.860216

3.146073 0.204 9,322,320.460472

3.149475 0.2041 9,274,513.075344

3.152877 0.2041 9,154,433.670663

3.156277 0.204 9,238,516.873386

3.159678 0.2041 9,345,954.972125

3.163080 0.2041 9,204,657.820294

3.166480 0.204 9,213,162.804216

3.169882 0.2041 9,259,731.417103

3.173283 0.2041 9,223,674.985950

3.176683 0.204 9,129,810.233208

3.180085 0.2041 9,168,283.960720

3.183487 0.2041 9,306,805.481837

3.186887 0.204 9,110,182.647696

3.190288 0.2041 9,050,467.338649

3.193690 0.2041 9,211,913.868974

3.197090 0.204 9,332,154.131995

3.200492 0.2041 9,490,186.060758

3.203893 0.2041 9,337,012.467543

3.207293 0.204 9,044,025.511698

3.210695 0.2041 9,058,197.751307

3.214097 0.2041 9,260,524.025139

3.217497 0.204 9,409,696.859609

3.220898 0.2041 9,324,422.280458

3.224300 0.2041 9,040,003.060223

3.227700 0.204 9,158,044.290076

3.231102 0.2041 9,398,304.136819

3.234503 0.2041 9,193,363.800394

3.237903 0.204 9,020,310.308109

3.241305 0.2041 9,072,173.067450

3.244707 0.2041 9,382,714.226270

3.248107 0.204 9,443,997.667192

3.251508 0.2041 9,084,058.264553

3.254910 0.2041 8,937,828.028836

3.258310 0.204 8,848,419.093659

3.261712 0.2041 9,006,088.782281

3.265113 0.2041 9,341,265.317059

3.268513 0.204 9,291,250.723180

3.271915 0.2041 9,103,234.653261

3.275317 0.2041 8,865,936.414283

3.278717 0.204 8,969,846.741878

3.282118 0.2041 9,469,266.595926

3.285520 0.2041 9,384,590.403870

3.288920 0.204 9,051,988.286557

3.292322 0.2041 9,207,701.307042

3.295723 0.2041 9,119,730.962740

3.299123 0.204 8,900,947.306240

3.302525 0.2041 9,082,891.564444

3.305927 0.2041 9,285,471.899021

3.309328 0.2041 9,149,283.860972

3.312728 0.204 9,126,398.121081

3.316130 0.2041 9,177,736.597101

3.319532 0.2041 9,019,600.067778

3.322932 0.204 9,337,816.239411

3.326333 0.2041 9,522,430.405114

3.329735 0.2041 9,338,148.321329

3.333135 0.204 9,463,231.390407

3.336537 0.2041 9,669,330.258559

3.339938 0.2041 9,867,952.611045

3.343338 0.204 10,005,137.234090

3.346740 0.2041 10,068,266.600465

3.350142 0.2041 10,372,845.043015

3.353542 0.204 10,610,976.488883

3.356943 0.2041 10,712,707.256366

3.360345 0.2041 11,163,206.847357

3.363745 0.204 11,558,237.937630

3.367147 0.2041 11,554,437.690926

3.370548 0.2041 11,780,878.203781

3.373948 0.204 12,305,574.822134

3.377350 0.2041 12,773,692.951125

3.380752 0.2041 13,090,251.276003

3.384152 0.204 13,122,954.289144

3.387553 0.2041 13,098,477.465498

3.390955 0.2041 12,902,551.791597

3.394355 0.204 12,640,542.884752

3.397757 0.2041 12,503,933.794657

3.401158 0.2041 12,173,522.589168

3.404558 0.204 11,860,639.814430

3.407960 0.2041 11,569,864.120635

3.411362 0.2041 11,064,910.052259

3.414762 0.204 10,710,460.403185

3.418163 0.2041 10,545,237.132734

3.421565 0.2041 10,243,989.448985

3.424965 0.204 9,924,603.974090

3.428367 0.2041 9,556,293.706508

3.431768 0.2041 9,482,602.926594

3.435168 0.204 9,530,485.416954

3.438570 0.2041 9,091,834.934797

3.441972 0.2041 9,042,133.749023

3.445372 0.204 9,387,869.210510

3.448773 0.2041 9,394,757.039678

3.452175 0.2041 9,156,861.017391

3.455575 0.204 9,133,136.720893

3.458977 0.2041 9,184,216.364481

3.462378 0.2041 9,004,144.316800

3.465778 0.204 9,193,851.832388

3.469180 0.2041 9,286,416.141524

3.472582 0.2041 9,224,361.418177

3.475982 0.204 9,433,104.329117

3.479383 0.2041 9,136,364.904575

3.482785 0.2041 8,867,242.388188

3.486185 0.204 9,080,727.432905

3.489587 0.2041 8,948,939.662656

3.492988 0.2041 8,953,596.255760

3.496388 0.204 9,218,628.432706

3.499790 0.2041 9,171,052.326588

3.503192 0.2041 9,209,712.617955

3.506592 0.204 9,155,799.013556

3.509993 0.2041 8,994,250.743824

3.513395 0.2041 8,945,416.321799

3.516795 0.204 8,955,487.720722

3.520197 0.2041 9,072,657.733251

3.523598 0.2041 9,004,245.479963

3.526998 0.204 9,107,436.112911

3.530400 0.2041 9,299,923.080226

3.533802 0.2041 9,171,086.593322

3.537202 0.204 9,095,895.444533

3.540603 0.2041 9,283,705.401880

3.544005 0.2041 9,211,497.430530

3.547405 0.204 9,047,589.209259

3.550807 0.2041 9,152,333.442156

3.554208 0.2041 9,150,568.382211

3.557608 0.204 9,231,885.940449

3.561010 0.2041 9,165,236.893200

3.564412 0.2041 8,989,488.163873

3.567812 0.204 9,075,976.746731

3.571213 0.2041 9,139,654.191354

3.574615 0.2041 8,973,796.686383

3.578015 0.204 9,058,568.460066

3.581417 0.2041 9,409,439.100622

3.584818 0.2041 9,484,832.025835

3.588218 0.204 9,348,295.522225

3.591620 0.2041 9,134,769.538789

3.595022 0.2041 9,222,796.313042

3.598422 0.204 9,243,739.235886

3.601823 0.2041 9,104,051.402973

3.605225 0.2041 9,319,194.495124

3.608625 0.204 9,229,130.787109

3.612027 0.2041 9,117,444.667741

3.615428 0.2041 9,163,169.988359

3.618828 0.204 8,943,544.237291

3.622230 0.2041 8,975,793.469211

3.625632 0.2041 9,120,849.384405

3.629032 0.204 9,204,743.135430

3.632433 0.2041 9,263,609.810568

3.635835 0.2041 9,218,305.987678

3.639235 0.204 9,136,652.851120

3.642637 0.2041 9,346,535.194771

3.646038 0.2041 9,472,921.974750

3.649438 0.204 9,111,129.530466

3.652840 0.2041 9,294,598.231016

3.656242 0.2041 9,498,500.633981

3.659642 0.204 9,188,325.582767

3.663043 0.2041 9,093,560.725953

3.666445 0.2041 9,066,135.029086

3.669845 0.204 8,964,252.847844

3.673247 0.2041 8,911,622.032807

3.676648 0.2041 8,998,728.143369

3.680050 0.2041 9,119,254.857248

3.683450 0.204 9,085,528.446740

3.686852 0.2041 9,225,744.455808

3.690253 0.2041 9,378,340.117565

3.693653 0.204 9,012,367.589778

3.697055 0.2041 8,769,641.427039

3.700457 0.2041 9,119,437.672013

3.703857 0.204 9,070,802.457074

3.707258 0.2041 8,989,780.889719

3.710660 0.2041 9,185,827.520226

3.714060 0.204 9,123,600.226882

3.717462 0.2041 9,192,525.024183

3.720863 0.2041 9,066,217.345857

3.724263 0.204 8,974,404.463138

3.727665 0.2041 9,035,705.274973

3.731067 0.2041 8,800,741.110016

3.734467 0.204 8,732,967.790288

3.737868 0.2041 8,946,394.554881

3.741270 0.2041 9,127,398.430841

3.744670 0.204 9,339,873.334321

3.748072 0.2041 9,210,205.743050

3.751473 0.2041 9,113,039.935074

3.754873 0.204 9,113,023.927171

3.758275 0.2041 8,866,021.114148

3.761677 0.2041 9,006,031.490734

3.765077 0.204 9,152,227.432291

3.768478 0.2041 9,041,998.539317

3.771880 0.2041 9,190,737.573716

3.775280 0.204 9,225,505.693330

3.778682 0.2041 8,963,535.024468

3.782083 0.2041 8,878,008.244848

3.785483 0.204 9,012,430.948696

3.788885 0.2041 9,135,034.524675

3.792287 0.2041 9,290,347.710292

3.795687 0.204 9,315,701.736811

3.799088 0.2041 9,129,349.669985

3.802490 0.2041 9,229,335.692658

3.805890 0.204 9,209,231.964837

3.809292 0.2041 9,021,417.952670

3.812693 0.2041 9,032,143.148419

3.816093 0.204 9,129,891.168341

3.819495 0.2041 8,997,241.965140

3.822897 0.2041 8,952,390.378938

3.826297 0.204 9,080,175.076561

3.829698 0.2041 9,054,904.682486

3.833100 0.2041 9,253,416.502964

3.836500 0.204 9,323,324.349692

3.839902 0.2041 9,261,953.277427

3.843303 0.2041 9,278,574.723567

3.846703 0.204 9,135,157.755075

3.850105 0.2041 9,023,858.455344

3.853507 0.2041 9,201,624.140830

3.856907 0.204 9,313,271.604621

3.860308 0.2041 9,096,236.277978

3.863710 0.2041 9,103,528.838864

3.867110 0.204 9,036,038.781149

3.870512 0.2041 8,878,715.115339

3.873913 0.2041 9,282,271.287908

3.877313 0.204 9,364,948.147990

3.880715 0.2041 9,100,024.655756

3.884117 0.2041 9,369,578.468201

3.887517 0.204 9,353,817.655504

3.890918 0.2041 8,983,790.876187

3.894320 0.2041 8,975,698.005309

3.897720 0.204 8,781,359.249328

3.901122 0.2041 8,780,457.156535

3.904523 0.2041 9,173,160.723543

3.907923 0.204 9,050,079.257753

3.911325 0.2041 8,961,545.932247

3.914727 0.2041 8,932,639.877651

3.918127 0.204 8,820,277.837733

3.921528 0.2041 9,204,498.888163

3.924930 0.2041 9,240,522.666929

3.928330 0.204 9,036,870.038983

3.931732 0.2041 9,137,385.825749

3.935133 0.2041 9,212,170.721013

3.938533 0.204 9,211,996.351237

3.941935 0.2041 9,011,989.066766

3.945337 0.2041 8,944,090.114765

3.948737 0.204 9,021,715.239559

3.952138 0.2041 8,994,767.847706

3.955540 0.2041 9,079,005.469577

3.958940 0.204 9,157,825.083475

3.962342 0.2041 9,170,273.739178

3.965743 0.2041 9,078,180.335924

3.969143 0.204 8,833,368.912476

3.972545 0.2041 8,767,203.556348

3.975947 0.2041 8,909,865.702232

3.979347 0.204 9,085,372.510002

3.982748 0.2041 9,040,753.343522

3.986150 0.2041 8,900,703.476146

3.989550 0.204 8,822,556.931246

3.992952 0.2041 8,839,364.055044

3.996353 0.2041 9,175,449.660844

3.999753 0.204 9,162,717.465608

4.003155 0.2041 9,064,572.141670

4.006557 0.2041 8,944,481.705553

4.009957 0.204 8,887,273.568362

4.013358 0.2041 9,278,293.356910

4.016760 0.2041 9,059,841.057047

4.020160 0.204 8,903,213.574485

4.023562 0.2041 8,991,409.503435

4.026963 0.2041 8,826,283.661753

4.030365 0.2041 8,933,955.235252

4.033765 0.204 8,865,863.030733

4.037167 0.2041 8,857,886.623006

4.040568 0.2041 9,204,331.340131

4.043968 0.204 9,203,502.153566

4.047370 0.2041 9,015,194.997625

4.050772 0.2041 9,014,900.142447

4.054172 0.204 9,092,679.374329

4.057573 0.2041 9,117,385.032893

4.060975 0.2041 8,964,297.461779

4.064375 0.204 8,856,654.612237

4.067777 0.2041 9,055,254.436935

4.071178 0.2041 9,205,315.809248

4.074578 0.204 9,173,395.083994

4.077980 0.2041 9,140,993.368532

4.081382 0.2041 9,153,181.781425

4.084782 0.204 9,173,941.818043

4.088183 0.2041 9,026,732.750092

4.091585 0.2041 8,904,705.664839

4.094985 0.204 8,968,763.946437

4.098387 0.2041 8,905,985.901855

4.101788 0.2041 8,863,867.558663

4.105188 0.204 9,058,017.558035

4.108590 0.2041 9,160,956.158402

4.111992 0.2041 9,057,942.312590

4.115392 0.204 9,034,144.592421

4.118793 0.2041 8,975,331.097225

4.122195 0.2041 8,876,784.384618

4.125595 0.204 9,015,906.563397

4.128997 0.2041 9,054,995.308402

4.132398 0.2041 8,968,943.528733

4.135798 0.204 8,979,316.028838

4.139200 0.2041 8,959,721.230619

4.142602 0.2041 8,967,938.550589

4.146002 0.204 8,762,167.954949

4.149403 0.2041 8,731,364.891151

4.152805 0.2041 8,970,429.436934

4.156205 0.204 8,842,433.783377

4.159607 0.2041 8,946,445.492142

4.163008 0.2041 9,047,694.206936

4.166408 0.204 9,024,316.761234

4.169810 0.2041 9,042,757.741441

4.173212 0.2041 8,976,708.385721

4.176612 0.204 8,868,877.169054

4.180013 0.2041 8,746,884.075540

4.183415 0.2041 9,002,668.197832

4.186815 0.204 9,200,934.135433

4.190217 0.2041 8,963,564.666069

4.193618 0.2041 8,622,418.824004

4.197018 0.204 8,781,427.051470

4.200420 0.2041 9,293,428.338010

4.203822 0.2041 9,407,667.227278

4.207222 0.204 9,182,856.681957

4.210623 0.2041 9,085,558.010452

4.214025 0.2041 9,070,733.300232

4.217425 0.204 9,094,305.176150

4.220827 0.2041 9,099,133.913677

4.224228 0.2041 9,081,017.711994

4.227628 0.204 9,079,249.604405

4.231030 0.2041 8,932,857.480473

4.234432 0.2041 8,978,619.944835

4.237832 0.204 9,230,495.783287

4.241233 0.2041 9,263,514.737685

4.244635 0.2041 9,104,100.976740

4.248035 0.204 9,053,880.801850

4.251437 0.2041 8,978,528.418974

4.254838 0.2041 8,714,227.342679

4.258238 0.204 8,746,193.016335

4.261640 0.2041 9,080,125.218459

4.265042 0.2041 8,965,069.384393

4.268442 0.204 8,846,370.062917

4.271843 0.2041 8,913,097.697294

4.275245 0.2041 8,839,877.617763

4.278645 0.204 9,096,556.882589

4.282047 0.2041 9,231,536.737731

4.285448 0.2041 9,206,104.507140

4.288848 0.204 9,241,382.152698

4.292250 0.2041 9,157,855.408136

4.295652 0.2041 9,019,878.987443

4.299052 0.204 8,870,149.707985

4.302453 0.2041 8,768,629.298976

4.305855 0.2041 8,968,421.998616

4.309255 0.204 9,132,684.179753

4.312657 0.2041 9,057,576.224383

4.316058 0.2041 9,125,859.243656

4.319458 0.204 8,983,904.426462

4.322860 0.2041 8,890,067.318108

4.326262 0.2041 9,121,859.899391

4.329662 0.204 9,110,758.764656

4.333063 0.2041 9,052,965.025438

4.336465 0.2041 9,134,729.892164

4.339865 0.204 9,009,130.558738

4.343267 0.2041 8,936,196.041189

4.346668 0.2041 9,000,558.436158

4.350068 0.204 9,061,844.640225

4.353470 0.2041 9,010,585.728883

4.356872 0.2041 8,973,502.918686

4.360272 0.204 9,104,572.859506

4.363673 0.2041 9,083,340.952763

4.367075 0.2041 9,159,580.581674

4.370475 0.204 9,265,535.279019

4.373877 0.2041 9,123,683.847161

4.377278 0.2041 8,860,236.589962

4.380678 0.204 8,857,998.919128

4.384080 0.2041 9,070,435.316559

4.387482 0.2041 8,804,406.250660

4.390883 0.2041 8,705,920.564123

4.394283 0.204 8,897,419.820767

4.397685 0.2041 8,976,582.346294

4.401087 0.2041 8,937,896.556421

4.404487 0.204 8,883,594.366719

4.407888 0.2041 9,103,471.735546

4.411290 0.2041 9,145,654.501669

4.414690 0.204 9,065,042.842082

4.418092 0.2041 9,166,386.526928

4.421493 0.2041 9,025,240.951992

4.424893 0.204 8,818,003.278619

4.428295 0.2041 9,169,159.626031

4.431697 0.2041 9,167,601.674414

4.435097 0.204 8,765,568.060556

4.438498 0.2041 8,937,667.636680

4.441900 0.2041 9,259,249.785955

4.445300 0.204 9,111,187.522607

4.448702 0.2041 9,032,244.599403

4.452103 0.2041 9,007,956.450913

4.455503 0.204 8,756,303.041768

4.458905 0.2041 8,867,384.588625

4.462307 0.2041 8,915,448.918077

4.465707 0.204 8,986,714.756698

4.469108 0.2041 9,277,496.983905

4.472510 0.2041 9,196,644.347943

4.475910 0.204 9,256,483.742889

4.479312 0.2041 9,194,290.820747

4.482713 0.2041 8,827,912.685958

4.486113 0.204 8,995,502.373935

4.489515 0.2041 9,142,273.521201

4.492917 0.2041 9,100,070.743649

4.496317 0.204 9,166,087.908156

4.499718 0.2041 9,200,959.044020

4.503120 0.2041 9,258,738.829652

4.506520 0.204 9,374,394.005284

4.509922 0.2041 9,165,518.722538

4.513323 0.2041 9,011,277.754943

4.516723 0.204 9,160,468.341841

4.520125 0.2041 9,128,026.951610

4.523527 0.2041 9,248,828.910653

4.526927 0.204 9,221,408.040335

4.530328 0.2041 9,286,902.348202

4.533730 0.2041 9,573,469.478238

4.537130 0.204 9,493,152.788817

4.540532 0.2041 9,410,627.502257

4.543933 0.2041 9,521,341.486163

4.547333 0.204 9,632,675.287258

4.550735 0.2041 9,845,977.295358

4.554137 0.2041 10,071,136.117826

4.557537 0.204 10,104,714.071374

4.560938 0.2041 10,137,783.337009

4.564340 0.2041 10,030,971.294316

4.567740 0.204 10,155,893.157880

4.571142 0.2041 10,443,888.832082

4.574543 0.2041 10,329,900.344622

4.577943 0.204 10,469,049.351326

4.581345 0.2041 10,780,093.871559

4.584747 0.2041 10,877,434.319921

4.588147 0.204 11,200,903.042027

4.591548 0.2041 11,630,499.128871

4.594950 0.2041 11,984,544.631901

4.598350 0.204 12,407,363.407310

4.601752 0.2041 12,742,705.697150

4.605153 0.2041 13,287,031.337741

4.608553 0.204 14,210,743.366017

4.611955 0.2041 14,613,196.571458

4.615357 0.2041 14,532,316.957704

4.618757 0.204 14,670,874.971124

4.622158 0.2041 15,188,195.510198

4.625560 0.2041 15,485,660.495744

4.628960 0.204 14,846,613.307958

4.632362 0.2041 14,648,244.545290

4.635763 0.2041 14,758,359.103026

4.639163 0.204 14,093,213.837542

4.642565 0.2041 13,445,921.175881

4.645967 0.2041 12,762,794.862550

4.649367 0.204 12,149,272.694770

4.652768 0.2041 11,814,910.826302

4.656170 0.2041 11,323,579.570590

4.659570 0.204 10,776,484.544651

4.662972 0.2041 10,501,526.283548

4.666373 0.2041 10,204,741.044646

4.669773 0.204 9,808,208.414317

4.673175 0.2041 9,569,521.914245

4.676577 0.2041 9,467,159.720756

4.679977 0.204 9,351,745.645711

4.683378 0.2041 9,182,380.462452

4.686780 0.2041 8,995,719.321145

4.690180 0.204 8,982,206.809307

4.693582 0.2041 9,401,657.246714

4.696983 0.2041 9,456,626.635319

4.700383 0.204 9,196,908.202869

4.703785 0.2041 9,164,949.437642

4.707187 0.2041 9,135,566.240493

4.710587 0.204 9,022,714.804408

4.713988 0.2041 8,844,545.063186

4.717390 0.2041 8,925,063.179184

4.720790 0.204 8,984,107.044786

4.724192 0.2041 9,096,093.160619

4.727593 0.2041 9,200,736.285541

4.730993 0.204 8,800,682.309509

4.734395 0.2041 8,773,007.948229

4.737797 0.2041 9,063,528.015052

4.741198 0.2041 8,937,627.242309

4.744598 0.204 8,836,243.310787

4.748000 0.2041 8,954,641.474654

4.751402 0.2041 9,275,904.731176

4.754802 0.204 9,303,248.738909

4.758203 0.2041 9,011,807.792615

4.761605 0.2041 8,962,948.775185

4.765005 0.204 8,757,155.348704

4.768407 0.2041 8,677,254.013261

4.771808 0.2041 8,931,962.283313

4.775208 0.204 9,073,353.013024

4.778610 0.2041 8,939,913.642032

4.782012 0.2041 8,859,811.387613

4.785412 0.204 9,211,916.064442

4.788813 0.2041 9,198,361.037388

4.792215 0.2041 9,068,026.495660

4.795615 0.204 9,081,055.589971

4.799017 0.2041 8,810,246.073902

4.802418 0.2041 8,994,689.206780

4.805818 0.204 9,294,575.485983

4.809220 0.2041 9,194,306.446472

4.812622 0.2041 9,092,911.776475

4.816022 0.204 9,131,336.562684

4.819423 0.2041 9,181,006.857532

4.822825 0.2041 9,183,722.556243

4.826225 0.204 9,101,784.090449

4.829627 0.2041 9,325,063.610928

4.833028 0.2041 9,303,495.983858

4.836428 0.204 8,936,497.544159

4.839830 0.2041 8,813,504.729433

4.843232 0.2041 8,592,198.875507

4.846632 0.204 8,830,932.965835

4.850033 0.2041 9,001,881.017441

4.853435 0.2041 8,949,345.884702

4.856835 0.204 9,219,304.127540

4.860237 0.2041 9,264,568.950853

4.863638 0.2041 9,162,632.582709

4.867038 0.204 9,047,758.727729

4.870440 0.2041 8,952,546.313293

4.873842 0.2041 9,064,413.252234

4.877242 0.204 9,118,440.824686

4.880643 0.2041 9,077,014.810261

4.884045 0.2041 9,059,692.089496

4.887445 0.204 9,184,622.805841

4.890847 0.2041 9,366,421.842644

4.894248 0.2041 9,201,024.290897

4.897648 0.204 8,983,377.928612

4.901050 0.2041 8,928,029.353890

4.904452 0.2041 9,037,456.094935

4.907852 0.204 8,915,796.096816

4.911253 0.2041 8,644,564.840149

4.914655 0.2041 8,826,243.886983

4.918055 0.204 8,846,897.556992

4.921457 0.2041 8,819,288.568864

4.924858 0.2041 9,090,542.520801

4.928258 0.204 9,031,088.617623

4.931660 0.2041 8,879,361.288415

4.935062 0.2041 8,905,638.414343

4.938462 0.204 9,061,473.245145

4.941863 0.2041 9,296,529.891869

4.945265 0.2041 9,207,138.570881

4.948665 0.204 9,063,130.768049

4.952067 0.2041 9,003,309.416163

4.955468 0.2041 9,031,898.022441

4.958868 0.204 9,007,117.331878

4.962270 0.2041 9,021,825.765573

4.965672 0.2041 8,988,973.139137

4.969072 0.204 8,888,075.203239

4.972473 0.2041 9,127,112.013830

4.975875 0.2041 9,010,292.215986

4.979275 0.204 8,830,809.579199

4.982677 0.2041 8,956,649.367867

4.986078 0.2041 8,856,578.732131

4.989478 0.204 8,771,317.148868

4.992880 0.2041 9,038,764.624248

4.996282 0.2041 9,082,730.604965

4.999682 0.204 9,055,573.176967

5.003083 0.2041 9,105,207.218080

5.006485 0.2041 8,825,654.233476

5.009885 0.204 8,803,389.530098

5.013287 0.2041 9,034,130.213694

5.016688 0.2041 9,035,436.315145

5.020088 0.204 8,888,263.700701

5.023490 0.2041 8,856,077.102480

5.026892 0.2041 8,918,923.907612

5.030292 0.204 9,036,857.250222

5.033693 0.2041 9,121,907.223473

5.037095 0.2041 8,900,636.780992

5.040495 0.204 8,796,597.410099

5.043897 0.2041 8,983,320.786445

5.047298 0.2041 8,927,834.527011

5.050698 0.204 9,073,340.760278

5.054100 0.2041 9,305,262.262231

5.057502 0.2041 9,102,970.818777

5.060902 0.204 9,064,848.680020

5.064303 0.2041 9,326,278.722874

5.067705 0.2041 9,289,004.799995

5.071105 0.204 9,129,077.750172

5.074507 0.2041 9,370,343.189084

5.077908 0.2041 9,368,572.857322

5.081308 0.204 9,240,608.661028

5.084710 0.2041 9,311,599.800651

5.088112 0.2041 9,480,641.838003

5.091513 0.2041 9,842,091.100705

5.094913 0.204 10,133,254.549461

5.098315 0.2041 10,485,277.346598

5.101717 0.2041 10,716,933.897657

5.105117 0.204 11,288,020.086550

5.108518 0.2041 11,930,830.422562

5.111920 0.2041 12,246,757.046921

5.115320 0.204 13,034,711.958105

5.118722 0.2041 13,967,268.061682

5.122123 0.2041 14,685,273.744805

5.125523 0.204 15,247,722.524119

5.128925 0.2041 15,865,393.019341

5.132327 0.2041 16,256,431.203988

5.135727 0.204 16,179,791.114320

5.139128 0.2041 15,987,906.183089

5.142530 0.2041 16,108,517.804921

5.145930 0.204 16,039,270.718959

5.149332 0.2041 15,266,486.248927

5.152733 0.2041 15,000,577.025016

5.156133 0.204 14,580,957.948003

5.159535 0.2041 13,468,989.725316

5.162937 0.2041 13,009,306.505717

5.166337 0.204 12,581,849.492034

5.169738 0.2041 11,834,647.046205

5.173140 0.2041 11,391,110.103045

5.176540 0.204 11,004,753.495210

5.179942 0.2041 10,651,751.983168

5.183343 0.2041 10,495,027.014856

5.186743 0.204 10,281,496.381453

5.190145 0.2041 10,072,944.955122

5.193547 0.2041 9,929,591.916259

5.196947 0.204 9,571,802.305783

5.200348 0.2041 9,558,311.076343

5.203750 0.2041 9,503,571.150525

5.207150 0.204 9,306,215.508176

5.210552 0.2041 9,405,806.276154

5.213953 0.2041 9,146,761.360971

5.217353 0.204 9,097,139.535963

5.220755 0.2041 9,134,122.449713

5.224157 0.2041 9,120,049.797335

5.227557 0.204 9,429,167.201463

5.230958 0.2041 9,366,549.021574

5.234360 0.2041 9,140,571.667409

5.237760 0.204 9,132,289.227409

5.241162 0.2041 9,037,951.460423

5.244563 0.2041 8,980,912.885322

5.247963 0.204 8,946,461.924207

5.251365 0.2041 8,884,992.416025

5.254767 0.2041 9,159,132.946091

5.258167 0.204 9,295,138.177483

5.261568 0.2041 9,000,537.706131

5.264970 0.2041 8,945,888.576477

5.268370 0.204 9,219,877.858027

5.271772 0.2041 9,281,080.869994

5.275173 0.2041 9,044,995.619085

5.278573 0.204 8,954,005.638650

5.281975 0.2041 8,905,975.950279

5.285377 0.2041 8,907,606.216123

5.288777 0.204 9,052,976.942525

5.292178 0.2041 9,055,828.619033

5.295580 0.2041 9,096,156.710827

5.298980 0.204 9,006,367.144786

5.302382 0.2041 8,837,130.515419

5.305783 0.2041 8,926,744.080215

5.309183 0.204 9,166,785.638826

5.312585 0.2041 9,134,489.296027

5.315987 0.2041 9,114,222.285058

5.319387 0.204 9,111,347.932447

5.322788 0.2041 8,874,429.341205

5.326190 0.2041 8,936,972.977450

5.329590 0.204 9,052,401.301837

5.332992 0.2041 9,017,727.477129

5.336393 0.2041 8,944,332.749003

5.339793 0.204 8,862,286.518368

5.343195 0.2041 8,784,763.552934

5.346597 0.2041 8,891,954.774775

5.349997 0.204 8,861,978.476767

5.353398 0.2041 8,962,254.823404

5.356800 0.2041 9,287,958.423711

5.360200 0.204 9,101,086.798828

5.363602 0.2041 9,082,106.568128

5.367003 0.2041 9,079,212.064670

5.370403 0.204 8,952,130.517184

5.373805 0.2041 9,050,101.989673

5.377207 0.2041 8,877,627.488960

5.380607 0.204 8,824,945.940404

5.384008 0.2041 8,991,890.239833

5.387410 0.2041 9,004,389.681851

5.390810 0.204 9,081,137.230333

5.394212 0.2041 8,976,304.088876

5.397613 0.2041 8,921,705.617820

5.401013 0.204 9,013,729.115104

5.404415 0.2041 8,894,023.106282

5.407817 0.2041 8,813,411.952291

5.411217 0.204 8,815,260.597498

5.414618 0.2041 8,877,235.673093

5.418020 0.2041 8,894,952.121225

5.421420 0.204 9,060,326.884516

5.424822 0.2041 9,146,681.700884

5.428223 0.2041 8,900,381.483617

5.431625 0.2041 8,975,239.414967

5.435025 0.204 9,177,686.462174

5.438427 0.2041 8,993,606.365168

5.441828 0.2041 8,968,006.525122

5.445228 0.204 9,183,406.120920

5.448630 0.2041 9,140,562.032296

5.452032 0.2041 8,960,377.082019

5.455432 0.204 9,035,700.952620

5.458833 0.2041 8,948,519.281448

5.462235 0.2041 8,674,928.347789

5.465635 0.204 8,780,278.563085

5.469037 0.2041 8,776,756.928158

5.472438 0.2041 8,859,854.920972

5.475838 0.204 9,021,641.514648

5.479240 0.2041 8,828,864.836379

5.482642 0.2041 8,802,447.641910

5.486042 0.204 8,864,307.900847

5.489443 0.2041 8,913,813.760304

5.492845 0.2041 8,860,431.842628

5.496245 0.204 8,734,178.926285

5.499647 0.2041 8,828,273.038608

5.503048 0.2041 9,013,044.117703

5.506448 0.204 8,998,791.816554

5.509850 0.2041 8,989,951.164698

5.513252 0.2041 9,095,938.845276

5.516652 0.204 8,807,353.593089

5.520053 0.2041 8,805,620.108928

5.523455 0.2041 9,011,419.057328

5.526855 0.204 9,055,005.442657

5.530257 0.2041 9,355,694.327678

5.533658 0.2041 9,314,701.380594

5.537058 0.204 8,939,364.794406

5.540460 0.2041 9,004,596.384641

5.543862 0.2041 9,377,575.489833

5.547262 0.204 9,393,694.585986

5.550663 0.2041 9,248,253.671088

5.554065 0.2041 9,126,543.982889

5.557465 0.204 8,972,692.795023

5.560867 0.2041 8,985,118.475870

5.564268 0.2041 8,973,689.846595

5.567668 0.204 8,781,243.421968

5.571070 0.2041 8,849,099.597727

5.574472 0.2041 9,057,378.605339

5.577872 0.204 9,089,215.529063

5.581273 0.2041 9,151,665.463316

5.584675 0.2041 9,006,887.199116

5.588075 0.204 8,941,266.013914

5.591477 0.2041 8,898,582.587875

5.594878 0.2041 8,727,305.743814

5.598278 0.204 8,894,790.729866

5.601680 0.2041 8,997,047.486741

5.605082 0.2041 8,975,142.789710

5.608482 0.204 8,932,161.306808

5.611883 0.2041 8,780,660.951011

5.615285 0.2041 9,017,373.423278

5.618685 0.204 8,957,240.393234

5.622087 0.2041 8,700,422.396993

5.625488 0.2041 9,192,234.957453

5.628888 0.204 9,276,954.363373

5.632290 0.2041 8,880,335.580951

5.635692 0.2041 8,953,214.810379

5.639092 0.204 9,062,931.511726

5.642493 0.2041 8,898,191.132734

5.645895 0.2041 8,700,563.943625

5.649295 0.204 8,731,380.055291

5.652697 0.2041 9,054,186.400348

5.656098 0.2041 9,100,773.311275

5.659498 0.204 8,976,623.771737

5.662900 0.2041 8,977,319.453675

5.666302 0.2041 8,791,370.220512

5.669702 0.204 8,712,465.802618

5.673103 0.2041 8,818,815.849888

5.676505 0.2041 8,772,827.222708

5.679905 0.204 8,819,764.268944

5.683307 0.2041 9,017,651.038660

5.686708 0.2041 9,161,591.030975

5.690108 0.204 9,158,365.399022

5.693510 0.2041 8,884,149.135539

5.696912 0.2041 8,866,974.051658

5.700312 0.204 9,017,939.832621

5.703713 0.2041 8,899,119.301727

5.707115 0.2041 8,846,833.077353

5.710515 0.204 8,913,926.807028

5.713917 0.2041 8,845,899.964934

5.717318 0.2041 8,859,376.085410

5.720718 0.204 8,857,186.078710

5.724120 0.2041 8,857,670.897373

5.727522 0.2041 9,023,483.224228

5.730922 0.204 8,976,797.155560

5.734323 0.2041 8,838,457.595594

5.737725 0.2041 8,845,429.353112

5.741125 0.204 8,737,871.661688

5.744527 0.2041 8,599,654.027420

5.747928 0.2041 8,728,751.956500

5.751328 0.204 8,946,051.847270

5.754730 0.2041 8,956,345.702229

5.758132 0.2041 8,871,591.754283

5.761532 0.204 8,908,612.222858

5.764933 0.2041 8,692,315.741380

5.768335 0.2041 8,715,166.232674

5.771735 0.204 8,936,394.950345

5.775137 0.2041 8,786,789.542088

5.778538 0.2041 8,732,853.332647

5.781940 0.2041 8,706,327.236617

5.785340 0.204 8,686,381.536812

5.788742 0.2041 8,898,089.513131

5.792143 0.2041 8,916,450.786871

5.795543 0.204 8,783,109.209729

5.798945 0.2041 8,805,850.386256

5.802347 0.2041 8,797,481.444517

5.805747 0.204 8,717,793.255495

5.809148 0.2041 8,880,880.202752

5.812550 0.2041 9,028,437.008926

5.815950 0.204 8,825,708.818158

5.819352 0.2041 8,960,826.727433

5.822753 0.2041 9,042,085.937196

5.826153 0.204 8,797,051.102017

5.829555 0.2041 8,878,321.066280

5.832957 0.2041 8,932,682.195815

5.836357 0.204 8,783,842.428176

5.839758 0.2041 8,887,963.901953

5.843160 0.2041 8,921,318.002919

5.846560 0.204 8,747,643.961711

5.849962 0.2041 8,676,847.058230

5.853363 0.2041 8,670,762.242159

5.856763 0.204 8,871,318.326895

5.860165 0.2041 8,846,492.366381

5.863567 0.2041 8,791,415.618362

5.866967 0.204 9,117,935.717303

5.870368 0.2041 9,058,405.178994

5.873770 0.2041 8,851,184.266831

5.877170 0.204 8,889,207.033563

5.880572 0.2041 8,739,373.067761

5.883973 0.2041 8,884,385.836477

5.887373 0.204 9,161,157.706423

5.890775 0.2041 8,892,389.496470

5.894177 0.2041 8,778,375.593004

5.897577 0.204 8,768,871.306431

5.900978 0.2041 8,707,340.544200

5.904380 0.2041 8,829,846.815273

5.907780 0.204 8,697,427.294291

5.911182 0.2041 8,652,761.495846

5.914583 0.2041 9,024,432.387271

5.917983 0.204 8,976,459.878170

5.921385 0.2041 8,710,332.149819

5.924787 0.2041 8,710,964.169097

5.928187 0.204 8,737,053.495933

5.931588 0.2041 8,689,998.055947

5.934990 0.2041 8,523,038.247949

5.938390 0.204 8,777,556.491369

5.941792 0.2041 8,920,583.825062

5.945193 0.2041 8,652,586.869407

5.948593 0.204 8,680,966.739389

5.951995 0.2041 8,677,655.095244

5.955397 0.2041 8,615,415.666865

5.958797 0.204 8,833,897.813789

5.962198 0.2041 8,826,696.859930

5.965600 0.2041 8,657,722.884265

5.969000 0.204 8,721,593.117436

5.972402 0.2041 8,799,780.335349

5.975803 0.2041 8,939,456.865728

5.979203 0.204 8,979,235.067147

5.982605 0.2041 8,869,971.301589

5.986007 0.2041 8,749,468.328853

5.989407 0.204 8,632,201.449898

5.992808 0.2041 8,563,114.240174

5.996210 0.2041 8,580,751.984162

5.999610 0.204 8,785,015.042163

6.003012 0.2041 8,840,768.232362

6.006413 0.2041 8,773,986.288878

6.009813 0.204 8,737,955.106592

6.013215 0.2041 8,665,583.677269

6.016617 0.2041 8,706,989.456250

6.020017 0.204 8,729,323.335985

6.023418 0.2041 8,796,704.268215

6.026820 0.2041 9,010,092.744468

6.030220 0.204 9,059,047.153034

6.033622 0.2041 8,935,419.137824

6.037023 0.2041 8,873,208.479200

6.040423 0.204 8,772,704.993451

6.043825 0.2041 8,822,277.250785

6.047227 0.2041 9,039,904.943733

6.050627 0.204 8,933,949.530955

6.054028 0.2041 8,759,388.340479

6.057430 0.2041 8,757,292.165414

6.060830 0.204 8,658,817.047005

6.064232 0.2041 8,651,855.576512

6.067633 0.2041 8,895,912.621787

6.071033 0.204 8,985,040.680388

6.074435 0.2041 8,759,324.558562

6.077837 0.2041 8,723,837.856615

6.081237 0.204 8,808,788.444249

6.084638 0.2041 8,794,080.675042

6.088040 0.2041 8,853,407.692151

6.091440 0.204 8,687,561.081237

6.094842 0.2041 8,509,653.799234

6.098243 0.2041 8,731,417.732061

6.101643 0.204 8,938,825.868195

6.105045 0.2041 8,920,887.964987

6.108447 0.2041 8,867,165.212478

6.111847 0.204 9,021,731.251512

6.115248 0.2041 9,278,712.990781

6.118650 0.2041 9,112,199.707063

6.122050 0.204 8,767,325.571433

6.125452 0.2041 8,793,843.170148

6.128853 0.2041 8,998,099.792271

6.132255 0.2041 8,944,183.885859

6.135655 0.204 8,736,063.822374

6.139057 0.2041 8,759,863.829133

6.142458 0.2041 8,803,116.195807

6.145858 0.204 8,785,193.284714

6.149260 0.2041 8,742,762.973768

6.152662 0.2041 8,721,802.497248

6.156062 0.204 8,960,144.447007

6.159463 0.2041 8,852,825.315623

6.162865 0.2041 8,594,070.085074

6.166265 0.204 8,813,691.039217

6.169667 0.2041 8,772,821.373518

6.173068 0.2041 8,632,830.635536

6.176468 0.204 8,566,929.164173

6.179870 0.2041 8,466,354.594815

6.183272 0.2041 8,645,818.400218

6.186672 0.204 8,807,549.566394

6.190073 0.2041 8,848,773.929394

6.193475 0.2041 8,830,450.062582

6.196875 0.204 8,831,466.001631

6.200277 0.2041 8,851,044.727838

6.203678 0.2041 8,911,423.315403

6.207078 0.204 8,838,579.168258

6.210480 0.2041 8,638,649.224028

6.213882 0.2041 8,554,487.365792

6.217282 0.204 8,563,921.156497

6.220683 0.2041 8,742,973.596533

6.224085 0.2041 8,739,926.191628

6.227485 0.204 8,615,183.894898

6.230887 0.2041 8,859,368.802017

6.234288 0.2041 8,865,479.923181

6.237688 0.204 8,454,323.256239

6.241090 0.2041 8,436,501.043657

6.244492 0.2041 8,890,710.955206

6.247892 0.204 8,875,631.430626

6.251293 0.2041 8,603,116.755531

6.254695 0.2041 8,653,164.554166

6.258095 0.204 8,771,779.375205

6.261497 0.2041 8,754,498.684954

6.264898 0.2041 8,708,353.276281

6.268298 0.204 8,900,847.785282

6.271700 0.2041 8,843,915.801201

6.275102 0.2041 8,761,229.373344

6.278502 0.204 8,830,338.341146

6.281903 0.2041 8,732,263.728742

6.285305 0.2041 8,739,806.477203

6.288705 0.204 8,890,454.414937

6.292107 0.2041 8,719,205.598473

6.295508 0.2041 8,505,771.196170

6.298908 0.204 8,594,142.240552

6.302310 0.2041 8,671,055.635743

6.305712 0.2041 8,608,079.675362

6.309112 0.204 8,607,939.747506

6.312513 0.2041 8,966,592.854401

6.315915 0.2041 9,027,417.170124

6.319315 0.204 8,803,157.981192

6.322717 0.2041 8,755,818.486916

6.326118 0.2041 8,797,641.233250

6.329518 0.204 8,790,445.984517

6.332920 0.2041 8,720,710.423791

6.336322 0.2041 8,515,005.591441

6.339722 0.204 8,483,020.389695

6.343123 0.2041 8,800,035.273277

6.346525 0.2041 8,937,356.321339

6.349925 0.204 8,875,309.010084

6.353327 0.2041 8,926,027.935423

6.356728 0.2041 8,956,321.822619

6.360128 0.204 8,763,456.449433

6.363530 0.2041 8,849,582.862727

6.366932 0.2041 8,823,721.651888

6.370332 0.204 8,663,932.545256

6.373733 0.2041 8,772,901.542833

6.377135 0.2041 8,638,221.481611

6.380535 0.204 8,637,531.167695

6.383937 0.2041 8,910,583.466030

6.387338 0.2041 8,746,507.255838

6.390738 0.204 8,455,919.221436

6.394140 0.2041 8,470,241.514240

6.397542 0.2041 8,646,286.913403

6.400942 0.204 8,817,807.282047

6.404343 0.2041 8,618,776.620425

6.407745 0.2041 8,534,111.266837

6.411145 0.204 8,866,992.488020

6.414547 0.2041 8,676,572.936536

6.417948 0.2041 8,374,989.442784

6.421348 0.204 8,817,123.501086

6.424750 0.2041 8,908,837.095417

6.428152 0.2041 8,600,708.312608

6.431552 0.204 8,742,628.541775

6.434953 0.2041 8,902,966.881423

6.438355 0.2041 8,718,776.096854

6.441755 0.204 8,566,161.769333

6.445157 0.2041 8,728,519.907438

6.448558 0.2041 8,783,889.391320

6.451958 0.204 8,855,547.379785

6.455360 0.2041 8,985,835.710258

6.458762 0.2041 8,806,109.095302

6.462162 0.204 8,825,873.558260

6.465563 0.2041 8,921,417.183484

6.468965 0.2041 8,923,358.323234

6.472365 0.204 8,883,924.332868

6.475767 0.2041 8,652,809.761187

6.479168 0.2041 8,736,881.138074

6.482568 0.204 8,879,964.319587

6.485970 0.2041 8,878,967.175904

6.489372 0.2041 8,872,474.968545

6.492773 0.2041 8,815,901.829510

6.496173 0.204 8,653,794.873781

6.499575 0.2041 8,571,093.163337

6.502977 0.2041 8,473,792.277336

6.506377 0.204 8,451,514.876559

6.509778 0.2041 8,895,333.793816

6.513180 0.2041 8,709,834.607653

6.516580 0.204 8,445,996.119850

6.519982 0.2041 8,917,086.805619

6.523383 0.2041 8,946,387.567205

6.526783 0.204 8,607,184.055253

6.530185 0.2041 8,728,126.523653

6.533587 0.2041 8,747,830.766286

6.536987 0.204 8,475,776.730907

6.540388 0.2041 8,506,344.075405

6.543790 0.2041 8,680,559.083656

6.547190 0.204 8,846,665.568714

6.550592 0.2041 8,804,017.397188

6.553993 0.2041 8,727,260.048966

6.557393 0.204 8,750,506.338973

6.560795 0.2041 8,613,006.231297

6.564197 0.2041 8,682,344.973331

6.567597 0.204 8,897,522.603014

6.570998 0.2041 8,747,955.139579

6.574400 0.2041 8,737,564.024576

6.577800 0.204 8,909,288.834220

6.581202 0.2041 8,915,099.298059

6.584603 0.2041 8,924,496.380507

6.588003 0.204 8,872,089.844357

6.591405 0.2041 8,789,439.429102

6.594807 0.2041 8,796,176.820476

6.598207 0.204 8,751,963.560640

6.601608 0.2041 8,526,280.099092

6.605010 0.2041 8,602,010.562940

6.608410 0.204 8,761,165.244127

6.611812 0.2041 8,754,054.222926

6.615213 0.2041 8,795,046.178096

6.618613 0.204 8,718,301.890142

6.622015 0.2041 8,681,344.390696

6.625417 0.2041 8,849,371.745400

6.628817 0.204 8,822,946.882095

6.632218 0.2041 8,529,389.427837

6.635620 0.2041 8,527,499.992862

6.639020 0.204 8,653,510.895637

6.642422 0.2041 8,783,806.092958

6.645823 0.2041 8,892,399.904276

6.649223 0.204 8,598,796.236420

6.652625 0.2041 8,324,902.966575

6.656027 0.2041 8,475,728.687905

6.659427 0.204 8,755,903.244666

6.662828 0.2041 8,982,758.165082

6.666230 0.2041 8,818,321.500399

6.669630 0.204 8,659,597.974780

6.673032 0.2041 8,582,458.246696

6.676433 0.2041 8,486,058.222405

6.679833 0.204 8,794,132.005278

6.683235 0.2041 8,839,507.529410

6.686637 0.2041 8,639,283.932698

6.690037 0.204 8,678,874.351822

6.693438 0.2041 8,780,755.955039

6.696840 0.2041 8,836,056.169252

6.700240 0.204 8,765,831.183769

6.703642 0.2041 8,794,854.942832

6.707043 0.2041 8,752,645.915395

6.710443 0.204 8,577,578.658391

6.713845 0.2041 8,878,563.694412

6.717247 0.2041 9,018,764.665748

6.720647 0.204 8,687,961.451884

6.724048 0.2041 8,655,844.847818

6.727450 0.2041 8,754,931.082351

6.730850 0.204 8,772,841.607587

6.734252 0.2041 8,810,268.666080

6.737653 0.2041 8,735,882.256573

6.741053 0.204 8,719,169.545365

6.744455 0.2041 8,784,807.966472

6.747857 0.2041 8,494,648.859496

6.751257 0.204 8,670,123.922800

6.754658 0.2041 8,961,815.340338

6.758060 0.2041 8,613,286.243579

6.761460 0.204 8,806,366.496012

6.764862 0.2041 9,038,477.113290

6.768263 0.2041 8,824,841.724979

6.771663 0.204 8,870,428.972608

6.775065 0.2041 8,999,276.806569

6.778467 0.2041 8,815,147.033518

6.781867 0.204 8,799,123.091163

6.785268 0.2041 8,886,311.217546

6.788670 0.2041 8,689,366.330037

6.792070 0.204 8,767,223.729545

6.795472 0.2041 9,024,843.889792

6.798873 0.2041 8,887,328.142527

6.802273 0.204 8,742,435.438314

6.805675 0.2041 8,952,080.145290

6.809077 0.2041 9,066,016.174128

6.812477 0.204 9,077,235.114452

6.815878 0.2041 9,001,659.098365

6.819280 0.2041 8,954,050.401553

6.822680 0.204 8,975,512.701824

6.826082 0.2041 8,880,247.587133

6.829483 0.2041 8,927,884.690899

6.832883 0.204 9,008,827.428321

6.836285 0.2041 8,960,262.604945

6.839687 0.2041 9,011,614.359703

6.843087 0.204 8,949,643.091457

6.846488 0.2041 8,874,464.179285

6.849890 0.2041 8,849,199.135249

6.853292 0.2041 8,648,390.704492

6.856692 0.204 8,928,491.134610

6.860093 0.2041 9,088,573.662760

6.863495 0.2041 8,795,049.663529

6.866895 0.204 8,859,158.996288

6.870297 0.2041 9,007,290.494806

6.873698 0.2041 8,751,721.236942

6.877098 0.204 8,601,720.800938

6.880500 0.2041 8,863,421.350658

6.883902 0.2041 8,810,060.939624

6.887302 0.204 8,675,344.324790

6.890703 0.2041 8,651,764.152082

6.894105 0.2041 8,643,672.478601

6.897505 0.204 8,699,405.267182

6.900907 0.2041 8,635,666.807091

6.904308 0.2041 8,611,693.942833

6.907708 0.204 8,915,844.162035

6.911110 0.2041 9,086,169.776277

6.914512 0.2041 8,791,138.637712

6.917912 0.204 8,805,065.364005

6.921313 0.2041 8,845,377.916556

6.924715 0.2041 8,758,672.409884

6.928115 0.204 8,861,502.386059

6.931517 0.2041 8,760,648.362222

6.934918 0.2041 8,740,600.247973

6.938318 0.204 8,750,932.484716

6.941720 0.2041 8,696,427.393961

6.945122 0.2041 8,740,612.605413

6.948522 0.204 8,651,112.460167

6.951923 0.2041 8,677,506.182302

6.955325 0.2041 8,815,587.831723

6.958725 0.204 8,955,284.811517

6.962127 0.2041 8,823,950.616379

6.965528 0.2041 8,525,030.939884

6.968928 0.204 8,621,754.098076

6.972330 0.2041 8,692,101.729563

6.975732 0.2041 8,720,459.146089

6.979132 0.204 8,862,751.272796

6.982533 0.2041 8,868,350.246350

6.985935 0.2041 8,802,996.236716

6.989335 0.204 8,739,774.939573

6.992737 0.2041 8,698,464.684736

6.996138 0.2041 8,703,674.097648

6.999538 0.204 8,814,541.665722

7.002940 0.2041 8,827,810.639663

7.006342 0.2041 8,612,719.409363

7.009742 0.204 8,680,222.192782

7.013143 0.2041 8,789,696.765230

7.016545 0.2041 8,509,687.555490

7.019945 0.204 8,591,371.381575

7.023347 0.2041 8,934,550.749958

7.026748 0.2041 8,828,808.838878

7.030148 0.204 8,791,677.561297

7.033550 0.2041 8,626,088.014824

7.036952 0.2041 8,423,241.886649

7.040352 0.204 8,704,673.133087

7.043753 0.2041 8,681,395.938149

7.047155 0.2041 8,654,950.246112

7.050555 0.204 8,813,110.418661

7.053957 0.2041 8,784,781.067029

7.057358 0.2041 8,813,971.748959

7.060758 0.204 8,771,970.110519

7.064160 0.2041 8,673,534.712408

7.067562 0.2041 8,618,290.615125

7.070962 0.204 8,564,213.095350

7.074363 0.2041 8,734,797.740458

7.077765 0.2041 8,957,509.306735

7.081165 0.204 8,926,279.823960

7.084567 0.2041 8,728,872.339469

7.087968 0.2041 8,619,704.190502

7.091368 0.204 8,843,924.750767

7.094770 0.2041 8,862,836.778229

7.098172 0.2041 8,562,132.519664

7.101572 0.204 8,542,448.955573

7.104973 0.2041 8,791,105.660843

7.108375 0.2041 8,907,007.788041

7.111775 0.204 8,747,746.467877

7.115177 0.2041 8,737,671.971944

7.118578 0.2041 8,775,200.706622

7.121978 0.204 8,678,633.095309

7.125380 0.2041 8,695,847.737189

7.128782 0.2041 8,843,309.628462

7.132182 0.204 8,824,506.442880

7.135583 0.2041 8,693,519.147302

7.138985 0.2041 8,703,183.154299

7.142385 0.204 8,574,178.140741

7.145787 0.2041 8,430,319.589513

7.149188 0.2041 8,634,845.010182

7.152588 0.204 8,894,966.983346

7.155990 0.2041 8,634,582.414794

7.159392 0.2041 8,605,909.715327

7.162792 0.204 8,924,499.863866

7.166193 0.2041 8,949,016.390791

7.169595 0.2041 8,881,856.985679

7.172995 0.204 8,722,380.609983

7.176397 0.2041 8,732,257.108370

7.179798 0.2041 8,833,241.803756

7.183198 0.204 8,812,803.404120

7.186600 0.2041 8,711,012.055784

7.190002 0.2041 8,744,995.139489

7.193402 0.204 8,780,492.657342

7.196803 0.2041 8,701,157.479745

7.200205 0.2041 8,806,299.633865

7.203607 0.2041 8,766,586.625662

7.207007 0.204 8,641,240.381566

7.210408 0.2041 8,787,322.120502

7.213810 0.2041 8,893,888.250181

7.217210 0.204 8,873,409.363226

7.220612 0.2041 8,957,282.173621

7.224013 0.2041 9,043,618.144842

7.227413 0.204 8,906,370.315378

7.230815 0.2041 8,913,428.243171

7.234217 0.2041 9,133,077.063038

7.237617 0.204 8,943,902.291157

7.241018 0.2041 8,775,152.494505

7.244420 0.2041 8,627,870.732924

7.247820 0.204 8,568,510.490748

7.251222 0.2041 8,853,104.079928

7.254623 0.2041 8,814,548.811455

7.258023 0.204 8,740,293.906357

7.261425 0.2041 8,790,316.020352

7.264827 0.2041 8,855,749.266688

7.268227 0.204 8,764,813.995658

7.271628 0.2041 8,726,899.591118

7.275030 0.2041 8,826,622.636808

7.278430 0.204 8,781,466.306698

7.281832 0.2041 8,668,597.936555

7.285233 0.2041 8,617,906.586237

7.288633 0.204 8,750,326.773146

7.292035 0.2041 8,703,640.274915

7.295437 0.2041 8,624,950.896816

7.298837 0.204 8,739,257.023475

7.302238 0.2041 8,715,799.695009

7.305640 0.2041 8,533,317.309544

7.309040 0.204 8,504,262.647136

7.312442 0.2041 8,582,284.753803

7.315843 0.2041 8,593,721.836393

7.319243 0.204 8,675,715.338003

7.322645 0.2041 8,773,467.493805

7.326047 0.2041 8,732,350.945789

7.329447 0.204 8,640,794.905812

7.332848 0.2041 8,561,963.893624

7.336250 0.2041 8,811,904.205746

7.339650 0.204 9,011,441.083465

7.343052 0.2041 8,795,447.020773

7.346453 0.2041 8,847,442.460263

7.349853 0.204 8,976,251.843040

7.353255 0.2041 8,873,379.974043

7.356657 0.2041 8,870,674.304468

7.360057 0.204 8,836,007.511358

7.363458 0.2041 8,958,216.144353

7.366860 0.2041 9,179,004.034786

7.370260 0.204 9,030,533.923351

7.373662 0.2041 8,899,443.632387

7.377063 0.2041 8,917,951.313862

7.380463 0.204 8,827,757.786985

7.383865 0.2041 8,939,949.905336

7.387267 0.2041 9,167,975.855867

7.390667 0.204 9,025,348.428255

7.394068 0.2041 8,989,633.225901

7.397470 0.2041 9,090,020.300614

7.400870 0.204 9,036,115.525975

7.404272 0.2041 8,954,286.695045

7.407673 0.2041 8,869,636.854844

7.411073 0.204 8,814,124.414715

7.414475 0.2041 8,715,397.231021

7.417877 0.2041 8,733,303.902853

7.421277 0.204 8,614,914.370370

7.424678 0.2041 8,784,081.607035

7.428080 0.2041 8,927,371.610778

7.431480 0.204 8,636,472.998659

7.434882 0.2041 8,854,506.238262

7.438283 0.2041 8,960,132.422557

7.441683 0.204 8,766,230.653484

7.445085 0.2041 8,747,753.367205

7.448487 0.2041 8,609,802.865480

7.451887 0.204 8,534,323.797883

7.455288 0.2041 8,699,734.998557

7.458690 0.2041 8,556,407.683057

7.462090 0.204 8,552,975.879159

7.465492 0.2041 8,986,434.407849

7.468893 0.2041 8,928,239.134732

7.472293 0.204 8,787,488.364356

7.475695 0.2041 8,673,273.246250

7.479097 0.2041 8,524,280.291847

7.482497 0.204 8,756,956.036856

7.485898 0.2041 8,834,782.641494

7.489300 0.2041 8,810,836.962926

7.492700 0.204 8,798,368.759270

7.496102 0.2041 8,588,689.388713

7.499503 0.2041 8,673,029.351552

7.502903 0.204 8,795,381.649992

7.506305 0.2041 8,738,373.775300

7.509707 0.2041 8,737,642.186355

7.513107 0.204 8,767,995.776804

7.516508 0.2041 8,928,585.295800

7.519910 0.2041 8,777,823.870912

7.523310 0.204 8,680,202.637150

7.526712 0.2041 8,783,937.449851

7.530113 0.2041 8,630,153.946866

7.533513 0.204 8,638,821.147594

7.536915 0.2041 8,800,217.303847

7.540317 0.2041 8,694,116.706154

7.543717 0.204 8,443,522.402808

7.547118 0.2041 8,520,390.289146

7.550520 0.2041 8,599,535.792374

7.553920 0.204 8,548,829.747857

7.557322 0.2041 8,662,295.697209

7.560723 0.2041 8,651,826.093954

7.564123 0.204 8,685,481.578775

7.567525 0.2041 8,722,233.373858

7.570927 0.2041 8,604,797.798810

7.574327 0.204 8,703,808.633192

7.577728 0.2041 8,691,584.022690

7.581130 0.2041 8,454,474.178829

7.584530 0.204 8,453,931.059747

7.587932 0.2041 8,637,198.218205

7.591333 0.2041 8,615,648.366174

7.594733 0.204 8,665,385.687410

7.598135 0.2041 8,917,544.431914

7.601537 0.2041 8,710,166.841315

7.604937 0.204 8,596,124.128358

7.608338 0.2041 8,860,939.050305

7.611740 0.2041 8,670,779.372270

7.615142 0.2041 8,472,002.748210

7.618542 0.204 8,684,327.541041

7.621943 0.2041 8,750,822.842153

7.625345 0.2041 8,529,546.620890

7.628745 0.204 8,704,143.608921

7.632147 0.2041 8,970,810.515535

7.635548 0.2041 8,671,552.689806

7.638948 0.204 8,677,528.436017

7.642350 0.2041 8,740,504.209327

7.645752 0.2041 8,485,151.216006

7.649152 0.204 8,642,575.823691

7.652553 0.2041 8,631,756.741002

7.655955 0.2041 8,626,178.626029

7.659355 0.204 8,788,378.929852

7.662757 0.2041 8,642,134.561909

7.666158 0.2041 8,749,977.122825

7.669558 0.204 8,714,677.468116

7.672960 0.2041 8,570,347.320324

7.676362 0.2041 8,643,754.124303

7.679762 0.204 8,583,593.088177

7.683163 0.2041 8,524,934.926852

7.686565 0.2041 8,562,551.138759

7.689965 0.204 8,686,248.906732

7.693367 0.2041 8,494,745.630590

7.696768 0.2041 8,284,343.436820

7.700168 0.204 8,558,351.823531

7.703570 0.2041 8,815,945.876402

7.706972 0.2041 8,739,644.763388

7.710372 0.204 8,570,971.231376

7.713773 0.2041 8,450,581.366954

7.717175 0.2041 8,333,861.881944

7.720575 0.204 8,461,660.122046

7.723977 0.2041 8,522,002.154998

7.727378 0.2041 8,535,470.657454

7.730778 0.204 8,709,845.560548

7.734180 0.2041 8,611,893.175447

7.737582 0.2041 8,502,205.071501

7.740982 0.204 8,554,821.826188

7.744383 0.2041 8,506,258.057434

7.747785 0.2041 8,705,578.304084

7.751185 0.204 8,890,038.102671

7.754587 0.2041 8,821,469.341048

7.757988 0.2041 8,874,321.336665

7.761388 0.204 8,829,336.659553

7.764790 0.2041 8,978,676.707841

7.768192 0.2041 9,349,595.520280

7.771592 0.204 9,628,833.473823

7.774993 0.2041 9,728,200.332946

7.778395 0.2041 9,808,633.035391

7.781795 0.204 10,155,486.594933

7.785197 0.2041 10,373,054.052914

7.788598 0.2041 10,382,984.753239

7.791998 0.204 10,400,533.560233

7.795400 0.2041 10,497,801.529154

7.798802 0.2041 10,656,437.343324

7.802202 0.204 10,462,280.573931

7.805603 0.2041 10,261,138.061878

7.809005 0.2041 10,458,105.233171

7.812405 0.204 10,251,181.479583

7.815807 0.2041 9,913,552.397649

7.819208 0.2041 9,916,943.778247

7.822608 0.204 9,879,740.035127

7.826010 0.2041 9,834,922.386943

7.829412 0.2041 9,595,127.305178

7.832812 0.204 9,343,529.334194

7.836213 0.2041 9,296,660.947915

7.839615 0.2041 9,105,819.077851

7.843015 0.204 8,978,872.918283

7.846417 0.2041 9,110,229.330776

7.849818 0.2041 9,004,576.397871

7.853218 0.204 8,863,932.463427

7.856620 0.2041 8,873,509.320798

7.860022 0.2041 8,808,334.062832

7.863422 0.204 8,801,410.320098

7.866823 0.2041 8,904,677.856950

7.870225 0.2041 8,861,856.831481

7.873625 0.204 8,751,506.491706

7.877027 0.2041 8,932,646.301811

7.880428 0.2041 8,913,079.820790

7.883828 0.204 8,725,976.233880

7.887230 0.2041 8,802,481.043603

7.890632 0.2041 8,860,738.169651

7.894032 0.204 8,788,649.195018

7.897433 0.2041 8,737,444.042880

7.900835 0.2041 8,686,466.314063

7.904235 0.204 8,673,641.113880

7.907637 0.2041 8,621,131.672763

7.911038 0.2041 8,460,277.745983

7.914438 0.204 8,656,798.313747

7.917840 0.2041 8,691,988.779060

7.921242 0.2041 8,391,537.420695

7.924642 0.204 8,401,911.906378

7.928043 0.2041 8,515,736.249575

7.931445 0.2041 8,541,849.798739

7.934845 0.204 8,604,975.475598

7.938247 0.2041 8,663,142.953394

7.941648 0.2041 8,731,751.790665

7.945048 0.204 8,679,731.591345

7.948450 0.2041 8,459,133.335638

7.951852 0.2041 8,655,107.237980

7.955253 0.2041 8,862,553.848983

7.958653 0.204 8,697,500.247821

7.962055 0.2041 8,709,102.404444

7.965457 0.2041 8,711,246.411426

7.968857 0.204 8,705,633.194583

7.972258 0.2041 8,718,869.225780

7.975660 0.2041 8,498,916.173926

7.979060 0.204 8,526,188.906601

7.982462 0.2041 8,799,310.873301

7.985863 0.2041 8,806,206.077467

7.989263 0.204 8,791,697.509125

7.992665 0.2041 8,786,081.811944

7.996067 0.2041 8,677,239.244509

7.999467 0.204 8,788,153.506446

8.002868 0.2041 8,987,315.240999

8.006270 0.2041 9,169,884.550977

8.009670 0.204 9,144,295.705421

8.013072 0.2041 9,460,566.957618

8.016473 0.2041 10,080,631.028007

8.019873 0.204 10,579,499.553895

8.023275 0.2041 11,468,642.424164

8.026677 0.2041 12,696,588.228744

8.030077 0.204 14,564,842.548318

8.033478 0.2041 16,946,127.633185

8.036880 0.2041 20,163,862.544630

8.040280 0.204 24,348,604.704249

8.043682 0.2041 28,681,416.381316

8.047083 0.2041 34,110,989.760007

8.050483 0.204 41,133,340.999005

8.053885 0.2041 48,862,396.201997

8.057287 0.2041 56,725,665.189846

8.060687 0.204 64,788,615.079875

8.064088 0.2041 73,557,615.640303

8.067490 0.2041 81,499,796.328627

8.070890 0.204 87,809,797.014100

8.074292 0.2041 94,404,112.265543

8.077693 0.2041 98,854,584.650328

8.081093 0.204 99,400,222.762705

8.084495 0.2041 98,507,912.737382

8.087897 0.2041 96,210,926.510771

8.091297 0.204 91,643,568.151200

8.094698 0.2041 83,226,325.723220

8.098100 0.2041 73,832,267.366203

8.101500 0.204 65,948,605.129142

8.104902 0.2041 57,266,075.439293

8.108303 0.2041 47,924,437.687697

8.111703 0.204 39,138,192.169674

8.115105 0.2041 32,431,315.676646

8.118507 0.2041 27,101,311.965900

8.121907 0.204 22,435,203.761483

8.125308 0.2041 18,839,150.385963

8.128710 0.2041 16,391,393.081483

8.132110 0.204 14,599,871.482374

8.135512 0.2041 13,119,763.127362

8.138913 0.2041 12,350,758.230195

8.142313 0.204 11,740,450.478419

8.145715 0.2041 11,320,761.675090

8.149117 0.2041 11,029,027.553941

8.152517 0.204 10,510,698.026537

8.155918 0.2041 10,395,696.610385

8.159320 0.2041 10,497,102.649137

8.162720 0.204 10,327,094.642237

8.166122 0.2041 10,273,973.599232

8.169523 0.2041 10,267,582.029781

8.172923 0.204 10,154,626.474693

8.176325 0.2041 9,947,933.823360

8.179727 0.2041 9,842,186.251897

8.183127 0.204 9,779,939.172458

8.186528 0.2041 9,758,856.074420

8.189930 0.2041 9,880,630.947412

8.193330 0.204 9,747,813.712909

8.196732 0.2041 9,530,464.169002

8.200133 0.2041 9,644,379.515604

8.203533 0.204 9,619,682.663139

8.206935 0.2041 9,459,950.291255

8.210337 0.2041 9,517,573.663897

8.213737 0.204 9,423,537.080110

8.217138 0.2041 9,310,604.166421

8.220540 0.2041 9,336,250.703013

8.223940 0.204 9,268,553.123896

8.227342 0.2041 9,369,687.053713

8.230743 0.2041 9,483,056.107277

8.234143 0.204 9,201,094.459617

8.237545 0.2041 9,148,091.325604

8.240947 0.2041 9,433,398.234602

8.244347 0.204 9,302,322.016924

8.247748 0.2041 9,247,710.121901

8.251150 0.2041 9,419,431.341022

8.254550 0.204 9,297,409.871693

8.257952 0.2041 9,115,060.408976

8.261353 0.2041 9,156,697.361123

8.264753 0.204 9,224,800.827817

8.268155 0.2041 9,149,206.143670

8.271557 0.2041 9,095,210.952196

8.274957 0.204 8,904,079.742775

8.278358 0.2041 8,889,190.008821

8.281760 0.2041 9,120,319.334368

8.285160 0.204 9,095,080.864281

8.288562 0.2041 8,839,341.291603

8.291963 0.2041 9,074,464.747832

8.295363 0.204 9,231,208.718717

8.298765 0.2041 8,893,227.824503

8.302167 0.2041 8,915,814.190697

8.305567 0.204 8,715,923.644828

8.308968 0.2041 8,555,245.470049

8.312370 0.2041 8,819,044.470489

8.315772 0.2041 8,935,491.947124

8.319172 0.204 9,051,798.749242

8.322573 0.2041 9,008,995.270046

8.325975 0.2041 8,915,998.315907

8.329375 0.204 8,951,396.380005

8.332777 0.2041 8,922,410.666189

8.336178 0.2041 8,870,727.083323

8.339578 0.204 8,930,715.856821

8.342980 0.2041 9,157,604.369659

8.346382 0.2041 9,016,403.160801

8.349782 0.204 8,752,462.654733

8.353183 0.2041 8,813,655.029041

8.356585 0.2041 8,906,476.339356

8.359985 0.204 8,899,984.184040

8.363387 0.2041 8,825,552.933427

8.366788 0.2041 8,735,473.038401

8.370188 0.204 8,878,527.905192

8.373590 0.2041 9,018,489.844170

8.376992 0.2041 8,842,612.177527

8.380392 0.204 8,868,370.379456

8.383793 0.2041 8,866,695.392760

8.387195 0.2041 8,762,074.646941

8.390595 0.204 8,756,666.716416

8.393997 0.2041 8,787,929.783814

8.397398 0.2041 8,903,669.712178

8.400798 0.204 8,976,703.214485

8.404200 0.2041 8,946,766.943087

8.407602 0.2041 9,077,049.675900

8.411002 0.204 9,097,634.626177

8.414403 0.2041 8,746,590.626862

8.417805 0.2041 8,893,903.820300

8.421205 0.204 9,110,953.805318

8.424607 0.2041 8,937,862.856658

8.428008 0.2041 8,953,224.704480

8.431408 0.204 9,083,663.098146

8.434810 0.2041 9,269,151.107788

8.438212 0.2041 9,315,314.517488

8.441612 0.204 9,287,313.538813

8.445013 0.2041 9,489,276.238386

8.448415 0.2041 9,812,818.703582

8.451815 0.204 10,106,896.336725

8.455217 0.2041 10,344,200.471964

8.458618 0.2041 10,489,565.640727

8.462018 0.204 10,476,237.303417

8.465420 0.2041 10,699,749.984039

8.468822 0.2041 10,865,354.819575

8.472222 0.204 10,587,808.431020

8.475623 0.2041 10,612,347.724000

8.479025 0.2041 10,650,170.003823

8.482425 0.204 10,285,548.738553

8.485827 0.2041 10,069,088.228523

8.489228 0.2041 9,851,580.989406

8.492628 0.204 9,481,626.523709

8.496030 0.2041 9,380,002.766219

8.499432 0.2041 9,206,414.539870

8.502832 0.204 8,908,748.566427

8.506233 0.2041 8,994,288.722163

8.509635 0.2041 8,957,469.004608

8.513035 0.204 8,734,185.922750

8.516437 0.2041 8,862,891.457056

8.519838 0.2041 8,937,512.150327

8.523238 0.204 8,773,526.082649

8.526640 0.2041 8,717,545.780279

8.530042 0.2041 8,707,782.464153

8.533442 0.204 8,723,034.654029

8.536843 0.2041 8,730,875.680561

8.540245 0.2041 8,821,635.661376

8.543645 0.204 8,851,811.706513

8.547047 0.2041 8,793,055.484150

8.550448 0.2041 8,957,709.652212

8.553848 0.204 8,900,918.089808

8.557250 0.2041 8,799,355.603312

8.560652 0.2041 8,951,181.453171

8.564052 0.204 8,904,009.475722

8.567453 0.2041 8,882,427.437775

8.570855 0.2041 8,936,565.853109

8.574255 0.204 8,822,568.063592

8.577657 0.2041 8,853,389.923403

8.581058 0.2041 8,873,588.752311

8.584458 0.204 8,824,318.975158

8.587860 0.2041 8,876,755.213894

8.591262 0.2041 8,965,096.612758

8.594662 0.204 8,942,839.549186

8.598063 0.2041 8,671,400.463107

8.601465 0.2041 8,555,753.719428

8.604865 0.204 8,705,724.907601

8.608267 0.2041 8,669,804.023747

8.611668 0.2041 8,475,958.539663

8.615068 0.204 8,625,151.348171

8.618470 0.2041 8,622,890.061977

8.621872 0.2041 8,436,940.724346

8.625272 0.204 8,760,167.543187

8.628673 0.2041 8,934,809.024561

8.632075 0.2041 8,893,993.951306

8.635475 0.204 8,890,686.302205

8.638877 0.2041 8,697,356.446384

8.642278 0.2041 8,628,991.617094

8.645678 0.204 8,795,797.576915

8.649080 0.2041 8,704,920.672486

8.652482 0.2041 8,385,012.930923

8.655883 0.2041 8,621,861.476996

8.659283 0.204 8,868,904.437621

8.662685 0.2041 8,768,018.058325

8.666087 0.2041 8,733,934.509879

8.669487 0.204 8,649,921.627755

8.672888 0.2041 8,689,417.097030

8.676290 0.2041 8,685,445.809127

8.679690 0.204 8,663,514.759732

8.683092 0.2041 8,744,140.987610

8.686493 0.2041 8,687,347.400800

8.689893 0.204 8,606,549.854559

8.693295 0.2041 8,476,622.898920

8.696697 0.2041 8,524,983.786042

8.700097 0.204 8,631,545.962424

8.703498 0.2041 8,519,510.260203

8.706900 0.2041 8,608,609.275544

8.710300 0.204 8,605,861.866889

8.713702 0.2041 8,469,635.205744

8.717103 0.2041 8,538,620.403429

8.720503 0.204 8,706,667.170638

8.723905 0.2041 8,634,237.447967

8.727307 0.2041 8,448,745.127808

8.730707 0.204 8,490,945.350640

8.734108 0.2041 8,648,582.325656

8.737510 0.2041 8,868,232.000006

8.740910 0.204 8,939,227.291266

8.744312 0.2041 8,752,354.064122

8.747713 0.2041 8,559,394.997368

8.751113 0.204 8,550,616.505455

8.754515 0.2041 8,352,004.182266

8.757917 0.2041 8,303,846.221419

8.761317 0.204 8,456,098.157404

8.764718 0.2041 8,430,879.906926

8.768120 0.2041 8,466,687.414245

8.771520 0.204 8,666,631.340723

8.774922 0.2041 8,871,028.050722

8.778323 0.2041 8,676,427.335755

8.781723 0.204 8,514,216.342544

8.785125 0.2041 8,620,836.825356

8.788527 0.2041 8,579,392.549165

8.791927 0.204 8,641,756.213554

8.795328 0.2041 8,802,916.451233

8.798730 0.2041 8,707,882.488195

8.802130 0.204 8,484,018.164647

8.805532 0.2041 8,504,966.482388

8.808933 0.2041 8,891,004.902264

8.812333 0.204 8,763,450.847048

8.815735 0.2041 8,427,645.778260

8.819137 0.2041 8,697,277.975788

8.822537 0.204 8,767,642.275567

8.825938 0.2041 8,532,088.582484

8.829340 0.2041 8,618,621.068739

8.832740 0.204 8,738,890.270496

8.836142 0.2041 8,555,183.443429

8.839543 0.2041 8,566,758.628199

8.842943 0.204 8,567,136.000104

8.846345 0.2041 8,521,126.815823

8.849747 0.2041 8,852,354.611369

8.853147 0.204 8,926,994.956725

8.856548 0.2041 8,708,906.085600

8.859950 0.2041 8,767,523.949820

8.863350 0.204 8,790,729.654373

8.866752 0.2041 8,615,096.112308

8.870153 0.2041 8,453,243.361078

8.873553 0.204 8,460,486.761323

8.876955 0.2041 8,642,925.240637

8.880357 0.2041 8,579,570.920200

8.883757 0.204 8,575,326.144444

8.887158 0.2041 8,551,728.084628

8.890560 0.2041 8,278,915.792952

8.893960 0.204 8,369,800.338626

8.897362 0.2041 8,481,312.157238

8.900763 0.2041 8,667,934.651443

8.904163 0.204 8,781,720.624906

8.907565 0.2041 8,505,955.635492

8.910967 0.2041 8,516,302.953343

8.914367 0.204 8,540,128.106037

8.917768 0.2041 8,690,242.244286

8.921170 0.2041 8,879,723.460963

8.924570 0.204 8,701,418.797273

8.927972 0.2041 8,702,812.264494

8.931373 0.2041 8,720,668.849665

8.934773 0.204 8,596,018.978603

8.938175 0.2041 8,686,645.714088

8.941577 0.2041 8,789,359.494951

8.944977 0.204 8,851,779.708014

8.948378 0.2041 8,918,544.500708

8.951780 0.2041 8,839,208.038195

8.955180 0.204 8,863,562.613234

8.958582 0.2041 8,846,762.553683

8.961983 0.2041 8,940,882.686905

8.965383 0.204 8,996,763.971261

8.968785 0.2041 8,789,427.770086

8.972187 0.2041 8,855,759.501755

8.975587 0.204 8,861,853.219822

8.978988 0.2041 8,886,877.917336

8.982390 0.2041 8,938,367.413289

8.985790 0.204 8,800,139.517495

8.989192 0.2041 8,814,190.007818

8.992593 0.2041 8,918,434.928653

8.995993 0.204 9,059,664.330318

8.999395 0.2041 8,861,897.874481

9.002797 0.2041 8,829,780.909765

9.006198 0.2041 9,010,915.882199

9.009598 0.204 9,153,863.077724

9.013000 0.2041 9,326,953.637393

9.016402 0.2041 9,750,195.561151

9.019802 0.204 10,925,355.155327

9.023203 0.2041 12,389,697.967651

9.026605 0.2041 14,631,630.990798

9.030005 0.204 18,267,041.249908

9.033407 0.2041 23,760,192.172754

9.036808 0.2041 31,581,260.627988

9.040208 0.204 42,086,388.746043

9.043610 0.2041 55,814,277.358833

9.047012 0.2041 75,202,508.205664

9.050412 0.204 100,642,802.874854

9.053813 0.2041 130,463,014.719426

9.057215 0.2041 165,957,286.340429

9.060615 0.204 206,997,527.403472

9.064017 0.2041 254,351,574.376544

9.067418 0.2041 305,409,293.093598

9.070818 0.204 363,405,183.296190

9.074220 0.2041 425,581,803.692868

9.077622 0.2041 484,536,682.080151

9.081022 0.204 549,604,822.577353

9.084423 0.2041 616,779,111.464725

9.087825 0.2041 674,085,612.661067

9.091225 0.204 730,373,849.327217

9.094627 0.2041 794,774,614.898305

9.098028 0.2041 857,197,262.973120

9.101428 0.204 910,782,162.261530

9.104830 0.2041 950,994,758.410043

9.108232 0.2041 990,747,865.545456

9.111632 0.204 1,026,031,292.629760

9.115033 0.2041 1,027,531,999.339830

9.118435 0.2041 998,833,168.411932

9.121835 0.204 938,111,934.110628

9.125237 0.2041 835,988,577.232971

9.128638 0.2041 698,884,087.003302

9.132038 0.204 546,938,682.188856

9.135440 0.2041 402,259,695.535608

9.138842 0.2041 274,511,816.444827

9.142242 0.204 177,045,783.264343

9.145643 0.2041 114,474,533.654208

9.149045 0.2041 74,739,442.017711

9.152445 0.204 51,454,884.638545

9.155847 0.2041 39,374,069.957188

9.159248 0.2041 32,131,698.167741

9.162648 0.204 27,820,820.332083

9.166050 0.2041 24,808,458.651943

9.169452 0.2041 23,082,349.058618

9.172852 0.204 21,991,449.330447

9.176253 0.2041 20,790,737.246706

9.179655 0.2041 20,164,151.816314

9.183055 0.204 19,683,186.969920

9.186457 0.2041 19,074,706.725607

9.189858 0.2041 18,498,299.745020

9.193258 0.204 17,984,675.402330

9.196660 0.2041 17,628,367.009801

9.200062 0.2041 17,235,477.638184

9.203462 0.204 16,755,306.855375

9.206863 0.2041 16,180,922.023459

9.210265 0.2041 16,026,878.205803

9.213665 0.204 16,090,486.800829

9.217067 0.2041 15,700,912.385072

9.220468 0.2041 15,550,014.471811

9.223868 0.204 15,324,239.409197

9.227270 0.2041 14,713,279.564183

9.230672 0.2041 14,575,883.666558

9.234072 0.204 14,336,068.300254

9.237473 0.2041 14,016,893.272347

9.240875 0.2041 14,101,152.091111

9.244275 0.204 13,914,751.840727

9.247677 0.2041 13,564,833.144144

9.251078 0.2041 13,532,857.953671

9.254478 0.204 13,488,613.232680

9.257880 0.2041 13,517,599.216937

9.261282 0.2041 13,491,202.546413

9.264682 0.204 13,220,734.653352

9.268083 0.2041 13,202,648.362058

9.271485 0.2041 12,930,565.368637

9.274885 0.204 12,629,143.944260

9.278287 0.2041 12,762,789.897078

9.281688 0.2041 12,431,112.099280

9.285088 0.204 12,144,750.971616

9.288490 0.2041 12,476,829.498205

9.291892 0.2041 12,429,535.286671

9.295292 0.204 12,250,086.214233

9.298693 0.2041 12,454,553.097390

9.302095 0.2041 12,389,622.636817

9.305495 0.204 12,282,721.194268

9.308897 0.2041 12,192,847.192637

9.312298 0.2041 12,053,736.170393

9.315698 0.204 12,149,408.975368

9.319100 0.2041 12,171,626.098545

9.322502 0.2041 12,084,102.799006

9.325902 0.204 12,032,407.312561

9.329303 0.2041 12,229,924.408878

9.332705 0.2041 12,077,041.110692

9.336105 0.204 11,768,052.091282

9.339507 0.2041 11,668,540.016842

9.342908 0.2041 11,538,688.247077

9.346308 0.204 11,671,139.954771

9.349710 0.2041 11,591,616.713478

9.353112 0.2041 11,452,615.816096

9.356513 0.2041 11,371,074.498472

9.359913 0.204 11,221,118.682076

9.363315 0.2041 11,146,790.201073

9.366717 0.2041 11,200,067.758063

9.370117 0.204 11,247,682.539465

9.373518 0.2041 11,263,373.001312

9.376920 0.2041 11,395,756.907540

9.380320 0.204 11,220,175.999455

9.383722 0.2041 11,235,669.010290

9.387123 0.2041 11,422,078.416168

9.390523 0.204 11,148,330.652991

9.393925 0.2041 11,001,797.102882

9.397327 0.2041 10,918,185.574446

9.400727 0.204 10,817,165.049467

9.404128 0.2041 10,984,363.951765

9.407530 0.2041 11,020,958.122344

9.410930 0.204 10,760,094.035056

9.414332 0.2041 10,823,830.250577

9.417733 0.2041 11,000,987.390649

9.421133 0.204 10,682,877.844438

9.424535 0.2041 10,468,262.713719

9.427937 0.2041 10,538,915.494152

9.431337 0.204 10,683,959.627234

9.434738 0.2041 10,607,292.047535

9.438140 0.2041 10,547,442.341115

9.441540 0.204 10,742,204.236279

9.444942 0.2041 10,717,558.465809

9.448343 0.2041 10,675,432.359843

9.451743 0.204 10,613,915.065364

9.455145 0.2041 10,692,081.647549

9.458547 0.2041 10,730,083.571550

9.461947 0.204 10,544,484.978631

9.465348 0.2041 10,450,386.109897

9.468750 0.2041 10,346,051.608812

9.472150 0.204 10,285,738.142852

9.475552 0.2041 10,334,836.780985

9.478953 0.2041 10,379,009.955465

9.482353 0.204 10,400,421.965768

9.485755 0.2041 10,497,633.033138

9.489157 0.2041 10,576,950.782516

9.492557 0.204 10,592,696.423085

9.495958 0.2041 10,427,916.509670

9.499360 0.2041 10,248,632.523629

9.502760 0.204 10,176,565.778955

9.506162 0.2041 10,199,204.866712

9.509563 0.2041 10,214,762.553828

9.512963 0.204 10,159,054.509781

9.516365 0.2041 10,231,447.747972

9.519767 0.2041 10,310,949.425309

9.523167 0.204 10,239,988.997153

9.526568 0.2041 10,223,877.938508

9.529970 0.2041 10,187,378.320888

9.533370 0.204 9,972,104.650975

9.536772 0.2041 9,917,645.794830

9.540173 0.2041 9,951,165.006254

9.543573 0.204 9,981,234.058548

9.546975 0.2041 10,058,189.215383

9.550377 0.2041 9,941,131.911939

9.553777 0.204 9,842,082.827743

9.557178 0.2041 10,085,150.664214

9.560580 0.2041 10,258,640.446776

9.563980 0.204 10,055,221.487678

9.567382 0.2041 9,906,148.600005

9.570783 0.2041 10,035,100.810344

9.574183 0.204 9,970,889.733744

9.577585 0.2041 10,000,267.472105

9.580987 0.2041 10,296,230.477284

9.584387 0.204 10,181,585.134688

9.587788 0.2041 10,105,331.001832

9.591190 0.2041 10,218,729.655859

9.594590 0.204 10,157,083.568382

9.597992 0.2041 10,178,387.415071

9.601393 0.2041 10,231,682.602660

9.604793 0.204 10,381,601.376109

9.608195 0.2041 10,508,105.715604

9.611597 0.2041 10,204,598.543863

9.614997 0.204 9,914,951.367343

9.618398 0.2041 10,191,693.157842

9.621800 0.2041 10,411,414.058839

9.625200 0.204 10,235,016.367108

9.628602 0.2041 9,995,536.641074

9.632003 0.2041 9,877,782.145116

9.635403 0.204 9,969,720.477839

9.638805 0.2041 9,975,316.266790

9.642207 0.2041 9,940,519.225897

9.645607 0.204 9,945,597.384476

9.649008 0.2041 9,783,892.158466

9.652410 0.2041 9,739,397.766462

9.655810 0.204 9,857,167.247607

9.659212 0.2041 9,748,347.478074

9.662613 0.2041 9,781,561.218347

9.666013 0.204 9,940,109.855210

9.669415 0.2041 9,937,029.963745

9.672817 0.2041 9,929,632.008188

9.676217 0.204 9,738,585.260175

9.679618 0.2041 9,592,537.861290

9.683020 0.2041 9,998,576.055579

9.686420 0.204 10,098,807.405131

9.689822 0.2041 9,798,503.219822

9.693223 0.2041 9,942,697.213672

9.696625 0.2041 9,830,188.493300

9.700025 0.204 9,657,506.044243

9.703427 0.2041 9,779,853.606849

9.706828 0.2041 9,761,316.802735

9.710228 0.204 9,800,828.047040

9.713630 0.2041 9,786,831.035659

9.717032 0.2041 9,816,607.513496

9.720432 0.204 9,846,136.781777

9.723833 0.2041 9,698,083.893898

9.727235 0.2041 9,733,296.966467

9.730635 0.204 9,758,723.671774

9.734037 0.2041 9,569,804.197462

9.737438 0.2041 9,608,605.631762

9.740838 0.204 9,885,437.752575

9.744240 0.2041 9,662,320.337636

9.747642 0.2041 9,491,222.026304

9.751042 0.204 9,710,474.756504

9.754443 0.2041 9,541,354.813893

9.757845 0.2041 9,524,531.246065

9.761245 0.204 9,760,235.405212

9.764647 0.2041 9,867,151.627160

9.768048 0.2041 9,854,411.766922

9.771448 0.204 9,670,813.435850

9.774850 0.2041 9,776,490.062552

9.778252 0.2041 9,990,979.581174

9.781652 0.204 9,925,667.077337

9.785053 0.2041 9,948,227.003675

9.788455 0.2041 10,071,390.800213

9.791855 0.204 9,919,097.677487

9.795257 0.2041 9,659,962.244951

9.798658 0.2041 9,789,330.446189

9.802058 0.204 10,003,648.336763

9.805460 0.2041 9,845,607.856590

9.808862 0.2041 9,477,742.327837

9.812262 0.204 9,794,081.802108

9.815663 0.2041 10,159,563.021004

9.819065 0.2041 9,676,123.598465

9.822465 0.204 9,619,475.722124

9.825867 0.2041 9,719,198.679378

9.829268 0.2041 9,617,038.761022

9.832668 0.204 9,686,419.816290

9.836070 0.2041 9,693,407.249388

9.839472 0.2041 9,697,864.659377

9.842872 0.204 9,690,964.154038

9.846273 0.2041 9,609,966.000510

9.849675 0.2041 9,606,414.105674

9.853075 0.204 9,646,286.985743

9.856477 0.2041 9,627,814.906669

9.859878 0.2041 9,703,400.346728

9.863278 0.204 9,670,180.205011

9.866680 0.2041 9,690,074.444416

9.870082 0.2041 9,626,444.257126

9.873482 0.204 9,467,606.786080

9.876883 0.2041 9,720,012.839562

9.880285 0.2041 9,790,686.030119

9.883685 0.204 9,540,933.691619

9.887087 0.2041 9,578,561.171751

9.890488 0.2041 9,524,119.573274

9.893888 0.204 9,294,383.046129

9.897290 0.2041 9,546,995.894183

9.900692 0.2041 9,788,456.470739

9.904092 0.204 9,531,272.751040

9.907493 0.2041 9,438,517.072448

9.910895 0.2041 9,638,217.187571

9.914295 0.204 9,809,936.624304

9.917697 0.2041 9,714,137.826134

9.921098 0.2041 9,451,904.351974

9.924498 0.204 9,415,535.066137

9.927900 0.2041 9,371,076.121026

9.931302 0.2041 9,196,110.196951

9.934702 0.204 9,238,734.843050

9.938103 0.2041 9,389,771.401543

9.941505 0.2041 9,393,560.082424

9.944905 0.204 9,325,077.694879

9.948307 0.2041 9,406,873.412176

9.951708 0.2041 9,427,736.695445

9.955108 0.204 9,397,449.332361

9.958510 0.2041 9,573,546.410504

9.961912 0.2041 9,424,084.516798

9.965312 0.204 9,324,440.492408

9.968713 0.2041 9,451,859.736705

9.972115 0.2041 9,304,261.126453

9.975515 0.204 9,323,641.432111

9.978917 0.2041 9,354,173.636912

9.982318 0.2041 9,352,323.081465

9.985718 0.204 9,520,483.390975

9.989120 0.2041 9,467,295.698441

9.992522 0.2041 9,382,720.311034

9.995922 0.204 9,306,606.704539

9.999323 0.2041 9,312,821.238720

10.002725 0.2041 9,274,116.003775

10.006125 0.204 9,092,640.770238

10.009527 0.2041 9,292,087.454807

10.012928 0.2041 9,473,396.325962

10.016328 0.204 9,236,027.599716

10.019730 0.2041 9,153,665.804253

10.023132 0.2041 9,282,709.235408

10.026532 0.204 9,173,139.854959

10.029933 0.2041 9,231,759.623973

10.033335 0.2041 9,410,956.932610

10.036735 0.204 9,234,841.684924

10.040137 0.2041 9,192,482.664764

10.043538 0.2041 9,338,835.779336

10.046940 0.2041 9,311,026.916607

10.050340 0.204 9,254,584.629101

10.053742 0.2041 9,295,542.205419

10.057143 0.2041 9,477,016.599111

10.060543 0.204 9,411,863.179703

10.063945 0.2041 9,365,787.457799

10.067347 0.2041 9,464,573.706024

10.070747 0.204 9,456,502.732424

10.074148 0.2041 9,407,559.066906

10.077550 0.2041 9,380,055.871819

10.080950 0.204 9,359,988.742977

10.084352 0.2041 9,298,939.884589

10.087753 0.2041 9,286,947.644751

10.091153 0.204 9,208,652.233386

10.094555 0.2041 9,120,543.547825

10.097957 0.2041 8,995,118.879569

10.101357 0.204 9,028,926.483674

10.104758 0.2041 9,040,784.851225

10.108160 0.2041 9,005,214.568910

10.111560 0.204 9,196,483.173158

10.114962 0.2041 9,258,399.647672

10.118363 0.2041 9,206,252.301594

10.121763 0.204 9,293,129.704915

10.125165 0.2041 9,235,093.121807

10.128567 0.2041 9,280,253.076053

10.131967 0.204 9,283,273.050887

10.135368 0.2041 9,146,129.449870

10.138770 0.2041 9,227,036.202553

10.142170 0.204 9,298,880.834865

10.145572 0.2041 9,224,272.728365

10.148973 0.2041 8,953,241.536630

10.152373 0.204 9,010,169.887115

10.155775 0.2041 9,227,389.837487

10.159177 0.2041 9,190,240.221087

10.162577 0.204 9,176,077.797212

10.165978 0.2041 9,120,508.630508

10.169380 0.2041 9,040,528.838264

10.172780 0.204 9,022,217.239587

10.176182 0.2041 9,152,618.010729

10.179583 0.2041 9,132,175.775215

10.182983 0.204 9,064,996.811616

10.186385 0.2041 9,264,646.230320

10.189787 0.2041 9,251,185.703226

10.193187 0.204 8,973,304.435391

10.196588 0.2041 8,887,937.717756

10.199990 0.2041 8,815,064.787214

10.203390 0.204 8,582,636.241259

10.206792 0.2041 8,722,882.664060

10.210193 0.2041 8,915,052.761973

10.213593 0.204 8,720,540.295567

10.216995 0.2041 8,564,709.856876

10.220397 0.2041 8,786,844.279456

10.223797 0.204 8,819,623.353838

10.227198 0.2041 8,474,127.435534

10.230600 0.2041 8,540,210.598275

10.234000 0.204 8,873,211.105707

10.237402 0.2041 8,880,205.860206

10.240803 0.2041 9,001,964.261228

10.244203 0.204 9,310,842.434254

10.247605 0.2041 9,426,385.437010

10.251007 0.2041 9,717,229.246784

10.254407 0.204 9,952,710.592293

10.257808 0.2041 10,061,805.302593

10.261210 0.2041 10,390,269.507062

10.264610 0.204 10,524,726.022874

10.268012 0.2041 10,304,700.150306

10.271413 0.2041 10,373,514.509797

10.274813 0.204 10,529,146.086422

10.278215 0.2041 10,314,921.596366

10.281617 0.2041 10,292,783.148745

10.285017 0.204 10,254,054.148948

10.288418 0.2041 9,900,990.239551

10.291820 0.2041 9,603,905.125076

10.295220 0.204 9,327,821.072275

10.298622 0.2041 8,931,108.734965

10.302023 0.2041 8,965,571.251765

10.305423 0.204 8,944,482.130034

10.308825 0.2041 8,706,221.860124

10.312227 0.2041 8,720,629.393566

10.315627 0.204 8,501,584.556493

10.319028 0.2041 8,431,946.428431

10.322430 0.2041 8,536,341.603241

10.325830 0.204 8,568,949.657533

10.329232 0.2041 8,470,572.988015

10.332633 0.2041 8,300,834.590654

10.336033 0.204 8,399,628.754894

10.339435 0.2041 8,430,667.474039

10.342837 0.2041 8,444,358.595489

10.346237 0.204 8,477,851.590958

10.349638 0.2041 8,489,559.388827

10.353040 0.2041 8,564,217.737580

10.356440 0.204 8,334,464.598082

10.359842 0.2041 8,243,731.683878

10.363243 0.2041 8,382,824.727811

10.366643 0.204 8,319,918.719385

10.370045 0.2041 8,339,382.664369

10.373447 0.2041 8,359,362.437329

10.376847 0.204 8,186,143.621822

10.380248 0.2041 8,175,156.242112

10.383650 0.2041 8,339,429.910989

10.387052 0.2041 8,272,889.558150

10.390452 0.204 8,202,964.804529

10.393853 0.2041 8,450,039.941009

10.397255 0.2041 8,533,888.321087

10.400655 0.204 8,569,134.724134

10.404057 0.2041 8,525,129.740186

10.407458 0.2041 8,372,966.405884

10.410858 0.204 8,455,345.160884

10.414260 0.2041 8,397,933.476158

10.417662 0.2041 8,374,339.838494

10.421062 0.204 8,370,089.513075

10.424463 0.2041 8,231,880.910916

10.427865 0.2041 8,324,862.734917

10.431265 0.204 8,396,387.205789

10.434667 0.2041 8,229,844.018716

10.438068 0.2041 8,388,344.853195

10.441468 0.204 8,459,205.328949

10.444870 0.2041 8,499,845.713629

10.448272 0.2041 8,733,475.662219

10.451672 0.204 8,458,879.114958

10.455073 0.2041 8,344,831.001805

10.458475 0.2041 8,337,425.873482

10.461875 0.204 8,050,499.872495

10.465277 0.2041 8,176,382.358670

10.468678 0.2041 8,413,611.727773

10.472078 0.204 8,420,960.795935

10.475480 0.2041 8,361,051.143146

10.478882 0.2041 8,285,765.365131

10.482282 0.204 8,438,176.370527

10.485683 0.2041 8,523,184.504559

10.489085 0.2041 8,402,685.497093

10.492485 0.204 8,431,560.442067

10.495887 0.2041 8,381,554.015799

10.499288 0.2041 8,256,657.050008

10.502688 0.204 8,309,751.704485

10.506090 0.2041 8,377,713.171397

10.509492 0.2041 8,427,562.645516

10.512892 0.204 8,422,662.409467

10.516293 0.2041 8,517,883.418100

10.519695 0.2041 8,800,477.301247

10.523095 0.204 8,977,028.116510

10.526497 0.2041 9,189,738.422868

10.529898 0.2041 9,467,723.328460

10.533298 0.204 9,713,547.269886

10.536700 0.2041 10,120,894.028809

10.540102 0.2041 10,459,519.225582

10.543502 0.204 10,746,070.021524

10.546903 0.2041 11,415,604.847875

10.550305 0.2041 11,970,020.549430

10.553705 0.204 12,213,561.376244

10.557107 0.2041 12,374,063.035373

10.560508 0.2041 12,367,227.474321

10.563908 0.204 12,284,071.910680

10.567310 0.2041 12,117,613.476519

10.570712 0.2041 11,824,842.885191

10.574112 0.204 11,523,229.625440

10.577513 0.2041 11,126,707.525460

10.580915 0.2041 10,628,245.945437

10.584315 0.204 10,254,592.183175

10.587717 0.2041 9,931,672.257428

10.591118 0.2041 9,688,914.223589

10.594518 0.204 9,278,087.243563

10.597920 0.2041 8,817,000.223165

10.601322 0.2041 8,707,723.047240

10.604722 0.204 8,655,150.267150

10.608123 0.2041 8,681,528.919628

10.611525 0.2041 8,580,331.591923

10.614925 0.204 8,365,150.350852

10.618327 0.2041 8,582,679.362116

10.621728 0.2041 8,625,503.149380

10.625128 0.204 8,592,028.331256

10.628530 0.2041 8,632,509.927960

10.631932 0.2041 8,432,920.855754

10.635332 0.204 8,293,559.544959

10.638733 0.2041 8,243,640.382634

10.642135 0.2041 8,299,438.607329

10.645535 0.204 8,294,059.423851

10.648937 0.2041 8,393,788.423501

10.652338 0.2041 8,269,585.600420

10.655738 0.204 8,223,591.524000

10.659140 0.2041 8,419,936.507750

10.662542 0.2041 8,431,873.994867

10.665942 0.204 8,425,918.423904

10.669343 0.2041 8,328,156.828679

10.672745 0.2041 8,344,625.922637

10.676145 0.204 8,281,491.467050

10.679547 0.2041 8,151,339.132808

10.682948 0.2041 8,243,775.083799

10.686348 0.204 8,494,246.877505

10.689750 0.2041 8,443,289.284142

10.693152 0.2041 8,412,145.216142

10.696552 0.204 8,500,831.344455

10.699953 0.2041 8,331,248.285551

10.703355 0.2041 8,265,280.982746

10.706755 0.204 8,197,476.946277

10.710157 0.2041 8,246,976.332207

10.713558 0.2041 8,336,546.729035

10.716958 0.204 8,248,435.774568

10.720360 0.2041 8,252,202.583157

10.723762 0.2041 8,402,992.857933

10.727162 0.204 8,503,056.416106

10.730563 0.2041 8,489,306.700419

10.733965 0.2041 8,305,468.736884

10.737367 0.2041 8,150,351.298689

10.740767 0.204 8,351,469.478156

10.744168 0.2041 8,453,628.235042

10.747570 0.2041 8,299,192.218823

10.750970 0.204 8,321,084.370040

10.754372 0.2041 8,381,242.209489

10.757773 0.2041 8,143,673.402597

10.761173 0.204 7,978,917.721146

10.764575 0.2041 8,144,139.729008

10.767977 0.2041 8,188,343.296884

10.771377 0.204 8,172,432.999017

10.774778 0.2041 8,274,600.427526

10.778180 0.2041 8,155,110.579917

10.781580 0.204 8,015,508.674088

10.784982 0.2041 8,070,031.105712

10.788383 0.2041 8,287,201.275123

10.791783 0.204 8,381,719.152096

10.795185 0.2041 8,221,115.058034

10.798587 0.2041 8,195,886.227828

10.801987 0.204 8,297,236.004762

10.805388 0.2041 8,244,286.590299

10.808790 0.2041 8,015,015.389713

10.812190 0.204 8,148,551.608385

10.815592 0.2041 8,356,590.297486

10.818993 0.2041 8,450,874.162686

10.822393 0.204 8,358,941.875597

10.825795 0.2041 8,181,578.501079

10.829197 0.2041 8,353,485.289638

10.832597 0.204 8,317,224.098052

10.835998 0.2041 8,437,936.435960

10.839400 0.2041 8,319,699.645609

10.842800 0.204 7,960,714.246196

10.846202 0.2041 8,143,737.813664

10.849603 0.2041 8,270,204.717748

10.853003 0.204 8,195,254.175348

10.856405 0.2041 8,100,940.598783

10.859807 0.2041 8,144,841.394284

10.863207 0.204 8,042,953.388579

10.866608 0.2041 7,987,240.543014

10.870010 0.2041 8,186,867.312501

10.873410 0.204 8,205,263.328691

10.876812 0.2041 8,274,122.853365

10.880213 0.2041 8,197,760.279012

10.883613 0.204 8,180,234.070586

10.887015 0.2041 8,424,543.893465

10.890417 0.2041 8,375,965.218722

10.893817 0.204 8,414,034.836394

10.897218 0.2041 8,404,858.128381

10.900620 0.2041 8,354,959.554176

10.904020 0.204 8,427,337.726310

10.907422 0.2041 8,212,115.259400

10.910823 0.2041 8,123,788.199343

10.914223 0.204 8,227,907.337179

10.917625 0.2041 8,251,564.001393

10.921027 0.2041 8,266,943.389448

10.924427 0.204 8,240,873.057828

10.927828 0.2041 8,253,630.248283

10.931230 0.2041 8,203,190.948048

10.934630 0.204 8,173,027.707774

10.938032 0.2041 8,442,874.576123

10.941433 0.2041 8,476,492.022604

10.944833 0.204 8,232,077.883385

10.948235 0.2041 8,174,989.183207

10.951637 0.2041 8,328,030.142284

10.955037 0.204 8,362,894.745255

10.958438 0.2041 8,249,676.720229

10.961840 0.2041 8,178,815.421657

10.965240 0.204 8,147,337.019632

10.968642 0.2041 8,324,108.186818

10.972043 0.2041 8,363,827.979340

10.975443 0.204 8,304,753.148942

10.978845 0.2041 8,476,196.629530

10.982247 0.2041 8,454,851.058017

10.985647 0.204 8,317,763.010755

10.989048 0.2041 8,171,093.564549

10.992450 0.2041 8,162,993.834501

10.995850 0.204 8,269,988.971560

10.999252 0.2041 8,024,176.612793

11.002653 0.2041 8,022,583.412185

11.006053 0.204 8,280,274.788274

11.009455 0.2041 8,327,683.538855

11.012857 0.2041 8,379,797.132452

11.016257 0.204 8,288,645.399246

11.019658 0.2041 8,077,146.119033

11.023060 0.2041 8,163,126.299426

11.026460 0.204 8,435,400.061694

11.029862 0.2041 8,338,395.022601

11.033263 0.2041 8,195,285.238712

11.036663 0.204 8,325,255.693811

11.040065 0.2041 8,446,190.237223

11.043467 0.2041 8,573,668.446177

11.046867 0.204 8,656,306.864049

11.050268 0.2041 8,595,936.381795

11.053670 0.2041 8,476,801.448974

11.057070 0.204 8,632,520.384219

11.060472 0.2041 8,669,167.207347

11.063873 0.2041 8,682,926.691744

11.067273 0.204 9,007,886.702244

11.070675 0.2041 9,084,570.931465

11.074077 0.2041 9,236,222.968760

11.077477 0.204 9,209,389.874815

11.080878 0.2041 9,133,375.799598

11.084280 0.2041 9,429,100.208582

11.087680 0.204 9,405,269.010194

11.091082 0.2041 9,416,194.329024

11.094483 0.2041 9,524,064.587891

11.097885 0.2041 9,397,021.481310

11.101285 0.204 9,377,367.590241

11.104687 0.2041 9,353,084.369243

11.108088 0.2041 9,441,779.532353

11.111488 0.204 9,367,479.723898

11.114890 0.2041 9,043,511.603479

11.118292 0.2041 9,025,353.878460

11.121692 0.204 8,780,215.711905

11.125093 0.2041 8,615,865.127186

11.128495 0.2041 8,702,113.102493

11.131895 0.204 8,494,699.962485

11.135297 0.2041 8,463,698.438289

11.138698 0.2041 8,412,448.438207

11.142098 0.204 8,189,427.532038

11.145500 0.2041 8,325,917.521950

11.148902 0.2041 8,461,462.688527

11.152302 0.204 8,292,260.102924

11.155703 0.2041 8,176,324.882606

11.159105 0.2041 8,380,689.238561

11.162505 0.204 8,658,208.663912

11.165907 0.2041 8,609,490.770316

11.169308 0.2041 8,323,526.544930

11.172708 0.204 8,119,112.440230

11.176110 0.2041 8,193,843.175828

11.179512 0.2041 8,452,487.116352

11.182912 0.204 8,420,363.141106

11.186313 0.2041 8,275,205.817654

11.189715 0.2041 8,545,335.097381

11.193115 0.204 8,487,489.600280

11.196517 0.2041 8,206,434.584525

11.199918 0.2041 8,324,608.024673

11.203318 0.204 8,233,352.568482

11.206720 0.2041 8,167,887.849251

11.210122 0.2041 8,324,678.716091

11.213522 0.204 8,240,302.878695

11.216923 0.2041 8,339,457.511470

11.220325 0.2041 8,460,713.273540

11.223725 0.204 8,259,299.068517

11.227127 0.2041 8,416,098.875578

11.230528 0.2041 8,578,565.812646

11.233928 0.204 8,407,116.252578

11.237330 0.2041 8,199,906.115627

11.240732 0.2041 8,296,109.328949

11.244132 0.204 8,466,969.694207

11.247533 0.2041 8,390,809.283919

11.250935 0.2041 8,311,234.516349

11.254335 0.204 8,184,569.443042

11.257737 0.2041 8,259,595.894914

11.261138 0.2041 8,185,452.048949

11.264538 0.204 8,236,070.382291

11.267940 0.2041 8,347,707.317496

11.271342 0.2041 8,174,207.572042

11.274742 0.204 8,217,045.463663

11.278143 0.2041 8,012,807.551677

11.281545 0.2041 7,988,154.764976

11.284945 0.204 8,249,799.749057

11.288347 0.2041 8,099,458.633567

11.291748 0.2041 8,051,423.445737

11.295148 0.204 8,195,956.629791

11.298550 0.2041 8,336,460.284862

11.301952 0.2041 8,392,097.498074

11.305352 0.204 8,276,207.270188

11.308753 0.2041 8,297,453.986660

11.312155 0.2041 8,249,949.197317

11.315555 0.204 8,195,575.282664

11.318957 0.2041 8,323,151.991606

11.322358 0.2041 8,249,492.072409

11.325758 0.204 8,082,432.760944

11.329160 0.2041 8,115,275.020147

11.332562 0.2041 8,225,433.945144

11.335962 0.204 8,280,060.950038

11.339363 0.2041 8,355,050.885569

11.342765 0.2041 8,319,056.585444

11.346165 0.204 8,259,569.290812

11.349567 0.2041 8,328,962.632727

11.352968 0.2041 8,299,712.307768

11.356368 0.204 8,247,390.807570

11.359770 0.2041 8,295,603.415411

11.363172 0.2041 8,313,654.785564

11.366572 0.204 8,301,189.264798

11.369973 0.2041 8,383,440.872553

11.373375 0.2041 8,357,795.903791

11.376775 0.204 8,357,219.891080

11.380177 0.2041 8,336,443.194400

11.383578 0.2041 8,215,536.934786

11.386978 0.204 8,271,463.002936

11.390380 0.2041 8,297,927.922178

11.393782 0.2041 8,264,039.517928

11.397182 0.204 8,277,928.706514

11.400583 0.2041 8,264,326.237003

11.403985 0.2041 8,201,626.070681

11.407385 0.204 8,359,602.283494

11.410787 0.2041 8,307,189.241102

11.414188 0.2041 8,208,987.242810

11.417588 0.204 8,468,974.001194

11.420990 0.2041 8,434,200.876582

11.424392 0.2041 8,394,368.115881

11.427792 0.204 8,177,911.627832

11.431193 0.2041 8,030,400.136557

11.434595 0.2041 8,270,453.284758

11.437997 0.2041 8,243,949.963050

11.441397 0.204 8,208,479.738116

11.444798 0.2041 8,311,958.456147

11.448200 0.2041 8,328,311.256175

11.451600 0.204 8,217,568.204073

11.455002 0.2041 8,154,486.284966

11.458403 0.2041 8,215,704.342203

11.461803 0.204 8,215,602.256381

11.465205 0.2041 8,219,916.357493

11.468607 0.2041 8,329,512.157794

11.472007 0.204 8,141,595.560179

11.475408 0.2041 8,030,122.700981

11.478810 0.2041 8,201,763.103050

11.482210 0.204 8,129,124.028292

11.485612 0.2041 8,111,717.946112

11.489013 0.2041 8,164,452.001502

11.492413 0.204 8,233,806.120529

11.495815 0.2041 8,355,108.779682

11.499217 0.2041 8,289,632.987298

11.502617 0.204 8,259,510.524356

11.506018 0.2041 8,450,710.950848

11.509420 0.2041 8,354,722.588917

11.512820 0.204 8,149,616.044515

11.516222 0.2041 8,410,929.405564

11.519623 0.2041 8,479,691.219598

11.523023 0.204 8,357,977.353913

11.526425 0.2041 8,436,680.995360

11.529827 0.2041 8,428,514.276777

11.533227 0.204 8,128,561.265561

11.536628 0.2041 7,990,773.278184

11.540030 0.2041 8,301,866.210855

11.543430 0.204 8,473,617.368308

11.546832 0.2041 8,424,346.631480

11.550233 0.2041 8,363,982.662156

11.553633 0.204 8,279,250.762419

11.557035 0.2041 8,298,400.543466

11.560437 0.2041 8,620,905.815320

11.563837 0.204 8,824,468.137859

11.567238 0.2041 8,736,806.628515

11.570640 0.2041 8,814,527.469447

11.574040 0.204 8,976,819.177810

11.577442 0.2041 9,024,847.699337

11.580843 0.2041 9,100,339.224414

11.584243 0.204 9,233,452.704813

11.587645 0.2041 9,274,020.545287

11.591047 0.2041 9,201,633.629494

11.594447 0.204 9,197,413.824352

11.597848 0.2041 9,227,356.663317

11.601250 0.2041 9,147,779.856826

11.604650 0.204 8,926,270.510831

11.608052 0.2041 8,778,416.791827

11.611453 0.2041 8,932,877.047259

11.614853 0.204 8,860,155.616330

11.618255 0.2041 8,468,516.395821

11.621657 0.2041 8,495,673.955451

11.625057 0.204 8,646,143.243386

11.628458 0.2041 8,537,577.678364

11.631860 0.2041 8,565,701.567566

11.635260 0.204 8,559,231.970794

11.638662 0.2041 8,458,130.507634

11.642063 0.2041 8,481,633.843983

11.645463 0.204 8,494,655.792285

11.648865 0.2041 8,572,740.918567

11.652267 0.2041 8,544,942.515699

11.655667 0.204 8,314,630.253330

11.659068 0.2041 8,378,401.573988

11.662470 0.2041 8,476,942.762405

11.665870 0.204 8,293,723.561091

11.669272 0.2041 8,367,138.116019

11.672673 0.2041 8,268,323.177694

11.676073 0.204 8,030,179.243448

11.679475 0.2041 8,272,959.219903

11.682877 0.2041 8,064,608.709557

11.686277 0.204 7,953,472.629627

11.689678 0.2041 8,276,750.527278

11.693080 0.2041 8,125,384.510913

11.696480 0.204 8,118,619.101198

11.699882 0.2041 8,109,158.174979

11.703283 0.2041 8,058,519.765095

11.706683 0.204 8,358,124.979398

11.710085 0.2041 8,263,939.838518

11.713487 0.2041 8,071,023.154503

11.716887 0.204 8,028,699.344804

11.720288 0.2041 7,962,904.867315

11.723690 0.2041 8,095,762.689508

11.727090 0.204 8,063,204.622244

11.730492 0.2041 8,045,318.732784

11.733893 0.2041 8,285,943.471217

11.737293 0.204 8,113,766.398653

11.740695 0.2041 8,013,649.763087

11.744097 0.2041 8,380,315.473446

11.747497 0.204 8,243,677.947521

11.750898 0.2041 8,075,239.191822

11.754300 0.2041 8,165,009.298859

11.757700 0.204 8,130,835.746562

11.761102 0.2041 8,196,360.656123

11.764503 0.2041 8,212,520.495286

11.767903 0.204 8,183,737.500898

11.771305 0.2041 8,162,652.252808

11.774707 0.2041 8,322,184.936320

11.778108 0.2041 8,346,161.542864

11.781508 0.204 8,111,826.376656

11.784910 0.2041 8,279,830.702738

11.788312 0.2041 8,393,210.319028

11.791712 0.204 8,124,588.287042

11.795113 0.2041 8,100,007.487156

11.798515 0.2041 8,287,530.723176

11.801915 0.204 8,173,733.064569

11.805317 0.2041 7,941,434.348026

11.808718 0.2041 8,107,384.336707

11.812118 0.204 8,327,670.599437

11.815520 0.2041 8,117,099.731568

11.818922 0.2041 8,122,295.832350

11.822322 0.204 8,342,800.594243

11.825723 0.2041 8,272,231.528087

11.829125 0.2041 8,117,434.366773

11.832525 0.204 8,111,365.393638

11.835927 0.2041 8,286,039.312611

11.839328 0.2041 8,283,944.757104

11.842728 0.204 8,273,697.420362

11.846130 0.2041 8,232,606.214373

11.849532 0.2041 8,159,288.006610

11.852932 0.204 8,233,913.373211

11.856333 0.2041 8,238,833.923274

11.859735 0.2041 8,220,920.695953

11.863135 0.204 8,183,799.724146

11.866537 0.2041 8,199,793.826443

11.869938 0.2041 8,349,261.439688

11.873338 0.204 8,299,712.802750

11.876740 0.2041 8,041,248.591089

11.880142 0.2041 8,102,119.074905

11.883542 0.204 8,135,463.876756

11.886943 0.2041 8,132,761.877977

11.890345 0.2041 8,144,544.842251

11.893745 0.204 8,085,739.853840

11.897147 0.2041 8,134,713.062857

11.900548 0.2041 8,171,594.404321

11.903948 0.204 8,152,946.517480

11.907350 0.2041 7,990,260.175953

11.910752 0.2041 8,169,680.454458

11.914152 0.204 8,324,436.850476

11.917553 0.2041 8,194,856.177768

11.920955 0.2041 8,101,667.491443

11.924355 0.204 8,114,831.963984

11.927757 0.2041 8,199,106.001664

11.931158 0.2041 8,181,316.053649

11.934558 0.204 8,371,912.772218

11.937960 0.2041 8,402,836.491574

11.941362 0.2041 8,206,338.724724

11.944762 0.204 8,396,266.242433

11.948163 0.2041 8,560,394.367876

11.951565 0.2041 8,536,497.944704

11.954965 0.204 8,507,546.020979

11.958367 0.2041 8,453,233.024774

11.961768 0.2041 8,599,777.981379

11.965168 0.204 8,574,355.595379

11.968570 0.2041 8,530,900.818786

11.971972 0.2041 8,635,967.993510

11.975372 0.204 8,610,942.340731

11.978773 0.2041 8,613,444.753501

11.982175 0.2041 8,549,886.522855

11.985575 0.204 8,473,347.376758

11.988977 0.2041 8,418,136.806664

11.992378 0.2041 8,367,155.710464

11.995778 0.204 8,284,246.028036

11.999180 0.2041 8,278,346.229561

12.002582 0.2041 8,271,363.672851

12.005982 0.204 8,161,289.169224

12.009383 0.2041 8,158,648.996340

12.012785 0.2041 8,020,565.611430

12.016185 0.204 8,028,875.345826

12.019587 0.2041 8,157,929.252870

12.022988 0.2041 8,188,680.435484

12.026388 0.204 8,302,935.980259

12.029790 0.2041 8,124,665.309852

12.033192 0.2041 8,149,686.436019

12.036592 0.204 8,543,385.753884

12.039993 0.2041 8,430,579.392679

12.043395 0.2041 8,235,302.678019

12.046795 0.204 8,252,267.157306

12.050197 0.2041 8,309,991.144716

12.053598 0.2041 8,497,162.882472

12.056998 0.204 8,752,513.318753

12.060400 0.2041 8,942,282.658543

12.063802 0.2041 8,856,696.643105

12.067202 0.204 9,113,917.883448

12.070603 0.2041 9,555,044.146231

12.074005 0.2041 9,793,745.667558

12.077405 0.204 9,853,476.785992

12.080807 0.2041 9,685,806.366995

12.084208 0.2041 9,822,290.471058

12.087608 0.204 9,839,145.110691

12.091010 0.2041 9,834,589.422079

12.094412 0.2041 9,821,750.329512

12.097812 0.204 9,426,833.074659

12.101213 0.2041 9,181,516.586256

12.104615 0.2041 8,998,058.151471

12.108015 0.204 8,901,653.673214

12.111417 0.2041 8,818,289.035642

12.114818 0.2041 8,618,375.744840

12.118218 0.204 8,646,582.561814

12.121620 0.2041 8,632,275.487992

12.125022 0.2041 8,492,373.263074

12.128423 0.2041 8,628,550.351617

12.131823 0.204 8,792,504.469334

12.135225 0.2041 8,641,950.800562

12.138627 0.2041 8,503,317.156805

12.142027 0.204 8,671,057.069748

12.145428 0.2041 8,725,205.550404

12.148830 0.2041 8,611,060.866631

12.152230 0.204 8,752,728.551295

12.155632 0.2041 8,789,161.491858

12.159033 0.2041 8,506,188.728922

12.162433 0.204 8,474,282.922656

12.165835 0.2041 8,708,502.092157

12.169237 0.2041 9,047,381.083239

12.172637 0.204 9,144,050.314321

12.176038 0.2041 9,272,397.587096

12.179440 0.2041 10,155,774.903491

12.182840 0.204 10,992,671.806827

12.186242 0.2041 11,939,867.140592

12.189643 0.2041 13,353,303.564160

12.193043 0.204 14,822,237.291578

12.196445 0.2041 16,640,729.036201

12.199847 0.2041 19,002,547.257865

12.203247 0.204 21,394,100.255629

12.206648 0.2041 22,750,576.060004

12.210050 0.2041 24,009,153.198598

12.213450 0.204 25,557,407.437991

12.216852 0.2041 26,250,071.464035

12.220253 0.2041 26,296,733.946056

12.223653 0.204 25,647,616.001044

12.227055 0.2041 24,522,346.784071

12.230457 0.2041 23,105,930.876918

12.233857 0.204 21,294,878.817712

12.237258 0.2041 19,338,335.456711

12.240660 0.2041 17,296,490.792932

12.244060 0.204 15,558,359.746999

12.247462 0.2041 14,090,126.217476

12.250863 0.2041 12,654,105.063891

12.254263 0.204 11,621,285.578875

12.257665 0.2041 10,897,398.083763

12.261067 0.2041 10,064,537.509896

12.264467 0.204 9,450,092.948698

12.267868 0.2041 9,264,238.885344

12.271270 0.2041 9,010,221.020539

12.274670 0.204 8,661,006.829886

12.278072 0.2041 8,436,780.581131

12.281473 0.2041 8,403,574.444090

12.284873 0.204 8,568,070.007858

12.288275 0.2041 8,555,129.309306

12.291677 0.2041 8,483,954.345264

12.295077 0.204 8,405,245.236957

12.298478 0.2041 8,349,141.919789

12.301880 0.2041 8,479,581.527965

12.305280 0.204 8,435,169.070344

12.308682 0.2041 8,399,583.481976

12.312083 0.2041 8,593,281.145367

12.315483 0.204 8,732,164.964611

12.318885 0.2041 8,650,465.544742

12.322287 0.2041 8,656,955.699980

12.325687 0.204 8,869,425.463995

12.329088 0.2041 8,971,839.010807

12.332490 0.2041 9,039,367.106117

12.335890 0.204 8,988,043.115779

12.339292 0.2041 8,883,334.252614

12.342693 0.2041 9,029,382.040757

12.346093 0.204 9,333,102.741487

12.349495 0.2041 9,386,648.350437

12.352897 0.2041 9,181,404.283793

12.356297 0.204 9,187,729.749416

12.359698 0.2041 9,154,780.709410

12.363100 0.2041 8,851,593.731346

12.366500 0.204 8,807,493.642975

12.369902 0.2041 8,795,491.484957

12.373303 0.2041 8,611,508.365209

12.376703 0.204 8,458,124.156905

12.380105 0.2041 8,228,443.101384

12.383507 0.2041 8,349,564.429686

12.386907 0.204 8,407,961.457500

12.390308 0.2041 8,247,075.215015

12.393710 0.2041 8,238,237.617038

12.397110 0.204 8,119,419.878318

12.400512 0.2041 8,090,099.762663

12.403913 0.2041 8,030,412.109884

12.407313 0.204 7,907,713.791424

12.410715 0.2041 7,943,678.954676

12.414117 0.2041 8,248,296.663902

12.417517 0.204 8,394,996.137752

12.420918 0.2041 8,161,618.207374

12.424320 0.2041 8,086,775.253120

12.427720 0.204 8,060,996.771931

12.431122 0.2041 7,897,019.943921

12.434523 0.2041 7,879,786.859840

12.437923 0.204 8,149,770.249453

12.441325 0.2041 8,162,675.043993

12.444727 0.2041 8,154,214.603483

12.448127 0.204 8,066,549.900218

12.451528 0.2041 7,915,676.733968

12.454930 0.2041 7,959,968.890366

12.458330 0.204 7,891,659.648565

12.461732 0.2041 8,136,802.478212

12.465133 0.2041 8,195,196.821607

12.468533 0.204 8,140,981.116942

12.471935 0.2041 8,264,571.354973

12.475337 0.2041 8,238,713.114562

12.478738 0.2041 8,214,630.841338

12.482138 0.204 8,200,500.031016

12.485540 0.2041 8,177,984.254434

12.488942 0.2041 8,185,735.917651

12.492342 0.204 8,184,272.594899

12.495743 0.2041 8,187,712.930636

12.499145 0.2041 8,454,919.990943

12.502545 0.204 8,260,027.259090

12.505947 0.2041 7,974,931.867698

12.509348 0.2041 8,280,071.191994

12.512748 0.204 8,020,727.840588

12.516150 0.2041 7,867,481.744063

12.519552 0.2041 8,351,511.921260

12.522952 0.204 8,435,108.909037

12.526353 0.2041 8,471,266.210036

12.529755 0.2041 8,593,205.891072

12.533155 0.204 8,357,900.573235

12.536557 0.2041 8,174,066.613445

12.539958 0.2041 8,293,314.504182

12.543358 0.204 8,393,416.629720

12.546760 0.2041 8,420,034.711913

12.550162 0.2041 8,400,811.564157

12.553562 0.204 8,313,126.903615

12.556963 0.2041 8,374,183.330944

12.560365 0.2041 8,317,343.982162

12.563765 0.204 8,188,427.902672

12.567167 0.2041 8,239,760.081159

12.570568 0.2041 8,336,379.581565

12.573968 0.204 8,245,254.820835

12.577370 0.2041 8,087,716.449053

12.580772 0.2041 8,278,588.123220

12.584172 0.204 8,177,032.643538

12.587573 0.2041 8,029,984.405705

12.590975 0.2041 8,137,571.809406

12.594375 0.204 7,979,505.566307

12.597777 0.2041 8,023,144.735914

12.601178 0.2041 8,079,111.235384

12.604578 0.204 8,079,284.271102

12.607980 0.2041 8,269,935.131532

12.611382 0.2041 8,269,885.552489

12.614782 0.204 8,178,761.836163

12.618183 0.2041 8,288,379.043234

12.621585 0.2041 8,382,428.992488

12.624985 0.204 8,233,167.597143

12.628387 0.2041 8,053,963.498331

12.631788 0.2041 8,143,722.232534

12.635188 0.204 8,307,531.611343

12.638590 0.2041 8,143,638.785014

12.641992 0.2041 8,137,057.479931

12.645392 0.204 8,109,781.734772

12.648793 0.2041 7,919,567.829888

12.652195 0.2041 8,019,275.129049

12.655595 0.204 8,011,184.716544

12.658997 0.2041 7,989,360.648163

12.662398 0.2041 8,146,119.044279

12.665798 0.204 8,090,006.316952

12.669200 0.2041 7,978,813.821711

12.672602 0.2041 8,044,901.997823

12.676002 0.204 7,994,158.802809

12.679403 0.2041 8,051,747.597759

12.682805 0.2041 8,061,865.437186

12.686205 0.204 7,822,265.309366

12.689607 0.2041 7,884,258.090164

12.693008 0.2041 8,182,184.288715

12.696408 0.204 8,411,650.995175

12.699810 0.2041 8,698,215.041939

12.703212 0.2041 9,120,053.517289

12.706612 0.204 9,913,512.192415

12.710013 0.2041 11,381,950.380512

12.713415 0.2041 13,209,793.559440

12.716815 0.204 15,781,937.917837

12.720217 0.2041 19,937,779.350632

12.723618 0.2041 25,505,934.714162

12.727018 0.204 31,807,487.349760

12.730420 0.2041 39,741,122.930218

12.733822 0.2041 49,631,978.737582

12.737222 0.204 60,764,189.684436

12.740623 0.2041 71,989,565.314442

12.744025 0.2041 83,817,772.691560

12.747425 0.204 95,101,781.522145

12.750827 0.2041 104,364,400.093669

12.754228 0.2041 112,284,813.884350

12.757628 0.204 115,117,878.896317

12.761030 0.2041 116,040,090.283947

12.764432 0.2041 114,152,989.631341

12.767832 0.204 106,222,972.056810

12.771233 0.2041 96,403,118.314074

12.774635 0.2041 84,284,584.907739

12.778035 0.204 71,410,934.852210

12.781437 0.2041 59,011,951.320966

12.784838 0.2041 47,609,863.252009

12.788238 0.204 38,311,497.057121

12.791640 0.2041 30,349,317.874384

12.795042 0.2041 24,191,678.629822

12.798442 0.204 19,745,853.820305

12.801843 0.2041 16,462,633.145862

12.805245 0.2041 14,331,465.476627

12.808645 0.204 12,605,035.667360

12.812047 0.2041 11,422,108.012743

12.815448 0.2041 10,704,063.550154

12.818848 0.204 10,156,648.138005

12.822250 0.2041 10,024,099.084080

12.825652 0.2041 9,848,681.916249

12.829052 0.204 9,733,918.399490

12.832453 0.2041 9,724,005.321425

12.835855 0.2041 9,603,885.466926

12.839255 0.204 9,593,340.405706

12.842657 0.2041 9,473,562.688401

12.846058 0.2041 9,343,115.953001

12.849460 0.2041 9,305,093.146345

12.852860 0.204 9,047,316.439385

12.856262 0.2041 9,037,240.824890

12.859663 0.2041 9,056,416.000389

12.863063 0.204 8,816,398.621205

12.866465 0.2041 8,746,665.632496

12.869867 0.2041 8,907,962.115699

12.873267 0.204 8,764,893.353933

12.876668 0.2041 8,528,933.364895

12.880070 0.2041 8,718,133.426583

12.883470 0.204 8,632,967.596527

12.886872 0.2041 8,366,073.394388

12.890273 0.2041 8,482,925.150239

12.893673 0.204 8,484,564.373817

12.897075 0.2041 8,431,147.721480

12.900477 0.2041 8,599,111.664469

12.903877 0.204 8,383,853.149661

12.907278 0.2041 8,369,113.332308

12.910680 0.2041 8,618,287.535664

12.914080 0.204 8,392,522.857088

12.917482 0.2041 8,284,403.230512

12.920883 0.2041 8,387,915.259368

12.924283 0.204 8,387,931.897992

12.927685 0.2041 8,371,038.298294

12.931087 0.2041 8,493,477.442882

12.934487 0.204 8,689,383.202244

12.937888 0.2041 8,626,295.397684

12.941290 0.2041 8,385,960.191673

12.944690 0.204 8,344,192.026531

12.948092 0.2041 8,524,792.007023

12.951493 0.2041 8,493,002.554907

12.954893 0.204 8,382,546.995748

12.958295 0.2041 8,559,058.846361

12.961697 0.2041 8,504,590.717323

12.965097 0.204 8,131,920.303809

12.968498 0.2041 8,026,082.555006

12.971900 0.2041 8,258,304.062441

12.975300 0.204 8,551,472.717154

12.978702 0.2041 8,515,199.112811

12.982103 0.2041 7,998,236.499950

12.985503 0.204 7,874,687.858629

12.988905 0.2041 8,195,139.992730

12.992307 0.2041 8,151,728.476190

12.995707 0.204 8,136,576.447463

12.999108 0.2041 8,118,344.580637

13.002510 0.2041 8,066,492.299988

13.005910 0.204 8,113,522.587785

13.009312 0.2041 8,135,439.632981

13.012713 0.2041 8,233,662.077255

13.016113 0.204 8,163,563.699481

13.019515 0.2041 8,211,710.704371

13.022917 0.2041 8,368,577.837208

13.026317 0.204 8,365,984.754749

13.029718 0.2041 8,263,937.016252

13.033120 0.2041 8,282,632.355093

13.036520 0.204 8,519,421.949194

13.039922 0.2041 8,441,021.197971

13.043323 0.2041 8,322,456.848546

13.046723 0.204 8,426,993.891929

13.050125 0.2041 8,478,908.504243

13.053527 0.2041 8,550,001.826852

13.056927 0.204 8,635,426.984607

13.060328 0.2041 8,585,621.162181

13.063730 0.2041 8,279,247.998329

13.067130 0.204 8,311,817.027574

13.070532 0.2041 8,419,466.559508

13.073933 0.2041 8,374,981.520276

13.077333 0.204 8,599,600.878412

13.080735 0.2041 8,429,370.795109

13.084137 0.2041 8,298,648.402949

13.087537 0.204 8,525,230.338201

13.090938 0.2041 8,639,298.821974

13.094340 0.2041 8,417,298.208125

13.097740 0.204 7,926,724.154543

13.101142 0.2041 7,980,847.337596

13.104543 0.2041 8,276,153.556992

13.107943 0.204 8,185,951.972228

13.111345 0.2041 8,124,766.593718

13.114747 0.2041 8,289,934.341467

13.118147 0.204 8,286,164.445557

13.121548 0.2041 8,208,182.138519

13.124950 0.2041 8,252,298.326060

13.128350 0.204 8,283,558.181523

13.131752 0.2041 8,288,322.617916

13.135153 0.2041 8,115,110.104542

13.138553 0.204 8,061,144.028822

13.141955 0.2041 8,294,563.114841

13.145357 0.2041 8,279,777.346136

13.148757 0.204 8,074,734.380583

13.152158 0.2041 8,235,789.966833

13.155560 0.2041 8,343,167.569896

13.158960 0.204 8,263,251.362374

13.162362 0.2041 8,111,062.666955

13.165763 0.2041 8,143,099.509457

13.169163 0.204 8,291,235.082106

13.172565 0.2041 8,081,587.491626

13.175967 0.2041 8,161,879.846005

13.179367 0.204 8,247,608.346515

13.182768 0.2041 8,179,651.970504

13.186170 0.2041 8,248,351.470162

13.189570 0.204 8,201,704.472541

13.192972 0.2041 8,298,056.623735

13.196373 0.2041 8,647,410.093712

13.199775 0.2041 8,582,823.894647

13.203175 0.204 8,328,517.516127

13.206577 0.2041 8,441,487.933807

13.209978 0.2041 8,239,025.589893

13.213378 0.204 8,125,660.403035

13.216780 0.2041 8,464,766.557670

13.220182 0.2041 8,275,724.560823

13.223582 0.204 7,949,294.812810

13.226983 0.2041 7,907,365.642944

13.230385 0.2041 8,044,492.377124

13.233785 0.204 8,335,953.146754

13.237187 0.2041 8,296,839.970093

13.240588 0.2041 8,252,207.954979

13.243988 0.204 8,209,183.420508

13.247390 0.2041 8,015,213.908633

13.250792 0.2041 7,979,391.347824

13.254192 0.204 8,150,955.019527

13.257593 0.2041 8,197,435.619227

13.260995 0.2041 8,080,926.418407

13.264395 0.204 8,122,585.806763

13.267797 0.2041 8,199,794.247146

13.271198 0.2041 7,951,703.103972

13.274598 0.204 7,861,368.226842

13.278000 0.2041 8,180,990.907639

13.281402 0.2041 8,162,020.148093

13.284802 0.204 8,090,818.669182

13.288203 0.2041 8,243,147.797281

13.291605 0.2041 8,130,679.382961

13.295005 0.204 7,952,221.473007

13.298407 0.2041 7,987,903.795468

13.301808 0.2041 8,109,245.500261

13.305208 0.204 8,139,882.588362

13.308610 0.2041 8,016,097.878611

13.312012 0.2041 8,046,108.207753

13.315412 0.204 8,138,872.017946

13.318813 0.2041 8,254,942.988435

13.322215 0.2041 8,100,248.829075

13.325615 0.204 8,037,950.651739

13.329017 0.2041 8,194,069.878174

13.332418 0.2041 7,837,532.760598

13.335818 0.204 7,706,979.813271

13.339220 0.2041 8,001,046.715977

13.342622 0.2041 8,084,681.896185

13.346022 0.204 8,087,949.060329

13.349423 0.2041 8,087,329.121976

13.352825 0.2041 7,890,058.265062

13.356225 0.204 7,885,867.537137

13.359627 0.2041 8,080,828.185989

13.363028 0.2041 8,115,574.358116

13.366428 0.204 8,031,260.460252

13.369830 0.2041 7,857,380.485078

13.373232 0.2041 7,898,575.785130

13.376632 0.204 8,251,689.483718

13.380033 0.2041 8,286,158.263440

13.383435 0.2041 8,514,851.910586

13.386835 0.204 8,869,696.627652

13.390237 0.2041 8,919,999.696565

13.393638 0.2041 9,541,485.735054

13.397038 0.204 10,183,681.662391

13.400440 0.2041 10,779,922.256843

13.403842 0.2041 11,664,242.038517

13.407242 0.204 12,556,450.461173

13.410643 0.2041 13,538,607.452080

13.414045 0.2041 14,558,216.896404

13.417445 0.204 15,550,367.248089

13.420847 0.2041 16,143,819.770641

13.424248 0.2041 16,593,380.519871

13.427648 0.204 16,586,307.935454

13.431050 0.2041 16,072,433.108598

13.434452 0.2041 15,766,127.414502

13.437852 0.204 15,480,923.118712

13.441253 0.2041 15,082,683.030150

13.444655 0.2041 14,186,046.441353

13.448055 0.204 13,339,359.977861

13.451457 0.2041 12,805,663.137149

13.454858 0.2041 11,943,690.476915

13.458258 0.204 11,262,766.258989

13.461660 0.2041 10,919,326.493080

13.465062 0.2041 10,622,275.893884

13.468462 0.204 10,433,511.914786

13.471863 0.2041 9,971,330.547052

13.475265 0.2041 9,438,897.492920

13.478665 0.204 9,226,270.745848

13.482067 0.2041 9,097,792.790485

13.485468 0.2041 8,904,657.035854

13.488868 0.204 8,798,600.580240

13.492270 0.2041 8,718,034.535824

13.495672 0.2041 8,569,581.163415

13.499072 0.204 8,433,627.498793

13.502473 0.2041 8,051,177.880673

13.505875 0.2041 8,126,504.287330

13.509275 0.204 8,433,560.950444

13.512677 0.2041 8,267,475.119012

13.516078 0.2041 8,066,921.698598

13.519478 0.204 8,088,792.666235

13.522880 0.2041 8,248,074.532323

13.526282 0.2041 8,094,371.743269

13.529682 0.204 8,003,819.934626

13.533083 0.2041 8,162,765.994782

13.536485 0.2041 8,296,670.025531

13.539887 0.2041 8,152,398.546527

13.543287 0.204 8,015,716.545346

13.546688 0.2041 8,282,900.978576

13.550090 0.2041 8,149,828.157603

13.553490 0.204 8,015,201.056412

13.556892 0.2041 8,087,534.841827

13.560293 0.2041 7,987,331.814833

13.563693 0.204 8,017,701.795799

13.567095 0.2041 7,981,189.349728

13.570497 0.2041 7,956,803.355290

13.573897 0.204 8,021,480.429357

13.577298 0.2041 8,064,857.399517

13.580700 0.2041 8,136,850.006353

13.584100 0.204 8,117,412.766793

13.587502 0.2041 8,019,446.408314

13.590903 0.2041 8,071,363.606759

13.594303 0.204 8,130,133.592637

13.597705 0.2041 8,100,261.204698

13.601107 0.2041 8,009,834.067461

13.604507 0.204 8,003,005.348050

13.607908 0.2041 8,014,835.138990

13.611310 0.2041 7,956,422.776870

13.614710 0.204 8,052,103.265245

13.618112 0.2041 8,033,526.741489

13.621513 0.2041 8,098,652.002570

13.624913 0.204 8,076,629.125056

13.628315 0.2041 8,070,423.013329

13.631717 0.2041 8,308,956.499032

13.635117 0.204 8,161,354.061415

13.638518 0.2041 8,137,887.820268

13.641920 0.2041 8,326,029.233419

13.645320 0.204 8,274,900.223898

13.648722 0.2041 8,242,056.679459

13.652123 0.2041 8,193,360.005159

13.655523 0.204 8,216,821.525536

13.658925 0.2041 8,293,831.626728

13.662327 0.2041 8,262,688.424308

13.665727 0.204 8,216,990.324991

13.669128 0.2041 8,306,274.166283

13.672530 0.2041 8,310,343.141088

13.675930 0.204 8,298,467.689743

13.679332 0.2041 8,617,095.706015

13.682733 0.2041 8,724,529.204876

13.686133 0.204 8,823,275.265959

13.689535 0.2041 9,090,807.615143

13.692937 0.2041 9,138,910.632851

13.696337 0.204 9,315,169.605884

13.699738 0.2041 9,522,829.546806

13.703140 0.2041 9,594,298.229628

13.706540 0.204 9,716,342.148093

13.709942 0.2041 9,843,018.181775

13.713343 0.2041 9,837,638.791243

13.716743 0.204 9,736,081.689421

13.720145 0.2041 9,458,329.005504

13.723547 0.2041 9,260,353.328780

13.726947 0.204 9,202,593.859501

13.730348 0.2041 9,047,343.219895

13.733750 0.2041 9,030,264.299680

13.737150 0.204 8,859,162.510195

13.740552 0.2041 8,601,365.199624

13.743953 0.2041 8,636,800.642463

13.747353 0.204 8,540,347.197658

13.750755 0.2041 8,280,727.066551

13.754157 0.2041 8,132,550.343922

13.757557 0.204 8,149,500.534624

13.760958 0.2041 8,188,744.050377

13.764360 0.2041 8,057,755.766474

13.767760 0.204 7,939,256.783912

13.771162 0.2041 7,935,480.408017

13.774563 0.2041 7,943,141.816885

13.777963 0.204 7,972,189.502225

13.781365 0.2041 8,022,939.169215

13.784767 0.2041 7,951,999.784356

13.788167 0.204 7,882,506.654310

13.791568 0.2041 7,926,263.590052

13.794970 0.2041 7,971,249.504037

13.798370 0.204 7,833,280.907075

13.801772 0.2041 7,844,929.465960

13.805173 0.2041 7,992,240.046623

13.808573 0.204 7,787,040.205201

13.811975 0.2041 7,745,536.241205

13.815377 0.2041 7,845,854.257093

13.818777 0.204 7,853,737.111786

13.822178 0.2041 8,132,459.044378

13.825580 0.2041 8,206,289.612012

13.828980 0.204 7,935,622.089835

13.832382 0.2041 7,849,254.202166

13.835783 0.2041 7,899,510.150321

13.839183 0.204 7,953,540.424719

13.842585 0.2041 7,928,706.411114

13.845987 0.2041 7,948,623.903841

13.849387 0.204 8,069,564.850306

13.852788 0.2041 7,958,800.370648

13.856190 0.2041 7,935,496.293738

13.859590 0.204 8,125,347.845131

13.862992 0.2041 8,077,402.570083

13.866393 0.2041 7,934,045.422302

13.869793 0.204 7,879,806.741335

13.873195 0.2041 7,843,394.359157

13.876597 0.2041 7,887,343.617794

13.879997 0.204 7,850,700.986344

13.883398 0.2041 7,969,666.194964

13.886800 0.2041 8,037,154.372202

13.890200 0.204 7,951,086.593037

13.893602 0.2041 7,967,265.384049

13.897003 0.2041 7,881,438.655487

13.900405 0.2041 8,032,495.509219

13.903805 0.204 8,176,881.396337

13.907207 0.2041 7,948,573.542570

13.910608 0.2041 7,846,921.961619

13.914008 0.204 7,952,814.702172

13.917410 0.2041 7,906,022.516637

13.920812 0.2041 8,004,051.725816

13.924212 0.204 8,050,785.027328

13.927613 0.2041 8,115,501.126002

13.931015 0.2041 8,203,418.206329

13.934415 0.204 8,044,532.004603

13.937817 0.2041 8,282,393.318843

13.941218 0.2041 8,241,097.957660

13.944618 0.204 7,974,382.839604

13.948020 0.2041 8,174,964.369743

13.951422 0.2041 8,213,654.507658

13.954822 0.204 8,149,326.394685

13.958223 0.2041 8,184,997.954723

13.961625 0.2041 8,059,586.352633

13.965025 0.204 7,956,860.878975

13.968427 0.2041 8,005,414.408378

13.971828 0.2041 7,899,232.188642

13.975228 0.204 7,912,835.169820

13.978630 0.2041 7,991,441.868985

13.982032 0.2041 7,957,764.682217

13.985432 0.204 7,969,868.192816

13.988833 0.2041 7,838,854.190214

13.992235 0.2041 7,875,700.507150

13.995635 0.204 7,964,857.419916

13.999037 0.2041 7,771,143.971717

14.002438 0.2041 7,670,788.827056

14.005838 0.204 7,771,477.509439

14.009240 0.2041 7,950,274.491255

14.012642 0.2041 8,105,037.358507

14.016042 0.204 8,216,795.359865

14.019443 0.2041 8,119,621.808682

14.022845 0.2041 7,752,886.264912

14.026245 0.204 7,892,595.119486

14.029647 0.2041 8,142,022.061600

14.033048 0.2041 8,083,901.916061

14.036448 0.204 8,070,262.600021

14.039850 0.2041 7,957,336.183361

14.043252 0.2041 7,884,911.518776

14.046652 0.204 7,851,302.553869

14.050053 0.2041 7,992,773.141384

14.053455 0.2041 8,003,280.764484

14.056855 0.204 7,842,893.534756

14.060257 0.2041 7,888,550.996262

14.063658 0.2041 8,073,796.167585

14.067058 0.204 8,181,884.646644

14.070460 0.2041 8,127,204.752370

14.073862 0.2041 8,044,577.326183

14.077262 0.204 7,848,740.439086

14.080663 0.2041 7,799,218.579128

14.084065 0.2041 7,854,529.505294

14.087465 0.204 7,855,890.804479

14.090867 0.2041 7,980,244.109454

14.094268 0.2041 7,961,058.385910

14.097668 0.204 7,983,590.375894

14.101070 0.2041 7,991,345.385169

14.104472 0.2041 7,684,958.488953

14.107872 0.204 7,876,080.856082

14.111273 0.2041 8,044,083.221426

14.114675 0.2041 7,883,961.750924

14.118075 0.204 7,815,843.731958

14.121477 0.2041 7,888,045.607258

14.124878 0.2041 8,074,191.576576

14.128278 0.204 7,836,129.371181

14.131680 0.2041 7,867,736.298774

14.135082 0.2041 8,069,088.381589

14.138482 0.204 7,965,406.060238

14.141883 0.2041 8,023,009.290693

14.145285 0.2041 8,193,472.023042

14.148685 0.204 8,296,332.949474

14.152087 0.2041 8,244,728.490009

14.155488 0.2041 8,303,962.057473

14.158888 0.204 8,294,842.297007

14.162290 0.2041 7,836,866.146255

14.165692 0.2041 7,749,243.038466

14.169092 0.204 7,991,227.493663

14.172493 0.2041 7,991,897.575704

14.175895 0.2041 8,095,188.755254

14.179295 0.204 7,953,808.915768

14.182697 0.2041 7,755,308.056527

14.186098 0.2041 7,962,071.813325

14.189498 0.204 7,983,489.414717

14.192900 0.2041 7,833,646.680751

14.196302 0.2041 7,820,605.703438

14.199702 0.204 7,848,651.868370

14.203103 0.2041 7,859,184.885970

14.206505 0.2041 7,816,406.532639

14.209905 0.204 7,759,351.772601

14.213307 0.2041 7,665,191.145014

14.216708 0.2041 7,774,516.209370

14.220108 0.204 7,834,487.131098

14.223510 0.2041 7,721,823.023563

14.226912 0.2041 7,897,039.318889

14.230312 0.204 7,968,129.648170

14.233713 0.2041 7,751,819.325069

14.237115 0.2041 7,824,631.728950

14.240517 0.2041 7,901,972.972368

14.243917 0.204 7,961,535.894857

14.247318 0.2041 8,101,922.094195

14.250720 0.2041 8,007,803.928858

14.254120 0.204 8,047,355.986193

14.257522 0.2041 8,031,735.744511

14.260923 0.2041 7,894,558.297475

14.264323 0.204 7,864,167.535211

14.267725 0.2041 7,880,472.090415

14.271127 0.2041 8,026,040.289570

14.274527 0.204 8,103,628.874235

14.277928 0.2041 7,974,707.791512

14.281330 0.2041 7,899,294.558859

14.284730 0.204 7,927,480.305180

14.288132 0.2041 8,009,328.022643

14.291533 0.2041 8,053,572.322987

14.294933 0.204 7,785,500.454977

14.298335 0.2041 7,760,701.061580

14.301737 0.2041 8,053,622.043382

14.305137 0.204 7,978,338.383210

14.308538 0.2041 7,876,871.295592

14.311940 0.2041 7,936,636.650076

14.315340 0.204 7,862,343.083090

14.318742 0.2041 7,758,775.490998

14.322143 0.2041 7,737,721.819517

14.325543 0.204 7,878,430.366114

14.328945 0.2041 7,959,242.332993

14.332347 0.2041 7,824,415.695779

14.335747 0.204 7,709,753.036726

14.339148 0.2041 7,589,357.375612

14.342550 0.2041 7,752,903.773456

14.345950 0.204 7,925,567.497056

14.349352 0.2041 7,807,683.097491

14.352753 0.2041 7,727,229.914368

14.356153 0.204 7,715,326.377962

14.359555 0.2041 7,831,934.546337

14.362957 0.2041 7,772,394.790721

14.366357 0.204 7,600,275.007365

14.369758 0.2041 7,623,516.654591

14.373160 0.2041 7,511,650.020289

14.376560 0.204 7,602,156.615320

14.379962 0.2041 7,891,918.794374

14.383363 0.2041 7,934,140.477752

14.386763 0.204 7,902,484.819061

14.390165 0.2041 7,872,878.102553

14.393567 0.2041 7,815,712.087799

14.396967 0.204 7,805,762.834026

14.400368 0.2041 7,947,822.070423

14.403770 0.2041 8,030,825.202511

14.407170 0.204 8,063,250.080225

14.410572 0.2041 8,095,158.617953

14.413973 0.2041 8,101,618.623229

14.417373 0.204 8,274,949.155619

14.420775 0.2041 8,345,076.180987

14.424177 0.2041 8,272,854.410753

14.427577 0.204 8,300,546.703852

14.430978 0.2041 8,326,614.336786

14.434380 0.2041 8,443,562.289235

14.437780 0.204 8,465,639.951992

14.441182 0.2041 8,414,514.348700

14.444583 0.2041 8,362,865.123676

14.447983 0.204 8,178,211.077528

14.451385 0.2041 8,185,563.608013

14.454787 0.2041 8,159,491.892845

14.458187 0.204 7,978,764.881894

14.461588 0.2041 7,923,770.682302

14.464990 0.2041 7,885,564.196319

14.468390 0.204 7,906,527.355326

14.471792 0.2041 7,988,143.021665

14.475193 0.2041 7,985,400.543112

14.478593 0.204 8,091,511.947478

14.481995 0.2041 8,193,504.840759

14.485397 0.2041 8,058,319.793271

14.488797 0.204 7,929,244.935803

14.492198 0.2041 8,010,898.630709

14.495600 0.2041 8,098,303.642020

14.499000 0.204 8,017,273.561245

14.502402 0.2041 8,088,592.547044

14.505803 0.2041 8,075,621.137988

14.509203 0.204 7,907,039.644581

14.512605 0.2041 7,940,601.483013

14.516007 0.2041 7,990,720.930682

14.519407 0.204 8,009,755.740232

14.522808 0.2041 7,958,156.606456

14.526210 0.2041 7,747,847.830459

14.529610 0.204 7,674,270.996172

14.533012 0.2041 7,921,226.378107

14.536413 0.2041 8,079,651.303849

14.539813 0.204 7,901,245.499106

14.543215 0.2041 7,851,966.335267

14.546617 0.2041 8,014,197.701278

14.550017 0.204 8,039,822.450644

14.553418 0.2041 7,906,337.814503

14.556820 0.2041 7,821,905.883793

14.560220 0.204 7,813,825.441585

14.563622 0.2041 7,883,578.213692

14.567023 0.2041 7,904,501.701786

14.570423 0.204 7,904,499.459040

14.573825 0.2041 8,023,118.495817

14.577227 0.2041 7,988,226.154928

14.580628 0.2041 8,019,020.711621

14.584028 0.204 8,272,072.028182

14.587430 0.2041 8,357,197.570253

14.590832 0.2041 8,353,023.395655

14.594232 0.204 8,425,759.657928

14.597633 0.2041 8,401,139.272010

14.601035 0.2041 8,406,207.092003

14.604435 0.204 8,406,384.333654

14.607837 0.2041 8,387,675.850833

14.611238 0.2041 8,533,802.762405

14.614638 0.204 8,362,757.456622

14.618040 0.2041 8,043,494.453253

14.621442 0.2041 8,025,554.493540

14.624842 0.204 8,127,202.125523

14.628243 0.2041 8,167,914.487029

14.631645 0.2041 7,954,938.384808

14.635045 0.204 7,888,753.310582

14.638447 0.2041 7,926,075.808022

14.641848 0.2041 7,771,965.959010

14.645248 0.204 7,795,473.413664

14.648650 0.2041 8,009,874.504671

14.652052 0.2041 7,893,815.850604

14.655452 0.204 7,711,455.038150

14.658853 0.2041 7,865,789.827274

14.662255 0.2041 7,922,012.731016

14.665655 0.204 7,886,977.057567

14.669057 0.2041 7,920,365.662526

14.672458 0.2041 7,808,500.888133

14.675858 0.204 7,819,917.088291

14.679260 0.2041 8,035,686.424205

14.682662 0.2041 7,858,613.980272

14.686062 0.204 7,740,488.063803

14.689463 0.2041 7,867,649.965063

14.692865 0.2041 7,731,918.294824

14.696265 0.204 7,658,668.897006

14.699667 0.2041 7,801,221.653593

14.703068 0.2041 7,770,510.626216

14.706468 0.204 7,624,190.472805

14.709870 0.2041 7,825,668.738640

14.713272 0.2041 7,970,564.392845

14.716672 0.204 7,942,403.933110

14.720073 0.2041 8,169,045.078494

14.723475 0.2041 8,406,458.787627

14.726875 0.204 8,643,387.737359

14.730277 0.2041 8,886,793.119317

14.733678 0.2041 9,121,097.618676

14.737078 0.204 9,613,142.036513

14.740480 0.2041 10,180,913.051458

14.743882 0.2041 10,984,215.683445

14.747282 0.204 11,803,637.184532

14.750683 0.2041 12,098,575.108415

14.754085 0.2041 12,631,014.394396

14.757485 0.204 13,296,514.559743

14.760887 0.2041 13,478,277.391691

14.764288 0.2041 13,534,496.520325

14.767688 0.204 13,247,737.132551

14.771090 0.2041 13,091,427.044921

14.774492 0.2041 12,813,257.680167

14.777892 0.204 12,022,572.929215

14.781293 0.2041 11,761,895.638390

14.784695 0.2041 11,197,391.855263

14.788095 0.204 10,483,538.971621

14.791497 0.2041 10,175,654.892265

14.794898 0.2041 9,573,344.613600

14.798298 0.204 9,425,002.996055

14.801700 0.2041 9,430,921.069988

14.805102 0.2041 9,050,765.215537

14.808502 0.204 8,763,851.727619

14.811903 0.2041 8,615,006.791664

14.815305 0.2041 8,657,315.827738

14.818705 0.204 8,662,763.997622

14.822107 0.2041 8,379,872.338782

14.825508 0.2041 8,205,707.239461

14.828908 0.204 8,348,664.981796

14.832310 0.2041 8,263,713.537573

14.835712 0.2041 8,119,539.394310

14.839112 0.204 8,154,045.716574

14.842513 0.2041 8,193,900.097437

14.845915 0.2041 8,247,004.830256

14.849315 0.204 8,105,976.049654

14.852717 0.2041 7,997,908.804661

14.856118 0.2041 8,256,652.522360

14.859518 0.204 8,273,564.069202

14.862920 0.2041 7,956,946.951457

14.866322 0.2041 8,097,139.063879

14.869722 0.204 8,273,089.582831

14.873123 0.2041 8,054,358.023935

14.876525 0.2041 8,115,142.969293

14.879925 0.204 8,205,511.292189

14.883327 0.2041 8,097,150.624502

14.886728 0.2041 8,231,698.719900

14.890128 0.204 8,166,978.019686

14.893530 0.2041 7,962,843.451584

14.896932 0.2041 7,915,877.384305

14.900332 0.204 7,904,222.587066

14.903733 0.2041 8,009,923.586882

14.907135 0.2041 7,996,698.542068

14.910535 0.204 7,866,158.463932

14.913937 0.2041 7,940,833.215344

14.917338 0.2041 8,144,273.191511

14.920740 0.2041 8,141,653.942106

14.924140 0.204 7,955,646.838319

14.927542 0.2041 8,075,187.418786

14.930943 0.2041 8,164,102.913146

14.934343 0.204 7,991,431.666465

14.937745 0.2041 8,074,845.921133

14.941147 0.2041 8,104,798.622367

14.944547 0.204 8,003,890.170713

14.947948 0.2041 8,069,441.597232

14.951350 0.2041 8,157,092.523706

14.954750 0.204 8,092,835.692996

14.958152 0.2041 7,948,855.146676

14.961553 0.2041 7,967,379.065303

14.964953 0.204 8,137,430.651744

14.968355 0.2041 8,168,414.272656

14.971757 0.2041 8,028,423.685104

14.975157 0.204 8,066,571.313649

14.978558 0.2041 7,932,629.131933

14.981960 0.2041 7,797,445.728438

14.985360 0.204 8,109,819.060665

14.988762 0.2041 8,099,818.746083

14.992163 0.2041 8,012,764.099631

14.995563 0.204 7,923,628.763297

14.998965 0.2041 7,736,771.443254

15.002367 0.2041 7,802,099.864227

15.005767 0.204 7,876,477.081780

15.009168 0.2041 8,010,498.069972

15.012570 0.2041 8,124,187.212174

15.015970 0.204 8,135,742.789314

15.019372 0.2041 8,119,456.267367

15.022773 0.2041 8,081,392.234647

15.026173 0.204 8,216,842.676755

15.029575 0.2041 8,458,186.736804

15.032977 0.2041 8,526,899.173705

15.036377 0.204 8,846,796.204997

15.039778 0.2041 8,951,040.981593

15.043180 0.2041 9,089,564.039999

15.046580 0.204 9,976,341.511948

15.049982 0.2041 10,409,653.651580

15.053383 0.2041 10,582,064.783089

15.056783 0.204 10,910,198.532754

15.060185 0.2041 11,127,684.872127

15.063587 0.2041 11,371,689.798618

15.066987 0.204 11,590,331.236330

15.070388 0.2041 11,657,290.230617

15.073790 0.2041 11,381,493.971733

15.077190 0.204 10,878,858.539405

15.080592 0.2041 10,582,357.147225

15.083993 0.2041 10,551,401.322917

15.087393 0.204 10,166,062.264356

15.090795 0.2041 9,844,743.485127

15.094197 0.2041 9,873,266.018847

15.097597 0.204 9,619,140.644846

15.100998 0.2041 9,572,449.166534

15.104400 0.2041 9,630,855.596379

15.107800 0.204 9,471,359.483317

15.111202 0.2041 9,593,127.891689

15.114603 0.2041 9,764,761.427725

15.118003 0.204 9,779,004.202354

15.121405 0.2041 9,819,985.734381

15.124807 0.2041 9,699,040.434979

15.128207 0.204 9,717,998.063066

15.131608 0.2041 9,714,102.595569

15.135010 0.2041 9,465,264.707572

15.138410 0.204 9,416,691.489601

15.141812 0.2041 9,236,028.211708

15.145213 0.2041 9,055,986.540997

15.148613 0.204 9,015,794.994097

15.152015 0.2041 8,755,831.569808

15.155417 0.2041 8,598,278.142587

15.158817 0.204 8,495,560.746067

15.162218 0.2041 8,237,627.613703

15.165620 0.2041 8,161,309.835939

15.169020 0.204 8,252,367.577925

15.172422 0.2041 8,308,284.269337

15.175823 0.2041 8,213,668.000548

15.179223 0.204 8,148,594.530262

15.182625 0.2041 8,263,457.447060

15.186027 0.2041 8,316,279.397791

15.189427 0.204 8,122,267.986585

15.192828 0.2041 8,073,308.583753

15.196230 0.2041 8,200,324.744089

15.199630 0.204 8,089,031.034577

15.203032 0.2041 8,095,607.978957

15.206433 0.2041 8,142,096.449828

15.209833 0.204 8,042,203.968294

15.213235 0.2041 8,116,395.175397

15.216637 0.2041 8,071,355.024591

15.220037 0.204 7,885,157.168117

15.223438 0.2041 7,871,856.050859

15.226840 0.2041 7,932,955.753462

15.230240 0.204 7,825,687.364015

15.233642 0.2041 7,741,528.445099

15.237043 0.2041 7,810,148.289461

15.240443 0.204 7,841,862.939959

15.243845 0.2041 7,762,047.746817

15.247247 0.2041 7,595,232.407124

15.250647 0.204 7,677,086.234803

15.254048 0.2041 7,769,012.176049

15.257450 0.2041 7,802,075.219877

15.260850 0.204 7,963,535.702867

15.264252 0.2041 7,980,277.233259

15.267653 0.2041 7,762,600.749087

15.271053 0.204 7,633,519.814367

15.274455 0.2041 7,754,361.138276

15.277857 0.2041 7,726,861.761197

15.281258 0.2041 7,724,374.542259

15.284658 0.204 7,956,817.837082

15.288060 0.2041 7,959,105.967538

15.291462 0.2041 7,812,191.551446

15.294862 0.204 7,949,652.888313

15.298263 0.2041 7,982,775.929330

15.301665 0.2041 7,729,600.113818

15.305065 0.204 7,775,024.662853

15.308467 0.2041 7,877,589.022281

15.311868 0.2041 7,907,648.998117

15.315268 0.204 7,911,607.038405

15.318670 0.2041 7,811,208.707596

15.322072 0.2041 7,855,670.249786

15.325472 0.204 7,672,511.655676

15.328873 0.2041 7,677,773.441346

15.332275 0.2041 8,016,424.895335

15.335675 0.204 7,952,080.061106

15.339077 0.2041 7,791,797.650454

15.342478 0.2041 7,878,017.867195

15.345878 0.204 7,848,021.983577

15.349280 0.2041 7,669,549.806638

15.352682 0.2041 7,796,977.548380

15.356082 0.204 7,871,985.269724

15.359483 0.2041 7,669,200.295517

15.362885 0.2041 7,799,393.470182

15.366285 0.204 7,720,119.940062

15.369687 0.2041 7,410,053.448705

15.373088 0.2041 7,627,495.973336

15.376488 0.204 7,846,662.508796

15.379890 0.2041 7,840,313.337963

15.383292 0.2041 7,844,937.512568

15.386692 0.204 7,909,408.357895

15.390093 0.2041 7,916,205.166237

15.393495 0.2041 7,953,413.069854

15.396895 0.204 8,097,618.903657

15.400297 0.2041 8,375,315.068508

15.403698 0.2041 8,898,462.358312

15.407098 0.204 9,690,070.879061

15.410500 0.2041 10,932,182.613343

15.413902 0.2041 12,651,096.393078

15.417302 0.204 15,479,065.970544

15.420703 0.2041 19,524,244.010018

15.424105 0.2041 24,139,134.306127

15.427505 0.204 30,048,831.178969

15.430907 0.2041 38,118,540.466166

15.434308 0.2041 47,518,600.013367

15.437708 0.204 57,238,648.268307

15.441110 0.2041 67,819,584.300728

15.444512 0.2041 78,666,542.001355

15.447912 0.204 88,165,656.112187

15.451313 0.2041 95,445,860.504574

15.454715 0.2041 100,837,351.088545

15.458115 0.204 105,328,572.737166

15.461517 0.2041 106,718,835.964620

15.464918 0.2041 102,502,016.604210

15.468318 0.204 94,541,956.174071

15.471720 0.2041 84,956,242.914477

15.475122 0.2041 74,098,196.523521

15.478522 0.204 63,184,323.050444

15.481923 0.2041 52,908,798.519075

15.485325 0.2041 43,511,194.206228

15.488725 0.204 35,039,714.362745

15.492127 0.2041 27,900,682.272638

15.495528 0.2041 22,533,143.628442

15.498928 0.204 18,356,895.209628

15.502330 0.2041 15,660,406.236302

15.505732 0.2041 14,090,471.224092

15.509132 0.204 12,958,680.335273

15.512533 0.2041 12,367,673.493274

15.515935 0.2041 12,471,419.524994

15.519335 0.204 13,166,047.975034

15.522737 0.2041 14,153,897.639991

15.526138 0.2041 15,467,492.673360

15.529538 0.204 17,159,894.011358

15.532940 0.2041 18,987,283.642896

15.536342 0.2041 20,927,417.350222

15.539742 0.204 23,075,822.672697

15.543143 0.2041 24,836,221.289902

15.546545 0.2041 26,332,951.921813

15.549945 0.204 27,140,309.207407

15.553347 0.2041 26,950,752.274345

15.556748 0.2041 26,468,423.275474

15.560148 0.204 25,203,102.122652

15.563550 0.2041 23,395,998.939145

15.566952 0.2041 21,984,678.598276

15.570352 0.204 20,287,992.138436

15.573753 0.2041 18,074,012.973090

15.577155 0.2041 16,306,559.121691

15.580555 0.204 14,798,412.376429

15.583957 0.2041 13,370,108.446375

15.587358 0.2041 12,202,241.472676

15.590758 0.204 11,221,062.865218

15.594160 0.2041 10,351,829.090054

15.597562 0.2041 9,967,676.067701

15.600962 0.204 9,757,688.984463

15.604363 0.2041 9,289,439.473410

15.607765 0.2041 8,943,931.544797

15.611165 0.204 8,808,068.274775

15.614567 0.2041 8,991,595.000085

15.617968 0.2041 9,003,420.101468

15.621368 0.204 8,742,745.178879

15.624770 0.2041 8,710,134.916983

15.628172 0.2041 8,907,904.635299

15.631573 0.2041 9,061,696.590375

15.634973 0.204 8,908,881.184900

15.638375 0.2041 8,851,778.785855

15.641777 0.2041 8,998,816.006032

15.645177 0.204 8,985,165.150510

15.648578 0.2041 8,947,174.110238

15.651980 0.2041 8,921,376.025759

15.655380 0.204 8,818,011.943810

15.658782 0.2041 8,554,006.752761

15.662183 0.2041 8,527,948.116449

15.665583 0.204 8,613,818.313160

15.668985 0.2041 8,509,194.542120

15.672387 0.2041 8,465,513.373021

15.675787 0.204 8,085,368.591926

15.679188 0.2041 8,092,772.720816

15.682590 0.2041 8,365,323.898771

15.685990 0.204 8,271,943.473652

15.689392 0.2041 8,261,588.431008

15.692793 0.2041 8,161,008.213758

15.696193 0.204 8,179,368.005402

15.699595 0.2041 8,174,825.258757

15.702997 0.2041 7,999,528.586715

15.706397 0.204 8,059,721.647634

15.709798 0.2041 8,076,414.475753

15.713200 0.2041 7,871,703.860252

15.716600 0.204 7,968,598.901661

15.720002 0.2041 8,088,814.765855

15.723403 0.2041 8,133,287.413158

15.726803 0.204 8,174,619.783326

15.730205 0.2041 8,010,699.271637

15.733607 0.2041 7,967,280.093190

15.737007 0.204 7,756,942.815191

15.740408 0.2041 7,791,436.189717

15.743810 0.2041 8,114,626.146427

15.747210 0.204 8,034,023.982902

15.750612 0.2041 8,008,820.070684

15.754013 0.2041 8,076,504.856811

15.757413 0.204 7,847,559.154135

15.760815 0.2041 7,769,346.562179

15.764217 0.2041 7,972,324.832144

15.767617 0.204 7,898,349.079610

15.771018 0.2041 7,913,468.890842

15.774420 0.2041 8,039,719.202923

15.777820 0.204 7,859,219.269435

15.781222 0.2041 7,708,342.797752

15.784623 0.2041 7,817,652.092307

15.788023 0.204 7,931,963.832282

15.791425 0.2041 7,836,510.360279

15.794827 0.2041 7,704,448.128437

15.798227 0.204 7,723,951.130164

15.801628 0.2041 8,011,877.949312

15.805030 0.2041 7,978,806.665802

15.808430 0.204 7,702,632.473244

15.811832 0.2041 7,720,847.184671

15.815233 0.2041 7,925,776.288779

15.818633 0.204 7,813,942.452748

15.822035 0.2041 7,695,314.229214

15.825437 0.2041 8,105,105.802702

15.828837 0.204 7,941,972.812656

15.832238 0.2041 7,696,875.222948

15.835640 0.2041 7,913,142.845778

15.839040 0.204 7,787,785.869115

15.842442 0.2041 7,672,277.949001

15.845843 0.2041 7,808,409.085625

15.849243 0.204 7,813,535.447974

15.852645 0.2041 7,657,137.390829

15.856047 0.2041 7,802,210.228746

15.859447 0.204 7,997,685.336518

15.862848 0.2041 7,935,547.903168

15.866250 0.2041 7,909,271.258479

15.869650 0.204 8,095,796.642207

15.873052 0.2041 8,031,822.845093

15.876453 0.2041 7,977,921.510043

15.879853 0.204 8,105,299.301685

15.883255 0.2041 7,941,390.788853

15.886657 0.2041 7,889,848.149265

15.890057 0.204 7,950,066.135898

15.893458 0.2041 8,066,443.762543

15.896860 0.2041 7,976,538.439962

15.900260 0.204 7,974,808.264826

15.903662 0.2041 8,211,805.564053

15.907063 0.2041 8,143,597.389629

15.910463 0.204 8,147,240.803797

15.913865 0.2041 8,306,303.722028

15.917267 0.2041 8,292,923.137600

15.920667 0.204 8,310,037.842332

15.924068 0.2041 8,465,225.688336

15.927470 0.2041 8,594,626.263021

15.930870 0.204 8,394,193.074473

15.934272 0.2041 8,213,055.067046

15.937673 0.2041 8,522,686.567478

15.941073 0.204 8,643,688.872237

15.944475 0.2041 8,587,560.549497

15.947877 0.2041 8,432,794.815472

15.951277 0.204 8,165,095.279296

15.954678 0.2041 8,210,510.762235

15.958080 0.2041 8,319,808.893168

15.961480 0.204 8,196,768.401641

15.964882 0.2041 8,089,992.534205

15.968283 0.2041 8,025,963.391153

15.971683 0.204 7,977,007.119498

15.975085 0.2041 7,973,772.940629

15.978487 0.2041 7,990,173.665897

15.981887 0.204 7,858,353.685709

15.985288 0.2041 7,714,695.263782

15.988690 0.2041 7,746,638.194972

15.992090 0.204 7,670,065.425471

15.995492 0.2041 7,775,256.319293

15.998893 0.2041 7,851,636.383287

16.002293 0.204 7,773,999.387867

16.005695 0.2041 7,713,025.426023

16.009097 0.2041 7,774,844.688236

16.012498 0.2041 7,755,948.288876

16.015898 0.204 7,731,074.203295

16.019300 0.2041 7,981,139.713938

16.022702 0.2041 7,824,561.664285

16.026102 0.204 7,629,554.108218

16.029503 0.2041 7,755,665.536346

16.032905 0.2041 7,681,699.585041

16.036305 0.204 7,687,462.518510

16.039707 0.2041 7,866,897.938928

16.043108 0.2041 7,926,626.832574

16.046508 0.204 7,917,273.571808

16.049910 0.2041 7,848,111.874642

16.053312 0.2041 7,865,012.987584

16.056712 0.204 7,636,670.713592

16.060113 0.2041 7,593,690.202582

16.063515 0.2041 7,875,036.906292

16.066915 0.204 7,752,904.026972

16.070317 0.2041 7,737,556.571067

16.073718 0.2041 7,828,816.137632

16.077118 0.204 7,752,386.249465

16.080520 0.2041 7,711,497.537538

16.083922 0.2041 7,668,854.544081

16.087322 0.204 7,619,882.303184

16.090723 0.2041 7,780,946.097892

16.094125 0.2041 7,806,709.781075

16.097525 0.204 7,530,814.420783

16.100927 0.2041 7,578,197.138176

16.104328 0.2041 7,780,194.511004

16.107728 0.204 7,905,501.339980

16.111130 0.2041 7,840,730.145426

16.114532 0.2041 7,759,871.110001

16.117932 0.204 7,950,402.336805

16.121333 0.2041 7,988,358.597124

16.124735 0.2041 8,000,958.857506

16.128135 0.204 8,375,688.155625

16.131537 0.2041 8,988,208.603268

16.134938 0.2041 9,996,066.377438

16.138338 0.204 11,646,059.863447

16.141740 0.2041 14,055,871.520324

16.145142 0.2041 18,463,737.113092

16.148542 0.204 24,890,038.259300

16.151943 0.2041 33,500,412.824540

16.155345 0.2041 45,617,169.951784

16.158745 0.204 61,184,132.777106

16.162147 0.2041 80,630,240.281948

16.165548 0.2041 104,353,062.195479

16.168948 0.204 132,642,306.601199

16.172350 0.2041 162,894,057.945569

16.175752 0.2041 193,901,093.176595

16.179152 0.204 227,729,535.964317

16.182553 0.2041 259,787,464.058702

16.185955 0.2041 289,910,417.775498

16.189355 0.204 315,573,512.223522

16.192757 0.2041 327,791,128.250083

16.196158 0.2041 332,274,378.750424

16.199558 0.204 331,297,615.017358

16.202960 0.2041 313,907,060.179950

16.206362 0.2041 282,056,356.682383

16.209762 0.204 246,130,340.223374

16.213163 0.2041 206,031,201.387515

16.216565 0.2041 165,168,081.065681

16.219965 0.204 129,041,221.888768

16.223367 0.2041 97,472,000.167033

16.226768 0.2041 72,004,573.864840

16.230168 0.204 52,254,123.138629

16.233570 0.2041 38,356,140.069544

16.236972 0.2041 29,393,674.074680

16.240372 0.204 22,969,500.769117

16.243773 0.2041 19,013,290.616295

16.247175 0.2041 16,443,845.505272

16.250575 0.204 14,718,607.983378

16.253977 0.2041 13,712,411.264089

16.257378 0.2041 12,921,898.227068

16.260778 0.204 12,319,021.446762

16.264180 0.2041 11,832,732.355707

16.267582 0.2041 11,491,511.717965

16.270982 0.204 11,131,464.996324

16.274383 0.2041 10,966,489.486108

16.277785 0.2041 10,981,345.064966

16.281185 0.204 10,761,809.504479

16.284587 0.2041 10,423,272.619518

16.287988 0.2041 10,142,141.782249

16.291388 0.204 10,112,624.533280

16.294790 0.2041 10,157,412.077236

16.298192 0.2041 9,929,346.095664

16.301592 0.204 9,774,212.508612

16.304993 0.2041 9,681,534.148549

16.308395 0.2041 9,515,235.475884

16.311795 0.204 9,516,676.836825

16.315197 0.2041 9,458,562.277813

16.318598 0.2041 9,284,318.903916

16.321998 0.204 9,226,831.775683

16.325400 0.2041 9,256,106.995826

16.328802 0.2041 9,262,532.159973

16.332202 0.204 9,214,307.442442

16.335603 0.2041 9,083,285.576540

16.339005 0.2041 9,040,998.349218

16.342405 0.204 9,004,963.910751

16.345807 0.2041 8,879,411.410297

16.349208 0.2041 9,106,029.307562

16.352608 0.204 9,148,394.975603

16.356010 0.2041 8,932,585.840628

16.359412 0.2041 9,073,043.636344

16.362813 0.2041 9,134,000.244817

16.366213 0.204 8,900,799.839618

16.369615 0.2041 8,887,785.983949

16.373017 0.2041 8,840,633.439654

16.376417 0.204 8,687,115.243415

16.379818 0.2041 8,806,904.079759

16.383220 0.2041 8,649,483.897383

16.386620 0.204 8,419,018.572935

16.390022 0.2041 8,636,855.192318

16.393423 0.2041 8,658,092.190880

16.396823 0.204 8,499,874.555630

16.400225 0.2041 8,603,864.067419

16.403627 0.2041 8,643,388.493282

16.407027 0.204 8,598,715.716462

16.410428 0.2041 8,352,811.675079

16.413830 0.2041 8,309,074.241595

16.417230 0.204 8,524,047.320372

16.420632 0.2041 8,281,067.888726

16.424033 0.2041 8,232,112.269283

16.427433 0.204 8,441,332.044246

16.430835 0.2041 8,355,764.315197

16.434237 0.2041 8,345,900.221846

16.437637 0.204 8,347,470.423624

16.441038 0.2041 8,202,884.333381

16.444440 0.2041 8,217,128.445989

16.447840 0.204 8,239,866.754553

16.451242 0.2041 8,204,911.923010

16.454643 0.2041 8,222,581.170158

16.458043 0.204 8,106,833.365104

16.461445 0.2041 8,104,541.057399

16.464847 0.2041 8,290,848.095056

16.468247 0.204 8,270,102.669739

16.471648 0.2041 8,166,527.189573

16.475050 0.2041 8,224,581.397003

16.478450 0.204 8,207,338.141304

16.481852 0.2041 8,011,593.971457

16.485253 0.2041 7,990,293.118434

16.488653 0.204 8,126,441.249994

16.492055 0.2041 8,215,053.459757

16.495457 0.2041 8,356,231.134155

16.498857 0.204 8,312,869.604531

16.502258 0.2041 8,149,479.959427

16.505660 0.2041 8,171,582.200384

16.509060 0.204 8,356,453.093173

16.512462 0.2041 8,353,644.820604

16.515863 0.2041 8,215,194.028020

16.519263 0.204 8,212,277.601330

16.522665 0.2041 8,126,252.760027

16.526067 0.2041 8,082,304.113031

16.529467 0.204 8,111,710.041025

16.532868 0.2041 7,966,063.853351

16.536270 0.2041 8,044,364.020500

16.539670 0.204 8,277,849.779707

16.543072 0.2041 8,140,226.477796

16.546473 0.2041 7,936,598.357971

16.549873 0.204 7,791,118.509207

16.553275 0.2041 7,748,113.432776

16.556677 0.2041 7,748,757.518296

16.560077 0.204 7,714,797.757047

16.563478 0.2041 7,805,247.828707

16.566880 0.2041 7,868,488.871317

16.570280 0.204 7,738,789.953837

16.573682 0.2041 7,804,581.123562

16.577083 0.2041 8,001,649.278069

16.580483 0.204 7,885,992.155629

16.583885 0.2041 7,854,002.971427

16.587287 0.2041 7,818,340.039517

16.590687 0.204 7,755,681.268532

16.594088 0.2041 7,949,027.695234

16.597490 0.2041 8,056,988.078153

16.600890 0.204 7,987,006.226570

16.604292 0.2041 7,804,970.028697

16.607693 0.2041 7,773,241.963399

16.611093 0.204 8,047,485.016646

16.614495 0.2041 8,133,473.020247

16.617897 0.2041 7,898,215.084852

16.621297 0.204 7,886,357.532976

16.624698 0.2041 7,803,239.520826

16.628100 0.2041 7,751,107.889598

16.631500 0.204 7,969,008.991697

16.634902 0.2041 7,807,069.586299

16.638303 0.2041 7,779,972.256254

16.641703 0.204 7,949,896.780333

16.645105 0.2041 7,836,121.582173

16.648507 0.2041 7,780,893.998462

16.651907 0.204 7,943,819.149543

16.655308 0.2041 8,016,901.769426

16.658710 0.2041 7,942,521.991601

16.662110 0.204 7,954,265.560471

16.665512 0.2041 8,008,250.829189

16.668913 0.2041 8,068,581.502146

16.672313 0.204 8,204,731.871681

16.675715 0.2041 8,140,519.046192

16.679117 0.2041 7,993,127.252483

16.682517 0.204 8,210,855.704935

16.685918 0.2041 8,205,398.650381

16.689320 0.2041 8,096,497.934591

16.692720 0.204 8,360,589.064481

16.696122 0.2041 8,312,419.385967

16.699523 0.2041 8,147,677.544211

16.702923 0.204 8,236,552.220919

16.706325 0.2041 8,139,941.958518

16.709727 0.2041 8,084,978.185971

16.713128 0.2041 8,239,049.006701

16.716528 0.204 8,163,122.359391

16.719930 0.2041 8,002,124.867891

16.723332 0.2041 8,057,877.805127

16.726732 0.204 7,903,993.390470

16.730133 0.2041 7,929,106.109708

16.733535 0.2041 8,113,438.210305

16.736935 0.204 7,709,217.511056

16.740337 0.2041 7,672,637.050452

16.743738 0.2041 8,100,774.381471

16.747138 0.204 8,144,311.414116

16.750540 0.2041 7,873,383.579520

16.753942 0.2041 7,848,316.122201

16.757342 0.204 8,137,621.882828

16.760743 0.2041 8,119,655.024339

16.764145 0.2041 8,067,889.245802

16.767545 0.204 8,017,629.826025

16.770947 0.2041 8,074,440.028974

16.774348 0.2041 8,148,251.900323

16.777748 0.204 7,893,590.813385

16.781150 0.2041 7,890,069.239233

16.784552 0.2041 7,926,107.241333

16.787952 0.204 7,850,780.932121

16.791353 0.2041 7,886,889.270429

16.794755 0.2041 7,997,276.574254

16.798155 0.204 8,000,077.380373

16.801557 0.2041 7,816,040.329944

16.804958 0.2041 8,034,390.433128

16.808358 0.204 8,261,316.105538

16.811760 0.2041 8,105,060.306875

16.815162 0.2041 7,975,012.008679

16.818562 0.204 7,807,418.481012

16.821963 0.2041 7,810,880.260131

16.825365 0.2041 7,960,898.874701

16.828765 0.204 7,948,356.763756

16.832167 0.2041 7,842,315.605383

16.835568 0.2041 7,887,669.187047

16.838968 0.204 8,089,847.701970

16.842370 0.2041 8,028,988.704587

16.845772 0.2041 7,941,756.829456

16.849172 0.204 8,160,625.536781

16.852573 0.2041 8,294,723.445430

16.855975 0.2041 8,331,528.951432

16.859375 0.204 8,375,992.913300

16.862777 0.2041 8,650,602.085673

16.866178 0.2041 8,928,266.200844

16.869578 0.204 8,805,959.018120

16.872980 0.2041 8,825,668.094912

16.876382 0.2041 8,845,770.393946

16.879782 0.204 8,808,851.013974

16.883183 0.2041 8,831,966.431752

16.886585 0.2041 8,813,200.041361

16.889985 0.204 8,797,510.334805

16.893387 0.2041 8,670,166.646441

16.896788 0.2041 8,788,518.687946

16.900188 0.204 9,067,335.945837

16.903590 0.2041 9,414,418.626115

16.906992 0.2041 10,279,303.354559

16.910392 0.204 11,517,336.944347

16.913793 0.2041 13,020,010.500287

16.917195 0.2041 15,172,866.811170

16.920595 0.204 17,934,768.408302

16.923997 0.2041 21,472,770.021640

16.927398 0.2041 25,673,988.596586

16.930798 0.204 30,166,646.419325

16.934200 0.2041 35,376,925.130660

16.937602 0.2041 40,802,855.230448

16.941002 0.204 45,566,435.748363

16.944403 0.2041 49,434,459.893828

16.947805 0.2041 52,492,997.396250

16.951205 0.204 54,641,689.782020

16.954607 0.2041 55,091,999.694460

16.958008 0.2041 54,442,932.476484

16.961408 0.204 52,124,487.036297

16.964810 0.2041 47,432,089.262007

16.968212 0.2041 42,749,939.269654

16.971612 0.204 37,842,278.792031

16.975013 0.2041 32,212,579.232452

16.978415 0.2041 27,017,580.145128

16.981815 0.204 22,551,679.233356

16.985217 0.2041 19,113,177.739337

16.988618 0.2041 16,124,135.066628

16.992018 0.204 13,704,226.423024

16.995420 0.2041 12,121,800.635220

16.998822 0.2041 10,916,860.527574

17.002222 0.204 9,989,677.441403

17.005623 0.2041 9,487,992.758804

17.009025 0.2041 9,202,187.106685

17.012425 0.204 8,997,969.067353

17.015827 0.2041 9,098,470.689183

17.019228 0.2041 9,169,725.309252

17.022628 0.204 9,275,011.569549

17.026030 0.2041 9,597,211.706797

17.029432 0.2041 9,995,219.224488

17.032832 0.204 10,408,475.695581

17.036233 0.2041 10,894,170.986562

17.039635 0.2041 11,435,628.781752

17.043035 0.204 11,953,721.557996

17.046437 0.2041 12,250,567.155083

17.049838 0.2041 12,505,792.371425

17.053238 0.204 12,833,212.987569

17.056640 0.2041 12,801,122.827183

17.060042 0.2041 12,634,406.926520

17.063442 0.204 12,348,265.244872

17.066843 0.2041 12,191,697.521286

17.070245 0.2041 12,175,111.224835

17.073645 0.204 11,897,082.703333

17.077047 0.2041 11,360,410.553451

17.080448 0.2041 10,887,433.457444

17.083850 0.2041 10,628,071.776652

17.087250 0.204 10,331,637.706969

17.090652 0.2041 10,035,713.755233

17.094053 0.2041 9,800,954.651628

17.097453 0.204 9,449,329.980613

17.100855 0.2041 9,061,747.074117

17.104257 0.2041 8,890,789.934575

17.107657 0.204 8,673,867.355908

17.111058 0.2041 8,643,457.967637

17.114460 0.2041 8,663,877.274088

17.117860 0.204 8,513,658.394374

17.121262 0.2041 8,575,538.803121

17.124663 0.2041 8,616,623.727840

17.128063 0.204 8,525,094.178359

17.131465 0.2041 8,307,782.725905

17.134867 0.2041 8,247,703.075780

17.138267 0.204 8,460,051.712310

17.141668 0.2041 8,395,696.859510

17.145070 0.2041 8,340,884.639691

17.148470 0.204 8,514,967.266660

17.151872 0.2041 8,473,725.408177

17.155273 0.2041 8,160,172.666247

17.158673 0.204 8,034,708.829970

17.162075 0.2041 8,185,468.684502

17.165477 0.2041 8,316,017.711967

17.168877 0.204 8,347,405.513525

17.172278 0.2041 8,281,625.380368

17.175680 0.2041 8,316,978.789009

17.179080 0.204 8,245,794.986163

17.182482 0.2041 8,196,022.321037

17.185883 0.2041 8,144,020.010515

17.189283 0.204 8,090,927.109669

17.192685 0.2041 8,216,389.981092

17.196087 0.2041 8,047,888.505106

17.199487 0.204 8,008,704.889539

17.202888 0.2041 8,121,509.404239

17.206290 0.2041 8,096,466.143008

17.209690 0.204 8,132,978.544238

17.213092 0.2041 8,048,580.139550

17.216493 0.2041 7,932,312.066073

17.219893 0.204 7,878,912.110572

17.223295 0.2041 7,919,147.527640

17.226697 0.2041 7,947,708.470384

17.230097 0.204 8,016,218.326099

17.233498 0.2041 8,080,243.723639

17.236900 0.2041 7,899,001.448042

17.240300 0.204 7,929,053.926515

17.243702 0.2041 8,086,760.414036

17.247103 0.2041 7,955,165.571714

17.250503 0.204 7,739,460.846064

17.253905 0.2041 7,681,322.679389

17.257307 0.2041 7,721,457.667017

17.260707 0.204 7,911,210.675902

17.264108 0.2041 8,084,117.011931

17.267510 0.2041 7,975,682.345496

17.270910 0.204 7,940,661.912831

17.274312 0.2041 7,930,048.417315

17.277713 0.2041 7,849,652.634850

17.281113 0.204 7,813,256.270423

17.284515 0.2041 7,736,917.423186

17.287917 0.2041 7,631,137.702982

17.291317 0.204 7,676,139.974213

17.294718 0.2041 7,817,967.146651

17.298120 0.2041 7,808,637.879534

17.301520 0.204 7,880,455.830567

17.304922 0.2041 7,875,426.313288

17.308323 0.2041 7,760,981.422901

17.311723 0.204 7,768,999.707101

17.315125 0.2041 7,649,413.989232

17.318527 0.2041 7,741,334.429836

17.321927 0.204 7,906,774.141674

17.325328 0.2041 7,709,465.456499

17.328730 0.2041 7,695,371.145474

17.332130 0.204 7,863,016.351245

17.335532 0.2041 7,734,162.282453

17.338933 0.2041 7,532,737.626178

17.342333 0.204 7,626,428.513683

17.345735 0.2041 7,708,128.238419

17.349137 0.2041 7,698,199.697634

17.352537 0.204 7,653,233.591268

17.355938 0.2041 7,719,316.087400

17.359340 0.2041 7,891,950.453692

17.362740 0.204 7,861,538.394417

17.366142 0.2041 7,842,365.579149

17.369543 0.2041 7,846,544.312724

17.372943 0.204 7,769,626.947583

17.376345 0.2041 7,805,938.969553

17.379747 0.2041 7,911,599.958258

17.383147 0.204 7,827,967.869428

17.386548 0.2041 7,751,960.306535

17.389950 0.2041 7,713,930.066699

17.393350 0.204 7,735,605.275195

17.396752 0.2041 7,731,617.719620

17.400153 0.2041 7,766,984.489720

17.403553 0.204 7,738,958.952587

17.406955 0.2041 7,689,142.576631

17.410357 0.2041 7,952,839.210198

17.413757 0.204 7,813,698.496202

17.417158 0.2041 7,635,360.720724

17.420560 0.2041 7,546,601.972931

17.423960 0.204 7,360,280.708487

17.427362 0.2041 7,547,748.649771

17.430763 0.2041 7,707,427.168325

17.434163 0.204 7,715,166.832204

17.437565 0.2041 7,741,499.124533

17.440967 0.2041 7,694,808.618103

17.444367 0.204 7,557,837.715260

17.447768 0.2041 7,677,449.156810

17.451170 0.2041 7,640,525.994687

17.454572 0.2041 7,437,718.047121

17.457972 0.204 7,580,419.380554

17.461373 0.2041 7,776,779.757011

17.464775 0.2041 7,815,944.215761

17.468175 0.204 7,747,814.305241

17.471577 0.2041 7,710,632.208788

17.474978 0.2041 7,804,749.453673

17.478378 0.204 7,901,660.217070

17.481780 0.2041 7,775,674.035034

17.485182 0.2041 7,762,966.647379

17.488582 0.204 7,853,021.989273

17.491983 0.2041 7,950,554.103280

17.495385 0.2041 8,276,030.947208

17.498785 0.204 8,494,950.025911

17.502187 0.2041 8,299,692.258221

17.505588 0.2041 8,061,915.326541

17.508988 0.204 8,209,687.971914

17.512390 0.2041 8,305,370.267987

17.515792 0.2041 8,034,114.313371

17.519192 0.204 7,957,020.656118

17.522593 0.2041 8,064,221.187079

17.525995 0.2041 7,987,225.876617

17.529395 0.204 7,907,528.885959

17.532797 0.2041 7,856,312.158223

17.536198 0.2041 7,815,941.449342

17.539598 0.204 7,867,583.749494

17.543000 0.2041 7,976,868.339643

17.546402 0.2041 7,929,684.056948

17.549802 0.204 7,764,510.474603

17.553203 0.2041 7,601,267.111090

17.556605 0.2041 7,737,499.399797

17.560005 0.204 8,208,769.541691

17.563407 0.2041 8,387,385.907193

17.566808 0.2041 8,795,286.855199

17.570208 0.204 9,355,842.601815

17.573610 0.2041 9,625,491.022234

17.577012 0.2041 10,196,726.294521

17.580412 0.204 10,707,142.150741

17.583813 0.2041 11,086,601.228338

17.587215 0.2041 11,731,323.240298

17.590615 0.204 12,229,377.465069

17.594017 0.2041 12,486,374.600808

17.597418 0.2041 12,816,749.522342

17.600818 0.204 12,877,135.187100

17.604220 0.2041 12,364,000.148243

17.607622 0.2041 12,028,706.488768

17.611022 0.204 11,882,903.235389

17.614423 0.2041 11,324,176.384149

17.617825 0.2041 10,644,016.631785

17.621225 0.204 9,978,563.217064

17.624627 0.2041 9,637,001.600147

17.628028 0.2041 9,425,662.271174

17.631428 0.204 8,855,179.598697

17.634830 0.2041 8,416,364.742550

17.638232 0.2041 8,397,473.287452

17.641632 0.204 8,370,294.890807

17.645033 0.2041 8,217,270.941346

17.648435 0.2041 8,140,089.707597

17.651835 0.204 8,011,061.746718

17.655237 0.2041 8,028,127.946122

17.658638 0.2041 8,136,706.095395

17.662038 0.204 7,943,601.010323

17.665440 0.2041 7,702,988.417222

17.668842 0.2041 7,780,236.986271

17.672242 0.204 7,905,114.929213

17.675643 0.2041 7,730,619.246851

17.679045 0.2041 7,631,171.387771

17.682445 0.204 7,601,622.336103

17.685847 0.2041 7,596,382.723707

17.689248 0.2041 7,564,845.451141

17.692648 0.204 7,411,810.345599

17.696050 0.2041 7,669,861.300572

17.699452 0.2041 7,749,671.425778

17.702852 0.204 7,540,757.893741

17.706253 0.2041 7,731,251.477428

17.709655 0.2041 7,752,196.424180

17.713055 0.204 7,595,503.177578

17.716457 0.2041 7,675,524.431522

17.719858 0.2041 7,733,725.191240

17.723258 0.204 7,640,632.378642

17.726660 0.2041 7,545,076.531059

17.730062 0.2041 7,559,699.310330

17.733462 0.204 7,486,151.002928

17.736863 0.2041 7,596,067.140106

17.740265 0.2041 7,756,087.501428

17.743665 0.204 7,569,920.062948

17.747067 0.2041 7,661,964.492013

17.750468 0.2041 7,723,123.255245

17.753868 0.204 7,583,613.672349

17.757270 0.2041 7,712,982.171054

17.760672 0.2041 7,661,098.937477

17.764072 0.204 7,542,386.942282

17.767473 0.2041 7,659,740.023683

17.770875 0.2041 7,582,700.166676

17.774275 0.204 7,383,774.669958

17.777677 0.2041 7,587,632.203899

17.781078 0.2041 7,757,229.434206

17.784478 0.204 7,617,132.575352

17.787880 0.2041 7,604,459.261208

17.791282 0.2041 7,649,763.680233

17.794682 0.204 7,551,337.515460

17.798083 0.2041 7,491,399.343912

17.801485 0.2041 7,591,603.640583

17.804885 0.204 7,646,386.616080

17.808287 0.2041 7,649,989.364883

17.811688 0.2041 7,658,369.835984

17.815090 0.2041 7,663,997.615962

17.818490 0.204 7,731,412.385730

17.821892 0.2041 7,821,954.255030

17.825293 0.2041 7,816,947.531576

17.828693 0.204 7,794,595.687532

17.832095 0.2041 7,906,254.589619

17.835497 0.2041 7,837,974.807194

17.838897 0.204 7,581,469.899615

17.842298 0.2041 7,633,522.796187

17.845700 0.2041 7,818,161.949704

17.849100 0.204 7,841,272.440795

17.852502 0.2041 7,789,113.029205

17.855903 0.2041 7,629,180.150708

17.859303 0.204 7,606,201.772526

17.862705 0.2041 7,805,921.632410

17.866107 0.2041 7,705,268.859290

17.869507 0.204 7,598,190.889735

17.872908 0.2041 7,582,631.192784

17.876310 0.2041 7,494,443.223613

17.879710 0.204 7,730,612.932954

17.883112 0.2041 7,642,763.892827

17.886513 0.2041 7,509,363.957698

17.889913 0.204 7,789,725.470186

17.893315 0.2041 7,907,910.881624

17.896717 0.2041 7,860,803.420922

17.900117 0.204 7,838,829.021187

17.903518 0.2041 7,926,608.343744

17.906920 0.2041 7,807,344.772913

17.910320 0.204 7,605,167.898416

17.913722 0.2041 7,724,226.132677

17.917123 0.2041 7,840,090.966143

17.920523 0.204 7,960,438.804583

17.923925 0.2041 8,061,608.156380

17.927327 0.2041 7,854,215.889136

17.930727 0.204 7,904,966.004081

17.934128 0.2041 8,107,156.426050

17.937530 0.2041 8,045,769.501241

17.940930 0.204 7,947,601.168829

17.944332 0.2041 8,034,336.767052

17.947733 0.2041 8,116,475.494660

17.951133 0.204 8,013,448.221197

17.954535 0.2041 8,128,897.627113

17.957937 0.2041 8,200,793.431362

17.961337 0.204 8,175,371.858775

17.964738 0.2041 8,427,550.968350

17.968140 0.2041 8,510,434.905147

17.971540 0.204 8,669,824.839207

17.974942 0.2041 9,093,061.963195

17.978343 0.2041 9,188,293.800161

17.981743 0.204 9,505,627.091892

17.985145 0.2041 10,263,710.091887

17.988547 0.2041 10,741,524.761834

17.991947 0.204 11,053,872.149863

17.995348 0.2041 11,687,614.318104

17.998750 0.2041 12,185,943.139471

18.002150 0.204 12,261,855.328272

18.005552 0.2041 12,286,804.630621

18.008953 0.2041 12,279,365.600711

18.012353 0.204 12,140,089.686131

18.015755 0.2041 11,898,553.813474

18.019157 0.2041 11,490,124.747634

18.022557 0.204 10,767,189.425643

18.025958 0.2041 10,074,561.590118

18.029360 0.2041 9,675,551.467178

18.032760 0.204 9,335,708.558900

18.036162 0.2041 8,867,734.643900

18.039563 0.2041 8,404,875.677263

18.042963 0.204 8,306,459.944646

18.046365 0.2041 8,071,361.077613

18.049767 0.2041 7,735,339.130976

18.053167 0.204 7,692,760.277402

18.056568 0.2041 7,747,336.851704

18.059970 0.2041 7,911,163.434474

18.063370 0.204 7,880,174.344797

18.066772 0.2041 7,913,809.441675

18.070173 0.2041 7,897,117.043318

18.073573 0.204 7,699,128.790356

18.076975 0.2041 7,710,431.097709

18.080377 0.2041 7,717,799.381730

18.083777 0.204 7,728,465.912607

18.087178 0.2041 7,652,991.355117

18.090580 0.2041 7,650,870.916335

18.093980 0.204 7,777,546.032805

18.097382 0.2041 7,695,169.664658

18.100783 0.2041 7,523,200.187610

18.104183 0.204 7,586,700.546071

18.107585 0.2041 7,856,713.279899

18.110987 0.2041 7,911,612.516873

18.114387 0.204 7,808,929.696829

18.117788 0.2041 7,853,496.772526

18.121190 0.2041 7,814,600.709162

18.124590 0.204 7,588,788.848818

18.127992 0.2041 7,622,401.829926

18.131393 0.2041 7,838,961.581448

18.134793 0.204 7,897,879.328848

18.138195 0.2041 8,005,494.361293

18.141597 0.2041 8,105,242.796517

18.144997 0.204 8,089,954.573518

18.148398 0.2041 8,092,174.885776

18.151800 0.2041 8,227,651.190567

18.155200 0.204 8,383,882.270706

18.158602 0.2041 8,487,639.107632

18.162003 0.2041 8,904,777.683367

18.165405 0.2041 9,142,599.389300

18.168805 0.204 9,188,510.585181

18.172207 0.2041 9,811,350.495015

18.175608 0.2041 10,237,295.952307

18.179008 0.204 10,375,244.687802

18.182410 0.2041 10,648,706.055604

18.185812 0.2041 10,735,943.642169

18.189212 0.204 10,798,092.898333

18.192613 0.2041 10,713,574.359822

18.196015 0.2041 10,523,633.830091

18.199415 0.204 10,188,599.556866

18.202817 0.2041 9,812,259.156399

18.206218 0.2041 9,866,567.874385

18.209618 0.204 9,750,638.085635

18.213020 0.2041 9,346,174.302674

18.216422 0.2041 9,037,042.146039

18.219822 0.204 8,723,890.995231

18.223223 0.2041 8,694,233.933768

18.226625 0.2041 8,734,017.824732

18.230025 0.204 8,448,337.017085

18.233427 0.2041 8,269,395.662865

18.236828 0.2041 8,124,163.924251

18.240228 0.204 7,984,165.657488

18.243630 0.2041 8,141,302.629631

18.247032 0.2041 8,256,634.630541

18.250432 0.204 8,199,301.695161

18.253833 0.2041 8,108,000.330913

18.257235 0.2041 8,087,021.844603

18.260635 0.204 8,199,631.164772

18.264037 0.2041 8,240,389.045452

18.267438 0.2041 8,450,447.055237

18.270838 0.204 8,677,305.278244

18.274240 0.2041 8,677,402.346129

18.277642 0.2041 8,728,371.163572

18.281042 0.204 8,784,418.564402

18.284443 0.2041 9,102,945.971059

18.287845 0.2041 9,349,086.129042

18.291245 0.204 9,265,810.140612

18.294647 0.2041 9,319,871.962534

18.298048 0.2041 9,291,887.620241

18.301448 0.204 9,230,719.045414

18.304850 0.2041 9,301,256.630801

18.308252 0.2041 9,196,638.942440

18.311652 0.204 9,007,519.928408

18.315053 0.2041 8,917,054.531250

18.318455 0.2041 8,975,256.840025

18.321855 0.204 8,814,690.453748

18.325257 0.2041 8,475,649.620052

18.328658 0.2041 8,511,303.988577

18.332058 0.204 8,575,351.406568

18.335460 0.2041 8,358,260.897522

18.338862 0.2041 8,170,102.829610

18.342262 0.204 8,100,654.670342

18.345663 0.2041 8,013,802.493179

18.349065 0.2041 7,857,468.505408

18.352465 0.204 8,039,726.980169

18.355867 0.2041 8,202,773.717827

18.359268 0.2041 7,985,448.448592

18.362668 0.204 7,949,364.731397

18.366070 0.2041 7,779,661.792932

18.369472 0.2041 7,743,635.959549

18.372872 0.204 7,937,147.753091

18.376273 0.2041 7,807,375.332260

18.379675 0.2041 7,942,508.444090

18.383075 0.204 8,003,631.228838

18.386477 0.2041 7,878,415.689790

18.389878 0.2041 7,958,634.453644

18.393278 0.204 7,829,239.483061

18.396680 0.2041 7,885,629.107607

18.400082 0.2041 7,927,908.776913

18.403482 0.204 7,826,744.110549

18.406883 0.2041 7,837,463.476200

18.410285 0.2041 7,868,261.917079

18.413685 0.204 7,987,142.776758

18.417087 0.2041 8,014,626.565281

18.420488 0.2041 7,827,857.440342

18.423888 0.204 7,633,567.741576

18.427290 0.2041 7,867,624.278316

18.430692 0.2041 7,995,555.278395

18.434092 0.204 7,818,446.439033

18.437493 0.2041 7,898,798.765709

18.440895 0.2041 7,937,176.397889

18.444295 0.204 7,555,646.725999

18.447697 0.2041 7,432,240.128150

18.451098 0.2041 7,680,947.078000

18.454498 0.204 7,583,558.569834

18.457900 0.2041 7,462,945.458663

18.461302 0.2041 7,527,555.379869

18.464702 0.204 7,617,281.138615

18.468103 0.2041 7,739,239.257713

18.471505 0.2041 7,745,114.643059

18.474905 0.204 7,595,270.523234

18.478307 0.2041 7,477,122.356292

18.481708 0.2041 7,588,525.751038

18.485108 0.204 7,711,927.321314

18.488510 0.2041 7,666,243.946023

18.491912 0.2041 7,736,492.425396

18.495312 0.204 7,846,359.810496

18.498713 0.2041 7,765,529.000182

18.502115 0.2041 7,792,358.081891

18.505515 0.204 7,778,142.755106

18.508917 0.2041 7,697,170.064206

18.512318 0.2041 7,720,984.866569

18.515718 0.204 7,708,254.724306

18.519120 0.2041 7,697,042.063470

18.522522 0.2041 7,684,057.548386

18.525923 0.2041 7,623,074.837397

18.529323 0.204 7,671,197.719787

18.532725 0.2041 7,789,134.252044

18.536127 0.2041 7,627,117.265788

18.539527 0.204 7,514,072.993833

18.542928 0.2041 7,672,685.601262

18.546330 0.2041 7,768,077.367954

18.549730 0.204 7,736,809.866186

18.553132 0.2041 7,810,405.693502

18.556533 0.2041 7,929,216.796325

18.559933 0.204 7,760,315.304513

18.563335 0.2041 7,615,213.363927

18.566737 0.2041 7,799,374.984360

18.570137 0.204 7,952,291.452880

18.573538 0.2041 7,831,449.706487

18.576940 0.2041 7,966,855.452192

18.580340 0.204 8,187,392.548262

18.583742 0.2041 8,230,719.754013

18.587143 0.2041 8,659,489.086421

18.590543 0.204 9,270,096.438758

18.593945 0.2041 9,981,695.486491

18.597347 0.2041 10,791,780.336823

18.600747 0.204 11,510,104.568574

18.604148 0.2041 12,475,936.690420

18.607550 0.2041 13,776,122.146639

18.610950 0.204 15,032,523.818367

18.614352 0.2041 15,853,108.915207

18.617753 0.2041 16,705,131.506730

18.621153 0.204 17,727,577.479056

18.624555 0.2041 18,387,874.959825

18.627957 0.2041 18,835,076.127359

18.631357 0.204 18,854,897.704261

18.634758 0.2041 18,551,290.638928

18.638160 0.2041 18,335,327.514742

18.641560 0.204 18,071,204.329116

18.644962 0.2041 17,737,672.213251

18.648363 0.2041 17,272,725.372063

18.651763 0.204 16,513,565.699268

18.655165 0.2041 15,787,496.888719

18.658567 0.2041 14,988,399.332327

18.661967 0.204 13,930,032.177190

18.665368 0.2041 13,268,450.001480

18.668770 0.2041 12,534,543.331720

18.672170 0.204 11,496,570.717829

18.675572 0.2041 10,777,131.742948

18.678973 0.2041 10,096,814.144313

18.682373 0.204 9,489,150.721017

18.685775 0.2041 9,037,043.771429

18.689177 0.2041 8,583,386.462007

18.692577 0.204 8,529,391.959295

18.695978 0.2041 8,542,472.994855

18.699380 0.2041 8,273,205.326261

18.702780 0.204 7,937,929.909113

18.706182 0.2041 7,770,879.634812

18.709583 0.2041 7,874,014.789939

18.712983 0.204 7,868,953.568144

18.716385 0.2041 7,807,092.230623

18.719787 0.2041 7,818,507.521249

18.723187 0.204 7,918,590.520271

18.726588 0.2041 7,849,615.273521

18.729990 0.2041 7,768,207.268718

18.733390 0.204 7,924,812.513904

18.736792 0.2041 7,977,604.919242

18.740193 0.2041 7,982,552.420282

18.743593 0.204 7,949,732.354984

18.746995 0.2041 8,196,716.886024

18.750397 0.2041 8,435,740.345861

18.753797 0.204 8,524,533.229117

18.757198 0.2041 8,845,837.372716

18.760600 0.2041 9,021,579.329322

18.764000 0.204 8,971,067.566929

18.767402 0.2041 9,218,047.435088

18.770803 0.2041 9,698,517.698297

18.774203 0.204 9,732,805.689449

18.777605 0.2041 9,566,740.037589

18.781007 0.2041 9,556,867.083143

18.784407 0.204 9,470,035.560922

18.787808 0.2041 9,223,415.433177

18.791210 0.2041 8,886,402.609398

18.794610 0.204 8,776,972.660996

18.798012 0.2041 8,793,817.587200

18.801413 0.2041 8,659,472.016758

18.804813 0.204 8,748,332.469431

18.808215 0.2041 8,855,683.735437

18.811617 0.2041 8,813,516.666081

18.815017 0.204 8,983,512.380292

18.818418 0.2041 9,253,868.313995

18.821820 0.2041 9,436,956.552133

18.825220 0.204 9,687,129.550506

18.828622 0.2041 10,173,409.476067

18.832023 0.2041 10,507,366.189786

18.835423 0.204 10,631,081.314410

18.838825 0.2041 10,980,670.084614

18.842227 0.2041 11,361,774.103961

18.845627 0.204 11,291,079.956776

18.849028 0.2041 11,173,389.722532

18.852430 0.2041 11,395,737.064001

18.855830 0.204 11,269,028.699767

18.859232 0.2041 11,016,789.671926

18.862633 0.2041 10,997,874.802112

18.866033 0.204 10,823,948.097483

18.869435 0.2041 10,746,131.819232

18.872837 0.2041 10,595,533.170335

18.876237 0.204 10,252,164.564518

18.879638 0.2041 10,177,071.602524

18.883040 0.2041 10,130,201.242894

18.886440 0.204 9,839,744.891080

18.889842 0.2041 9,599,047.177842

18.893243 0.2041 9,377,234.090746

18.896643 0.204 9,212,965.794112

18.900045 0.2041 9,274,366.043577

18.903447 0.2041 9,079,977.834926

18.906848 0.2041 8,808,197.220693

18.910248 0.204 8,902,492.408721

18.913650 0.2041 8,860,877.343105

18.917052 0.2041 8,808,253.291274

18.920452 0.204 8,917,997.458380

18.923853 0.2041 8,727,923.622031

18.927255 0.2041 8,707,467.231948

18.930655 0.204 8,942,704.770666

18.934057 0.2041 8,813,068.628049

18.937458 0.2041 8,717,732.236693

18.940858 0.204 8,654,601.937707

18.944260 0.2041 8,360,121.338943

18.947662 0.2041 8,331,271.431418

18.951062 0.204 8,411,140.546021

18.954463 0.2041 8,410,426.556159

18.957865 0.2041 8,358,475.078017

18.961265 0.204 8,328,900.969896

18.964667 0.2041 8,416,660.569964

18.968068 0.2041 8,223,955.423943

18.971468 0.204 8,032,334.468881

18.974870 0.2041 7,990,065.770716

18.978272 0.2041 7,840,292.648492

18.981672 0.204 7,831,390.738726

18.985073 0.2041 7,987,127.500726

18.988475 0.2041 8,044,839.634869

18.991875 0.204 7,816,709.290007

18.995277 0.2041 7,622,270.636404

18.998678 0.2041 7,607,707.334709

19.002078 0.204 7,660,981.800853

19.005480 0.2041 7,792,974.520620

19.008882 0.2041 7,819,649.497571

19.012282 0.204 7,650,967.121753

19.015683 0.2041 7,586,928.997633

19.019085 0.2041 7,651,379.889256

19.022485 0.204 7,575,781.805541

19.025887 0.2041 7,615,919.547847

19.029288 0.2041 7,735,338.256600

19.032688 0.204 7,643,592.930107

19.036090 0.2041 7,536,945.618149

19.039492 0.2041 7,549,124.971796

19.042892 0.204 7,572,508.902902

19.046293 0.2041 7,661,231.507466

19.049695 0.2041 7,671,570.743101

19.053095 0.204 7,530,650.203187

19.056497 0.2041 7,350,394.011380

19.059898 0.2041 7,381,696.128085

19.063298 0.204 7,656,076.871875

19.066700 0.2041 7,739,780.801346

19.070102 0.2041 7,708,019.389123

19.073502 0.204 7,646,118.366693

19.076903 0.2041 7,507,712.297737

19.080305 0.2041 7,520,416.418196

19.083705 0.204 7,567,199.518827

19.087107 0.2041 7,599,708.913346

19.090508 0.2041 7,733,658.960211

19.093908 0.204 7,708,637.863070

19.097310 0.2041 7,613,019.051900

19.100712 0.2041 7,573,195.413142

19.104112 0.204 7,490,961.928811

19.107513 0.2041 7,524,350.566674

19.110915 0.2041 7,559,492.086115

19.114315 0.204 7,464,165.899571

19.117717 0.2041 7,641,745.744473

19.121118 0.2041 7,706,578.491416

19.124518 0.204 7,497,039.961130

19.127920 0.2041 7,610,548.478183

19.131322 0.2041 7,547,293.588950

19.134722 0.204 7,519,626.360475

19.138123 0.2041 7,756,969.304089

19.141525 0.2041 7,801,562.107936

19.144925 0.204 7,839,507.315573

19.148327 0.2041 7,720,658.789386

19.151728 0.2041 7,608,302.523059

19.155128 0.204 7,708,174.444099

19.158530 0.2041 7,753,663.868500

19.161932 0.2041 7,590,685.618691

19.165332 0.204 7,531,568.289010

19.168733 0.2041 7,602,879.196429

19.172135 0.2041 7,551,580.907530

19.175535 0.204 7,566,456.977758

19.178937 0.2041 7,600,591.504841

19.182338 0.2041 7,423,036.757134

19.185738 0.204 7,368,931.687148

19.189140 0.2041 7,657,840.693722

19.192542 0.2041 7,624,096.790670

19.195942 0.204 7,458,704.704083

19.199343 0.2041 7,651,125.209120

19.202745 0.2041 7,621,668.473101

19.206145 0.204 7,440,037.350003

19.209547 0.2041 7,453,719.737328

19.212948 0.2041 7,599,381.536075

19.216348 0.204 7,727,243.886146

19.219750 0.2041 7,577,825.356754

19.223152 0.2041 7,592,378.087156

19.226552 0.204 7,735,850.061624

19.229953 0.2041 7,594,810.021053

19.233355 0.2041 7,624,743.978039

19.236755 0.204 7,800,140.381294

19.240157 0.2041 7,825,914.999839

19.243558 0.2041 7,840,132.197458

19.246958 0.204 7,884,207.591117

19.250360 0.2041 8,015,743.336532

19.253762 0.2041 8,014,511.892004

19.257162 0.204 8,033,906.005326

19.260563 0.2041 8,129,553.109059

19.263965 0.2041 7,986,463.512760

19.267365 0.204 7,932,491.287208

19.270767 0.2041 7,857,410.516814

19.274168 0.2041 7,796,442.238351

19.277570 0.2041 7,823,174.292662

19.280970 0.204 7,740,569.564318

19.284372 0.2041 7,702,443.250933

19.287773 0.2041 7,679,039.468430

19.291173 0.204 7,592,687.100646

19.294575 0.2041 7,520,316.093869

19.297977 0.2041 7,569,646.781746

19.301377 0.204 7,620,408.044823

19.304778 0.2041 7,643,911.260298

19.308180 0.2041 7,560,089.492254

19.311580 0.204 7,579,187.101145

19.314982 0.2041 7,698,070.068825

19.318383 0.2041 7,566,443.338179

19.321783 0.204 7,673,784.708808

19.325185 0.2041 7,807,575.436122

19.328587 0.2041 7,813,204.881776

19.331987 0.204 8,034,051.368277

19.335388 0.2041 7,949,363.516501

19.338790 0.2041 7,894,841.373101

19.342190 0.204 7,898,709.936144

19.345592 0.2041 7,731,585.635559

19.348993 0.2041 7,798,142.271809

19.352393 0.204 7,926,614.098999

19.355795 0.2041 8,114,819.732181

19.359197 0.2041 8,185,969.982354

19.362597 0.204 8,098,557.612211

19.365998 0.2041 8,339,932.994027

19.369400 0.2041 8,772,769.105180

19.372800 0.204 8,834,378.522525

19.376202 0.2041 9,140,903.102352

19.379603 0.2041 9,723,410.215244

19.383003 0.204 10,023,502.844411

19.386405 0.2041 10,397,437.933913

19.389807 0.2041 10,656,088.545902

19.393207 0.204 10,852,551.206477

19.396608 0.2041 11,057,037.450123

19.400010 0.2041 11,086,292.904651

19.403410 0.204 10,996,067.984596

19.406812 0.2041 10,685,180.210408

19.410213 0.2041 10,203,120.465047

19.413613 0.204 9,781,583.284288

19.417015 0.2041 9,365,821.349286

19.420417 0.2041 8,902,558.269049

19.423817 0.204 8,661,162.993435

19.427218 0.2041 8,481,647.452059

19.430620 0.2041 8,246,868.485981

19.434020 0.204 8,097,322.452365

19.437422 0.2041 7,905,683.789915

19.440823 0.2041 7,751,628.288128

19.444223 0.204 7,707,734.751062

19.447625 0.2041 7,552,898.566288

19.451027 0.2041 7,510,928.973285

19.454427 0.204 7,647,168.581667

19.457828 0.2041 7,580,786.130100

19.461230 0.2041 7,432,010.624067

19.464630 0.204 7,506,802.668786

19.468032 0.2041 7,666,789.525800

19.471433 0.2041 7,697,708.723229

19.474833 0.204 7,698,302.063326

19.478235 0.2041 7,613,375.856633

19.481637 0.2041 7,629,486.228637

19.485037 0.204 7,858,455.928166

19.488438 0.2041 7,828,232.972509

19.491840 0.2041 7,814,256.243132

19.495240 0.204 7,931,966.238719

19.498642 0.2041 7,822,045.111945

19.502043 0.2041 7,793,917.196320

19.505443 0.204 7,880,860.894717

19.508845 0.2041 7,918,856.437753

19.512247 0.2041 7,985,227.655490

19.515647 0.204 8,087,980.631566

19.519048 0.2041 7,993,537.400261

19.522450 0.2041 7,840,233.350166

19.525850 0.204 7,919,590.655626

19.529252 0.2041 8,006,353.372834

19.532653 0.2041 7,805,472.615572

19.536053 0.204 7,724,513.675692

19.539455 0.2041 8,027,029.363316

19.542857 0.2041 8,067,618.334727

19.546257 0.204 7,901,527.787616

19.549658 0.2041 7,851,773.433449

19.553060 0.2041 7,618,967.480020

19.556460 0.204 7,505,880.146339

19.559862 0.2041 7,523,131.338998

19.563263 0.2041 7,528,024.274044

19.566663 0.204 7,703,160.190887

19.570065 0.2041 7,697,093.277937

19.573467 0.2041 7,666,048.363170

19.576867 0.204 7,625,466.649542

19.580268 0.2041 7,635,147.013697

19.583670 0.2041 7,770,501.434881

19.587070 0.204 7,588,561.711583

19.590472 0.2041 7,466,300.519126

19.593873 0.2041 7,595,106.772737

19.597273 0.204 7,494,436.376191

19.600675 0.2041 7,448,192.525688

19.604077 0.2041 7,458,593.563781

19.607477 0.204 7,375,812.927046

19.610878 0.2041 7,505,730.269179

19.614280 0.2041 7,435,202.207299

19.617682 0.2041 7,372,325.945184

19.621082 0.204 7,522,757.243408

19.624483 0.2041 7,597,609.666065

19.627885 0.2041 7,728,690.939812

19.631285 0.204 7,761,695.492237

19.634687 0.2041 7,570,840.700404

19.638088 0.2041 7,352,639.895288

19.641488 0.204 7,535,640.221480

19.644890 0.2041 7,749,358.723669

19.648292 0.2041 7,663,809.942506

19.651692 0.204 7,690,175.960879

19.655093 0.2041 7,605,550.722922

19.658495 0.2041 7,436,813.864368

19.661895 0.204 7,409,699.088103

19.665297 0.2041 7,438,358.437234

19.668698 0.2041 7,610,912.899954

19.672098 0.204 7,664,629.629988

19.675500 0.2041 7,580,559.343028

19.678902 0.2041 7,753,776.915895

19.682302 0.204 7,804,315.873820

19.685703 0.2041 7,560,888.598761

19.689105 0.2041 7,602,069.360599

19.692505 0.204 7,732,412.175639

19.695907 0.2041 7,634,322.447751

19.699308 0.2041 7,767,093.577231

19.702708 0.204 7,747,956.840844

19.706110 0.2041 7,755,132.670493

19.709512 0.2041 8,088,538.025050

19.712912 0.204 7,960,982.532966

19.716313 0.2041 7,801,548.548778

19.719715 0.2041 7,973,630.219436

19.723115 0.204 8,136,619.628117

19.726517 0.2041 8,326,457.592852

19.729918 0.2041 8,225,029.498563

19.733318 0.204 7,962,262.462302

19.736720 0.2041 8,008,258.967489

19.740122 0.2041 7,933,498.258616

19.743522 0.204 7,804,753.646073

19.746923 0.2041 7,694,654.686326

19.750325 0.2041 7,698,013.417232

19.753725 0.204 7,679,771.474207

19.757127 0.2041 7,470,777.321203

19.760528 0.2041 7,718,104.421551

19.763928 0.204 7,697,056.227370

19.767330 0.2041 7,539,721.757757

19.770732 0.2041 7,777,969.582787

19.774132 0.204 7,706,720.747712

19.777533 0.2041 7,458,333.062410

19.780935 0.2041 7,372,681.419456

19.784335 0.204 7,429,040.990695

19.787737 0.2041 7,360,680.988048

19.791138 0.2041 7,389,388.093955

19.794538 0.204 7,487,420.373651

19.797940 0.2041 7,429,886.475766

19.801342 0.2041 7,391,918.795182

19.804742 0.204 7,444,598.909027

19.808143 0.2041 7,603,407.691152

19.811545 0.2041 7,519,340.628768

19.814945 0.204 7,477,221.268785

19.818347 0.2041 7,654,645.125470

19.821748 0.2041 7,616,066.602478

19.825148 0.204 7,706,196.244463

19.828550 0.2041 8,122,069.237127

19.831952 0.2041 8,513,240.418142

19.835352 0.204 9,104,181.098447

19.838753 0.2041 9,790,369.978073

19.842155 0.2041 10,732,830.019783

19.845555 0.204 12,130,521.307552

19.848957 0.2041 13,554,713.087536

19.852358 0.2041 15,270,024.550248

19.855758 0.204 17,339,918.440734

19.859160 0.2041 19,585,072.976344

19.862562 0.2041 21,587,293.464088

19.865962 0.204 23,356,157.150549

19.869363 0.2041 25,111,615.302536

19.872765 0.2041 25,805,960.317392

19.876165 0.204 25,554,634.172000

19.879567 0.2041 25,532,342.981902

19.882968 0.2041 24,878,209.988754

19.886368 0.204 22,910,412.656382

19.889770 0.2041 21,060,724.071576

19.893172 0.2041 19,038,633.514606

19.896572 0.204 16,473,821.109396

19.899973 0.2041 14,446,868.799784

19.903375 0.2041 12,617,353.720979

19.906775 0.204 11,128,453.485477

19.910177 0.2041 10,143,315.273233

19.913578 0.2041 9,245,546.439308

19.916978 0.204 8,575,195.143167

19.920380 0.2041 8,254,016.409726

19.923782 0.2041 8,156,531.538465

19.927182 0.204 8,038,191.883166

19.930583 0.2041 7,907,919.029569

19.933985 0.2041 7,716,317.926033

19.937385 0.204 7,610,679.503021

19.940787 0.2041 7,785,842.906772

19.944188 0.2041 7,809,721.595058

19.947588 0.204 7,557,238.776857

19.950990 0.2041 7,535,729.771011

19.954392 0.2041 7,668,884.120821

19.957792 0.204 7,596,015.414802

19.961193 0.2041 7,333,376.540141

19.964595 0.2041 7,382,153.943170

19.967997 0.2041 7,665,575.980482

19.971397 0.204 7,644,409.116998

19.974798 0.2041 7,692,076.845637

19.978200 0.2041 7,662,634.199226

19.981600 0.204 7,586,986.521000

19.985002 0.2041 7,610,499.340521

19.988403 0.2041 7,593,781.826520

19.991803 0.204 7,711,333.437450

19.995205 0.2041 7,636,101.234024

19.998607 0.2041 7,609,465.373756

20.002007 0.204 7,639,456.868176

20.005408 0.2041 7,583,794.940849

20.008810 0.2041 7,764,635.347768

20.012210 0.204 7,735,626.613559

20.015612 0.2041 7,593,934.071416

20.019013 0.2041 7,775,908.925926

20.022413 0.204 7,889,635.741083

20.025815 0.2041 7,897,743.311076

20.029217 0.2041 8,151,320.086050

20.032617 0.204 8,273,933.573670

20.036018 0.2041 8,345,063.516073

20.039420 0.2041 8,792,006.520171

20.042820 0.204 9,104,332.568262

20.046222 0.2041 9,218,844.473914

20.049623 0.2041 9,521,043.604233

20.053023 0.204 10,072,878.191026

20.056425 0.2041 10,412,997.143395

20.059827 0.2041 10,445,958.769205

20.063227 0.204 10,788,463.126006

20.066628 0.2041 11,251,080.006801

20.070030 0.2041 11,224,294.720267

20.073430 0.204 10,928,217.686194

20.076832 0.2041 10,967,462.657035

20.080233 0.2041 11,057,709.270149

20.083633 0.204 10,538,235.398947

20.087035 0.2041 10,009,282.876593

20.090437 0.2041 9,812,383.572932

20.093837 0.204 9,445,332.539456

20.097238 0.2041 9,060,803.087617

20.100640 0.2041 8,696,405.149036

20.104040 0.204 8,442,842.236433

20.107442 0.2041 8,301,179.866885

20.110843 0.2041 8,105,514.730777

20.114243 0.204 8,018,183.623482

20.117645 0.2041 7,927,479.247611

20.121047 0.2041 7,854,983.840987

20.124447 0.204 7,779,187.361342

20.127848 0.2041 7,570,544.214483

20.131250 0.2041 7,543,938.790439

20.134650 0.204 7,615,593.522842

20.138052 0.2041 7,526,187.322740

20.141453 0.2041 7,466,647.790122

20.144853 0.204 7,362,918.189437

20.148255 0.2041 7,301,220.912865

20.151657 0.2041 7,461,650.913141

20.155057 0.204 7,683,149.645152

20.158458 0.2041 7,630,844.107207

20.161860 0.2041 7,442,518.747915

20.165260 0.204 7,471,777.207962

20.168662 0.2041 7,506,264.922540

20.172063 0.2041 7,632,906.394778

20.175463 0.204 7,669,633.300667

20.178865 0.2041 7,463,026.240608

20.182267 0.2041 7,506,294.180409

20.185667 0.204 7,684,436.448930

20.189068 0.2041 7,536,350.025717

20.192470 0.2041 7,431,740.305269

20.195870 0.204 7,502,501.329519

20.199272 0.2041 7,461,456.411502

20.202673 0.2041 7,508,593.581997

20.206073 0.204 7,589,286.606371

20.209475 0.2041 7,480,200.741902

20.212877 0.2041 7,456,517.359381

20.216277 0.204 7,377,748.962879

20.219678 0.2041 7,381,946.893094

20.223080 0.2041 7,515,356.688244

20.226480 0.204 7,497,385.102722

20.229882 0.2041 7,624,267.569899

20.233283 0.2041 7,506,216.714659

20.236683 0.204 7,368,880.980994

20.240085 0.2041 7,513,969.032718

20.243487 0.2041 7,549,168.646458

20.246887 0.204 7,553,344.323036

20.250288 0.2041 7,673,422.455016

20.253690 0.2041 7,485,781.845634

20.257090 0.204 7,300,020.157909

20.260492 0.2041 7,651,506.052974

20.263893 0.2041 7,664,997.880559

20.267293 0.204 7,447,757.768286

20.270695 0.2041 7,507,847.479222

20.274097 0.2041 7,524,062.077320

20.277497 0.204 7,434,895.755288

20.280898 0.2041 7,468,638.486347

20.284300 0.2041 7,491,252.907904

20.287700 0.204 7,363,609.089113

20.291102 0.2041 7,356,226.281218

20.294503 0.2041 7,497,129.361147

20.297903 0.204 7,547,556.385837

20.301305 0.2041 7,478,852.071182

20.304707 0.2041 7,473,306.993164

20.308107 0.204 7,494,555.430726

20.311508 0.2041 7,543,672.886856

20.314910 0.2041 7,531,420.875748

20.318310 0.204 7,520,562.266089

20.321712 0.2041 7,637,781.585367

20.325113 0.2041 7,784,109.171020

20.328515 0.2041 7,722,663.353898

20.331915 0.204 7,762,211.937602

20.335317 0.2041 8,134,487.591070

20.338718 0.2041 8,247,291.751142

20.342118 0.204 8,041,164.662316

20.345520 0.2041 8,048,385.998590

20.348922 0.2041 8,067,719.702684

20.352322 0.204 7,861,517.642808

20.355723 0.2041 7,883,899.184579

20.359125 0.2041 7,949,197.576885

20.362525 0.204 7,927,477.607203

20.365927 0.2041 7,973,259.240052

20.369328 0.2041 7,813,546.045859

20.372728 0.204 7,618,717.674348

20.376130 0.2041 7,573,899.876026

20.379532 0.2041 7,546,170.296918

20.382932 0.204 7,609,130.934096

20.386333 0.2041 7,714,962.404028

20.389735 0.2041 7,739,169.029499

20.393135 0.204 7,726,159.159149

20.396537 0.2041 7,740,553.974423

20.399938 0.2041 7,662,392.395604

20.403338 0.204 7,671,747.385913

20.406740 0.2041 7,526,208.169883

20.410142 0.2041 7,376,396.252468

20.413542 0.204 7,524,161.083787

20.416943 0.2041 7,402,827.768343

20.420345 0.2041 7,290,809.565238

20.423745 0.204 7,381,448.693816

20.427147 0.2041 7,285,228.579366

20.430548 0.2041 7,192,056.268762

20.433948 0.204 7,264,990.882425

20.437350 0.2041 7,246,673.604841

20.440752 0.2041 7,206,633.611453

20.444152 0.204 7,333,967.090375

20.447553 0.2041 7,385,456.115144

20.450955 0.2041 7,305,702.003515

20.454355 0.204 7,292,075.126314

20.457757 0.2041 7,255,542.360841

20.461158 0.2041 7,333,790.689970

20.464558 0.204 7,333,666.573360

20.467960 0.2041 7,312,363.826982

20.471362 0.2041 7,324,351.853704

20.474762 0.204 7,370,977.945308

20.478163 0.2041 7,464,854.595900

20.481565 0.2041 7,328,524.513869

20.484965 0.204 7,441,430.696619

20.488367 0.2041 7,610,806.664493

20.491768 0.2041 7,449,802.500667

20.495168 0.204 7,394,485.365209

20.498570 0.2041 7,362,262.188349

20.501972 0.2041 7,354,261.375968

20.505372 0.204 7,433,120.392383

20.508773 0.2041 7,438,823.172959

20.512175 0.2041 7,471,287.843939

20.515575 0.204 7,423,561.719470

20.518977 0.2041 7,395,714.759112

20.522378 0.2041 7,263,476.403137

20.525778 0.204 7,224,077.653406

20.529180 0.2041 7,426,681.703701

20.532582 0.2041 7,413,904.130264

20.535982 0.204 7,458,485.659158

20.539383 0.2041 7,391,292.587375

20.542785 0.2041 7,270,643.434021

20.546185 0.204 7,371,752.203060

20.549587 0.2041 7,295,673.306925

20.552988 0.2041 7,302,034.776703

20.556388 0.204 7,488,143.190890

20.559790 0.2041 7,534,187.726965

20.563192 0.2041 7,531,916.008809

20.566592 0.204 7,493,901.107110

20.569993 0.2041 7,541,729.226497

20.573395 0.2041 7,713,497.379929

20.576795 0.204 7,618,997.913580

20.580197 0.2041 7,537,802.175060

20.583598 0.2041 7,720,213.649234

20.586998 0.204 7,933,195.005915

20.590400 0.2041 7,972,389.257324

20.593802 0.2041 7,910,932.025306

20.597202 0.204 7,899,834.423495

20.600603 0.2041 7,931,787.872128

20.604005 0.2041 8,118,414.628512

20.607405 0.204 8,172,260.316764

20.610807 0.2041 7,943,844.448545

20.614208 0.2041 8,004,876.966804

20.617608 0.204 8,175,449.076096

20.621010 0.2041 7,964,116.563853

20.624412 0.2041 7,875,380.648805

20.627812 0.204 7,815,545.207475

20.631213 0.2041 7,726,879.189281

20.634615 0.2041 7,765,682.064336

20.638015 0.204 7,676,250.975436

20.641417 0.2041 7,553,880.583979

20.644818 0.2041 7,512,882.949085

20.648218 0.204 7,574,578.344817

20.651620 0.2041 7,566,770.374082

20.655022 0.2041 7,495,575.148070

20.658422 0.204 7,541,676.231702

20.661823 0.2041 7,596,483.346962

20.665225 0.2041 7,480,093.205721

20.668625 0.204 7,394,019.348833

20.672027 0.2041 7,393,775.597733

20.675428 0.2041 7,312,781.343186

20.678830 0.2041 7,223,119.785808

20.682230 0.204 7,358,900.885636

20.685632 0.2041 7,414,716.292811

20.689033 0.2041 7,225,886.451254

20.692433 0.204 7,284,063.526019

20.695835 0.2041 7,400,636.396133

20.699237 0.2041 7,446,971.501658

20.702637 0.204 7,505,571.468478

20.706038 0.2041 7,422,904.966773

20.709440 0.2041 7,337,814.331373

20.712840 0.204 7,407,085.219201

20.716242 0.2041 7,415,230.470605

20.719643 0.2041 7,385,098.188418

20.723043 0.204 7,491,167.116016

20.726445 0.2041 7,531,388.383154

20.729847 0.2041 7,344,957.066853

20.733247 0.204 7,199,070.421943

20.736648 0.2041 7,444,308.856799

20.740050 0.2041 7,501,407.892537

20.743450 0.204 7,408,610.037820

20.746852 0.2041 7,364,077.742644

20.750253 0.2041 7,281,910.042031

20.753653 0.204 7,322,012.607975

20.757055 0.2041 7,335,607.570099

20.760457 0.2041 7,503,421.751297

20.763857 0.204 7,561,052.851792

20.767258 0.2041 7,520,320.641822

20.770660 0.2041 7,473,750.178783

20.774060 0.204 7,448,997.276201

20.777462 0.2041 7,434,162.317913

20.780863 0.2041 7,399,632.163686

20.784263 0.204 7,562,288.121478

20.787665 0.2041 7,474,714.482459

20.791067 0.2041 7,292,350.683437

20.794467 0.204 7,359,264.514814

20.797868 0.2041 7,329,565.002625

20.801270 0.2041 7,247,046.258112

20.804670 0.204 7,514,159.841750

20.808072 0.2041 7,588,185.883589

20.811473 0.2041 7,335,052.972023

20.814873 0.204 7,403,312.821239

20.818275 0.2041 7,430,046.216363

20.821677 0.2041 7,375,469.496208

20.825077 0.204 7,432,704.638750

20.828478 0.2041 7,510,327.120452

20.831880 0.2041 7,528,606.858917

20.835280 0.204 7,427,643.362414

20.838682 0.2041 7,413,396.036582

20.842083 0.2041 7,383,347.723241

20.845483 0.204 7,366,288.710578

20.848885 0.2041 7,340,177.979088

20.852287 0.2041 7,369,788.436834

20.855687 0.204 7,404,939.055883

20.859088 0.2041 7,266,109.771116

20.862490 0.2041 7,296,384.433021

20.865890 0.204 7,388,743.290461

20.869292 0.2041 7,347,775.626669

20.872693 0.2041 7,347,615.321859

20.876093 0.204 7,317,591.425945

20.879495 0.2041 7,201,247.452125

20.882897 0.2041 7,303,857.450346

20.886297 0.204 7,373,584.529074

20.889698 0.2041 7,446,665.580832

20.893100 0.2041 7,635,793.653819

20.896500 0.204 7,581,237.124844

20.899902 0.2041 7,508,254.404429

20.903303 0.2041 7,533,186.116657

20.906703 0.204 7,668,696.572660

20.910105 0.2041 7,599,228.639710

20.913507 0.2041 7,492,969.497942

20.916907 0.204 7,751,296.095252

20.920308 0.2041 7,799,068.262924

20.923710 0.2041 7,634,741.920486

20.927110 0.204 7,498,181.990466

20.930512 0.2041 7,472,127.967636

20.933913 0.2041 7,518,951.743388

20.937313 0.204 7,399,352.798802

20.940715 0.2041 7,425,827.169089

20.944117 0.2041 7,546,629.945464

20.947517 0.204 7,550,754.164521

20.950918 0.2041 7,609,456.511606

20.954320 0.2041 7,662,727.339095

20.957720 0.204 7,453,538.014948

20.961122 0.2041 7,050,495.353879

20.964523 0.2041 7,186,432.348526

20.967923 0.204 7,364,141.018934

20.971325 0.2041 7,156,716.902456

20.974727 0.2041 7,256,636.519782

20.978127 0.204 7,373,832.134138

20.981528 0.2041 7,289,335.972201

20.984930 0.2041 7,297,025.059311

20.988330 0.204 7,287,364.687700

20.991732 0.2041 7,236,533.852900

20.995133 0.2041 7,223,037.712012

20.998533 0.204 7,271,765.055803

21.001935 0.2041 7,372,460.374153

21.005337 0.2041 7,334,215.394480

21.008737 0.204 7,242,505.961314

21.012138 0.2041 7,315,991.597753

21.015540 0.2041 7,395,035.162262

21.018940 0.204 7,440,630.419388

21.022342 0.2041 7,475,173.699100

21.025743 0.2041 7,298,782.804098

21.029145 0.2041 7,120,632.748758

21.032545 0.204 7,154,412.417018

21.035947 0.2041 7,301,531.966850

21.039348 0.2041 7,323,729.332189

21.042748 0.204 7,203,838.797397

21.046150 0.2041 7,206,922.979467

21.049552 0.2041 7,280,728.503960

21.052952 0.204 7,348,837.152518

21.056353 0.2041 7,394,295.502797

21.059755 0.2041 7,240,950.234072

21.063155 0.204 7,316,854.591706

21.066557 0.2041 7,425,091.612298

21.069958 0.2041 7,201,852.669481

21.073358 0.204 7,273,176.814683

21.076760 0.2041 7,320,584.448478

21.080162 0.2041 7,141,345.272441

21.083562 0.204 7,411,485.574203

21.086963 0.2041 7,554,334.234265

21.090365 0.2041 7,312,251.651473

21.093765 0.204 7,239,958.439998

21.097167 0.2041 7,255,546.340503

21.100568 0.2041 7,471,381.690950

21.103968 0.204 7,397,230.371189

21.107370 0.2041 7,277,998.487508

21.110772 0.2041 7,462,447.285948

21.114172 0.204 7,431,901.761645

21.117573 0.2041 7,377,505.384364

21.120975 0.2041 7,378,522.800229

21.124375 0.204 7,440,051.785736

21.127777 0.2041 7,524,160.734434

21.131178 0.2041 7,551,510.047559

21.134578 0.204 7,465,469.397842

21.137980 0.2041 7,309,197.328154

21.141382 0.2041 7,419,552.621711

21.144782 0.204 7,533,504.608095

21.148183 0.2041 7,554,194.007573

21.151585 0.2041 7,596,315.549379

21.154985 0.204 7,412,010.526699

21.158387 0.2041 7,294,385.813115

21.161788 0.2041 7,522,287.617053

21.165188 0.204 7,752,435.196603

21.168590 0.2041 7,643,679.196647

21.171992 0.2041 7,371,075.440329

21.175392 0.204 7,369,045.543240

21.178793 0.2041 7,631,138.698625

21.182195 0.2041 7,663,232.810876

21.185595 0.204 7,547,941.270840

21.188997 0.2041 7,415,006.038527

21.192398 0.2041 7,511,274.026175

21.195798 0.204 7,732,367.685389

21.199200 0.2041 7,781,788.202631

21.202602 0.2041 7,866,862.481336

21.206002 0.204 7,724,106.003589

21.209403 0.2041 7,639,482.531919

21.212805 0.2041 7,756,237.193396

21.216205 0.204 7,757,362.367421

21.219607 0.2041 7,725,792.793791

21.223008 0.2041 7,622,073.103406

21.226408 0.204 7,646,954.731533

21.229810 0.2041 7,739,236.126278

21.233212 0.2041 7,568,448.094282

21.236612 0.204 7,436,761.627052

21.240013 0.2041 7,469,761.690340

21.243415 0.2041 7,580,355.322827

21.246815 0.204 7,585,754.783350

21.250217 0.2041 7,419,871.674222

21.253618 0.2041 7,419,199.936084

21.257018 0.204 7,472,797.593818

21.260420 0.2041 7,305,547.369821

21.263822 0.2041 7,230,875.406905

21.267222 0.204 7,347,011.757019

21.270623 0.2041 7,258,044.725956

21.274025 0.2041 6,981,997.591987

21.277425 0.204 7,060,437.580404

21.280827 0.2041 7,253,074.243316

21.284228 0.2041 7,273,945.104028

21.287628 0.204 7,541,084.160738

21.291030 0.2041 7,629,065.029643

21.294432 0.2041 7,590,984.292737

21.297832 0.204 7,710,773.274916

21.301233 0.2041 7,788,538.237586

21.304635 0.2041 7,746,277.569424

21.308035 0.204 7,849,136.597795

21.311437 0.2041 8,167,482.927968

21.314838 0.2041 8,397,008.630100

21.318238 0.204 8,389,165.491194

21.321640 0.2041 8,235,402.346899

21.325042 0.2041 8,393,793.476331

21.328442 0.204 8,674,439.722543

21.331843 0.2041 8,606,387.750667

21.335245 0.2041 8,469,418.573101

21.338645 0.204 8,319,147.620916

21.342047 0.2041 7,980,920.061446

21.345448 0.2041 7,920,385.531549

21.348848 0.204 7,985,956.434852

21.352250 0.2041 7,965,461.966190

21.355652 0.2041 7,868,075.053149

21.359052 0.204 7,663,391.405463

21.362453 0.2041 7,721,365.732874

21.365855 0.2041 7,747,164.497191

21.369255 0.204 7,592,948.486671

21.372657 0.2041 7,485,596.099545

21.376058 0.2041 7,373,221.688494

21.379458 0.204 7,364,021.928997

21.382860 0.2041 7,526,352.194710

21.386262 0.2041 7,610,899.427332

21.389662 0.204 7,582,646.579094

21.393063 0.2041 7,525,048.453063

21.396465 0.2041 7,378,968.144958

21.399867 0.2041 7,431,458.316659

21.403267 0.204 7,641,713.045521

21.406668 0.2041 7,624,048.814401

21.410070 0.2041 7,601,904.239228

21.413470 0.204 7,614,362.437389

21.416872 0.2041 7,633,519.395667

21.420273 0.2041 7,620,826.809908

21.423673 0.204 7,629,547.347494

21.427075 0.2041 7,691,224.561434

21.430477 0.2041 7,600,038.545892

21.433877 0.204 7,705,069.894937

21.437278 0.2041 7,764,807.434462

21.440680 0.2041 7,460,498.804324

21.444080 0.204 7,457,375.985008

21.447482 0.2041 7,598,423.457688

21.450883 0.2041 7,556,399.582467

21.454283 0.204 7,707,844.443600

21.457685 0.2041 7,540,061.406088

21.461087 0.2041 7,403,601.127922

21.464487 0.204 7,488,732.873816

21.467888 0.2041 7,344,986.712422

21.471290 0.2041 7,348,682.832815

21.474690 0.204 7,352,307.463785

21.478092 0.2041 7,280,895.069349

21.481493 0.2041 7,153,213.015923

21.484893 0.204 7,197,699.175502

21.488295 0.2041 7,430,003.096064

21.491697 0.2041 7,484,315.165412

21.495097 0.204 7,388,407.494079

21.498498 0.2041 7,348,435.282192

21.501900 0.2041 7,543,815.819012

21.505300 0.204 7,630,573.199844

21.508702 0.2041 7,589,050.887084

21.512103 0.2041 7,629,066.855533

21.515503 0.204 7,655,234.082769

21.518905 0.2041 7,638,582.076604

21.522307 0.2041 7,589,164.623940

21.525707 0.204 7,614,672.497260

21.529108 0.2041 7,617,186.289411

21.532510 0.2041 7,516,334.935321

21.535910 0.204 7,432,279.699359

21.539312 0.2041 7,549,005.250258

21.542713 0.2041 7,644,551.576067

21.546113 0.204 7,651,608.185643

21.549515 0.2041 7,823,986.967001

21.552917 0.2041 8,232,908.354517

21.556317 0.204 9,224,074.169987

21.559718 0.2041 10,546,868.902433

21.563120 0.2041 12,086,718.335516

21.566520 0.204 14,690,919.600113

21.569922 0.2041 18,305,157.335601

21.573323 0.2041 22,889,596.180846

21.576723 0.204 29,444,496.204862

21.580125 0.2041 37,073,848.939634

21.583527 0.2041 45,612,881.861241

21.586927 0.204 55,286,657.456099

21.590328 0.2041 65,205,209.998707

21.593730 0.2041 74,298,695.721719

21.597130 0.204 82,184,724.992584

21.600532 0.2041 90,296,517.822740

21.603933 0.2041 96,423,403.342931

21.607333 0.204 97,955,085.931183

21.610735 0.2041 97,159,701.686572

21.614137 0.2041 94,284,938.142586

21.617537 0.204 86,195,340.076545

21.620938 0.2041 76,491,312.371493

21.624340 0.2041 67,968,880.015477

21.627740 0.204 58,856,659.866462

21.631142 0.2041 49,321,365.596493

21.634543 0.2041 39,536,361.172517

21.637943 0.204 31,623,361.649287

21.641345 0.2041 25,692,986.338687

21.644747 0.2041 20,563,142.159076

21.648147 0.204 16,951,009.672501

21.651548 0.2041 14,545,589.621488

21.654950 0.2041 12,788,143.370258

21.658350 0.204 11,394,904.207516

21.661752 0.2041 10,421,542.447224

21.665153 0.2041 9,894,117.717555

21.668553 0.204 9,523,445.809912

21.671955 0.2041 9,234,339.717389

21.675357 0.2041 9,015,617.637423

21.678757 0.204 8,941,767.337450

21.682158 0.2041 9,040,685.006730

21.685560 0.2041 9,045,137.486562

21.688960 0.204 8,924,625.311010

21.692362 0.2041 8,925,814.363702

21.695763 0.2041 9,035,044.223027

21.699163 0.204 8,919,146.179154

21.702565 0.2041 8,915,315.846315

21.705967 0.2041 9,131,129.261430

21.709367 0.204 8,842,685.805725

21.712768 0.2041 8,731,065.765055

21.716170 0.2041 8,804,129.331943

21.719570 0.204 8,523,936.236972

21.722972 0.2041 8,390,631.326062

21.726373 0.2041 8,175,974.936198

21.729773 0.204 7,913,062.250013

21.733175 0.2041 7,834,759.028589

21.736577 0.2041 7,808,939.475761

21.739977 0.204 7,838,854.755828

21.743378 0.2041 7,843,623.179650

21.746780 0.2041 7,751,881.943437

21.750182 0.2041 7,618,911.477064

21.753582 0.204 7,572,871.167802

21.756983 0.2041 7,673,167.953190

21.760385 0.2041 7,845,933.068557

21.763785 0.204 8,020,296.219897

21.767187 0.2041 8,005,226.161373

21.770588 0.2041 7,835,876.792232

21.773988 0.204 7,831,109.506252

21.777390 0.2041 7,914,630.672228

21.780792 0.2041 7,854,007.441431

21.784192 0.204 7,918,627.360563

21.787593 0.2041 8,072,970.357575

21.790995 0.2041 7,957,952.702616

21.794395 0.204 7,939,125.915289

21.797797 0.2041 7,914,694.186628

21.801198 0.2041 7,897,386.661243

21.804598 0.204 7,967,343.755126

21.808000 0.2041 8,038,634.988354

21.811402 0.2041 8,145,657.004659

21.814802 0.204 8,008,743.453473

21.818203 0.2041 8,023,516.615478

21.821605 0.2041 8,121,317.085081

21.825005 0.204 7,969,084.171195

21.828407 0.2041 7,903,393.524504

21.831808 0.2041 7,869,813.015446

21.835208 0.204 7,848,343.608616

21.838610 0.2041 7,771,278.716419

21.842012 0.2041 7,752,940.173621

21.845412 0.204 7,902,987.944721

21.848813 0.2041 7,746,959.223118

21.852215 0.2041 7,615,276.155349

21.855615 0.204 7,737,706.586512

21.859017 0.2041 7,589,500.139321

21.862418 0.2041 7,283,976.093621

21.865818 0.204 7,444,304.137256

21.869220 0.2041 7,629,668.092254

21.872622 0.2041 7,485,506.324133

21.876022 0.204 7,581,338.690698

21.879423 0.2041 7,599,506.524618

21.882825 0.2041 7,448,044.874050

21.886225 0.204 7,575,121.198595

21.889627 0.2041 7,543,976.133964

21.893028 0.2041 7,295,855.635064

21.896428 0.204 7,431,301.601553

21.899830 0.2041 7,524,497.663851

21.903232 0.2041 7,310,923.851711

21.906632 0.204 7,315,151.080050

21.910033 0.2041 7,528,134.905082

21.913435 0.2041 7,620,651.871763

21.916835 0.204 7,485,363.121179

21.920237 0.2041 7,562,102.474006

21.923638 0.2041 7,655,291.858917

21.927038 0.204 7,488,096.216688

21.930440 0.2041 7,516,184.231441

21.933842 0.2041 7,652,867.853713

21.937242 0.204 7,661,151.535796

21.940643 0.2041 7,565,541.291568

21.944045 0.2041 7,518,387.876197

21.947445 0.204 7,532,979.833659

21.950847 0.2041 7,446,172.314654

21.954248 0.2041 7,357,182.817481

21.957648 0.204 7,508,049.789916

21.961050 0.2041 7,633,584.720956

21.964452 0.2041 7,442,419.393495

21.967852 0.204 7,444,835.644449

21.971253 0.2041 7,655,455.972175

21.974655 0.2041 7,485,251.885201

21.978055 0.204 7,314,183.202937

21.981457 0.2041 7,479,172.230438

21.984858 0.2041 7,530,083.221971

21.988258 0.204 7,451,783.872483

21.991660 0.2041 7,540,234.156306

21.995062 0.2041 7,624,473.478094

21.998462 0.204 7,504,745.918025

22.001863 0.2041 7,465,044.187874

22.005265 0.2041 7,587,892.218940

22.008665 0.204 7,559,260.227003

22.012067 0.2041 7,468,068.354352

22.015468 0.2041 7,425,499.534872

22.018868 0.204 7,377,664.619728

22.022270 0.2041 7,387,776.263579

22.025672 0.2041 7,415,800.401471

22.029072 0.204 7,344,118.170763

22.032473 0.2041 7,453,817.067384

22.035875 0.2041 7,703,689.491299

22.039275 0.204 7,590,505.416573

22.042677 0.2041 7,412,760.309174

22.046078 0.2041 7,623,244.041936

22.049478 0.204 7,601,234.105002

22.052880 0.2041 7,351,021.158452

22.056282 0.2041 7,467,707.498419

22.059682 0.204 7,501,609.398876

22.063083 0.2041 7,562,253.679386

22.066485 0.2041 7,670,780.699130

22.069885 0.204 7,498,165.886772

22.073287 0.2041 7,548,345.348961

22.076688 0.2041 7,703,151.025543

22.080088 0.204 7,531,512.619471

22.083490 0.2041 7,438,622.129050

22.086892 0.2041 7,463,043.069494

22.090293 0.2041 7,480,184.854718

22.093693 0.204 7,523,506.111064

22.097095 0.2041 7,519,839.161331

22.100497 0.2041 7,535,428.224598

22.103897 0.204 7,574,825.693473

22.107298 0.2041 7,605,494.868623

22.110700 0.2041 7,751,197.276755

22.114100 0.204 7,857,726.982199

22.117502 0.2041 7,777,742.491629

22.120903 0.2041 7,945,576.570100

22.124303 0.204 8,140,236.650997

22.127705 0.2041 8,028,329.254013

22.131107 0.2041 7,915,237.202473

22.134507 0.204 7,958,744.497357

22.137908 0.2041 8,011,213.199774

22.141310 0.2041 8,078,015.495353

22.144710 0.204 8,051,172.132490

22.148112 0.2041 7,815,396.196053

22.151513 0.2041 7,739,425.034562

22.154913 0.204 7,647,828.573623

22.158315 0.2041 7,354,917.071588

22.161717 0.2041 7,437,891.108556

22.165117 0.204 7,686,974.502561

22.168518 0.2041 7,573,214.123383

22.171920 0.2041 7,612,131.675471

22.175320 0.204 7,585,235.989400

22.178722 0.2041 7,227,691.943752

22.182123 0.2041 7,332,618.803004

22.185523 0.204 7,522,211.935607

22.188925 0.2041 7,505,630.700951

22.192327 0.2041 7,426,377.315353

22.195727 0.204 7,105,142.118511

22.199128 0.2041 7,330,309.000010

22.202530 0.2041 7,408,669.833917

22.205930 0.204 7,152,106.291690

22.209332 0.2041 7,412,942.627474

22.212733 0.2041 7,411,215.999161

22.216133 0.204 7,199,475.129084

22.219535 0.2041 7,157,812.226585

22.222937 0.2041 7,267,445.893187

22.226337 0.204 7,309,834.222148

22.229738 0.2041 7,121,204.170982

22.233140 0.2041 7,166,865.974133

22.236540 0.204 7,315,678.562669

22.239942 0.2041 7,341,778.431446

22.243343 0.2041 7,352,008.221318

22.246743 0.204 7,266,254.405509

22.250145 0.2041 7,220,314.187412

22.253547 0.2041 7,274,733.152513

22.256947 0.204 7,154,688.424242

22.260348 0.2041 7,042,849.526226

22.263750 0.2041 7,243,545.332569

22.267150 0.204 7,264,623.342658

22.270552 0.2041 7,135,618.293584

22.273953 0.2041 7,221,118.976154

22.277353 0.204 7,153,291.511479

22.280755 0.2041 7,031,768.749027

22.284157 0.2041 7,190,939.724707

22.287557 0.204 7,447,565.771875

22.290958 0.2041 7,308,333.707460

22.294360 0.2041 7,312,089.672769

22.297760 0.204 7,567,075.631399

22.301162 0.2041 7,425,129.368525

22.304563 0.2041 7,425,270.671032

22.307963 0.204 7,402,383.860884

22.311365 0.2041 7,439,298.679686

22.314767 0.2041 7,673,409.738678

22.318167 0.204 7,581,355.127304

22.321568 0.2041 7,639,861.183742

22.324970 0.2041 7,828,386.463427

22.328370 0.204 7,724,592.447996

22.331772 0.2041 7,826,984.510069

22.335173 0.2041 8,035,511.730261

22.338573 0.204 8,106,374.359620

22.341975 0.2041 8,199,247.791405

22.345377 0.2041 8,279,203.792346

22.348777 0.204 8,397,234.146218

22.352178 0.2041 8,441,497.429988

22.355580 0.2041 8,472,730.982362

22.358980 0.204 8,422,017.379081

22.362382 0.2041 8,388,817.290374

22.365783 0.2041 8,568,097.898538

22.369183 0.204 8,547,515.973769

22.372585 0.2041 8,435,024.382463

22.375987 0.2041 8,403,240.825425

22.379387 0.204 8,331,633.939285

22.382788 0.2041 8,425,868.197360

22.386190 0.2041 8,557,626.657035

22.389590 0.204 8,689,400.531028

22.392992 0.2041 8,930,101.311467

22.396393 0.2041 9,047,013.533193

22.399793 0.204 9,204,544.563712

22.403195 0.2041 9,555,391.046270

22.406597 0.2041 9,904,903.536008

22.409997 0.204 10,380,078.676881

22.413398 0.2041 10,597,093.391915

22.416800 0.2041 10,670,504.299402

22.420200 0.204 11,235,924.482195

22.423602 0.2041 11,814,754.979300

22.427003 0.2041 12,032,218.523299

22.430403 0.204 12,189,899.182981

22.433805 0.2041 12,482,073.736017

22.437207 0.2041 12,425,773.009893

22.440607 0.204 12,170,480.647106

22.444008 0.2041 12,181,315.113768

22.447410 0.2041 11,924,002.498977

22.450810 0.204 11,452,502.659563

22.454212 0.2041 11,031,942.628810

22.457613 0.2041 10,501,542.694309

22.461015 0.2041 9,958,496.944619

22.464415 0.204 9,705,514.594037

22.467817 0.2041 9,463,281.275056

22.471218 0.2041 9,100,191.331207

22.474618 0.204 8,872,205.603203

22.478020 0.2041 8,667,972.305616

22.481422 0.2041 8,649,246.056162

22.484822 0.204 8,615,525.213549

22.488223 0.2041 8,452,544.326473

22.491625 0.2041 8,464,611.495947

22.495025 0.204 8,565,049.251398

22.498427 0.2041 8,549,735.173502

22.501828 0.2041 8,332,547.737303

22.505228 0.204 8,154,619.019040

22.508630 0.2041 8,195,742.714067

22.512032 0.2041 8,167,772.371954

22.515432 0.204 7,951,712.930642

22.518833 0.2041 7,836,355.373923

22.522235 0.2041 7,925,562.726546

22.525635 0.204 8,067,473.573545

22.529037 0.2041 8,076,966.439826

22.532438 0.2041 7,735,359.923401

22.535838 0.204 7,592,138.313251

22.539240 0.2041 7,685,412.843197

22.542642 0.2041 7,630,082.269871

22.546042 0.204 7,687,796.077906

22.549443 0.2041 7,684,887.925038

22.552845 0.2041 7,623,664.106468

22.556245 0.204 7,471,756.502066

22.559647 0.2041 7,501,333.323085

22.563048 0.2041 7,742,411.931749

22.566448 0.204 7,584,995.979674

22.569850 0.2041 7,443,838.266922

22.573252 0.2041 7,482,227.718926

22.576652 0.204 7,481,324.416398

22.580053 0.2041 7,578,387.916529

22.583455 0.2041 7,547,224.591059

22.586855 0.204 7,582,600.017057

22.590257 0.2041 7,708,887.291904

22.593658 0.2041 7,650,371.528831

22.597058 0.204 7,602,349.423782

22.600460 0.2041 7,406,522.002501

22.603862 0.2041 7,433,331.876734

22.607262 0.204 7,965,067.119984

22.610663 0.2041 8,059,348.981687

22.614065 0.2041 7,884,497.292736

22.617465 0.204 8,151,807.871171

22.620867 0.2041 8,350,660.814764

22.624268 0.2041 8,452,347.372473

22.627668 0.204 8,329,854.159701

22.631070 0.2041 8,177,115.697915

22.634472 0.2041 8,263,016.135865

22.637872 0.204 8,211,206.133780

22.641273 0.2041 8,298,784.985773

22.644675 0.2041 8,295,888.530082

22.648075 0.204 8,263,344.448030

22.651477 0.2041 8,295,621.205536

22.654878 0.2041 8,191,538.144327

22.658278 0.204 8,402,555.755060

22.661680 0.2041 8,435,763.484124

22.665082 0.2041 8,027,508.798987

22.668482 0.204 8,011,981.087154

22.671883 0.2041 8,022,488.588207

22.675285 0.2041 7,781,966.171548

22.678685 0.204 7,858,902.091668

22.682087 0.2041 7,808,380.774035

22.685488 0.2041 7,667,335.215870

22.688888 0.204 7,816,387.053499

22.692290 0.2041 7,717,572.953618

22.695692 0.2041 7,652,383.458795

22.699092 0.204 7,621,771.173065

22.702493 0.2041 7,518,585.945113

22.705895 0.2041 7,551,594.369450

22.709295 0.204 7,361,037.753929

22.712697 0.2041 7,433,208.259921

22.716098 0.2041 7,593,335.223170

22.719498 0.204 7,379,792.057393

22.722900 0.2041 7,301,737.977096

22.726302 0.2041 7,443,819.225115

22.729702 0.204 7,577,144.585633

22.733103 0.2041 7,417,815.187284

22.736505 0.2041 7,253,383.009646

22.739905 0.204 7,430,691.307369

22.743307 0.2041 7,424,568.908593

22.746708 0.2041 7,172,978.738778

22.750108 0.204 7,239,790.872231

22.753510 0.2041 7,488,142.577427

22.756912 0.2041 7,469,973.838334

22.760312 0.204 7,256,101.104020

22.763713 0.2041 7,109,225.750833

22.767115 0.2041 7,133,337.414124

22.770515 0.204 7,182,984.552437

22.773917 0.2041 7,175,705.875534

22.777318 0.2041 7,077,562.433296

22.780718 0.204 7,068,884.456841

22.784120 0.2041 7,298,973.926212

22.787522 0.2041 7,281,923.064363

22.790922 0.204 7,239,405.400221

22.794323 0.2041 7,276,612.448859

22.797725 0.2041 7,257,802.549454

22.801125 0.204 7,182,842.378528

22.804527 0.2041 7,055,592.588437

22.807928 0.2041 7,204,312.799105

22.811330 0.2041 7,110,846.646516

22.814730 0.204 7,102,565.647307

22.818132 0.2041 7,414,999.245974

22.821533 0.2041 7,346,885.275470

22.824933 0.204 7,274,073.325712

22.828335 0.2041 7,337,468.345417

22.831737 0.2041 7,154,965.252933

22.835137 0.204 7,131,654.151526

22.838538 0.2041 7,211,513.942254

22.841940 0.2041 7,126,806.472813

22.845340 0.204 7,342,766.821771

22.848742 0.2041 7,461,925.812063

22.852143 0.2041 7,348,517.002896

22.855543 0.204 7,194,587.247780

22.858945 0.2041 7,200,992.608261

22.862347 0.2041 7,324,404.363299

22.865747 0.204 7,211,233.087129

22.869148 0.2041 7,166,654.092062

22.872550 0.2041 7,228,472.076268

22.875950 0.204 7,245,075.469714

22.879352 0.2041 7,304,541.031346

22.882753 0.2041 7,452,223.922678

22.886153 0.204 7,323,612.218670

22.889555 0.2041 7,087,778.508558

22.892957 0.2041 7,258,659.078533

22.896357 0.204 7,354,658.150911

22.899758 0.2041 7,198,827.565985

22.903160 0.2041 7,192,554.324581

22.906560 0.204 7,247,065.800446

22.909962 0.2041 7,239,374.919498

22.913363 0.2041 7,066,598.468526

22.916763 0.204 7,059,424.718358

22.920165 0.2041 7,246,728.855511

22.923567 0.2041 7,151,295.350927

22.926967 0.204 7,205,225.791483

22.930368 0.2041 7,269,236.103358

22.933770 0.2041 7,241,404.093140

22.937170 0.204 7,263,724.591540

22.940572 0.2041 7,060,038.134460

22.943973 0.2041 7,181,818.081855

22.947373 0.204 7,362,667.318849

22.950775 0.2041 7,307,846.257435

22.954177 0.2041 7,391,395.671187

22.957577 0.204 7,304,017.951573

22.960978 0.2041 7,318,734.817450

22.964380 0.2041 7,427,901.858794

22.967780 0.204 7,304,845.794202

22.971182 0.2041 7,424,546.659209

22.974583 0.2041 7,531,785.816108

22.977983 0.204 7,502,575.915872

22.981385 0.2041 7,511,495.801512

22.984787 0.2041 7,374,273.110569

22.988187 0.204 7,454,174.778762

22.991588 0.2041 7,425,652.262542

22.994990 0.2041 7,367,507.346138

22.998390 0.204 7,382,803.851886

23.001792 0.2041 7,171,615.564284

23.005193 0.2041 7,149,596.845109

23.008593 0.204 7,238,936.818443

23.011995 0.2041 7,339,150.971402

23.015397 0.2041 7,356,819.243757

23.018797 0.204 7,430,963.772163

23.022198 0.2041 7,541,776.264976

23.025600 0.2041 7,565,280.336663

23.029000 0.204 7,578,913.764943

23.032402 0.2041 7,440,968.445551

23.035803 0.2041 7,444,599.732866

23.039203 0.204 7,566,105.769417

23.042605 0.2041 7,548,371.949975

23.046007 0.2041 7,603,600.661599

23.049407 0.204 7,640,865.317862

23.052808 0.2041 7,575,037.302402

23.056210 0.2041 7,519,704.228181

23.059610 0.204 7,461,520.849063

23.063012 0.2041 7,591,208.158035

23.066413 0.2041 7,499,461.168905

23.069813 0.204 7,378,065.582488

23.073215 0.2041 7,555,810.753164

23.076617 0.2041 7,353,128.109287

23.080017 0.204 7,437,974.310486

23.083418 0.2041 7,883,824.289627

23.086820 0.2041 7,749,565.578153

23.090220 0.204 7,895,910.563830

23.093622 0.2041 8,428,378.525577

23.097023 0.2041 8,640,705.472733

23.100423 0.204 8,901,116.197591

23.103825 0.2041 9,401,792.254050

23.107227 0.2041 10,228,590.946893

23.110627 0.204 10,625,828.608514

23.114028 0.2041 11,011,655.057942

23.117430 0.2041 11,973,309.878111

23.120830 0.204 12,534,427.276809

23.124232 0.2041 12,883,088.831846

23.127633 0.2041 13,112,772.752811

23.131033 0.204 13,395,987.127598

23.134435 0.2041 13,502,436.859472

23.137837 0.2041 13,168,620.272259

23.141237 0.204 12,899,239.937487

23.144638 0.2041 12,305,596.836529

23.148040 0.2041 11,695,880.494337

23.151440 0.204 11,296,207.288146

23.154842 0.2041 10,607,306.707184

23.158243 0.2041 10,078,263.414476

23.161643 0.204 9,748,131.036198

23.165045 0.2041 9,385,844.729137

23.168447 0.2041 9,202,302.214043

23.171848 0.2041 8,934,999.292050

23.175248 0.204 8,785,313.964115

23.178650 0.2041 8,609,647.232807

23.182052 0.2041 8,434,842.136737

23.185452 0.204 8,487,797.118663

23.188853 0.2041 8,318,593.221170

23.192255 0.2041 8,407,437.288305

23.195655 0.204 8,425,758.922738

23.199057 0.2041 8,164,377.869938

23.202458 0.2041 8,183,729.623479

23.205858 0.204 8,115,069.481472

23.209260 0.2041 8,076,411.440884

23.212662 0.2041 8,076,309.076426

23.216062 0.204 7,850,490.825230

23.219463 0.2041 7,903,541.948131

23.222865 0.2041 8,130,721.986808

23.226265 0.204 7,958,517.344322

23.229667 0.2041 7,937,605.907768

23.233068 0.2041 8,087,982.461741

23.236468 0.204 8,106,184.934655

23.239870 0.2041 8,109,846.004608

23.243272 0.2041 7,953,780.954862

23.246672 0.204 7,947,997.320332

23.250073 0.2041 7,970,899.837683

23.253475 0.2041 7,998,589.031839

23.256875 0.204 7,959,550.223625

23.260277 0.2041 7,723,587.297589

23.263678 0.2041 7,750,095.783441

23.267078 0.204 7,732,056.157473

23.270480 0.2041 7,577,530.007616

23.273882 0.2041 7,634,167.440369

23.277282 0.204 7,525,306.335817

23.280683 0.2041 7,464,734.325715

23.284085 0.2041 7,593,420.334027

23.287485 0.204 7,507,622.669601

23.290887 0.2041 7,412,189.325125

23.294288 0.2041 7,444,707.396511

23.297688 0.204 7,481,389.934603

23.301090 0.2041 7,396,547.847300

23.304492 0.2041 7,303,187.989807

23.307892 0.204 7,388,037.417719

23.311293 0.2041 7,509,490.754571

23.314695 0.2041 7,369,455.303415

23.318095 0.204 7,355,095.553160

23.321497 0.2041 7,490,620.739174

23.324898 0.2041 7,398,019.620126

23.328298 0.204 7,335,891.137313

23.331700 0.2041 7,360,384.120464

23.335102 0.2041 7,385,027.952481

23.338502 0.204 7,449,727.759942

23.341903 0.2041 7,569,701.477924

23.345305 0.2041 7,534,988.567093

23.348705 0.204 7,362,144.141331

23.352107 0.2041 7,299,809.710078

23.355508 0.2041 7,266,737.649024

23.358908 0.204 7,176,135.596251

23.362310 0.2041 7,221,955.770863

23.365712 0.2041 7,324,178.802532

23.369112 0.204 7,247,180.773624

23.372513 0.2041 7,147,203.130676

23.375915 0.2041 7,111,181.274824

23.379315 0.204 7,142,578.512878

23.382717 0.2041 7,280,134.315122

23.386118 0.2041 7,230,576.121964

23.389518 0.204 7,108,577.838227

23.392920 0.2041 7,249,428.536760

23.396322 0.2041 7,243,211.203839

23.399722 0.204 7,073,397.808186

23.403123 0.2041 6,977,839.227072

23.406525 0.2041 7,046,103.042324

23.409925 0.204 7,158,734.379448

23.413327 0.2041 7,155,049.068961

23.416728 0.2041 7,056,500.366901

23.420128 0.204 6,979,075.898684

23.423530 0.2041 7,252,615.353876

23.426932 0.2041 7,286,596.240600

23.430332 0.204 7,180,304.292201

23.433733 0.2041 7,283,920.844963

23.437135 0.2041 7,208,129.858335

23.440535 0.204 7,318,792.588604

23.443937 0.2041 7,383,892.580180

23.447338 0.2041 7,222,450.636195

23.450738 0.204 7,171,393.346569

23.454140 0.2041 7,199,282.426739

23.457542 0.2041 7,368,959.734722

23.460942 0.204 7,319,456.577958

23.464343 0.2041 6,970,268.463864

23.467745 0.2041 6,883,203.178336

23.471145 0.204 7,057,270.382576

23.474547 0.2041 7,153,217.256416

23.477948 0.2041 7,060,544.219991

23.481348 0.204 7,023,657.544917

23.484750 0.2041 6,987,548.156826

23.488152 0.2041 7,066,691.999789

23.491552 0.204 7,256,626.238929

23.494953 0.2041 7,180,642.569995

23.498355 0.2041 7,088,067.936048

23.501755 0.204 7,093,041.715890

23.505157 0.2041 7,111,008.625880

23.508558 0.2041 7,169,207.509521

23.511960 0.2041 7,210,464.355149

23.515360 0.204 7,136,259.514114

23.518762 0.2041 7,117,095.856226

23.522163 0.2041 7,211,308.900951

23.525563 0.204 7,237,503.060166

23.528965 0.2041 6,996,261.294315

23.532367 0.2041 7,024,363.557597

23.535767 0.204 7,357,660.158152

23.539168 0.2041 7,289,926.029246

23.542570 0.2041 7,305,105.168490

23.545970 0.204 7,340,171.662598

23.549372 0.2041 7,361,499.761594

23.552773 0.2041 7,418,080.392920

23.556173 0.204 7,380,016.473311

23.559575 0.2041 7,419,222.194172

23.562977 0.2041 7,276,332.765972

23.566377 0.204 7,311,677.157849

23.569778 0.2041 7,283,863.288171

23.573180 0.2041 7,226,596.062207

23.576580 0.204 7,421,126.446145

23.579982 0.2041 7,329,236.160077

23.583383 0.2041 7,290,210.142823

23.586783 0.204 7,311,892.743965

23.590185 0.2041 7,274,911.232354

23.593587 0.2041 7,452,144.030594

23.596987 0.204 7,430,267.169253

23.600388 0.2041 7,553,641.154030

23.603790 0.2041 7,935,165.709457

23.607190 0.204 7,819,563.180818

23.610592 0.2041 7,691,682.193454

23.613993 0.2041 7,827,340.868911

23.617393 0.204 7,892,388.424060

23.620795 0.2041 8,099,781.831876

23.624197 0.2041 8,340,925.823745

23.627597 0.204 8,442,735.324569

23.630998 0.2041 8,676,011.335853

23.634400 0.2041 8,926,471.909063

23.637800 0.204 8,931,993.843561

23.641202 0.2041 9,082,574.267164

23.644603 0.2041 9,256,488.600364

23.648003 0.204 9,356,669.202734

23.651405 0.2041 9,489,040.916790

23.654807 0.2041 9,435,189.897490

23.658207 0.204 9,457,907.272781

23.661608 0.2041 9,426,831.160341

23.665010 0.2041 9,349,218.182682

23.668410 0.204 9,382,354.447126

23.671812 0.2041 9,221,197.796226

23.675213 0.2041 9,001,558.516175

23.678613 0.204 8,855,679.583302

23.682015 0.2041 8,657,762.885170

23.685417 0.2041 8,746,409.161152

23.688817 0.204 8,594,617.784552

23.692218 0.2041 8,031,929.523675

23.695620 0.2041 8,124,915.495193

23.699020 0.204 8,195,857.501925

23.702422 0.2041 7,692,929.137020

23.705823 0.2041 7,605,761.761681

23.709223 0.204 7,809,709.584161

23.712625 0.2041 7,633,775.666721

23.716027 0.2041 7,557,177.699236

23.719427 0.204 7,429,099.525699

23.722828 0.2041 7,144,584.929513

23.726230 0.2041 7,294,179.085625

23.729630 0.204 7,349,176.593411

23.733032 0.2041 7,284,123.677221

23.736433 0.2041 7,341,794.164456

23.739833 0.204 7,222,355.337419

23.743235 0.2041 7,164,445.323482

23.746637 0.2041 7,355,051.894048

23.750037 0.204 7,339,665.709868

23.753438 0.2041 7,326,532.710771

23.756840 0.2041 7,374,586.253983

23.760240 0.204 7,164,226.165542

23.763642 0.2041 7,194,539.140021

23.767043 0.2041 7,248,633.058448

23.770443 0.204 7,246,921.651399

23.773845 0.2041 7,274,458.932092

23.777247 0.2041 7,154,334.635709

23.780647 0.204 7,167,271.052236

23.784048 0.2041 7,270,714.274441

23.787450 0.2041 7,346,760.884046

23.790850 0.204 7,211,413.730492

23.794252 0.2041 7,137,208.190762

23.797653 0.2041 7,271,623.474403

23.801053 0.204 7,254,660.292580

23.804455 0.2041 7,236,815.185236

23.807857 0.2041 7,241,717.773706

23.811257 0.204 7,325,679.223946

23.814658 0.2041 7,272,115.506951

23.818060 0.2041 7,107,257.100128

23.821460 0.204 7,129,853.527277

23.824862 0.2041 7,124,699.042668

23.828263 0.2041 7,247,830.042154

23.831663 0.204 7,246,753.868393

23.835065 0.2041 7,031,080.775638

23.838467 0.2041 7,193,489.270198

23.841867 0.204 7,272,757.355513

23.845268 0.2041 7,034,058.883235

23.848670 0.2041 7,075,171.081065

23.852070 0.204 7,262,341.303298

23.855472 0.2041 7,334,606.907907

23.858873 0.2041 7,355,490.543986

23.862273 0.204 7,408,977.261862

23.865675 0.2041 7,370,118.144061

23.869077 0.2041 7,245,032.575038

23.872478 0.2041 7,174,855.499908

23.875878 0.204 7,325,536.911550

23.879280 0.2041 7,493,934.487937

23.882682 0.2041 7,469,962.170581

23.886082 0.204 7,580,064.287591

23.889483 0.2041 7,633,177.052505

23.892885 0.2041 7,524,179.052755

23.896285 0.204 7,442,376.626751

23.899687 0.2041 7,708,793.508797

23.903088 0.2041 7,645,462.121725

23.906488 0.204 7,489,271.007193

23.909890 0.2041 7,849,477.454134

23.913292 0.2041 7,699,659.152596

23.916692 0.204 7,436,892.309862

23.920093 0.2041 7,462,218.835909

23.923495 0.2041 7,617,291.275469

23.926895 0.204 7,667,481.802027

23.930297 0.2041 7,597,248.902850

23.933698 0.2041 7,415,937.589248

23.937098 0.204 7,280,686.124079

23.940500 0.2041 7,278,568.330669

23.943902 0.2041 7,024,912.488402

23.947302 0.204 7,210,099.475880

23.950703 0.2041 7,340,328.005828

23.954105 0.2041 7,134,743.232331

23.957505 0.204 7,274,312.943146

23.960907 0.2041 7,182,178.069975

23.964308 0.2041 6,887,776.224673

23.967708 0.204 7,022,652.193665

23.971110 0.2041 7,292,814.638066

23.974512 0.2041 7,272,168.622275

23.977912 0.204 7,180,705.158674

23.981313 0.2041 7,273,337.823973

23.984715 0.2041 7,293,606.672171

23.988115 0.204 7,216,531.949684

23.991517 0.2041 7,136,490.926035

23.994918 0.2041 7,017,442.100474

23.998318 0.204 7,162,337.929767

24.001720 0.2041 7,369,399.570219

24.005122 0.2041 7,151,718.968328

24.008522 0.204 6,965,776.427850

24.011923 0.2041 7,079,868.550875

24.015325 0.2041 7,026,751.342586

24.018725 0.204 7,063,270.259935

24.022127 0.2041 7,301,167.034526

24.025528 0.2041 7,234,220.305328

24.028928 0.204 7,200,165.590689

24.032330 0.2041 7,027,816.145520

24.035732 0.2041 6,948,661.541282

24.039132 0.204 7,271,616.301786

24.042533 0.2041 7,223,555.680146

24.045935 0.2041 7,300,253.159098

24.049335 0.204 7,254,509.996351

24.052737 0.2041 7,130,661.151105

24.056138 0.2041 7,293,701.733179

24.059538 0.204 7,129,694.179167

24.062940 0.2041 7,271,383.348472

24.066342 0.2041 7,482,199.985616

24.069742 0.204 7,295,366.792093

24.073143 0.2041 7,236,348.135694

24.076545 0.2041 7,281,726.119182

24.079945 0.204 7,327,651.713568

24.083347 0.2041 7,195,175.839009

24.086748 0.2041 7,112,172.139329

24.090148 0.204 7,207,810.261570

24.093550 0.2041 7,177,943.171890

24.096952 0.2041 7,129,820.439260

24.100352 0.204 7,034,360.195266

24.103753 0.2041 7,069,840.002469

24.107155 0.2041 7,228,843.421277

24.110555 0.204 7,305,104.038579

24.113957 0.2041 7,282,198.851352

24.117358 0.2041 7,186,020.617045

24.120758 0.204 7,162,896.922203

24.124160 0.2041 7,159,340.315564

24.127562 0.2041 7,013,318.142812

24.130962 0.204 6,941,186.271520

24.134363 0.2041 7,156,846.299472

24.137765 0.2041 7,312,614.334720

24.141165 0.204 7,218,423.137250

24.144567 0.2041 7,129,245.438847

24.147968 0.2041 7,269,745.108808

24.151368 0.204 7,096,163.479319

24.154770 0.2041 6,938,214.793604

24.158172 0.2041 6,989,497.399861

24.161572 0.204 6,997,806.520502

24.164973 0.2041 7,141,269.572504

24.168375 0.2041 7,012,921.902276

24.171775 0.204 7,032,394.923628

24.175177 0.2041 7,160,114.886422

24.178578 0.2041 6,985,389.391778

24.181978 0.204 6,984,851.706211

24.185380 0.2041 7,189,443.368483

24.188782 0.2041 7,214,548.550402

24.192182 0.204 7,090,576.299126

24.195583 0.2041 7,100,161.975781

24.198985 0.2041 7,176,488.036093

24.202385 0.204 7,096,897.415726

24.205787 0.2041 7,041,914.356069

24.209188 0.2041 7,135,017.772123

24.212590 0.2041 7,238,990.499274

24.215990 0.204 7,241,992.244472

24.219392 0.2041 7,091,129.584103

24.222793 0.2041 7,062,350.052868

24.226193 0.204 7,130,197.689290

24.229595 0.2041 7,043,098.008097

24.232997 0.2041 7,072,622.571059

24.236397 0.204 7,277,989.211897

24.239798 0.2041 7,274,295.794764

24.243200 0.2041 7,066,897.289337

24.246600 0.204 6,982,374.544948

24.250002 0.2041 7,009,571.747678

24.253403 0.2041 6,937,625.315886

24.256803 0.204 6,954,952.728473

24.260205 0.2041 7,243,445.020007

24.263607 0.2041 7,261,302.752196

24.267007 0.204 7,050,954.098645

24.270408 0.2041 7,146,755.602514

24.273810 0.2041 7,302,866.571960

24.277210 0.204 7,219,241.960968

24.280612 0.2041 7,093,409.641790

24.284013 0.2041 7,031,414.888928

24.287413 0.204 6,845,090.696162

24.290815 0.2041 6,947,004.514889

24.294217 0.2041 7,076,820.482588

24.297617 0.204 6,944,460.926343

24.301018 0.2041 7,037,293.946272

24.304420 0.2041 7,097,922.135411

24.307820 0.204 7,207,015.375265

24.311222 0.2041 7,323,288.310437

24.314623 0.2041 7,238,101.460753

24.318023 0.204 7,208,850.915438

24.321425 0.2041 7,256,166.114885

24.324827 0.2041 7,270,826.071113

24.328227 0.204 7,083,197.733750

24.331628 0.2041 7,067,011.648675

24.335030 0.2041 7,317,015.234534

24.338430 0.204 7,295,206.345519

24.341832 0.2041 7,169,221.454859

24.345233 0.2041 7,122,777.105091

24.348633 0.204 7,206,459.112803

24.352035 0.2041 7,371,571.951898

24.355437 0.2041 7,326,414.662615

24.358837 0.204 7,243,926.170899

24.362238 0.2041 7,207,401.541066

24.365640 0.2041 7,214,617.512911

24.369040 0.204 7,182,649.868952

24.372442 0.2041 7,113,295.090881

24.375843 0.2041 7,218,950.182976

24.379243 0.204 7,220,674.320178

24.382645 0.2041 7,129,683.555583

24.386047 0.2041 7,209,058.413036

24.389447 0.204 7,200,328.132694

24.392848 0.2041 7,142,210.544675

24.396250 0.2041 7,090,528.294554

24.399650 0.204 7,196,281.789112

24.403052 0.2041 7,534,871.981153

24.406453 0.2041 7,366,323.374540

24.409853 0.204 7,117,827.703399

24.413255 0.2041 7,315,168.158546

24.416657 0.2041 7,262,134.093177

24.420057 0.204 7,119,371.803209

24.423458 0.2041 7,117,387.648049

24.426860 0.2041 7,186,962.689656

24.430260 0.204 7,294,218.708788

24.433662 0.2041 7,139,406.647355

24.437063 0.2041 7,173,310.825851

24.440463 0.204 7,190,538.944163

24.443865 0.2041 7,065,694.596490

24.447267 0.2041 7,244,995.037961

24.450667 0.204 7,264,226.543625

24.454068 0.2041 7,192,279.070157

24.457470 0.2041 7,124,235.793443

24.460870 0.204 7,090,128.600341

24.464272 0.2041 7,132,833.690736

24.467673 0.2041 7,114,497.151444

24.471073 0.204 7,248,496.425286

24.474475 0.2041 7,278,773.828394

24.477877 0.2041 7,117,131.131933

24.481277 0.204 7,109,915.630865

24.484678 0.2041 7,205,827.194242

24.488080 0.2041 7,156,768.149729

24.491480 0.204 7,181,741.678986

24.494882 0.2041 7,334,675.955827

24.498283 0.2041 7,193,762.105817

24.501683 0.204 7,088,776.762033

24.505085 0.2041 7,161,351.548028

24.508487 0.2041 7,096,830.825820

24.511887 0.204 7,002,300.025532

24.515288 0.2041 7,114,473.509029

24.518690 0.2041 7,389,052.781040

24.522090 0.204 7,260,781.498341

24.525492 0.2041 7,161,140.959914

24.528893 0.2041 7,303,180.977815

24.532293 0.204 7,248,228.702619

24.535695 0.2041 7,248,995.922386

24.539097 0.2041 7,205,646.674566

24.542497 0.204 7,180,643.894656

24.545898 0.2041 7,246,705.460740

24.549300 0.2041 7,107,526.785997

24.552700 0.204 7,100,375.637446

24.556102 0.2041 7,231,596.792503

24.559503 0.2041 7,185,882.976237

24.562905 0.2041 7,259,958.198661

24.566305 0.204 7,331,272.124299

24.569707 0.2041 7,135,712.545497

24.573108 0.2041 7,001,569.862403

24.576508 0.204 7,124,414.787687

24.579910 0.2041 7,159,353.656341

24.583312 0.2041 7,051,730.110635

24.586712 0.204 7,077,892.905610

24.590113 0.2041 7,179,605.102305

24.593515 0.2041 7,244,237.901301

24.596915 0.204 7,142,160.672936

24.600317 0.2041 6,999,170.764904

24.603718 0.2041 7,031,022.208410

24.607118 0.204 7,031,682.524805

24.610520 0.2041 7,056,611.937174

24.613922 0.2041 7,043,054.113091

24.617322 0.204 6,885,173.091553

24.620723 0.2041 6,919,877.033594

24.624125 0.2041 7,082,142.976544

24.627525 0.204 7,056,160.806412

24.630927 0.2041 7,072,859.988212

24.634328 0.2041 7,119,347.164411

24.637728 0.204 6,983,900.791906

24.641130 0.2041 7,032,587.110126

24.644532 0.2041 7,012,075.671601

24.647932 0.204 6,982,371.313466

24.651333 0.2041 7,151,547.794959

24.654735 0.2041 7,265,365.241039

24.658135 0.204 7,284,761.517632

24.661537 0.2041 7,152,149.702371

24.664938 0.2041 7,158,526.138006

24.668338 0.204 7,150,952.589882

24.671740 0.2041 7,114,162.133229

24.675142 0.2041 7,195,196.918050

24.678542 0.204 7,196,750.548167

24.681943 0.2041 7,250,732.034894

24.685345 0.2041 7,157,325.839033

24.688745 0.204 6,948,579.033398

24.692147 0.2041 6,881,159.456456

24.695548 0.2041 6,944,244.367876

24.698948 0.204 7,051,691.363085

24.702350 0.2041 7,045,610.259353

24.705752 0.2041 6,993,393.504166

24.709152 0.204 7,003,897.986185

24.712553 0.2041 7,115,677.175070

24.715955 0.2041 7,019,190.759642

24.719355 0.204 6,845,879.328597

24.722757 0.2041 6,971,228.825435

24.726158 0.2041 7,132,463.055496

24.729558 0.204 7,294,853.483834

24.732960 0.2041 7,246,066.150987

24.736362 0.2041 7,135,302.047473

24.739762 0.204 7,282,592.471140

24.743163 0.2041 7,416,790.088203

24.746565 0.2041 7,363,177.265529

24.749965 0.204 7,390,931.395701

24.753367 0.2041 7,639,157.306032

24.756768 0.2041 7,704,515.137472

24.760168 0.204 7,571,082.756763

24.763570 0.2041 7,528,174.021329

24.766972 0.2041 7,633,589.547886

24.770372 0.204 7,726,698.196807

24.773773 0.2041 7,877,139.661747

24.777175 0.2041 7,908,685.866621

24.780575 0.204 7,809,993.337304

24.783977 0.2041 7,961,676.346289

24.787378 0.2041 8,034,353.905336

24.790778 0.204 7,988,025.422819

24.794180 0.2041 7,827,727.581060

24.797582 0.2041 7,755,336.282622

24.800982 0.204 8,057,337.587994

24.804383 0.2041 8,024,830.957891

24.807785 0.2041 7,900,606.104920

24.811185 0.204 8,065,108.196913

24.814587 0.2041 8,115,577.013083

24.817988 0.2041 7,868,418.513483

24.821388 0.204 7,675,025.236667

24.824790 0.2041 7,880,816.614548

24.828192 0.2041 7,844,818.746856

24.831592 0.204 7,533,049.623322

24.834993 0.2041 7,376,271.118743

24.838395 0.2041 7,455,889.289983

24.841795 0.204 7,631,436.765216

24.845197 0.2041 7,684,483.440331

24.848598 0.2041 7,595,974.105230

24.851998 0.204 7,411,414.398820

24.855400 0.2041 7,250,064.528906

24.858802 0.2041 7,136,249.455580

24.862202 0.204 7,191,329.591044

24.865603 0.2041 7,244,023.408869

24.869005 0.2041 7,112,809.591839

24.872405 0.204 7,156,466.816173

24.875807 0.2041 7,347,002.312413

24.879208 0.2041 7,440,669.589049

24.882608 0.204 7,238,451.206054

24.886010 0.2041 7,068,674.600195

24.889412 0.2041 7,293,757.497259

24.892812 0.204 7,145,983.117527

24.896213 0.2041 7,019,555.201605

24.899615 0.2041 7,169,582.907397

24.903015 0.204 7,159,485.210729

24.906417 0.2041 7,292,376.402504

24.909818 0.2041 7,123,053.357356

24.913220 0.2041 6,972,302.864070

24.916620 0.204 7,139,168.900451

24.920022 0.2041 7,063,599.456064

24.923423 0.2041 7,015,827.135335

24.926823 0.204 7,017,087.206100

24.930225 0.2041 7,117,174.413732

24.933627 0.2041 7,186,303.019672

24.937027 0.204 7,147,659.835623

24.940428 0.2041 7,235,868.347933

24.943830 0.2041 7,325,729.817044

24.947230 0.204 7,430,606.640770

24.950632 0.2041 7,566,062.466817

24.954033 0.2041 7,567,975.785654

24.957433 0.204 7,629,880.958839

24.960835 0.2041 7,684,975.582014

24.964237 0.2041 7,601,684.515151

24.967637 0.204 7,919,118.364646

24.971038 0.2041 7,972,919.144178

24.974440 0.2041 7,663,629.834870

24.977840 0.204 7,739,869.659842

24.981242 0.2041 7,927,070.094978

24.984643 0.2041 7,965,369.915385

24.988043 0.204 7,825,458.386712

24.991445 0.2041 7,819,378.231398

24.994847 0.2041 7,892,342.920359

24.998247 0.204 7,770,281.728725

25.001648 0.2041 7,636,777.765242

25.005050 0.2041 7,607,758.965244

25.008450 0.204 7,608,483.252290

25.011852 0.2041 7,581,338.829479

25.015253 0.2041 7,587,126.120024

25.018653 0.204 7,567,176.535913

25.022055 0.2041 7,519,548.679270

25.025457 0.2041 7,381,594.839893

25.028857 0.204 7,294,670.838730

25.032258 0.2041 7,473,257.958526

25.035660 0.2041 7,541,096.692995

25.039060 0.204 7,572,947.527183

25.042462 0.2041 7,572,109.372269

25.045863 0.2041 7,529,484.840422

25.049263 0.204 7,304,145.029941

25.052665 0.2041 7,105,853.718457

25.056067 0.2041 7,330,397.335517

25.059467 0.204 7,484,855.160502

25.062868 0.2041 7,429,444.917067

25.066270 0.2041 7,269,681.921825

25.069670 0.204 7,299,209.184370

25.073072 0.2041 7,363,307.962554

25.076473 0.2041 7,339,591.321629

25.079873 0.204 7,389,800.690271

25.083275 0.2041 7,282,547.182957

25.086677 0.2041 7,115,674.220436

25.090077 0.204 7,143,294.367686

25.093478 0.2041 7,180,424.997220

25.096880 0.2041 7,061,330.860894

25.100280 0.204 7,076,962.722257

25.103682 0.2041 7,143,365.770693

25.107083 0.2041 7,080,630.413795

25.110483 0.204 7,118,573.861529

25.113885 0.2041 7,124,329.162880

25.117287 0.2041 7,103,122.195079

25.120687 0.204 6,968,214.936656

25.124088 0.2041 6,894,627.017938

25.127490 0.2041 7,101,283.798723

25.130890 0.204 7,156,481.136799

25.134292 0.2041 7,058,951.769014

25.137693 0.2041 6,948,761.406451

25.141093 0.204 6,956,828.051324

25.144495 0.2041 6,971,975.875211

25.147897 0.2041 6,986,384.328854

25.151297 0.204 7,142,584.557427

25.154698 0.2041 7,127,584.897486

25.158100 0.2041 6,938,888.499559

25.161500 0.204 7,013,097.997701

25.164902 0.2041 7,086,282.788588

25.168303 0.2041 6,999,935.601165

25.171703 0.204 7,043,505.683091

25.175105 0.2041 6,971,854.722608

25.178507 0.2041 7,003,015.087325

25.181907 0.204 7,166,447.176228

25.185308 0.2041 7,121,909.699144

25.188710 0.2041 7,187,083.292942

25.192110 0.204 7,217,333.510620

25.195512 0.2041 7,350,846.600002

25.198913 0.2041 7,542,963.436662

25.202313 0.204 7,322,840.547879

25.205715 0.2041 7,338,981.743126

25.209117 0.2041 7,624,719.467247

25.212517 0.204 7,609,440.620937

25.215918 0.2041 7,453,457.827512

25.219320 0.2041 7,469,189.403096

25.222720 0.204 7,550,309.963918

25.226122 0.2041 7,548,496.183438

25.229523 0.2041 7,468,971.525128

25.232923 0.204 7,304,170.359152

25.236325 0.2041 7,301,870.813319

25.239727 0.2041 7,351,282.925016

25.243127 0.204 7,193,226.479080

25.246528 0.2041 7,148,730.840338

25.249930 0.2041 7,211,190.580368

25.253332 0.2041 7,246,186.863897

25.256732 0.204 7,165,632.643348

25.260133 0.2041 7,068,081.205650

25.263535 0.2041 7,155,537.986605

25.266935 0.204 7,085,678.246002

25.270337 0.2041 7,134,183.817696

25.273738 0.2041 7,179,120.555487

25.277138 0.204 7,144,146.143617

25.280540 0.2041 7,298,111.910155

25.283942 0.2041 7,210,889.365678

25.287342 0.204 7,147,147.579720

25.290743 0.2041 7,191,414.676975

25.294145 0.2041 7,098,255.884681

25.297545 0.204 7,105,241.268786

25.300947 0.2041 7,244,538.466608

25.304348 0.2041 7,244,541.446844

25.307748 0.204 7,096,165.522903

25.311150 0.2041 7,185,118.622241

25.314552 0.2041 7,333,711.318397

25.317952 0.204 7,137,991.191353

25.321353 0.2041 7,103,960.805048

25.324755 0.2041 7,259,004.899474

25.328155 0.204 7,164,013.711455

25.331557 0.2041 7,138,610.036869

25.334958 0.2041 7,137,417.392691

25.338358 0.204 7,111,083.702204

25.341760 0.2041 7,274,046.618020

25.345162 0.2041 7,116,420.223386

25.348562 0.204 6,982,543.220685

25.351963 0.2041 7,258,129.669578

25.355365 0.2041 7,124,156.053159

25.358765 0.204 7,082,021.071428

25.362167 0.2041 7,275,378.182967

25.365568 0.2041 7,207,025.365667

25.368968 0.204 7,444,351.254395

25.372370 0.2041 7,601,905.170859

25.375772 0.2041 7,313,710.838653

25.379172 0.204 7,329,150.042693

25.382573 0.2041 7,592,638.642368

25.385975 0.2041 7,675,660.737319

25.389375 0.204 7,668,468.330589

25.392777 0.2041 7,727,008.351939

25.396178 0.2041 8,023,655.450195

25.399578 0.204 8,289,303.160866

25.402980 0.2041 8,396,327.505841

25.406382 0.2041 8,440,129.491202

25.409782 0.204 8,721,131.212111

25.413183 0.2041 9,140,576.923933

25.416585 0.2041 9,214,436.684263

25.419985 0.204 9,331,973.564162

25.423387 0.2041 9,401,593.535414

25.426788 0.2041 9,520,939.615471

25.430188 0.204 9,706,054.333329

25.433590 0.2041 9,791,872.219911

25.436992 0.2041 9,987,645.003318

25.440392 0.204 9,911,577.929497

25.443793 0.2041 9,841,987.099150

25.447195 0.2041 9,826,513.498664

25.450595 0.204 9,510,368.604947

25.453997 0.2041 9,353,072.578790

25.457398 0.2041 9,298,088.977658

25.460798 0.204 9,060,821.897770

25.464200 0.2041 8,906,922.763083

25.467602 0.2041 8,776,851.860914

25.471002 0.204 8,689,794.205187

25.474403 0.2041 8,794,106.017827

25.477805 0.2041 8,808,837.163062

25.481205 0.204 8,638,743.756743

25.484607 0.2041 8,713,263.521090

25.488008 0.2041 8,688,476.221055

25.491408 0.204 8,493,412.155375

25.494810 0.2041 8,578,438.221437

25.498212 0.2041 8,678,238.414038

25.501612 0.204 8,605,629.487615

25.505013 0.2041 8,500,226.074408

25.508415 0.2041 8,299,784.581823

25.511815 0.204 8,114,697.460824

25.515217 0.2041 8,088,756.747484

25.518618 0.2041 7,988,345.887211

25.522018 0.204 7,850,477.710223

25.525420 0.2041 7,779,969.770416

25.528822 0.2041 7,741,010.947892

25.532222 0.204 7,571,706.645281

25.535623 0.2041 7,394,882.737172

25.539025 0.2041 7,339,468.913991

25.542425 0.204 7,250,477.519425

25.545827 0.2041 7,174,828.045446

25.549228 0.2041 7,107,165.813984

25.552628 0.204 6,967,652.615842

25.556030 0.2041 6,896,478.210262

25.559432 0.2041 7,037,810.892478

25.562832 0.204 7,110,945.345696

25.566233 0.2041 7,127,962.487356

25.569635 0.2041 7,123,634.735428

25.573035 0.204 6,899,929.383144

25.576437 0.2041 6,915,128.247750

25.579838 0.2041 7,019,258.376281

25.583238 0.204 6,798,588.346089

25.586640 0.2041 6,904,712.470300

25.590042 0.2041 7,083,044.718976

25.593442 0.204 6,924,236.085224

25.596843 0.2041 7,072,495.799005

25.600245 0.2041 7,179,425.632789

25.603645 0.204 7,022,464.453242

25.607047 0.2041 6,925,117.178295

25.610448 0.2041 6,936,985.898980

25.613850 0.2041 7,133,365.761434

25.617250 0.204 7,029,881.472923

25.620652 0.2041 6,885,327.628634

25.624053 0.2041 6,963,797.497077

25.627453 0.204 6,978,852.473820

25.630855 0.2041 7,070,636.721234

25.634257 0.2041 7,095,536.446868

25.637657 0.204 7,040,636.411791

25.641058 0.2041 7,020,495.812991

25.644460 0.2041 6,970,217.243065

25.647860 0.204 6,985,610.860401

25.651262 0.2041 7,106,805.703110

25.654663 0.2041 7,107,388.956674

25.658063 0.204 6,930,482.590773

25.661465 0.2041 6,878,029.930681

25.664867 0.2041 6,890,886.592104

25.668267 0.204 6,779,211.530352

25.671668 0.2041 6,928,180.281591

25.675070 0.2041 7,079,156.969378

25.678470 0.204 6,993,885.096595

25.681872 0.2041 7,059,554.832107

25.685273 0.2041 7,009,635.018881

25.688673 0.204 6,952,336.427446

25.692075 0.2041 7,092,892.901440

25.695477 0.2041 7,058,692.256995

25.698877 0.204 7,088,675.949473

25.702278 0.2041 7,056,083.860366

25.705680 0.2041 7,024,715.866222

25.709080 0.204 7,159,654.791028

25.712482 0.2041 7,081,575.823431

25.715883 0.2041 7,014,646.086512

25.719283 0.204 7,097,142.989406

25.722685 0.2041 7,301,563.887390

25.726087 0.2041 7,285,959.644142

25.729487 0.204 7,072,639.608454

25.732888 0.2041 7,091,402.696825

25.736290 0.2041 7,142,918.272305

25.739690 0.204 7,176,622.151382

25.743092 0.2041 7,212,699.125615

25.746493 0.2041 7,078,473.347528

25.749893 0.204 7,179,996.587367

25.753295 0.2041 7,156,162.662359

25.756697 0.2041 7,009,357.462636

25.760097 0.204 7,101,857.575953

25.763498 0.2041 6,898,038.322114

25.766900 0.2041 6,859,756.315553

25.770300 0.204 6,922,150.878664

25.773702 0.2041 6,890,069.889192

25.777103 0.2041 7,116,651.987835

25.780503 0.204 7,088,184.789048

25.783905 0.2041 6,951,866.047700

25.787307 0.2041 6,865,268.812441

25.790707 0.204 6,820,791.186505

25.794108 0.2041 7,061,186.467669

25.797510 0.2041 7,047,748.965257

25.800910 0.204 6,823,586.996271

25.804312 0.2041 6,961,612.277889

25.807713 0.2041 7,038,582.155064

25.811113 0.204 6,812,766.973563

25.814515 0.2041 6,803,698.310305

25.817917 0.2041 6,865,973.194264

25.821317 0.204 6,954,577.554081

25.824718 0.2041 7,154,271.060996

25.828120 0.2041 7,090,014.316810

25.831520 0.204 6,992,779.699932

25.834922 0.2041 7,013,950.413269

25.838323 0.2041 6,987,208.024158

25.841723 0.204 7,048,071.906615

25.845125 0.2041 7,022,533.576122

25.848527 0.2041 6,814,949.096605

25.851927 0.204 6,749,952.283678

25.855328 0.2041 6,979,013.731062

25.858730 0.2041 7,223,588.853005

25.862130 0.204 7,157,220.882071

25.865532 0.2041 7,008,737.645518

25.868933 0.2041 6,994,177.271312

25.872333 0.204 7,044,109.252514

25.875735 0.2041 7,088,643.118970

25.879137 0.2041 7,091,552.769618

25.882537 0.204 7,133,608.697366

25.885938 0.2041 7,252,137.860382

25.889340 0.2041 7,081,826.777088

25.892740 0.204 6,942,268.835889

25.896142 0.2041 6,974,650.542441

25.899543 0.2041 6,960,471.251855

25.902943 0.204 7,109,895.488070

25.906345 0.2041 7,042,591.022117

25.909747 0.2041 6,942,211.627676

25.913147 0.204 7,046,137.791037

25.916548 0.2041 7,178,528.236126

25.919950 0.2041 7,154,059.809994

25.923350 0.204 6,965,596.801802

25.926752 0.2041 6,904,035.488030

25.930153 0.2041 6,765,818.325484

25.933553 0.204 6,884,084.186054

25.936955 0.2041 6,959,705.205035

25.940357 0.2041 6,836,995.294981

25.943757 0.204 7,018,072.901127

25.947158 0.2041 6,995,081.243238

25.950560 0.2041 6,965,812.395972

25.953962 0.2041 7,141,185.252662

25.957362 0.204 7,082,367.427406

25.960763 0.2041 6,919,821.006376

25.964165 0.2041 7,129,165.956055

25.967565 0.204 7,349,363.065292

25.970967 0.2041 7,100,190.571635

25.974368 0.2041 6,977,372.839970

25.977768 0.204 7,108,554.145836

25.981170 0.2041 7,098,802.224869

25.984572 0.2041 7,011,658.361265

25.987972 0.204 7,125,011.237253

25.991373 0.2041 7,197,229.266182

25.994775 0.2041 7,108,578.550381

25.998175 0.204 7,073,634.518084

26.001577 0.2041 7,076,930.729278

26.004978 0.2041 7,087,596.229006

26.008378 0.204 6,998,201.152724

26.011780 0.2041 7,098,594.224579

26.015182 0.2041 7,135,593.771717

26.018582 0.204 7,074,606.335132

26.021983 0.2041 7,116,317.147678

26.025385 0.2041 7,115,595.337627

26.028785 0.204 7,198,746.300645

26.032187 0.2041 7,152,387.620063

26.035588 0.2041 7,144,019.132775

26.038988 0.204 7,158,171.153388

26.042390 0.2041 7,089,900.909996

26.045792 0.2041 7,096,353.208261

26.049192 0.204 7,135,818.027160

26.052593 0.2041 7,200,496.833827

26.055995 0.2041 7,245,860.687143

26.059395 0.204 7,223,294.591535

26.062797 0.2041 7,259,425.500492

26.066198 0.2041 7,290,626.156836

26.069598 0.204 7,098,308.307751

26.073000 0.2041 6,879,280.098531

26.076402 0.2041 6,965,041.278199

26.079802 0.204 7,146,108.973368

26.083203 0.2041 7,012,904.687347

26.086605 0.2041 6,902,099.903651

26.090005 0.204 6,935,522.340208

26.093407 0.2041 6,977,461.515140

26.096808 0.2041 6,968,833.644442

26.100208 0.204 6,935,088.551595

26.103610 0.2041 6,977,491.055836

26.107012 0.2041 7,090,249.517822

26.110412 0.204 7,017,139.758512

26.113813 0.2041 6,695,829.289276

26.117215 0.2041 6,793,647.116834

26.120615 0.204 6,938,342.986569

26.124017 0.2041 6,931,520.532940

26.127418 0.2041 7,140,771.995786

26.130818 0.204 6,999,873.969799

26.134220 0.2041 6,792,014.352359

26.137622 0.2041 6,859,988.797247

26.141022 0.204 6,964,853.797227

26.144423 0.2041 7,009,449.331248

26.147825 0.2041 6,866,222.272444

26.151225 0.204 6,928,581.135730

26.154627 0.2041 7,173,944.729546

26.158028 0.2041 7,030,854.589124

26.161428 0.204 6,696,725.980933

26.164830 0.2041 6,643,803.061012

26.168232 0.2041 6,839,812.365422

26.171632 0.204 6,900,207.723313

26.175033 0.2041 6,871,058.653146

26.178435 0.2041 6,885,369.363410

26.181835 0.204 6,811,819.937757

26.185237 0.2041 6,756,140.194883

26.188638 0.2041 6,776,135.423389

26.192038 0.204 6,986,453.534608

26.195440 0.2041 7,039,721.859414

26.198842 0.2041 6,893,847.769239

26.202242 0.204 6,796,637.644999

26.205643 0.2041 6,764,581.988333

26.209045 0.2041 6,879,323.971278

26.212445 0.204 6,824,687.930758

26.215847 0.2041 6,854,077.934091

26.219248 0.2041 6,798,049.519566

26.222648 0.204 6,618,321.173967

26.226050 0.2041 6,783,327.005078

26.229452 0.2041 6,840,140.516300

26.232852 0.204 6,696,255.997010

26.236253 0.2041 6,682,221.230470

26.239655 0.2041 6,830,480.434107

26.243055 0.204 6,725,182.311505

26.246457 0.2041 6,690,641.632015

26.249858 0.2041 7,012,328.620762

26.253258 0.204 6,978,660.159946

26.256660 0.2041 6,841,647.930215

26.260062 0.2041 6,922,943.083199

26.263462 0.204 6,922,400.100217

26.266863 0.2041 6,793,174.015619

26.270265 0.2041 6,784,389.457377

26.273665 0.204 6,788,047.998846

26.277067 0.2041 6,791,388.527439

26.280468 0.2041 6,979,633.462368

26.283868 0.204 6,819,331.416628

26.287270 0.2041 6,689,912.287536

26.290672 0.2041 6,825,046.738735

26.294072 0.204 6,849,946.131644

26.297473 0.2041 6,867,363.893910

26.300875 0.2041 6,774,276.576266

26.304277 0.2041 6,756,204.934763

26.307677 0.204 6,764,607.691721

26.311078 0.2041 6,832,388.358140

26.314480 0.2041 6,887,376.934831

26.317880 0.204 6,931,390.107346

26.321282 0.2041 6,987,225.997358

26.324683 0.2041 6,907,043.659164

26.328083 0.204 6,927,990.886442

26.331485 0.2041 7,031,653.011707

26.334887 0.2041 6,943,931.097528

26.338287 0.204 6,839,401.072337

26.341688 0.2041 7,074,802.499086

26.345090 0.2041 7,024,612.481049

26.348490 0.204 6,777,223.566024

26.351892 0.2041 6,906,107.292840

26.355293 0.2041 6,969,692.369768

26.358693 0.204 6,960,168.951298

26.362095 0.2041 7,108,360.754871

26.365497 0.2041 7,073,060.954950

26.368897 0.204 6,882,354.036550

26.372298 0.2041 6,868,006.474218

26.375700 0.2041 6,948,496.982697

26.379100 0.204 6,986,814.820619

26.382502 0.2041 7,016,789.340567

26.385903 0.2041 7,013,586.494062

26.389303 0.204 7,084,758.897570

26.392705 0.2041 7,195,726.830418

26.396107 0.2041 7,300,355.663388

26.399507 0.204 7,557,673.335912

26.402908 0.2041 7,889,383.084816

26.406310 0.2041 8,209,402.865070

26.409710 0.204 8,390,552.984027

26.413112 0.2041 8,663,812.721828

26.416513 0.2041 9,237,048.297851

26.419913 0.204 9,661,318.629614

26.423315 0.2041 9,752,835.279394

26.426717 0.2041 9,945,076.958290

26.430117 0.204 10,113,425.084427

26.433518 0.2041 9,956,178.361702

26.436920 0.2041 9,696,704.076191

26.440320 0.204 9,292,267.314749

26.443722 0.2041 8,788,354.181038

26.447123 0.2041 8,544,232.782011

26.450523 0.204 8,429,181.675171

26.453925 0.2041 8,045,658.391168

26.457327 0.2041 7,687,479.835980

26.460727 0.204 7,570,469.935905

26.464128 0.2041 7,464,721.942902

26.467530 0.2041 7,332,693.946458

26.470930 0.204 7,257,991.658312

26.474332 0.2041 7,220,358.100600

26.477733 0.2041 7,089,492.120918

26.481133 0.204 7,005,930.356396

26.484535 0.2041 7,196,037.184584

26.487937 0.2041 7,202,528.619478

26.491337 0.204 6,917,986.253115

26.494738 0.2041 6,835,380.586055

26.498140 0.2041 6,969,445.637180

26.501540 0.204 6,964,108.942831

26.504942 0.2041 7,041,651.081950

26.508343 0.2041 7,126,942.849253

26.511743 0.204 7,116,979.765083

26.515145 0.2041 7,135,865.108209

26.518547 0.2041 7,059,706.882956

26.521947 0.204 7,257,540.658515

26.525348 0.2041 7,172,917.630387

26.528750 0.2041 7,015,715.883183

26.532150 0.204 7,311,072.952846

26.535552 0.2041 7,272,729.304096

26.538953 0.2041 7,188,089.254884

26.542353 0.204 7,306,478.770275

26.545755 0.2041 7,391,540.916851

26.549157 0.2041 7,311,459.221981

26.552557 0.204 7,159,678.259053

26.555958 0.2041 7,398,833.199984

26.559360 0.2041 7,560,594.950686

26.562760 0.204 7,511,419.038346

26.566162 0.2041 7,674,374.100227

26.569563 0.2041 7,756,745.561716

26.572963 0.204 7,870,879.006563

26.576365 0.2041 8,240,934.759972

26.579767 0.2041 8,513,387.940346

26.583167 0.204 8,765,545.314646

26.586568 0.2041 9,305,761.355141

26.589970 0.2041 9,834,137.647216

26.593370 0.204 9,998,694.436637

26.596772 0.2041 10,105,911.837355

26.600173 0.2041 10,331,236.674424

26.603573 0.204 10,448,876.620755

26.606975 0.2041 10,185,636.672200

26.610377 0.2041 9,614,375.449487

26.613777 0.204 9,195,766.909462

26.617178 0.2041 8,968,876.100646

26.620580 0.2041 8,816,469.841740

26.623980 0.204 8,513,064.527841

26.627382 0.2041 8,055,399.004028

26.630783 0.2041 7,847,024.456758

26.634183 0.204 7,608,333.686740

26.637585 0.2041 7,215,487.850067

26.640987 0.2041 7,184,603.218035

26.644387 0.204 7,197,228.971619

26.647788 0.2041 6,878,120.529426

26.651190 0.2041 6,877,674.558339

26.654592 0.2041 6,984,734.073854

26.657992 0.204 7,029,873.271419

26.661393 0.2041 7,127,199.441979

26.664795 0.2041 6,990,914.545002

26.668195 0.204 6,958,404.258814

26.671597 0.2041 6,905,570.097812

26.674998 0.2041 6,922,091.895336

26.678398 0.204 7,121,630.475788

26.681800 0.2041 7,060,510.643682

26.685202 0.2041 6,962,385.571521

26.688602 0.204 6,937,889.007754

26.692003 0.2041 7,001,867.285943

26.695405 0.2041 6,898,834.030741

26.698805 0.204 6,727,910.111747

26.702207 0.2041 6,935,614.270891

26.705608 0.2041 6,992,031.404739

26.709008 0.204 6,868,965.895664

26.712410 0.2041 6,773,407.603812

26.715812 0.2041 6,814,455.800196

26.719212 0.204 6,968,407.363095

26.722613 0.2041 6,915,925.408016

26.726015 0.2041 6,938,713.863218

26.729415 0.204 7,016,820.642620

26.732817 0.2041 6,904,497.802081

26.736218 0.2041 6,770,838.915832

26.739618 0.204 6,792,740.228873

26.743020 0.2041 6,941,029.704062

26.746422 0.2041 6,995,283.506127

26.749822 0.204 6,778,895.325684

26.753223 0.2041 6,726,608.392668

26.756625 0.2041 6,902,998.066208

26.760025 0.204 6,854,360.070437

26.763427 0.2041 6,956,282.598245

26.766828 0.2041 7,105,880.338817

26.770228 0.204 7,202,275.134740

26.773630 0.2041 7,221,413.000292

26.777032 0.2041 7,167,857.863243

26.780432 0.204 7,380,890.664329

26.783833 0.2041 7,411,462.710118

26.787235 0.2041 7,454,254.297184

26.790635 0.204 7,522,439.695156

26.794037 0.2041 7,360,654.944123

26.797438 0.2041 7,495,634.505782

26.800838 0.204 7,577,364.736512

26.804240 0.2041 7,284,684.739697

26.807642 0.2041 7,136,269.154149

26.811042 0.204 7,326,327.107144

26.814443 0.2041 7,398,668.494090

26.817845 0.2041 7,279,521.823835

26.821245 0.204 7,248,724.636924

26.824647 0.2041 7,099,397.659459

26.828048 0.2041 7,212,491.223242

26.831448 0.204 7,359,763.214424

26.834850 0.2041 7,143,410.755912

26.838252 0.2041 7,115,988.826334

26.841652 0.204 7,155,313.844781

26.845053 0.2041 7,076,693.392687

26.848455 0.2041 7,086,198.993753

26.851855 0.204 7,315,840.734023

26.855257 0.2041 7,200,744.011276

26.858658 0.2041 6,953,822.063817

26.862058 0.204 7,028,666.401517

26.865460 0.2041 6,997,398.177206

26.868862 0.2041 7,070,673.338886

26.872262 0.204 7,158,345.564968

26.875663 0.2041 7,228,200.184073

26.879065 0.2041 7,171,272.736793

26.882465 0.204 6,961,013.085557

26.885867 0.2041 7,101,980.927148

26.889268 0.2041 6,984,351.205397

26.892668 0.204 6,869,065.836640

26.896070 0.2041 7,063,517.373788

26.899472 0.2041 6,971,183.445502

26.902872 0.204 6,960,256.650379

26.906273 0.2041 7,043,411.946408

26.909675 0.2041 6,886,968.332174

26.913075 0.204 6,811,057.383763

26.916477 0.2041 6,784,544.574533

26.919878 0.2041 6,824,030.636918

26.923278 0.204 6,992,443.724943

26.926680 0.2041 6,935,706.482981

26.930082 0.2041 7,005,973.682486

26.933482 0.204 7,122,197.387310

26.936883 0.2041 7,144,679.431010

26.940285 0.2041 7,430,236.664110

26.943685 0.204 7,667,104.269002

26.947087 0.2041 8,029,573.914956

26.950488 0.2041 8,547,173.737292

26.953888 0.204 8,869,865.974488

26.957290 0.2041 9,334,743.779802

26.960692 0.2041 9,925,514.772350

26.964092 0.204 10,538,348.307236

26.967493 0.2041 11,264,407.797110

26.970895 0.2041 11,779,648.925265

26.974295 0.204 12,159,511.888379

26.977697 0.2041 12,542,759.238899

26.981098 0.2041 12,647,183.119865

26.984498 0.204 12,643,526.564553

26.987900 0.2041 12,516,156.023861

26.991302 0.2041 12,095,940.383626

26.994702 0.204 11,601,057.432451

26.998103 0.2041 10,967,808.595605

27.001505 0.2041 10,194,587.435853

27.004905 0.204 9,565,901.211667

27.008307 0.2041 9,003,646.481615

27.011708 0.2041 8,453,696.006539

27.015110 0.2041 7,960,531.908323

27.018510 0.204 7,678,969.101171

27.021912 0.2041 7,576,319.467396

27.025313 0.2041 7,507,430.588920

27.028713 0.204 7,403,198.904708

27.032115 0.2041 7,186,546.342160

27.035517 0.2041 7,010,385.464842

27.038917 0.204 6,939,424.264228

27.042318 0.2041 6,903,518.844445

27.045720 0.2041 6,810,841.577898

27.049120 0.204 6,972,231.203106

27.052522 0.2041 6,985,171.247616

27.055923 0.2041 6,707,942.931899

27.059323 0.204 6,967,708.376486

27.062725 0.2041 7,179,552.150916

27.066127 0.2041 6,948,541.302802

27.069527 0.204 6,921,521.385497

27.072928 0.2041 6,922,473.186918

27.076330 0.2041 6,799,962.417558

27.079730 0.204 6,836,005.969475

27.083132 0.2041 6,956,533.127029

27.086533 0.2041 6,913,753.217950

27.089933 0.204 6,787,377.160131

27.093335 0.2041 6,859,777.380248

27.096737 0.2041 6,882,742.511145

27.100137 0.204 6,753,409.378585

27.103538 0.2041 6,886,627.818993

27.106940 0.2041 6,950,300.800001

27.110340 0.204 6,881,063.736164

27.113742 0.2041 6,981,904.805149

27.117143 0.2041 6,935,801.505047

27.120543 0.204 6,684,085.068090

27.123945 0.2041 6,791,329.726325

27.127347 0.2041 7,029,377.333853

27.130747 0.204 6,901,149.415203

27.134148 0.2041 6,826,128.497823

27.137550 0.2041 6,742,367.804207

27.140950 0.204 6,703,278.514765

27.144352 0.2041 6,890,135.403247

27.147753 0.2041 6,937,936.499219

27.151153 0.204 6,752,851.757248

27.154555 0.2041 6,625,816.017056

27.157957 0.2041 6,666,654.788864

27.161357 0.204 6,677,391.616863

27.164758 0.2041 6,761,560.391390

27.168160 0.2041 6,882,117.728366

27.171560 0.204 6,811,753.721908

27.174962 0.2041 6,756,090.493894

27.178363 0.2041 6,803,162.756477

27.181763 0.204 6,832,486.557602

27.185165 0.2041 6,843,313.184862

27.188567 0.2041 6,895,788.659996

27.191967 0.204 6,976,906.711826

27.195368 0.2041 6,899,593.009112

27.198770 0.2041 6,824,518.839169

27.202170 0.204 6,773,194.742532

27.205572 0.2041 6,854,034.824254

27.208973 0.2041 7,103,276.614474

27.212373 0.204 6,909,438.044540

27.215775 0.2041 6,882,124.777240

27.219177 0.2041 7,103,761.847581

27.222577 0.204 6,819,537.008429

27.225978 0.2041 6,694,888.288060

27.229380 0.2041 6,726,964.774188

27.232780 0.204 6,641,133.528398

27.236182 0.2041 6,669,872.526000

27.239583 0.2041 6,768,871.398188

27.242983 0.204 6,687,592.098808

27.246385 0.2041 6,582,735.514698

27.249787 0.2041 6,804,141.217447

27.253187 0.204 6,880,866.208102

27.256588 0.2041 6,772,411.993206

27.259990 0.2041 6,722,345.723512

27.263390 0.204 6,753,303.647135

27.266792 0.2041 6,933,455.315925

27.270193 0.2041 6,975,716.344538

27.273593 0.204 6,809,737.048883

27.276995 0.2041 6,791,200.850092

27.280397 0.2041 6,865,497.762954

27.283797 0.204 6,851,657.224249

27.287198 0.2041 6,762,697.573199

27.290600 0.2041 6,843,458.943442

27.294000 0.204 6,998,271.866787

27.297402 0.2041 6,925,926.471686

27.300803 0.2041 6,845,266.262422

27.304203 0.204 6,766,678.374033

27.307605 0.2041 6,775,252.985876

27.311007 0.2041 6,795,416.110834

27.314407 0.204 6,780,133.256604

27.317808 0.2041 6,658,568.584611

27.321210 0.2041 6,580,663.805339

27.324610 0.204 6,783,217.345808

27.328012 0.2041 6,767,520.432403

27.331413 0.2041 6,545,517.213418

27.334813 0.204 6,501,778.970984

27.338215 0.2041 6,597,480.242860

27.341617 0.2041 6,627,195.247686

27.345017 0.204 6,686,493.050523

27.348418 0.2041 6,873,944.826157

27.351820 0.2041 6,865,964.458481

27.355222 0.2041 6,727,541.988996

27.358622 0.204 6,745,592.289557

27.362023 0.2041 6,810,705.247318

27.365425 0.2041 6,911,802.173176

27.368825 0.204 6,846,537.088719

27.372227 0.2041 6,723,611.359656

27.375628 0.2041 7,027,319.497511

27.379028 0.204 7,158,303.017786

27.382430 0.2041 7,002,046.336300

27.385832 0.2041 7,102,933.902942

27.389232 0.204 7,104,409.293963

27.392633 0.2041 7,091,613.702425

27.396035 0.2041 7,189,130.214737

27.399435 0.204 6,979,272.435901

27.402837 0.2041 6,878,976.434530

27.406238 0.2041 7,096,020.782724

27.409638 0.204 7,083,071.286682

27.413040 0.2041 6,823,565.567687

27.416442 0.2041 6,684,190.260228

27.419842 0.204 6,770,535.403755

27.423243 0.2041 6,806,499.619209

27.426645 0.2041 6,763,966.662735

27.430045 0.204 6,824,721.377128

27.433447 0.2041 6,838,725.873683

27.436848 0.2041 6,949,277.670214

27.440248 0.204 6,969,949.382354

27.443650 0.2041 6,865,374.836127

27.447052 0.2041 7,011,601.564281

27.450452 0.204 7,093,498.859791

27.453853 0.2041 7,036,318.655047

27.457255 0.2041 7,038,654.632829

27.460655 0.204 7,050,251.794478

27.464057 0.2041 7,179,631.954720

27.467458 0.2041 7,194,197.012113

27.470858 0.204 6,996,529.349269

27.474260 0.2041 7,011,495.315728

27.477662 0.2041 7,064,262.189150

27.481062 0.204 6,997,549.410166

27.484463 0.2041 6,911,084.168397

27.487865 0.2041 6,888,225.056768

27.491265 0.204 6,949,229.077341

27.494667 0.2041 6,801,880.577658

27.498068 0.2041 6,658,596.446355

27.501468 0.204 6,761,886.478925

27.504870 0.2041 6,781,368.171381

27.508272 0.2041 6,774,751.811388

27.511672 0.204 6,852,559.002145

27.515073 0.2041 6,857,859.351911

27.518475 0.2041 6,857,819.376932

27.521875 0.204 6,837,664.554290

27.525277 0.2041 6,716,313.687762

27.528678 0.2041 6,755,206.127977

27.532078 0.204 7,024,225.533991

27.535480 0.2041 7,018,997.092606

27.538882 0.2041 6,900,931.269882

27.542282 0.204 6,860,575.947758

27.545683 0.2041 6,879,537.449082

27.549085 0.2041 6,949,651.116830

27.552485 0.204 6,789,211.332982

27.555887 0.2041 6,841,090.838447

27.559288 0.2041 7,037,135.560255

27.562688 0.204 6,948,486.170842

27.566090 0.2041 6,808,726.512402

27.569492 0.2041 6,839,308.886531

27.572892 0.204 6,835,100.371875

27.576293 0.2041 6,848,348.976572

27.579695 0.2041 7,007,614.322024

27.583095 0.204 7,164,318.957036

27.586497 0.2041 7,302,185.998935

27.589898 0.2041 7,462,190.198222

27.593298 0.204 7,617,066.889371

27.596700 0.2041 7,727,526.868051

27.600102 0.2041 7,936,677.327611

27.603502 0.204 8,125,976.982907

27.606903 0.2041 8,227,044.511205

27.610305 0.2041 8,180,849.137563

27.613705 0.204 8,075,529.925076

27.617107 0.2041 8,068,343.103123

27.620508 0.2041 8,158,858.130254

27.623908 0.204 8,003,969.427308

27.627310 0.2041 7,859,709.823395

27.630712 0.2041 8,067,465.219477

27.634112 0.204 8,122,796.108430

27.637513 0.2041 8,475,943.789872

27.640915 0.2041 9,225,532.515451

27.644315 0.204 10,262,804.022310

27.647717 0.2041 11,881,915.146679

27.651118 0.2041 14,135,279.796944

27.654518 0.204 17,518,561.345804

27.657920 0.2041 22,316,074.664901

27.661322 0.2041 28,464,635.725602

27.664722 0.204 36,349,368.873084

27.668123 0.2041 44,839,699.616274

27.671525 0.2041 53,921,282.070937

27.674925 0.204 64,556,677.575710

27.678327 0.2041 73,565,503.643192

27.681728 0.2041 81,464,685.038447

27.685128 0.204 89,378,272.717816

27.688530 0.2041 92,935,780.593400

27.691932 0.2041 93,362,134.418252

27.695332 0.204 94,046,080.272181

27.698733 0.2041 90,027,618.124186

27.702135 0.2041 80,404,832.768086

27.705535 0.204 70,523,123.461735

27.708937 0.2041 60,777,267.948095

27.712338 0.2041 50,694,333.865721

27.715740 0.2041 40,914,161.620621

27.719140 0.204 32,314,206.430486

27.722542 0.2041 25,255,897.672171

27.725943 0.2041 19,579,643.919864

27.729343 0.204 15,397,600.294965

27.732745 0.2041 12,553,237.111814

27.736147 0.2041 10,505,223.079104

27.739547 0.204 9,086,054.781986

27.742948 0.2041 8,329,933.981689

27.746350 0.2041 7,845,411.765620

27.749750 0.204 7,433,324.685883

27.753152 0.2041 7,239,548.924896

27.756553 0.2041 7,235,237.618991

27.759953 0.204 7,029,166.638365

27.763355 0.2041 6,895,974.776145

27.766757 0.2041 6,877,536.955910

27.770157 0.204 6,885,593.476529

27.773558 0.2041 6,916,308.270751

27.776960 0.2041 6,866,886.185972

27.780360 0.204 6,852,484.595576

27.783762 0.2041 6,822,256.596559

27.787163 0.2041 6,746,586.439796

27.790563 0.204 6,767,078.674133

27.793965 0.2041 6,817,674.025227

27.797367 0.2041 6,788,465.976874

27.800767 0.204 6,841,662.874601

27.804168 0.2041 6,793,262.805653

27.807570 0.2041 6,691,944.128822

27.810970 0.204 6,797,811.606840

27.814372 0.2041 6,897,966.823840

27.817773 0.2041 7,033,749.772208

27.821173 0.204 6,954,577.933906

27.824575 0.2041 6,924,691.921856

27.827977 0.2041 7,061,481.607223

27.831377 0.204 7,057,198.727412

27.834778 0.2041 7,037,319.372317

27.838180 0.2041 6,858,067.420265

27.841580 0.204 6,914,412.432671

27.844982 0.2041 7,055,128.962657

27.848383 0.2041 6,794,096.047691

27.851783 0.204 6,770,147.974219

27.855185 0.2041 6,969,656.051909

27.858587 0.2041 6,855,362.826036

27.861987 0.204 6,712,858.651317

27.865388 0.2041 6,606,008.909357

27.868790 0.2041 6,606,272.718931

27.872190 0.204 6,777,917.334198

27.875592 0.2041 6,709,897.861301

27.878993 0.2041 6,589,317.793332

27.882393 0.204 6,858,516.141109

27.885795 0.2041 6,972,652.042382

27.889197 0.2041 6,790,474.757333

27.892597 0.204 6,761,052.120968

27.895998 0.2041 6,815,053.408138

27.899400 0.2041 6,826,584.775159

27.902800 0.204 6,756,510.133333

27.906202 0.2041 6,698,406.414798

27.909603 0.2041 6,758,710.002739

27.913003 0.204 6,915,259.277252

27.916405 0.2041 6,944,115.646648

27.919807 0.2041 6,988,007.044146

27.923207 0.204 7,073,363.722445

27.926608 0.2041 7,023,933.609173

27.930010 0.2041 7,002,312.341957

27.933410 0.204 7,003,769.255989

27.936812 0.2041 7,073,807.018844

27.940213 0.2041 7,102,978.088140

27.943613 0.204 7,092,687.601675

27.947015 0.2041 7,130,760.615447

27.950417 0.2041 7,147,029.633270

27.953817 0.204 7,182,069.364214

27.957218 0.2041 7,115,391.754282

27.960620 0.2041 7,074,166.584615

27.964020 0.204 7,160,002.632683

27.967422 0.2041 7,209,974.655440

27.970823 0.2041 7,193,097.197512

27.974223 0.204 7,246,439.151447

27.977625 0.2041 7,250,167.254763

27.981027 0.2041 7,332,964.573899

27.984427 0.204 7,532,882.991835

27.987828 0.2041 7,559,891.755658

27.991230 0.2041 7,651,340.209968

27.994630 0.204 7,694,544.073410

27.998032 0.2041 7,633,017.592306

28.001433 0.2041 7,531,534.570850

28.004833 0.204 7,506,224.700812

28.008235 0.2041 7,658,482.289820

28.011637 0.2041 7,501,218.945222

28.015037 0.204 7,244,449.387758

28.018438 0.2041 7,287,987.561541

28.021840 0.2041 7,130,725.049658

28.025240 0.204 7,072,382.015715

28.028642 0.2041 7,189,098.373116

28.032043 0.2041 7,082,939.139860

28.035443 0.204 7,147,680.771205

28.038845 0.2041 7,224,968.603883

28.042247 0.2041 7,167,614.302041

28.045647 0.204 7,107,909.720802

28.049048 0.2041 7,219,457.332109

28.052450 0.2041 7,191,052.151340

28.055850 0.204 6,908,713.614089

28.059252 0.2041 7,015,689.441179

28.062653 0.2041 7,264,974.910464

28.066055 0.2041 7,015,902.237912

28.069455 0.204 6,738,578.609175

28.072857 0.2041 6,856,262.621521

28.076258 0.2041 6,862,891.853417

28.079658 0.204 6,882,470.114300

28.083060 0.2041 6,869,045.741770

28.086462 0.2041 6,793,992.238471

28.089862 0.204 6,819,653.293731

28.093263 0.2041 6,818,778.529323

28.096665 0.2041 6,845,511.114869

28.100065 0.204 6,808,612.810425

28.103467 0.2041 6,823,259.593023

28.106868 0.2041 6,885,932.298742

28.110268 0.204 6,742,740.423402

28.113670 0.2041 6,663,904.566172

28.117072 0.2041 6,797,347.812287

28.120472 0.204 6,749,843.017684

28.123873 0.2041 6,809,986.435726

28.127275 0.2041 6,939,497.687130

28.130675 0.204 6,859,575.583207

28.134077 0.2041 6,753,651.749591

28.137478 0.2041 6,826,037.674040

28.140878 0.204 6,977,639.090088

28.144280 0.2041 6,834,127.469602

28.147682 0.2041 6,807,742.425528

28.151082 0.204 6,928,704.567288

28.154483 0.2041 6,927,324.108780

28.157885 0.2041 7,081,078.738424

28.161285 0.204 7,075,047.285810

28.164687 0.2041 6,913,758.969021

28.168088 0.2041 6,928,492.350544

28.171488 0.204 6,887,968.529526

28.174890 0.2041 6,830,663.521317

28.178292 0.2041 6,735,644.246869

28.181692 0.204 6,783,971.286081

28.185093 0.2041 7,007,911.181911

28.188495 0.2041 7,002,440.041737

28.191895 0.204 6,859,461.760263

28.195297 0.2041 6,844,368.255187

28.198698 0.2041 6,899,619.038058

28.202098 0.204 6,953,850.759043

28.205500 0.2041 6,867,829.785489

28.208902 0.2041 6,530,408.969139

28.212302 0.204 6,569,423.360232

28.215703 0.2041 6,792,675.700512

28.219105 0.2041 6,628,804.802624

28.222505 0.204 6,638,966.363868

28.225907 0.2041 6,593,572.117419

28.229308 0.2041 6,543,219.242842

28.232708 0.204 6,838,559.075154

28.236110 0.2041 6,844,732.593102

28.239512 0.2041 6,662,540.161388

28.242912 0.204 6,559,697.805998

28.246313 0.2041 6,672,342.664095

28.249715 0.2041 6,750,296.644072

28.253115 0.204 6,639,278.768613

28.256517 0.2041 6,564,730.619751

28.259918 0.2041 6,398,452.065404

28.263318 0.204 6,572,196.971610

28.266720 0.2041 6,842,524.390494

28.270122 0.2041 6,781,043.584196

28.273522 0.204 6,824,479.435770

28.276923 0.2041 6,771,641.418452

28.280325 0.2041 6,689,216.320888

28.283725 0.204 6,769,135.487031

28.287127 0.2041 6,721,945.085729

28.290528 0.2041 6,862,672.341212

28.293928 0.204 6,894,164.335391

28.297330 0.2041 6,802,820.009772

28.300732 0.2041 7,120,555.408789

28.304132 0.204 7,206,473.350298

28.307533 0.2041 7,256,714.207882

28.310935 0.2041 7,537,188.676887

28.314335 0.204 7,609,785.598919

28.317737 0.2041 7,676,731.182428

28.321138 0.2041 7,766,718.146898

28.324538 0.204 7,891,308.306907

28.327940 0.2041 8,171,734.969977

28.331342 0.2041 8,338,176.224979

28.334742 0.204 8,275,998.065523

28.338143 0.2041 8,041,921.130025

28.341545 0.2041 7,867,123.430683

28.344945 0.204 7,942,539.986878

28.348347 0.2041 7,863,747.821730

28.351748 0.2041 7,594,215.162771

28.355148 0.204 7,539,380.044720

28.358550 0.2041 7,442,329.166285

28.361952 0.2041 7,301,957.543864

28.365352 0.204 7,410,441.820025

28.368753 0.2041 7,318,168.632818

28.372155 0.2041 7,315,652.065657

28.375555 0.204 7,407,824.364059

28.378957 0.2041 7,375,746.076069

28.382358 0.2041 7,587,122.948027

28.385758 0.204 7,722,978.141626

28.389160 0.2041 7,768,064.073061

28.392562 0.2041 7,740,656.628340

28.395962 0.204 7,723,958.024768

28.399363 0.2041 7,712,039.338938

28.402765 0.2041 7,525,267.775912

28.406165 0.204 7,400,496.681495

28.409567 0.2041 7,374,409.207274

28.412968 0.2041 7,337,094.085991

28.416370 0.2041 7,039,742.057860

28.419770 0.204 6,689,294.088116

28.423172 0.2041 6,729,970.198195

28.426573 0.2041 6,843,156.635224

28.429973 0.204 6,740,363.121468

28.433375 0.2041 6,728,215.731297

28.436777 0.2041 6,791,656.920589

28.440177 0.204 6,643,332.875076

28.443578 0.2041 6,686,183.528626

28.446980 0.2041 6,784,853.607268

28.450380 0.204 6,632,947.151033

28.453782 0.2041 6,579,776.707839

28.457183 0.2041 6,512,165.468652

28.460583 0.204 6,668,229.509386

28.463985 0.2041 6,820,499.898860

28.467387 0.2041 6,719,599.966482

28.470787 0.204 6,741,574.836829

28.474188 0.2041 6,778,167.443472

28.477590 0.2041 6,862,646.890532

28.480990 0.204 6,896,909.352638

28.484392 0.2041 6,816,447.069063

28.487793 0.2041 6,909,877.690482

28.491193 0.204 7,031,467.384981

28.494595 0.2041 7,124,431.741279

28.497997 0.2041 7,273,895.454371

28.501397 0.204 7,230,128.928988

28.504798 0.2041 7,167,509.930178

28.508200 0.2041 7,297,621.521735

28.511600 0.204 7,486,611.257736

28.515002 0.2041 7,407,493.643616

28.518403 0.2041 7,153,883.876469

28.521803 0.204 7,136,295.951139

28.525205 0.2041 6,984,992.222468

28.528607 0.2041 6,945,576.174906

28.532007 0.204 7,122,141.997462

28.535408 0.2041 7,120,755.003793

28.538810 0.2041 7,300,926.013633

28.542210 0.204 7,818,087.363581

28.545612 0.2041 8,350,781.137902

28.549013 0.2041 8,878,377.675704

28.552413 0.204 9,716,533.363389

28.555815 0.2041 10,844,837.836207

28.559217 0.2041 12,144,707.791487

28.562617 0.204 13,436,382.378355

28.566018 0.2041 14,841,464.484576

28.569420 0.2041 16,012,391.269178

28.572820 0.204 16,868,696.299773

28.576222 0.2041 17,747,855.094486

28.579623 0.2041 17,959,828.997140

28.583023 0.204 17,321,081.461560

28.586425 0.2041 16,582,890.317354

28.589827 0.2041 15,983,060.137049

28.593227 0.204 15,144,285.108421

28.596628 0.2041 13,956,370.628191

28.600030 0.2041 12,898,256.498325

28.603430 0.204 12,171,160.407067

28.606832 0.2041 11,463,500.535307

28.610233 0.2041 10,785,476.297747

28.613633 0.204 10,212,995.001367

28.617035 0.2041 9,719,939.032601

28.620437 0.2041 9,248,935.989529

28.623837 0.204 8,952,614.756834

28.627238 0.2041 8,676,257.848375

28.630640 0.2041 8,167,257.781479

28.634040 0.204 7,847,426.503581

28.637442 0.2041 7,858,889.554694

28.640843 0.2041 7,750,611.899977

28.644243 0.204 7,450,541.698433

28.647645 0.2041 7,293,460.196281

28.651047 0.2041 7,241,545.738827

28.654447 0.204 7,214,946.750202

28.657848 0.2041 7,144,477.032067

28.661250 0.2041 7,118,204.659127

28.664650 0.204 7,207,726.750016

28.668052 0.2041 7,089,895.281832

28.671453 0.2041 7,005,769.248402

28.674853 0.204 7,175,816.335351

28.678255 0.2041 7,286,486.428825

28.681657 0.2041 7,315,017.550038

28.685057 0.204 7,077,765.798946

28.688458 0.2041 7,058,123.379897

28.691860 0.2041 7,314,688.361145

28.695260 0.204 7,144,124.804378

28.698662 0.2041 7,226,368.529216

28.702063 0.2041 7,404,097.043488

28.705463 0.204 7,259,081.038230

28.708865 0.2041 7,497,685.281164

28.712267 0.2041 7,674,561.989935

28.715667 0.204 7,526,990.865710

28.719068 0.2041 7,573,678.243161

28.722470 0.2041 7,717,325.574458

28.725870 0.204 7,779,533.033872

28.729272 0.2041 7,745,289.740044

28.732673 0.2041 7,706,061.397537

28.736073 0.204 7,566,355.199276

28.739475 0.2041 7,341,339.554351

28.742877 0.2041 7,403,742.967938

28.746277 0.204 7,436,316.079551

28.749678 0.2041 7,184,048.302154

28.753080 0.2041 7,190,297.665780

28.756480 0.204 7,295,911.120373

28.759882 0.2041 7,237,698.831964

28.763283 0.2041 7,116,171.476369

28.766683 0.204 6,996,736.100181

28.770085 0.2041 7,066,034.172243

28.773487 0.2041 7,121,189.492048

28.776888 0.2041 7,073,285.383610

28.780288 0.204 7,106,822.291535

28.783690 0.2041 7,115,301.063034

28.787092 0.2041 7,045,435.474270

28.790492 0.204 7,047,821.819931

28.793893 0.2041 7,006,198.090953

28.797295 0.2041 6,878,807.422394

28.800695 0.204 6,944,185.609046

28.804097 0.2041 7,015,638.869821

28.807498 0.2041 6,687,946.523418

28.810898 0.204 6,680,073.733506

28.814300 0.2041 6,934,504.667258

28.817702 0.2041 6,737,543.531202

28.821102 0.204 6,655,223.792369

28.824503 0.2041 6,808,018.873698

28.827905 0.2041 6,870,053.491594

28.831305 0.204 6,887,733.645970

28.834707 0.2041 6,966,430.889642

28.838108 0.2041 6,900,591.888610

28.841508 0.204 6,893,930.396185

28.844910 0.2041 6,896,096.487418

28.848312 0.2041 6,800,995.763719

28.851712 0.204 6,884,426.211217

28.855113 0.2041 6,935,912.485576

28.858515 0.2041 6,928,765.872934

28.861915 0.204 6,956,767.972975

28.865317 0.2041 7,080,774.676947

28.868718 0.2041 7,075,062.527636

28.872118 0.204 6,984,449.007241

28.875520 0.2041 7,035,075.534876

28.878922 0.2041 6,922,705.686960

28.882322 0.204 6,965,491.816496

28.885723 0.2041 7,160,395.341060

28.889125 0.2041 7,044,645.817946

28.892525 0.204 7,009,061.805470

28.895927 0.2041 7,143,496.416407

28.899328 0.2041 7,017,241.990400

28.902728 0.204 6,952,608.072650

28.906130 0.2041 7,160,509.588079

28.909532 0.2041 7,212,003.778733

28.912932 0.204 7,123,153.043296

28.916333 0.2041 7,153,165.925718

28.919735 0.2041 7,289,353.623230

28.923135 0.204 7,201,953.164398

28.926537 0.2041 7,074,700.354027

28.929938 0.2041 7,200,886.568999

28.933338 0.204 7,349,588.975567

28.936740 0.2041 7,262,186.221842

28.940142 0.2041 7,300,359.675983

28.943542 0.204 7,497,751.464188

28.946943 0.2041 7,476,032.690862

28.950345 0.2041 7,291,061.035100

28.953745 0.204 7,205,685.180545

28.957147 0.2041 7,163,463.250954

28.960548 0.2041 6,897,660.103281

28.963948 0.204 6,947,766.956855

28.967350 0.2041 7,005,470.622071

28.970752 0.2041 6,663,133.734311

28.974152 0.204 6,687,945.832257

28.977553 0.2041 6,791,282.871955

28.980955 0.2041 6,610,643.015257

28.984355 0.204 6,600,408.913890

28.987757 0.2041 6,722,571.125279

28.991158 0.2041 6,787,761.046464

28.994558 0.204 6,744,383.341785

28.997960 0.2041 6,638,008.343318

29.001362 0.2041 6,572,210.221051

29.004762 0.204 6,477,722.674938

29.008163 0.2041 6,511,276.949604

29.011565 0.2041 6,668,637.693067

29.014965 0.204 6,605,346.839091

29.018367 0.2041 6,645,026.660122

29.021768 0.2041 6,758,551.492314

29.025168 0.204 6,723,623.355323

29.028570 0.2041 6,752,416.779100

29.031972 0.2041 6,775,545.084180

29.035372 0.204 6,857,471.907316

29.038773 0.2041 6,953,811.639274

29.042175 0.2041 6,976,843.839038

29.045575 0.204 7,102,420.363519

29.048977 0.2041 7,284,503.038015

29.052378 0.2041 7,351,645.854745

29.055778 0.204 7,148,377.499453

29.059180 0.2041 7,040,191.560336

29.062582 0.2041 7,133,228.017672

29.065982 0.204 7,080,648.022699

29.069383 0.2041 7,196,098.752442

29.072785 0.2041 7,239,632.130173

29.076185 0.204 7,147,733.687211

29.079587 0.2041 7,410,175.314558

29.082988 0.2041 7,564,072.522531

29.086388 0.204 7,692,317.415864

29.089790 0.2041 8,062,247.681702

29.093192 0.2041 8,148,859.384644

29.096592 0.204 8,050,776.155376

29.099993 0.2041 8,283,898.847878

29.103395 0.2041 8,469,096.923592

29.106795 0.204 8,099,385.651222

29.110197 0.2041 7,863,522.909659

29.113598 0.2041 7,819,939.628113

29.116998 0.204 7,669,690.634632

29.120400 0.2041 7,660,830.549878

29.123802 0.2041 7,390,179.360746

29.127202 0.204 7,063,662.731661

29.130603 0.2041 7,092,640.386326

29.134005 0.2041 6,958,013.324816

29.137405 0.204 6,871,252.855407

29.140807 0.2041 6,968,494.846494

29.144208 0.2041 6,733,301.147467

29.147610 0.2041 6,539,689.098746

29.151010 0.204 6,656,865.531433

29.154412 0.2041 6,606,373.275941

29.157813 0.2041 6,594,909.059950

29.161213 0.204 6,738,891.046121

29.164615 0.2041 6,651,428.747869

29.168017 0.2041 6,554,172.409735

29.171417 0.204 6,751,497.710920

29.174818 0.2041 7,156,669.108534

29.178220 0.2041 7,500,453.081731

29.181620 0.204 8,075,259.404043

29.185022 0.2041 8,925,519.334289

29.188423 0.2041 10,206,385.018001

29.191823 0.204 12,033,467.469714

29.195225 0.2041 13,930,881.752450

29.198627 0.2041 16,202,368.207584

29.202027 0.204 18,661,909.152720

29.205428 0.2041 20,812,318.260018

29.208830 0.2041 22,965,050.017849

29.212230 0.204 25,268,083.072760

29.215632 0.2041 26,563,015.331500

29.219033 0.2041 26,432,642.060677

29.222433 0.204 25,903,914.350354

29.225835 0.2041 24,640,063.385250

29.229237 0.2041 22,124,587.583258

29.232637 0.204 19,375,097.526843

29.236038 0.2041 16,917,612.091327

29.239440 0.2041 14,617,525.040361

29.242840 0.204 12,654,298.770612

29.246242 0.2041 10,819,964.549975

29.249643 0.2041 9,304,505.129184

29.253043 0.204 8,457,456.609851

29.256445 0.2041 7,780,410.531253

29.259847 0.2041 7,287,532.116214

29.263247 0.204 7,084,441.252166

29.266648 0.2041 6,911,221.640831

29.270050 0.2041 6,800,963.164609

29.273450 0.204 6,688,935.030724

29.276852 0.2041 6,751,471.635532

29.280253 0.2041 6,830,456.365280

29.283653 0.204 6,872,717.812202

29.287055 0.2041 6,939,156.970690

29.290457 0.2041 6,728,078.700102

29.293857 0.204 6,725,580.708264

29.297258 0.2041 6,755,192.556104

29.300660 0.2041 6,676,878.720480

29.304060 0.204 6,778,029.896609

29.307462 0.2041 6,758,015.788216

29.310863 0.2041 6,664,962.225308

29.314263 0.204 6,658,895.035772

29.317665 0.2041 6,638,426.418777

29.321067 0.2041 6,643,116.063086

29.324467 0.204 6,740,785.754930

29.327868 0.2041 6,821,105.180286

29.331270 0.2041 6,868,712.624173

29.334670 0.204 6,662,063.547909

29.338072 0.2041 6,355,203.980226

29.341473 0.2041 6,393,548.129146

29.344873 0.204 6,735,715.728111

29.348275 0.2041 6,833,046.117034

29.351677 0.2041 6,679,077.706782

29.355077 0.204 6,828,128.451352

29.358478 0.2041 6,868,878.071868

29.361880 0.2041 6,920,768.072831

29.365280 0.204 7,141,805.434378

29.368682 0.2041 7,102,122.490619

29.372083 0.2041 7,162,998.188673

29.375483 0.204 7,382,541.027100

29.378885 0.2041 7,280,385.743720

29.382287 0.2041 7,206,455.700129

29.385687 0.204 7,199,843.052789

29.389088 0.2041 7,014,214.581574

29.392490 0.2041 6,953,370.109027

29.395890 0.204 6,869,846.256940

29.399292 0.2041 6,778,155.051602

29.402693 0.2041 6,682,049.833136

29.406093 0.204 6,672,163.432844

29.409495 0.2041 6,714,662.698961

29.412897 0.2041 6,651,532.979272

29.416297 0.204 6,713,643.428042

29.419698 0.2041 6,750,008.371567

29.423100 0.2041 6,768,879.513744

29.426500 0.204 6,692,527.710235

29.429902 0.2041 6,644,891.659379

29.433303 0.2041 6,751,162.113988

29.436703 0.204 6,871,335.269592

29.440105 0.2041 6,838,662.586753

29.443507 0.2041 6,654,635.495582

29.446907 0.204 6,666,042.340741

29.450308 0.2041 6,696,285.545835

29.453710 0.2041 6,595,467.669641

29.457110 0.204 6,566,825.650145

29.460512 0.2041 6,655,956.077646

29.463913 0.2041 6,673,751.047669

29.467313 0.204 6,523,647.157429

29.470715 0.2041 6,505,343.592865

29.474117 0.2041 6,617,534.209934

29.477517 0.204 6,533,548.894272

29.480918 0.2041 6,583,758.352320

29.484320 0.2041 6,755,136.950598

29.487720 0.204 6,661,214.388123

29.491122 0.2041 6,617,950.796891

29.494523 0.2041 6,604,285.434274

29.497925 0.2041 6,599,398.096078

29.501325 0.204 6,747,006.944734

29.504727 0.2041 6,671,719.802522

29.508128 0.2041 6,634,367.418961

29.511528 0.204 6,760,972.810847

29.514930 0.2041 6,575,258.431436

29.518332 0.2041 6,511,357.572749

29.521732 0.204 6,703,821.642978

29.525133 0.2041 6,689,591.402943

29.528535 0.2041 6,528,164.198991

29.531935 0.204 6,520,292.865676

29.535337 0.2041 6,708,090.936764

29.538738 0.2041 6,726,690.705256

29.542138 0.204 6,663,935.957947

29.545540 0.2041 6,629,325.464728

29.548942 0.2041 6,625,104.101785

29.552342 0.204 6,634,059.379862

29.555743 0.2041 6,613,157.053708

29.559145 0.2041 6,631,203.323847

29.562545 0.204 6,628,947.950051

29.565947 0.2041 6,659,716.469943

29.569348 0.2041 6,607,999.814092

29.572748 0.204 6,605,466.561279

29.576150 0.2041 6,728,646.010991

29.579552 0.2041 6,714,293.242270

29.582952 0.204 6,572,898.486594

29.586353 0.2041 6,556,379.290372

29.589755 0.2041 6,603,064.612666

29.593155 0.204 6,626,363.407801

29.596557 0.2041 6,628,985.392677

29.599958 0.2041 6,640,526.011576

29.603358 0.204 6,595,987.808073

29.606760 0.2041 6,525,277.520244

29.610162 0.2041 6,489,557.854172

29.613562 0.204 6,524,637.229770

29.616963 0.2041 6,616,106.712154

29.620365 0.2041 6,474,422.881157

29.623765 0.204 6,538,846.453890

29.627167 0.2041 6,609,099.571418

29.630568 0.2041 6,375,819.090334

29.633968 0.204 6,478,717.008863

29.637370 0.2041 6,621,312.126814

29.640772 0.2041 6,450,116.808450

29.644172 0.204 6,496,825.983280

29.647573 0.2041 6,571,597.951154

29.650975 0.2041 6,424,112.632680

29.654375 0.204 6,450,110.040742

29.657777 0.2041 6,584,362.848123

29.661178 0.2041 6,512,454.511830

29.664578 0.204 6,527,706.985188

29.667980 0.2041 6,533,111.412133

29.671382 0.2041 6,327,749.501010

29.674782 0.204 6,313,845.838521

29.678183 0.2041 6,474,578.363750

29.681585 0.2041 6,575,037.339677

29.684985 0.204 6,523,922.477290

29.688387 0.2041 6,478,515.265580

29.691788 0.2041 6,543,403.741882

29.695188 0.204 6,543,067.947356

29.698590 0.2041 6,551,121.182182

29.701992 0.2041 6,567,839.575037

29.705392 0.204 6,461,331.828298

29.708793 0.2041 6,416,361.298776

29.712195 0.2041 6,650,593.192983

29.715595 0.204 6,977,045.018884

29.718997 0.2041 7,194,127.403979

29.722398 0.2041 7,921,219.332911

29.725798 0.204 9,383,869.966901

29.729200 0.2041 11,604,992.490157

29.732602 0.2041 15,851,082.620295

29.736002 0.204 22,836,491.414199

29.739403 0.2041 33,006,911.684335

29.742805 0.2041 48,472,076.181588

29.746205 0.204 69,653,492.908043

29.749607 0.2041 95,501,249.538435

29.753008 0.2041 126,467,828.862626

29.756408 0.204 161,868,534.294173

29.759810 0.2041 201,546,072.785926

29.763212 0.2041 241,236,433.361943

29.766612 0.204 274,048,929.478842

29.770013 0.2041 295,824,671.897659

29.773415 0.2041 302,091,151.781367

29.776815 0.204 296,502,795.750807

29.780217 0.2041 277,885,081.992166

29.783618 0.2041 251,182,264.430824

29.787018 0.204 223,412,956.341751

29.790420 0.2041 189,518,038.470185

29.793822 0.2041 154,040,591.671738

29.797222 0.204 120,208,122.111693

29.800623 0.2041 90,349,070.786897

29.804025 0.2041 66,677,787.279903

29.807425 0.204 49,572,275.628488

29.810827 0.2041 37,139,973.626379

29.814228 0.2041 28,225,689.730338

29.817628 0.204 23,187,044.336818

29.821030 0.2041 19,053,661.754312

29.824432 0.2041 16,423,460.071837

29.827832 0.204 14,977,135.508225

29.831233 0.2041 13,736,208.648402

29.834635 0.2041 12,871,199.370793

29.838035 0.204 12,283,762.020476

29.841437 0.2041 11,877,769.280245

29.844838 0.2041 11,456,154.696880

29.848240 0.2041 11,214,350.005493

29.851640 0.204 11,003,328.443208

29.855042 0.2041 10,593,300.212880

29.858443 0.2041 10,451,543.562722

29.861843 0.204 10,296,932.105952

29.865245 0.2041 10,143,276.509715

29.868647 0.2041 10,382,403.504467

29.872047 0.204 10,111,907.545283

29.875448 0.2041 9,695,052.190676

29.878850 0.2041 9,650,392.054987

29.882250 0.204 9,574,433.255610

29.885652 0.2041 9,446,330.772110

29.889053 0.2041 9,180,190.617094

29.892453 0.204 9,030,973.578573

29.895855 0.2041 8,941,229.361992

29.899257 0.2041 8,857,723.250075

29.902657 0.204 8,779,184.681105

29.906058 0.2041 8,671,439.168347

29.909460 0.2041 8,575,315.118175

29.912860 0.204 8,550,070.000829

29.916262 0.2041 8,573,719.658451

29.919663 0.2041 8,398,511.562051

29.923063 0.204 8,256,162.084771

29.926465 0.2041 8,334,942.631405

29.929867 0.2041 8,389,781.626515

29.933267 0.204 8,296,364.095572

29.936668 0.2041 8,390,625.511111

29.940070 0.2041 8,555,969.937058

29.943470 0.204 8,561,117.300001

29.946872 0.2041 8,624,938.473487

29.950273 0.2041 8,767,604.706778

29.953673 0.204 8,736,229.552825

29.957075 0.2041 8,551,926.483304

29.960477 0.2041 8,460,445.059554

29.963877 0.204 8,385,187.971387

29.967278 0.2041 8,541,101.483108

29.970680 0.2041 8,500,687.257645

29.974080 0.204 8,236,840.088738

29.977482 0.2041 8,176,390.765524

29.980883 0.2041 8,102,397.699089

29.984283 0.204 7,962,035.075860

29.987685 0.2041 7,739,304.269265

29.991087 0.2041 7,845,457.016809

29.994487 0.204 7,927,742.660542

29.997888 0.2041 7,802,946.647774

30.001290 0.2041 7,796,579.554780

30.004690 0.204 7,763,140.807525

30.008092 0.2041 7,772,584.605573

30.011493 0.2041 7,745,922.665582

30.014893 0.204 7,697,169.284138

30.018295 0.2041 7,631,198.766112

30.021697 0.2041 7,572,639.399345

30.025097 0.204 7,564,119.874437

30.028498 0.2041 7,468,052.690176

30.031900 0.2041 7,435,887.086581

30.035300 0.204 7,488,596.124735

30.038702 0.2041 7,400,079.625966

30.042103 0.2041 7,426,504.966686

30.045503 0.204 7,508,115.060003

30.048905 0.2041 7,605,021.146985

30.052307 0.2041 7,799,434.361132

30.055707 0.204 7,759,806.273645

30.059108 0.2041 7,854,652.948778

30.062510 0.2041 8,086,383.256455

30.065910 0.204 8,226,558.782374

30.069312 0.2041 8,481,974.386448

30.072713 0.2041 8,631,341.854694

30.076113 0.204 8,932,020.468198

30.079515 0.2041 9,343,689.068040

30.082917 0.2041 9,314,890.068099

30.086317 0.204 9,214,017.202095

30.089718 0.2041 9,190,809.012033

30.093120 0.2041 9,076,864.364575

30.096520 0.204 8,847,424.132224

30.099922 0.2041 8,581,440.763341

30.103323 0.2041 8,395,174.316413

30.106723 0.204 8,196,893.397663

30.110125 0.2041 7,949,394.965345

30.113527 0.2041 7,782,121.643525

30.116927 0.204 7,634,098.182546

30.120328 0.2041 7,295,273.018746

30.123730 0.2041 7,296,725.273954

30.127130 0.204 7,379,030.392093

30.130532 0.2041 7,306,909.199657

30.133933 0.2041 7,388,195.814627

30.137333 0.204 7,314,558.065285

30.140735 0.2041 7,338,068.175821

30.144137 0.2041 7,375,061.963240

30.147537 0.204 7,118,351.928026

30.150938 0.2041 7,187,829.977200

30.154340 0.2041 7,329,715.204177

30.157740 0.204 7,149,885.712843

30.161142 0.2041 7,165,212.999876

30.164543 0.2041 7,138,309.597557

30.167943 0.204 7,037,836.226066

30.171345 0.2041 6,969,117.802357

30.174747 0.2041 6,932,010.263006

30.178147 0.204 7,042,187.016660

30.181548 0.2041 7,094,393.990794

30.184950 0.2041 7,132,924.421606

30.188350 0.204 7,120,011.150487

30.191752 0.2041 7,098,724.113712

30.195153 0.2041 7,187,816.604018

30.198553 0.204 7,113,461.135647

30.201955 0.2041 6,993,493.003695

30.205357 0.2041 7,114,167.115659

30.208757 0.204 7,049,943.138792

30.212158 0.2041 6,932,566.503419

30.215560 0.2041 7,002,720.618040

30.218960 0.204 7,009,574.529822

30.222362 0.2041 6,915,788.487519

30.225763 0.2041 6,958,145.591278

30.229163 0.204 6,979,644.617636

30.232565 0.2041 6,894,045.591089

30.235967 0.2041 7,090,851.569867

30.239367 0.204 7,059,241.829588

30.242768 0.2041 6,845,549.792265

30.246170 0.2041 6,849,835.220332

30.249572 0.2041 6,960,846.723474

30.252972 0.204 7,109,963.395871

30.256373 0.2041 7,146,302.470397

30.259775 0.2041 6,957,571.583474

30.263175 0.204 6,849,240.754429

30.266577 0.2041 7,028,729.097003

30.269978 0.2041 6,895,550.798159

30.273378 0.204 6,907,090.570863

30.276780 0.2041 7,035,421.062023

30.280182 0.2041 6,755,797.256082

30.283582 0.204 6,814,101.815990

30.286983 0.2041 7,129,183.499968

30.290385 0.2041 7,070,203.427176

30.293785 0.204 6,817,152.521898

30.297187 0.2041 6,884,197.298742

30.300588 0.2041 6,957,024.750284

30.303988 0.204 6,770,577.255343

30.307390 0.2041 6,867,073.075046

30.310792 0.2041 6,910,005.323088

30.314192 0.204 6,827,626.024093

30.317593 0.2041 6,947,641.386072

30.320995 0.2041 6,975,249.095573

30.324395 0.204 6,897,229.273532

30.327797 0.2041 6,884,902.535778

30.331198 0.2041 7,024,361.371108

30.334598 0.204 6,942,358.371586

30.338000 0.2041 6,895,175.761289

30.341402 0.2041 6,847,827.159940

30.344802 0.204 6,761,346.977891

30.348203 0.2041 6,911,959.511114

30.351605 0.2041 6,766,273.791541

30.355005 0.204 6,725,416.479501

30.358407 0.2041 6,792,705.183240

30.361808 0.2041 6,673,992.982627

30.365208 0.204 6,715,161.439564

30.368610 0.2041 6,779,439.574451

30.372012 0.2041 6,788,037.850693

30.375412 0.204 6,741,731.282632

30.378813 0.2041 6,617,604.839977

30.382215 0.2041 6,595,060.236627

30.385615 0.204 6,680,558.904171

30.389017 0.2041 6,690,776.134578

30.392418 0.2041 6,708,167.186652

30.395818 0.204 6,840,414.848845

30.399220 0.2041 6,942,571.098102

30.402622 0.2041 6,729,003.304307

30.406022 0.204 6,582,173.639045

30.409423 0.2041 6,778,027.280034

30.412825 0.2041 6,743,527.487215

30.416225 0.204 6,660,386.404551

30.419627 0.2041 6,779,662.630151

30.423028 0.2041 6,832,668.382289

30.426428 0.204 6,853,524.261089

30.429830 0.2041 6,791,655.225863

30.433232 0.2041 6,741,765.032468

30.436632 0.204 6,729,374.904938

30.440033 0.2041 6,728,903.309850

30.443435 0.2041 6,858,225.988276

30.446835 0.204 6,907,693.641471

30.450237 0.2041 6,931,608.301809

30.453638 0.2041 7,040,954.578174

30.457038 0.204 7,252,367.252058

30.460440 0.2041 7,575,989.799472

30.463842 0.2041 7,825,556.730152

30.467242 0.204 8,200,658.428844

30.470643 0.2041 8,765,252.991323

30.474045 0.2041 9,334,982.742878

30.477445 0.204 9,868,318.394162

30.480847 0.2041 10,269,211.435860

30.484248 0.2041 10,647,118.867334

30.487648 0.204 10,916,420.968233

30.491050 0.2041 10,862,708.590976

30.494452 0.2041 10,815,036.438962

30.497852 0.204 10,697,349.541989

30.501253 0.2041 10,213,684.184403

30.504655 0.2041 9,686,817.626662

30.508055 0.204 9,162,912.825176

30.511457 0.2041 8,739,896.241076

30.514858 0.2041 8,335,179.909914

30.518258 0.204 7,746,666.718851

30.521660 0.2041 7,450,884.864997

30.525062 0.2041 7,334,810.708899

30.528462 0.204 7,142,224.472262

30.531863 0.2041 6,924,856.557045

30.535265 0.2041 6,799,996.064354

30.538665 0.204 6,855,485.244994

30.542067 0.2041 6,836,159.465166

30.545468 0.2041 6,809,125.992722

30.548868 0.204 6,942,748.799347

30.552270 0.2041 6,986,592.538971

30.555672 0.2041 6,871,428.547033

30.559072 0.204 6,870,827.320285

30.562473 0.2041 6,705,962.070989

30.565875 0.2041 6,714,921.175495

30.569275 0.204 6,992,794.052885

30.572677 0.2041 6,955,839.288543

30.576078 0.2041 6,938,317.862199

30.579478 0.204 6,945,481.876962

30.582880 0.2041 6,991,669.932198

30.586282 0.2041 7,015,707.863514

30.589682 0.204 6,970,280.149242

30.593083 0.2041 6,938,627.547522

30.596485 0.2041 7,052,320.273435

30.599885 0.204 7,215,594.966304

30.603287 0.2041 7,174,890.944089

30.606688 0.2041 7,299,197.080371

30.610090 0.2041 7,523,451.849388

30.613490 0.204 7,708,920.498812

30.616892 0.2041 7,649,594.951880

30.620293 0.2041 7,392,326.470115

30.623693 0.204 7,474,946.167046

30.627095 0.2041 7,595,004.248730

30.630497 0.2041 7,402,412.646334

30.633897 0.204 7,255,903.537343

30.637298 0.2041 7,095,046.609998

30.640700 0.2041 7,004,860.779676

30.644100 0.204 7,121,768.061742

30.647502 0.2041 7,056,419.546595

30.650903 0.2041 7,045,219.821438

30.654303 0.204 7,180,602.446515

30.657705 0.2041 7,093,182.582677

30.661107 0.2041 7,159,788.306038

30.664507 0.204 7,457,972.989944

30.667908 0.2041 7,448,409.725727

30.671310 0.2041 7,491,250.275336

30.674710 0.204 7,558,403.462959

30.678112 0.2041 7,593,430.519654

30.681513 0.2041 7,720,390.375763

30.684913 0.204 7,781,341.371301

30.688315 0.2041 7,813,484.200013

30.691717 0.2041 7,624,715.195771

30.695117 0.204 7,475,486.767986

30.698518 0.2041 7,442,491.979994

30.701920 0.2041 7,443,161.223015

30.705320 0.204 7,287,994.642376

30.708722 0.2041 7,066,338.122269

30.712123 0.2041 6,990,804.029483

30.715523 0.204 6,903,623.555527

30.718925 0.2041 6,910,312.954192

30.722327 0.2041 6,936,676.318504

30.725727 0.204 6,989,736.101625

30.729128 0.2041 7,023,243.011870

30.732530 0.2041 7,156,344.015934

30.735930 0.204 7,321,871.614876

30.739332 0.2041 7,214,004.073749

30.742733 0.2041 7,235,901.008634

30.746133 0.204 7,338,627.378677

30.749535 0.2041 7,238,948.859282

30.752937 0.2041 7,378,567.451821

30.756337 0.204 7,348,744.324737

30.759738 0.2041 7,079,868.225922

30.763140 0.2041 7,033,415.842545

30.766540 0.204 6,941,117.317388

30.769942 0.2041 6,893,010.926257

30.773343 0.2041 6,850,530.112157

30.776743 0.204 6,810,347.977454

30.780145 0.2041 6,813,489.345730

30.783547 0.2041 6,678,748.446356

30.786947 0.204 6,649,302.518029

30.790348 0.2041 6,648,318.927398

30.793750 0.2041 6,661,546.057765

30.797150 0.204 6,638,940.846586

30.800552 0.2041 6,552,382.608134

30.803953 0.2041 6,735,478.936708

30.807353 0.204 6,886,490.696453

30.810755 0.2041 6,780,609.060263

30.814157 0.2041 6,617,761.439217

30.817557 0.204 6,693,264.587814

30.820958 0.2041 6,810,598.718100

30.824360 0.2041 6,755,710.541011

30.827760 0.204 6,646,934.003981

30.831162 0.2041 6,835,758.632729

30.834563 0.2041 7,255,564.588332

30.837963 0.204 7,498,867.717013

30.841365 0.2041 7,712,154.325193

30.844767 0.2041 8,197,083.125291

30.848167 0.204 8,712,915.033368

30.851568 0.2041 9,289,981.808163

30.854970 0.2041 9,900,882.897835

30.858370 0.204 10,001,652.304719

30.861772 0.2041 10,320,402.707714

30.865173 0.2041 10,651,272.130700

30.868573 0.204 10,494,067.628288

30.871975 0.2041 10,290,865.097329

30.875377 0.2041 9,879,123.359796

30.878777 0.204 9,264,012.667056

30.882178 0.2041 8,646,954.603268

30.885580 0.2041 8,169,465.802474

30.888980 0.204 7,991,334.916410

30.892382 0.2041 7,701,138.286854

30.895783 0.2041 7,284,650.172743

30.899183 0.204 7,309,901.934636

30.902585 0.2041 7,151,549.829789

30.905987 0.2041 6,860,097.751569

30.909387 0.204 6,991,881.116014

30.912788 0.2041 7,089,549.442136

30.916190 0.2041 7,134,036.803657

30.919590 0.204 7,044,954.838049

30.922992 0.2041 6,801,233.318257

30.926393 0.2041 6,884,256.812961

30.929793 0.204 7,053,443.783218

30.933195 0.2041 6,960,556.407633

30.936597 0.2041 6,966,726.582447

30.939997 0.204 6,958,543.128686

30.943398 0.2041 6,795,079.877307

30.946800 0.2041 6,762,508.706721

30.950200 0.204 6,760,551.106512

30.953602 0.2041 6,751,451.429121

30.957003 0.2041 6,762,152.466786

30.960403 0.204 6,773,922.085060

30.963805 0.2041 6,682,255.261699

30.967207 0.2041 6,656,260.835565

30.970607 0.204 6,675,165.713665

30.974008 0.2041 6,511,690.403013

30.977410 0.2041 6,603,656.799319

30.980812 0.2041 6,684,089.499018

30.984212 0.204 6,584,178.392184

30.987613 0.2041 6,594,430.898571

30.991015 0.2041 6,659,274.741812

30.994415 0.204 6,641,506.537621

30.997817 0.2041 6,596,463.901819

31.001218 0.2041 6,573,389.935613

31.004618 0.204 6,438,463.295405

31.008020 0.2041 6,366,180.517945

31.011422 0.2041 6,477,127.799725

31.014822 0.204 6,752,123.668998

31.018223 0.2041 6,793,489.421728

31.021625 0.2041 6,619,632.592161

31.025025 0.204 6,564,820.370515

31.028427 0.2041 6,584,089.765128

31.031828 0.2041 6,611,123.224711

31.035228 0.204 6,615,873.244431

31.038630 0.2041 6,714,394.787897

31.042032 0.2041 6,676,212.772432

31.045432 0.204 6,564,146.783977

31.048833 0.2041 6,519,293.171585

31.052235 0.2041 6,367,276.068665

31.055635 0.204 6,591,441.427020

31.059037 0.2041 6,777,592.132365

31.062438 0.2041 6,640,601.918789

31.065838 0.204 6,546,732.500304

31.069240 0.2041 6,464,487.414190

31.072642 0.2041 6,543,800.227880

31.076042 0.204 6,630,619.984715

31.079443 0.2041 6,702,531.677387

31.082845 0.2041 6,538,928.028327

31.086245 0.204 6,481,665.130926

31.089647 0.2041 6,695,848.728577

31.093048 0.2041 6,615,339.687902

31.096448 0.204 6,452,144.537440

31.099850 0.2041 6,475,097.561832

31.103252 0.2041 6,652,130.410982

31.106652 0.204 6,705,823.019597

31.110053 0.2041 6,652,885.315017

31.113455 0.2041 6,684,594.459068

31.116855 0.204 6,634,782.368677

31.120257 0.2041 6,718,183.503216

31.123658 0.2041 6,915,742.906658

31.127058 0.204 6,823,773.918871

31.130460 0.2041 6,826,567.711390

31.133862 0.2041 6,987,376.982246

31.137262 0.204 6,891,177.818046

31.140663 0.2041 6,911,089.522387

31.144065 0.2041 6,947,519.749824

31.147465 0.204 6,828,013.116897

31.150867 0.2041 6,962,406.063045

31.154268 0.2041 6,920,004.091798

31.157668 0.204 6,732,416.951552

31.161070 0.2041 6,664,771.510520

31.164472 0.2041 6,712,452.493858

31.167872 0.204 6,909,755.536264

31.171273 0.2041 6,805,532.538597

31.174675 0.2041 6,673,293.988212

31.178075 0.204 6,707,336.754449

31.181477 0.2041 6,658,214.904142

31.184878 0.2041 6,709,680.513186

31.188278 0.204 6,635,229.641727

31.191680 0.2041 6,501,312.794091

31.195082 0.2041 6,531,514.582767

31.198482 0.204 6,466,341.926314

31.201883 0.2041 6,591,039.576788

31.205285 0.2041 6,706,593.311066

31.208685 0.204 6,491,943.753936

31.212087 0.2041 6,542,677.010918

31.215488 0.2041 6,726,836.887146

31.218888 0.204 6,683,951.270359

31.222290 0.2041 6,592,110.118826

31.225692 0.2041 6,557,949.450983

31.229092 0.204 6,459,401.339052

31.232493 0.2041 6,531,450.565713

31.235895 0.2041 6,710,729.767923

31.239295 0.204 6,494,033.763084

31.242697 0.2041 6,390,269.473955

31.246098 0.2041 6,567,334.522080

31.249498 0.204 6,718,889.362403

31.252900 0.2041 6,681,312.661209

31.256302 0.2041 6,502,975.320661

31.259702 0.204 6,419,055.635356

31.263103 0.2041 6,476,455.354732

31.266505 0.2041 6,561,403.860950

31.269905 0.204 6,692,132.235570

31.273307 0.2041 6,706,953.636714

31.276708 0.2041 6,676,745.800242

31.280108 0.204 6,788,587.114110

31.283510 0.2041 6,680,938.755661

31.286912 0.2041 6,708,402.100092

31.290312 0.204 6,734,793.730500

31.293713 0.2041 6,632,519.083034

31.297115 0.2041 6,701,755.913827

31.300515 0.204 6,798,562.026758

31.303917 0.2041 6,749,589.027382

31.307318 0.2041 6,661,863.939630

31.310718 0.204 6,737,252.246603

31.314120 0.2041 6,712,852.982064

31.317522 0.2041 6,621,572.103212

31.320922 0.204 6,589,987.229274

31.324323 0.2041 6,565,701.100607

31.327725 0.2041 6,716,164.448865

31.331125 0.204 6,825,689.026518

31.334527 0.2041 6,535,985.320994

31.337928 0.2041 6,334,974.629909

31.341328 0.204 6,396,018.428986

31.344730 0.2041 6,481,891.806227

31.348132 0.2041 6,574,425.981235

31.351532 0.204 6,508,837.844190

31.354933 0.2041 6,393,859.550303

31.358335 0.2041 6,429,484.390817

31.361737 0.2041 6,474,443.069693

31.365137 0.204 6,514,864.005284

31.368538 0.2041 6,580,036.558089

31.371940 0.2041 6,503,489.025662

31.375340 0.204 6,428,310.140093

31.378742 0.2041 6,444,763.549141

31.382143 0.2041 6,513,959.749051

31.385543 0.204 6,536,261.594735

31.388945 0.2041 6,402,936.855409

31.392347 0.2041 6,346,346.112545

31.395747 0.204 6,517,659.991885

31.399148 0.2041 6,632,306.727041

31.402550 0.2041 6,657,739.214058

31.405950 0.204 6,647,875.653047

31.409352 0.2041 6,577,686.154986

31.412753 0.2041 6,660,700.903644

31.416153 0.204 6,818,813.508677

31.419555 0.2041 6,821,272.093603

31.422957 0.2041 6,688,372.198363

31.426357 0.204 6,701,158.330409

31.429758 0.2041 6,796,416.639419

31.433160 0.2041 6,623,139.871259

31.436560 0.204 6,436,364.616082

31.439962 0.2041 6,488,605.982697

31.443363 0.2041 6,472,150.222441

31.446763 0.204 6,555,364.512610

31.450165 0.2041 6,567,461.806628

31.453567 0.2041 6,387,422.987571

31.456967 0.204 6,436,856.802587

31.460368 0.2041 6,445,780.294129

31.463770 0.2041 6,476,529.162506

31.467170 0.204 6,478,383.128622

31.470572 0.2041 6,376,909.169441

31.473973 0.2041 6,389,183.468442

31.477373 0.204 6,428,210.284294

31.480775 0.2041 6,500,561.501478

31.484177 0.2041 6,448,873.145835

31.487577 0.204 6,472,608.816931

31.490978 0.2041 6,467,776.030032

31.494380 0.2041 6,342,333.751965

31.497780 0.204 6,395,251.544543

31.501182 0.2041 6,442,689.955492

31.504583 0.2041 6,436,521.613087

31.507983 0.204 6,495,822.167209

31.511385 0.2041 6,612,086.311225

31.514787 0.2041 6,504,628.567634

31.518187 0.204 6,502,812.386450

31.521588 0.2041 6,656,560.194071

31.524990 0.2041 6,566,086.309410

31.528390 0.204 6,508,047.091088

31.531792 0.2041 6,534,880.154372

31.535193 0.2041 6,495,979.204096

31.538593 0.204 6,551,925.368828

31.541995 0.2041 6,608,862.734893

31.545397 0.2041 6,536,264.883421

31.548797 0.204 6,499,039.025745

31.552198 0.2041 6,500,405.584984

31.555600 0.2041 6,448,777.136823

31.559000 0.204 6,416,700.422583

31.562402 0.2041 6,374,074.043750

31.565803 0.2041 6,282,416.203376

31.569203 0.204 6,393,557.375561

31.572605 0.2041 6,529,507.083827

31.576007 0.2041 6,583,264.986103

31.579407 0.204 6,704,287.790196

31.582808 0.2041 6,828,984.120255

31.586210 0.2041 7,032,031.793428

31.589610 0.204 7,240,537.136609

31.593012 0.2041 7,193,640.441422

31.596413 0.2041 7,436,209.294686

31.599813 0.204 8,009,557.812934

31.603215 0.2041 8,231,959.389601

31.606617 0.2041 8,734,314.447380

31.610017 0.204 9,540,107.858847

31.613418 0.2041 10,058,746.234369

31.616820 0.2041 10,586,266.053835

31.620220 0.204 11,077,139.158159

31.623622 0.2041 11,179,086.122517

31.627023 0.2041 11,022,393.636328

31.630423 0.204 10,976,354.854368

31.633825 0.2041 10,723,608.211017

31.637227 0.2041 10,113,258.997441

31.640627 0.204 9,475,502.942340

31.644028 0.2041 8,943,297.180151

31.647430 0.2041 8,433,818.216132

31.650830 0.204 7,852,895.589317

31.654232 0.2041 7,345,988.487931

31.657633 0.2041 7,092,824.324482

31.661033 0.204 6,980,264.635883

31.664435 0.2041 6,790,309.789963

31.667837 0.2041 6,678,258.633259

31.671237 0.204 6,509,433.437555

31.674638 0.2041 6,434,100.685074

31.678040 0.2041 6,538,463.512970

31.681440 0.204 6,439,492.452322

31.684842 0.2041 6,261,225.113847

31.688243 0.2041 6,325,662.763883

31.691643 0.204 6,527,127.187823

31.695045 0.2041 6,444,763.799910

31.698447 0.2041 6,386,123.064158

31.701847 0.204 6,572,029.639542

31.705248 0.2041 6,525,779.704282

31.708650 0.2041 6,416,211.000829

31.712052 0.2041 6,463,673.756599

31.715452 0.204 6,452,502.802105

31.718853 0.2041 6,276,800.451153

31.722255 0.2041 6,233,354.389181

31.725655 0.204 6,425,645.827547

31.729057 0.2041 6,370,906.095157

31.732458 0.2041 6,407,310.562275

31.735858 0.204 6,554,299.652843

31.739260 0.2041 6,376,186.762997

31.742662 0.2041 6,479,934.888362

31.746062 0.204 6,777,699.908905

31.749463 0.2041 6,639,199.285313

31.752865 0.2041 6,620,760.335948

31.756265 0.204 6,758,272.582638

31.759667 0.2041 6,654,387.107097

31.763068 0.2041 6,635,502.908470

31.766468 0.204 6,638,633.354501

31.769870 0.2041 6,553,243.287209

31.773272 0.2041 6,508,300.108623

31.776672 0.204 6,496,569.449261

31.780073 0.2041 6,437,049.764555

31.783475 0.2041 6,434,166.564835

31.786875 0.204 6,515,083.857869

31.790277 0.2041 6,552,787.277719

31.793678 0.2041 6,530,313.560256

31.797078 0.204 6,436,555.733280

31.800480 0.2041 6,455,044.811798

31.803882 0.2041 6,477,139.020897

31.807282 0.204 6,351,439.641393

31.810683 0.2041 6,436,549.442914

31.814085 0.2041 6,488,259.681421

31.817485 0.204 6,528,497.425426

31.820887 0.2041 6,624,663.514506

31.824288 0.2041 6,519,930.952932

31.827688 0.204 6,483,966.873152

31.831090 0.2041 6,429,897.086930

31.834492 0.2041 6,388,073.568051

31.837892 0.204 6,497,273.341094

31.841293 0.2041 6,452,511.844688

31.844695 0.2041 6,314,581.660858

31.848095 0.204 6,400,303.575700

31.851497 0.2041 6,400,537.065677

31.854898 0.2041 6,375,251.434947

31.858298 0.204 6,475,583.287914

31.861700 0.2041 6,561,079.407218

31.865102 0.2041 6,749,514.025725

31.868502 0.204 6,750,934.975046

31.871903 0.2041 6,548,672.997486

31.875305 0.2041 6,490,622.915123

31.878705 0.204 6,609,024.917156

31.882107 0.2041 6,596,536.176400

31.885508 0.2041 6,478,849.093036

31.888908 0.204 6,559,125.456954

31.892310 0.2041 6,494,621.334113

31.895712 0.2041 6,479,386.460995

31.899112 0.204 6,600,772.109481

31.902513 0.2041 6,426,104.446853

31.905915 0.2041 6,482,390.177097

31.909315 0.204 6,695,658.113500

31.912717 0.2041 6,660,235.722554

31.916118 0.2041 6,548,784.579524

31.919518 0.204 6,510,378.442165

31.922920 0.2041 6,568,402.836722

31.926322 0.2041 6,464,719.663071

31.929722 0.204 6,351,857.846336

31.933123 0.2041 6,449,090.469101

31.936525 0.2041 6,505,635.715110

31.939925 0.204 6,330,093.393762

31.943327 0.2041 6,343,375.475285

31.946728 0.2041 6,494,066.777727

31.950128 0.204 6,417,619.386360

31.953530 0.2041 6,517,998.248506

31.956932 0.2041 6,684,010.661514

31.960332 0.204 6,507,927.223289

31.963733 0.2041 6,342,253.399380

31.967135 0.2041 6,351,492.906750

31.970535 0.204 6,350,796.545431

31.973937 0.2041 6,435,335.013502

31.977338 0.2041 6,421,615.927815

31.980738 0.204 6,365,652.589973

31.984140 0.2041 6,405,672.516236

31.987542 0.2041 6,334,465.289457

31.990942 0.204 6,162,338.625366

31.994343 0.2041 6,210,183.469378

31.997745 0.2041 6,391,097.604246

32.001145 0.204 6,274,452.682384

32.004547 0.2041 6,212,211.533617

32.007948 0.2041 6,284,708.069880

32.011348 0.204 6,209,533.402987

32.014750 0.2041 6,114,845.848750

32.018152 0.2041 6,257,344.087159

32.021552 0.204 6,348,852.990597

32.024953 0.2041 6,263,626.972949

32.028355 0.2041 6,357,598.733833

32.031755 0.204 6,398,196.772149

32.035157 0.2041 6,335,585.908969

32.038558 0.2041 6,423,083.503719

32.041958 0.204 6,689,461.384568

32.045360 0.2041 6,806,860.672523

32.048762 0.2041 6,812,060.085179

32.052163 0.2041 6,955,327.457131

32.055563 0.204 7,048,928.661634

32.058965 0.2041 7,090,999.448757

32.062367 0.2041 7,239,586.631685

32.065767 0.204 7,217,412.292828

32.069168 0.2041 6,979,173.499105

32.072570 0.2041 6,959,400.966258

32.075970 0.204 6,960,487.870803

32.079372 0.2041 6,652,051.551584

32.082773 0.2041 6,503,354.260484

32.086173 0.204 6,621,295.419423

32.089575 0.2041 6,474,532.006775

32.092977 0.2041 6,303,980.331734

32.096377 0.204 6,352,690.182154

32.099778 0.2041 6,462,543.452003

32.103180 0.2041 6,569,638.388276

32.106580 0.204 6,594,249.169245

32.109982 0.2041 6,548,688.944903

32.113383 0.2041 6,663,621.324482

32.116783 0.204 6,900,903.771575

32.120185 0.2041 6,794,495.030290

32.123587 0.2041 6,735,315.120815

32.126987 0.204 7,055,797.014862

32.130388 0.2041 7,052,496.846684

32.133790 0.2041 6,965,337.055542

32.137190 0.204 6,905,582.868194

32.140592 0.2041 6,690,327.000247

32.143993 0.2041 6,663,814.196730

32.147393 0.204 6,544,457.764563

32.150795 0.2041 6,463,003.849599

32.154197 0.2041 6,440,969.436110

32.157597 0.204 6,233,578.881914

32.160998 0.2041 6,190,770.316495

32.164400 0.2041 6,281,100.022020

32.167800 0.204 6,330,908.164329

32.171202 0.2041 6,476,100.500774

32.174603 0.2041 6,503,617.212170

32.178003 0.204 6,476,973.825141

32.181405 0.2041 6,526,234.829281

32.184807 0.2041 6,556,305.413291

32.188207 0.204 6,563,540.478369

32.191608 0.2041 6,493,624.298123

32.195010 0.2041 6,541,766.746651

32.198410 0.204 6,740,491.545209

32.201812 0.2041 6,850,901.043749

32.205213 0.2041 7,273,744.672941

32.208613 0.204 7,946,896.124755

32.212015 0.2041 8,942,439.158871

32.215417 0.2041 10,660,901.055507

32.218817 0.204 12,889,642.640709

32.222218 0.2041 15,671,277.535107

32.225620 0.2041 18,655,033.343387

32.229020 0.204 21,691,537.218212

32.232422 0.2041 24,368,695.956604

32.235823 0.2041 25,401,115.789983

32.239223 0.204 25,144,105.986971

32.242625 0.2041 24,552,714.245099

32.246027 0.2041 22,404,934.743848

32.249427 0.204 19,273,946.104477

32.252828 0.2041 16,452,294.714118

32.256230 0.2041 13,652,551.702004

32.259630 0.204 11,448,194.754955

32.263032 0.2041 9,638,476.502119

32.266433 0.2041 8,360,306.991205

32.269833 0.204 7,799,807.821239

32.273235 0.2041 7,419,672.718668

32.276637 0.2041 7,053,091.342300

32.280037 0.204 6,753,163.866262

32.283438 0.2041 6,972,846.399738

32.286840 0.2041 7,011,936.137816

32.290240 0.204 6,787,081.549894

32.293642 0.2041 6,875,304.127274

32.297043 0.2041 6,713,147.838699

32.300443 0.204 6,636,368.037592

32.303845 0.2041 6,659,578.347649

32.307247 0.2041 6,579,570.705958

32.310647 0.204 6,660,315.581679

32.314048 0.2041 6,566,355.350162

32.317450 0.2041 6,569,789.848598

32.320850 0.204 6,704,300.328249

32.324252 0.2041 6,691,553.074148

32.327653 0.2041 6,627,387.314337

32.331053 0.204 6,426,064.499003

32.334455 0.2041 6,339,345.465266

32.337857 0.2041 6,376,701.778193

32.341257 0.204 6,459,548.945355

32.344658 0.2041 6,634,629.180177

32.348060 0.2041 6,548,726.041577

32.351460 0.204 6,381,226.586015

32.354862 0.2041 6,427,311.837744

32.358263 0.2041 6,422,345.189251

32.361663 0.204 6,357,974.616030

32.365065 0.2041 6,408,918.791556

32.368467 0.2041 6,468,414.686437

32.371867 0.204 6,486,845.981391

32.375268 0.2041 6,521,190.633989

32.378670 0.2041 6,560,089.467945

32.382070 0.204 6,587,123.926138

32.385472 0.2041 6,522,210.484677

32.388873 0.2041 6,498,461.194931

32.392273 0.204 6,577,918.261378

32.395675 0.2041 6,556,027.616771

32.399077 0.2041 6,553,361.584078

32.402477 0.204 6,627,553.173607

32.405878 0.2041 6,620,651.236489

32.409280 0.2041 6,554,130.316542

32.412682 0.2041 6,516,472.273000

32.416082 0.204 6,572,163.700610

32.419483 0.2041 6,486,355.122498

32.422885 0.2041 6,413,581.289355

32.426285 0.204 6,565,174.053709

32.429687 0.2041 6,426,308.493860

32.433088 0.2041 6,457,804.585775

32.436488 0.204 6,591,941.016935

32.439890 0.2041 6,347,471.354117

32.443292 0.2041 6,304,383.028163

32.446692 0.204 6,370,312.108229

32.450093 0.2041 6,428,134.673065

32.453495 0.2041 6,348,438.301442

32.456895 0.204 6,482,795.014357

32.460297 0.2041 6,922,695.865506

32.463698 0.2041 7,176,531.936944

32.467098 0.204 7,572,176.014468

32.470500 0.2041 8,286,794.757964

32.473902 0.2041 9,153,866.468508

32.477302 0.204 10,052,048.199383

32.480703 0.2041 11,060,306.322287

32.484105 0.2041 12,033,527.987780

32.487505 0.204 12,569,925.187700

32.490907 0.2041 12,773,619.005020

32.494308 0.2041 12,693,764.743156

32.497708 0.204 12,144,052.361970

32.501110 0.2041 11,256,600.350865

32.504512 0.2041 10,479,471.946802

32.507912 0.204 9,631,428.235539

32.511313 0.2041 8,614,805.238212

32.514715 0.2041 8,204,167.543881

32.518115 0.204 7,968,306.274743

32.521517 0.2041 7,483,551.758740

32.524918 0.2041 7,174,062.987246

32.528318 0.204 7,041,298.534297

32.531720 0.2041 6,961,409.428157

32.535122 0.2041 7,007,630.172377

32.538522 0.204 7,081,363.497646

32.541923 0.2041 7,016,445.207093

32.545325 0.2041 7,143,004.459601

32.548725 0.204 7,236,917.223348

32.552127 0.2041 7,183,518.884058

32.555528 0.2041 7,199,989.847448

32.558928 0.204 7,187,830.794718

32.562330 0.2041 7,232,666.266055

32.565732 0.2041 7,159,784.359151

32.569132 0.204 7,127,252.884710

32.572533 0.2041 7,119,674.626089

32.575935 0.2041 7,007,554.060354

32.579335 0.204 7,149,538.036356

32.582737 0.2041 7,361,140.271272

32.586138 0.2041 7,268,268.428462

32.589538 0.204 7,088,363.985810

32.592940 0.2041 7,250,619.353906

32.596342 0.2041 7,377,815.175446

32.599742 0.204 7,243,855.905265

32.603143 0.2041 7,347,386.321983

32.606545 0.2041 7,542,755.703610

32.609945 0.204 7,439,236.567988

32.613347 0.2041 7,348,814.986173

32.616748 0.2041 7,450,184.708375

32.620148 0.204 7,406,135.129739

32.623550 0.2041 7,352,503.339532

32.626952 0.2041 7,338,231.966080

32.630352 0.204 7,169,856.182678

32.633753 0.2041 7,080,054.989413

32.637155 0.2041 7,073,254.876366

32.640555 0.204 7,217,281.386222

32.643957 0.2041 7,399,916.511956

32.647358 0.2041 7,289,310.539746

32.650758 0.204 7,155,600.813444

32.654160 0.2041 7,011,252.625567

32.657562 0.2041 6,941,013.937840

32.660962 0.204 7,066,238.765542

32.664363 0.2041 6,847,534.412530

32.667765 0.2041 6,756,324.711241

32.671165 0.204 6,962,278.718019

32.674567 0.2041 6,868,996.175118

32.677968 0.2041 6,668,523.721131

32.681368 0.204 6,579,979.131006

32.684770 0.2041 6,611,677.687772

32.688172 0.2041 6,550,148.345282

32.691572 0.204 6,482,050.911306

32.694973 0.2041 6,565,090.912560

32.698375 0.2041 6,610,145.499676

32.701775 0.204 6,576,157.850632

32.705177 0.2041 6,651,461.747926

32.708578 0.2041 6,726,894.450562

32.711978 0.204 6,643,218.547652

32.715380 0.2041 6,522,419.736657

32.718782 0.2041 6,497,696.004565

32.722182 0.204 6,620,876.259305

32.725583 0.2041 6,674,119.795406

32.728985 0.2041 6,534,097.899071

32.732385 0.204 6,522,018.855943

32.735787 0.2041 6,618,511.588394

32.739188 0.2041 6,557,462.314346

32.742588 0.204 6,554,953.676482

32.745990 0.2041 6,472,382.633565

32.749392 0.2041 6,311,716.850642

32.752792 0.204 6,467,350.026968

32.756193 0.2041 6,539,066.101255

32.759595 0.2041 6,380,104.503443

32.762997 0.2041 6,393,823.735011

32.766397 0.204 6,481,706.346430

32.769798 0.2041 6,445,195.800205

32.773200 0.2041 6,341,905.541744

32.776600 0.204 6,365,717.429249

32.780002 0.2041 6,396,327.880681

32.783403 0.2041 6,504,126.520966

32.786803 0.204 6,543,571.300763

32.790205 0.2041 6,344,602.071709

32.793607 0.2041 6,300,558.515268

32.797007 0.204 6,328,931.721855

32.800408 0.2041 6,457,889.286157

32.803810 0.2041 6,521,378.647878

32.807210 0.204 6,381,803.422758

32.810612 0.2041 6,354,829.052700

32.814013 0.2041 6,396,070.482045

32.817413 0.204 6,354,456.212725

32.820815 0.2041 6,230,729.183514

32.824217 0.2041 6,304,520.163411

32.827617 0.204 6,355,669.438534

32.831018 0.2041 6,282,117.754567

32.834420 0.2041 6,404,742.598730

32.837820 0.204 6,559,669.808955

32.841222 0.2041 6,531,397.088136

32.844623 0.2041 6,348,815.192777

32.848023 0.204 6,356,085.542301

32.851425 0.2041 6,474,803.587375

32.854827 0.2041 6,678,741.603594

32.858227 0.204 6,687,060.807084

32.861628 0.2041 6,447,559.318204

32.865030 0.2041 6,648,669.052233

32.868430 0.204 6,733,362.492126

32.871832 0.2041 6,687,755.919303

32.875233 0.2041 6,904,549.575166

32.878633 0.204 6,744,920.545529

32.882035 0.2041 6,472,663.200345

32.885437 0.2041 6,541,497.043217

32.888837 0.204 6,460,658.987482

32.892238 0.2041 6,494,651.517301

32.895640 0.2041 6,657,204.932720

32.899040 0.204 6,579,428.327591

32.902442 0.2041 6,647,401.582628

32.905843 0.2041 6,689,144.968194

32.909243 0.204 6,664,642.359653

32.912645 0.2041 6,729,587.010565

32.916047 0.2041 6,857,078.149468

32.919447 0.204 6,911,090.042062

32.922848 0.2041 6,991,058.024891

32.926250 0.2041 7,241,004.893216

32.929650 0.204 7,352,989.062523

32.933052 0.2041 7,432,501.876218

32.936453 0.2041 7,587,355.051183

32.939853 0.204 7,634,743.269132

32.943255 0.2041 7,829,977.355927

32.946657 0.2041 8,009,942.896584

32.950057 0.204 8,057,879.038088

32.953458 0.2041 8,081,378.148708

32.956860 0.2041 7,720,886.680866

32.960260 0.204 7,415,156.686434

32.963662 0.2041 7,349,039.815584

32.967063 0.2041 7,205,476.982829

32.970463 0.204 7,085,894.617503

32.973865 0.2041 6,939,170.673820

32.977267 0.2041 6,767,493.336489

32.980667 0.204 6,644,621.189911

32.984068 0.2041 6,649,921.005141

32.987470 0.2041 6,633,730.606595

32.990870 0.204 6,453,016.409182

32.994272 0.2041 6,482,926.691322

32.997673 0.2041 6,642,238.172508

33.001073 0.204 6,561,929.740886

33.004475 0.2041 6,345,014.381184

33.007877 0.2041 6,332,541.625843

33.011277 0.204 6,457,701.221311

33.014678 0.2041 6,495,733.634629

33.018080 0.2041 6,501,101.260629

33.021480 0.204 6,533,326.697230

33.024882 0.2041 6,502,302.707969

33.028283 0.2041 6,468,012.485003

33.031683 0.204 6,571,685.499197

33.035085 0.2041 6,565,066.178825

33.038487 0.2041 6,530,055.123627

33.041887 0.204 6,549,013.404614

33.045288 0.2041 6,469,233.616064

33.048690 0.2041 6,460,350.394251

33.052090 0.204 6,496,523.711745

33.055492 0.2041 6,343,961.312508

33.058893 0.2041 6,492,053.171126

33.062293 0.204 6,811,843.595590

33.065695 0.2041 6,925,750.907355

33.069097 0.2041 7,389,544.843056

33.072497 0.204 8,053,311.123581

33.075898 0.2041 8,930,584.024298

33.079300 0.2041 10,048,947.168706

33.082700 0.204 11,246,425.971282

33.086102 0.2041 12,363,433.127596

33.089503 0.2041 13,244,977.827167

33.092903 0.204 14,273,186.483702

33.096305 0.2041 14,838,095.563706

33.099707 0.2041 14,385,902.452211

33.103107 0.204 13,650,026.324197

33.106508 0.2041 12,692,030.586014

33.109910 0.2041 11,478,408.561006

33.113312 0.2041 10,303,760.204776

33.116712 0.204 9,150,033.914668

33.120113 0.2041 8,336,420.246256

33.123515 0.2041 7,725,256.400019

33.126915 0.204 7,284,623.384135

33.130317 0.2041 7,118,330.757905

33.133718 0.2041 6,973,867.589237

33.137118 0.204 6,875,624.375221

33.140520 0.2041 6,758,919.280481

33.143922 0.2041 6,647,147.940289

33.147322 0.204 6,654,278.504919

33.150723 0.2041 6,643,144.188676

33.154125 0.2041 6,574,758.386907

33.157525 0.204 6,635,259.902898

33.160927 0.2041 6,765,591.230225

33.164328 0.2041 6,791,666.855476

33.167728 0.204 6,837,595.261759

33.171130 0.2041 6,946,863.484407

33.174532 0.2041 6,896,903.699341

33.177932 0.204 6,934,728.536557

33.181333 0.2041 7,033,085.123859

33.184735 0.2041 7,019,406.294353

33.188135 0.204 7,002,021.839846

33.191537 0.2041 6,922,941.426778

33.194938 0.2041 6,869,606.163840

33.198338 0.204 6,749,794.432508

33.201740 0.2041 6,612,591.151541

33.205142 0.2041 6,680,969.343194

33.208542 0.204 6,792,404.728998

33.211943 0.2041 6,666,643.740203

33.215345 0.2041 6,551,615.626751

33.218745 0.204 6,606,323.785410

33.222147 0.2041 6,693,302.985611

33.225548 0.2041 6,669,684.776383

33.228948 0.204 6,564,592.079960

33.232350 0.2041 6,499,950.579939

33.235752 0.2041 6,570,983.418174

33.239152 0.204 6,571,684.117200

33.242553 0.2041 6,419,533.242116

33.245955 0.2041 6,496,206.409852

33.249355 0.204 6,436,164.520028

33.252757 0.2041 6,363,281.737909

33.256158 0.2041 6,352,728.765289

33.259558 0.204 6,330,622.597205

33.262960 0.2041 6,462,541.276864

33.266362 0.2041 6,476,617.792026

33.269762 0.204 6,475,608.515921

33.273163 0.2041 6,479,555.929510

33.276565 0.2041 6,425,422.141111

33.279965 0.204 6,359,827.783887

33.283367 0.2041 6,447,172.039842

33.286768 0.2041 6,455,369.590592

33.290168 0.204 6,493,659.122212

33.293570 0.2041 6,724,312.155469

33.296972 0.2041 6,795,469.930762

33.300372 0.204 6,958,434.751056

33.303773 0.2041 6,973,459.981579

33.307175 0.2041 6,985,371.677929

33.310575 0.204 7,193,263.521318

33.313977 0.2041 7,195,276.062093

33.317378 0.2041 7,106,331.662642

33.320778 0.204 6,961,110.844291

33.324180 0.2041 6,845,276.306196

33.327582 0.2041 6,730,991.861789

33.330982 0.204 6,609,358.883541

33.334383 0.2041 6,643,061.553399

33.337785 0.2041 6,586,871.626655

33.341185 0.204 6,447,361.022152

33.344587 0.2041 6,403,362.653278

33.347988 0.2041 6,376,201.495368

33.351388 0.204 6,323,611.215619

33.354790 0.2041 6,384,694.383260

33.358192 0.2041 6,495,814.629381

33.361592 0.204 6,443,150.293238

33.364993 0.2041 6,405,736.473711

33.368395 0.2041 6,391,827.513249

33.371795 0.204 6,321,554.588112

33.375197 0.2041 6,383,832.557194

33.378598 0.2041 6,492,269.020971

33.381998 0.204 6,400,972.776452

33.385400 0.2041 6,402,213.635284

33.388802 0.2041 6,455,276.796082

33.392202 0.204 6,325,580.175443

33.395603 0.2041 6,425,582.829678

33.399005 0.2041 6,497,044.267458

33.402405 0.204 6,412,437.721950

33.405807 0.2041 6,567,720.824251

33.409208 0.2041 6,698,866.244866

33.412608 0.204 6,922,794.626979

33.416010 0.2041 7,273,613.582253

33.419412 0.2041 7,539,805.633157

33.422812 0.204 7,866,609.334267

33.426213 0.2041 8,109,598.432022

33.429615 0.2041 8,386,585.589083

33.433015 0.204 8,685,958.379901

33.436417 0.2041 8,412,810.920388

33.439818 0.2041 7,937,220.421687

33.443218 0.204 7,732,226.040121

33.446620 0.2041 7,535,440.890935

33.450022 0.2041 7,343,535.115638

33.453422 0.204 6,954,342.007206

33.456823 0.2041 6,731,095.923472

33.460225 0.2041 6,886,367.251972

33.463625 0.204 6,689,015.488426

33.467027 0.2041 6,450,159.722446

33.470428 0.2041 6,444,671.738739

33.473830 0.2041 6,456,450.641489

33.477230 0.204 6,514,534.498223

33.480632 0.2041 6,502,069.333348

33.484033 0.2041 6,502,454.592723

33.487433 0.204 6,633,326.353096

33.490835 0.2041 6,705,066.618755

33.494237 0.2041 6,806,813.327151

33.497637 0.204 6,804,016.294746

33.501038 0.2041 6,657,645.293887

33.504440 0.2041 6,737,975.167783

33.507840 0.204 6,691,219.310317

33.511242 0.2041 6,715,376.432315

33.514643 0.2041 6,819,265.468842

33.518043 0.204 6,625,126.419698

33.521445 0.2041 6,513,580.934243

33.524847 0.2041 6,395,486.073505

33.528247 0.204 6,446,292.656496

33.531648 0.2041 6,595,397.359878

33.535050 0.2041 6,617,039.818357

33.538450 0.204 6,659,906.079869

33.541852 0.2041 6,621,176.824751

33.545253 0.2041 6,601,100.461634

33.548653 0.204 6,547,366.645170

33.552055 0.2041 6,487,775.897925

33.555457 0.2041 6,590,293.560364

33.558857 0.204 6,638,049.832653

33.562258 0.2041 6,535,236.951618

33.565660 0.2041 6,614,312.553770

33.569060 0.204 6,648,589.449503

33.572462 0.2041 6,443,669.651047

33.575863 0.2041 6,496,875.579820

33.579263 0.204 6,507,280.676900

33.582665 0.2041 6,347,819.976528

33.586067 0.2041 6,364,189.848401

33.589467 0.204 6,229,961.306973

33.592868 0.2041 6,309,483.090495

33.596270 0.2041 6,595,398.599840

33.599670 0.204 6,379,046.757999

33.603072 0.2041 6,220,859.637431

33.606473 0.2041 6,310,859.333436

33.609873 0.204 6,214,282.835150

33.613275 0.2041 6,323,697.400706

33.616677 0.2041 6,492,127.183675

33.620077 0.204 6,430,695.685747

33.623478 0.2041 6,369,870.989626

33.626880 0.2041 6,326,985.759697

33.630280 0.204 6,390,499.541307

33.633682 0.2041 6,450,072.830000

33.637083 0.2041 6,408,541.069913

33.640483 0.204 6,381,316.407512

33.643885 0.2041 6,448,818.832238

33.647287 0.2041 6,598,946.115510

33.650687 0.204 6,589,267.983844

33.654088 0.2041 6,578,660.929269

33.657490 0.2041 6,720,686.597452

33.660890 0.204 6,666,153.602784

33.664292 0.2041 6,383,306.788793

33.667693 0.2041 6,361,725.840523

33.671093 0.204 6,529,289.332005

33.674495 0.2041 6,586,190.002085

33.677897 0.2041 6,547,302.670664

33.681297 0.204 6,340,152.005418

33.684698 0.2041 6,378,502.515077

33.688100 0.2041 6,436,199.958640

33.691500 0.204 6,345,868.838726

33.694902 0.2041 6,491,180.779338

33.698303 0.2041 6,610,140.777124

33.701703 0.204 6,615,889.104780

33.705105 0.2041 6,653,887.443816

33.708507 0.2041 6,647,220.112280

33.711907 0.204 6,681,064.537677

33.715308 0.2041 6,895,558.920827

33.718710 0.2041 6,835,760.005411

33.722110 0.204 6,629,477.783994

33.725512 0.2041 6,620,467.622333

33.728913 0.2041 6,648,058.606306

33.732313 0.204 6,690,465.992937

33.735715 0.2041 6,606,987.166878

33.739117 0.2041 6,415,932.980937

33.742517 0.204 6,374,212.963696

33.745918 0.2041 6,509,313.669489

33.749320 0.2041 6,510,608.564488

33.752720 0.204 6,408,545.186774

33.756122 0.2041 6,353,430.521851

33.759523 0.2041 6,364,994.838506

33.762923 0.204 6,255,347.677169

33.766325 0.2041 6,144,210.792664

33.769727 0.2041 6,274,744.298027

33.773127 0.204 6,337,522.998406

33.776528 0.2041 6,300,809.331309

33.779930 0.2041 6,307,664.918222

33.783330 0.204 6,396,501.831502

33.786732 0.2041 6,524,018.742791

33.790133 0.2041 6,751,838.434276

33.793533 0.204 6,866,799.350619

33.796935 0.2041 7,089,963.523549

33.800337 0.2041 7,624,544.878993

33.803737 0.204 8,111,964.940330

33.807138 0.2041 8,588,929.843404

33.810540 0.2041 8,977,702.129888

33.813942 0.2041 9,426,778.858408

33.817342 0.204 9,820,718.807422

33.820743 0.2041 9,645,507.128251

33.824145 0.2041 9,262,038.958053

33.827545 0.204 8,997,356.665575

33.830947 0.2041 8,529,687.273800

33.834348 0.2041 7,996,158.288863

33.837748 0.204 7,606,581.558421

33.841150 0.2041 7,293,308.359188

33.844552 0.2041 6,923,240.719460

33.847952 0.204 6,643,465.276837

33.851353 0.2041 6,683,115.105240

33.854755 0.2041 6,642,885.551602

33.858155 0.204 6,382,873.940012

33.861557 0.2041 6,273,463.590572

33.864958 0.2041 6,368,729.146874

33.868358 0.204 6,435,871.708401

33.871760 0.2041 6,379,260.583390

33.875162 0.2041 6,379,386.650747

33.878562 0.204 6,365,479.822222

33.881963 0.2041 6,378,286.148609

33.885365 0.2041 6,407,688.852605

33.888765 0.204 6,245,329.797567

33.892167 0.2041 6,233,563.024367

33.895568 0.2041 6,297,036.962407

33.898968 0.204 6,332,061.598912

33.902370 0.2041 6,228,961.729999

33.905772 0.2041 6,091,234.900846

33.909172 0.204 6,259,428.170451

33.912573 0.2041 6,289,261.402722

33.915975 0.2041 6,192,192.263524

33.919375 0.204 6,179,059.967542

33.922777 0.2041 6,118,146.697722

33.926178 0.2041 6,143,589.696402

33.929578 0.204 6,209,366.609548

33.932980 0.2041 6,297,890.554625

33.936382 0.2041 6,413,801.320309

33.939782 0.204 6,332,209.399457

33.943183 0.2041 6,211,200.788816

33.946585 0.2041 6,390,584.142384

33.949985 0.204 6,611,176.952407

33.953387 0.2041 6,628,816.960627

33.956788 0.2041 6,692,225.587819

33.960188 0.204 6,825,038.785302

33.963590 0.2041 6,962,144.872170

33.966992 0.2041 7,092,471.017406

33.970392 0.204 7,193,068.316811

33.973793 0.2041 7,230,266.353331

33.977195 0.2041 7,255,060.522581

33.980595 0.204 7,146,379.955842

33.983997 0.2041 6,948,096.787069

33.987398 0.2041 6,832,575.299034

33.990798 0.204 6,627,111.728768

33.994200 0.2041 6,497,215.399244

33.997602 0.2041 6,560,863.299914

34.001002 0.204 6,587,037.362408

34.004403 0.2041 6,359,475.694256

34.007805 0.2041 6,176,567.714624

34.011205 0.204 6,217,535.250904

34.014607 0.2041 6,251,386.194728

34.018008 0.2041 6,357,366.960529

34.021408 0.204 6,483,085.218007

34.024810 0.2041 6,363,302.034767

34.028212 0.2041 6,134,181.033843

34.031612 0.204 6,187,004.515913

34.035013 0.2041 6,415,621.580604

34.038415 0.2041 6,490,667.336809

34.041815 0.204 6,490,738.867618

34.045217 0.2041 6,607,248.364504

34.048618 0.2041 6,745,852.129797

34.052018 0.204 6,800,078.033641

34.055420 0.2041 7,169,451.878360

34.058822 0.2041 7,566,423.335255

34.062222 0.204 7,842,450.242811

34.065623 0.2041 8,429,350.650319

34.069025 0.2041 9,248,478.109621

34.072425 0.204 10,085,284.256125

34.075827 0.2041 10,728,957.250492

34.079228 0.2041 11,184,580.585131

34.082628 0.204 11,368,686.495048

34.086030 0.2041 11,663,328.276049

34.089432 0.2041 11,589,440.341563

34.092832 0.204 10,851,364.238729

34.096233 0.2041 10,282,671.213291

34.099635 0.2041 9,829,625.762189

34.103035 0.204 9,021,281.816633

34.106437 0.2041 8,258,192.403191

34.109838 0.2041 8,201,958.158448

34.113238 0.204 7,934,928.501413

34.116640 0.2041 7,718,443.909557

34.120042 0.2041 7,898,097.841061

34.123442 0.204 7,686,407.009138

34.126843 0.2041 7,624,254.120684

34.130245 0.2041 7,592,526.940491

34.133645 0.204 7,353,739.969235

34.137047 0.2041 7,316,064.991350

34.140448 0.2041 7,227,634.684004

34.143848 0.204 7,097,770.391706

34.147250 0.2041 7,068,832.860785

34.150652 0.2041 7,004,896.825450

34.154052 0.204 6,899,051.625000

34.157453 0.2041 6,840,598.766549

34.160855 0.2041 6,811,357.632941

34.164257 0.2041 6,761,222.632878

34.167657 0.204 6,636,708.200620

34.171058 0.2041 6,549,979.544861

34.174460 0.2041 6,610,119.492093

34.177860 0.204 6,616,788.704635

34.181262 0.2041 6,559,526.501714

34.184663 0.2041 6,604,152.723152

34.188063 0.204 6,693,428.286959

34.191465 0.2041 6,603,555.739757

34.194867 0.2041 6,515,166.857013

34.198267 0.204 6,504,777.837035

34.201668 0.2041 6,524,912.884145

34.205070 0.2041 6,554,288.350758

34.208470 0.204 6,445,167.741246

34.211872 0.2041 6,389,179.700357

34.215273 0.2041 6,477,873.834007

34.218673 0.204 6,470,285.219030

34.222075 0.2041 6,462,715.638360

34.225477 0.2041 6,570,722.602391

34.228877 0.204 6,534,305.279310

34.232278 0.2041 6,396,795.858787

34.235680 0.2041 6,495,681.387125

34.239080 0.204 6,456,661.047507

34.242482 0.2041 6,382,659.227394

34.245883 0.2041 6,579,598.425965

34.249283 0.204 6,502,095.292879

34.252685 0.2041 6,388,118.094062

34.256087 0.2041 6,438,961.240609

34.259487 0.204 6,453,812.388112

34.262888 0.2041 6,484,097.778968

34.266290 0.2041 6,582,131.020121

34.269690 0.204 6,585,651.771750

34.273092 0.2041 6,435,977.665399

34.276493 0.2041 6,426,272.594996

34.279893 0.204 6,402,554.130931

34.283295 0.2041 6,414,271.941338

34.286697 0.2041 6,511,688.772652

34.290097 0.204 6,511,078.548742

34.293498 0.2041 6,423,892.932890

34.296900 0.2041 6,263,456.253128

34.300300 0.204 6,323,828.264337

34.303702 0.2041 6,490,185.459927

34.307103 0.2041 6,447,729.461264

34.310503 0.204 6,368,301.209883

34.313905 0.2041 6,342,085.204657

34.317307 0.2041 6,332,098.422578

34.320707 0.204 6,385,939.240723

34.324108 0.2041 6,319,800.657618

34.327510 0.2041 6,262,725.970328

34.330910 0.204 6,373,985.250103

34.334312 0.2041 6,390,352.891186

34.337713 0.2041 6,358,201.258514

34.341113 0.204 6,277,641.649217

34.344515 0.2041 6,286,552.499405

34.347917 0.2041 6,280,964.640268

34.351317 0.204 6,277,043.629521

34.354718 0.2041 6,408,230.155391

34.358120 0.2041 6,402,125.293015

34.361520 0.204 6,344,502.216202

34.364922 0.2041 6,285,928.819689

34.368323 0.2041 6,438,185.244760

34.371723 0.204 6,563,625.385978

34.375125 0.2041 6,546,645.739912

34.378527 0.2041 6,650,684.043106

34.381927 0.204 6,569,188.888223

34.385328 0.2041 6,463,174.070150

34.388730 0.2041 6,634,131.985598

34.392130 0.204 6,745,011.671191

34.395532 0.2041 6,616,589.791282

34.398933 0.2041 6,587,452.060404

34.402333 0.204 6,721,929.259857

34.405735 0.2041 6,640,965.871881

34.409137 0.2041 6,604,567.397697

34.412537 0.204 6,745,821.762489

34.415938 0.2041 6,821,182.503731

34.419340 0.2041 6,857,980.166813

34.422740 0.204 6,837,773.097943

34.426142 0.2041 6,814,177.632706

34.429543 0.2041 6,856,189.731448

34.432943 0.204 7,008,040.584858

34.436345 0.2041 6,721,003.119373

34.439747 0.2041 6,411,208.883175

34.443147 0.204 6,568,888.622980

34.446548 0.2041 6,644,372.966067

34.449950 0.2041 6,577,075.953749

34.453350 0.204 6,499,031.822391

34.456752 0.2041 6,452,484.311736

34.460153 0.2041 6,457,697.132765

34.463553 0.204 6,429,967.966743

34.466955 0.2041 6,397,141.439886

34.470357 0.2041 6,502,390.026246

34.473757 0.204 6,494,458.046516

34.477158 0.2041 6,390,331.033968

34.480560 0.2041 6,440,176.489493

34.483960 0.204 6,517,800.412052

34.487362 0.2041 6,483,402.198972

34.490763 0.2041 6,404,011.609141

34.494163 0.204 6,398,627.504453

34.497565 0.2041 6,539,690.438968

34.500967 0.2041 6,535,913.830245

34.504367 0.204 6,388,941.513724

34.507768 0.2041 6,535,166.033380

34.511170 0.2041 6,752,200.381327

34.514570 0.204 6,817,539.623059

34.517972 0.2041 6,860,978.447958

34.521373 0.2041 6,757,960.231791

34.524775 0.2041 6,743,572.699731

34.528175 0.204 6,900,871.457132

34.531577 0.2041 6,733,796.435586

34.534978 0.2041 6,621,787.630062

34.538378 0.204 6,694,959.704786

34.541780 0.2041 6,551,778.284163

34.545182 0.2041 6,499,042.908404

34.548582 0.204 6,622,153.448359

34.551983 0.2041 6,602,736.911696

34.555385 0.2041 6,544,281.604379

34.558785 0.204 6,605,661.419090

34.562187 0.2041 6,535,270.258790

34.565588 0.2041 6,399,038.967729

34.568988 0.204 6,448,158.320081

34.572390 0.2041 6,475,519.798982

34.575792 0.2041 6,617,815.990180

34.579192 0.204 6,763,267.012487

34.582593 0.2041 6,673,909.278749

34.585995 0.2041 6,721,679.363118

34.589395 0.204 6,789,921.296584

34.592797 0.2041 6,742,091.973183

34.596198 0.2041 6,675,018.891560

34.599598 0.204 6,524,513.807574

34.603000 0.2041 6,413,605.603674

34.606402 0.2041 6,342,574.646781

34.609802 0.204 6,165,110.843640

34.613203 0.2041 6,322,145.792005

34.616605 0.2041 6,434,129.363572

34.620005 0.204 6,296,894.710701

34.623407 0.2041 6,437,981.909946

34.626808 0.2041 6,568,453.906363

34.630208 0.204 6,495,956.888500

34.633610 0.2041 6,416,251.071432

34.637012 0.2041 6,573,385.404298

34.640412 0.204 6,717,185.746918

34.643813 0.2041 6,707,447.180721

34.647215 0.2041 6,744,709.634109

34.650615 0.204 6,830,986.164861

34.654017 0.2041 6,763,931.756648

34.657418 0.2041 6,796,088.997591

34.660818 0.204 6,955,508.287541

34.664220 0.2041 6,855,512.612354

34.667622 0.2041 6,765,911.806777

34.671022 0.204 6,717,520.420830

34.674423 0.2041 6,678,614.211491

34.677825 0.2041 6,628,449.022884

34.681225 0.204 6,496,929.478149

34.684627 0.2041 6,452,433.054722

34.688028 0.2041 6,511,398.723937

34.691428 0.204 6,514,788.547645

34.694830 0.2041 6,375,030.096144

34.698232 0.2041 6,271,449.395770

34.701632 0.204 6,427,770.740905

34.705033 0.2041 6,639,114.878507

34.708435 0.2041 6,491,000.076513

34.711835 0.204 6,322,687.874689

34.715237 0.2041 6,497,383.300788

34.718638 0.2041 6,501,290.304134

34.722038 0.204 6,450,480.190036

34.725440 0.2041 6,733,597.833518

34.728842 0.2041 6,813,336.888507

34.732242 0.204 6,655,145.259358

34.735643 0.2041 6,667,725.062918

34.739045 0.2041 6,753,511.410683

34.742445 0.204 6,933,168.199541

34.745847 0.2041 7,129,176.294110

34.749248 0.2041 7,095,719.737498

34.752648 0.204 7,040,694.127099

34.756050 0.2041 6,938,107.195128

34.759452 0.2041 6,776,780.664851

34.762852 0.204 6,805,199.038252

34.766253 0.2041 6,711,367.790727

34.769655 0.2041 6,513,047.905081

34.773055 0.204 6,563,181.899313

34.776457 0.2041 6,568,121.813257

34.779858 0.2041 6,507,860.593992

34.783258 0.204 6,585,529.119505

34.786660 0.2041 6,505,660.284311

34.790062 0.2041 6,351,327.822321

34.793462 0.204 6,449,212.849562

34.796863 0.2041 6,500,969.145797

34.800265 0.2041 6,451,948.306793

34.803665 0.204 6,504,702.889306

34.807067 0.2041 6,524,891.520775

34.810468 0.2041 6,564,838.768816

34.813868 0.204 6,512,870.131689

34.817270 0.2041 6,386,556.015744

34.820672 0.2041 6,421,456.000830

34.824072 0.204 6,465,597.103329

34.827473 0.2041 6,429,891.943604

34.830875 0.2041 6,402,601.442966

34.834275 0.204 6,441,574.239935

34.837677 0.2041 6,496,604.229452

34.841078 0.2041 6,462,682.527176

34.844478 0.204 6,431,926.588236

34.847880 0.2041 6,544,235.390064

34.851282 0.2041 6,731,807.918011

34.854682 0.204 6,770,930.649454

34.858083 0.2041 6,725,325.717946

34.861485 0.2041 6,689,121.312765

34.864885 0.204 6,648,912.393335

34.868287 0.2041 6,674,523.789815

34.871688 0.2041 6,626,693.219427

34.875088 0.204 6,497,928.661192

34.878490 0.2041 6,553,387.114157

34.881892 0.2041 6,552,090.994990

34.885293 0.2041 6,503,774.635536

34.888693 0.204 6,632,077.417255

34.892095 0.2041 6,636,868.242661

34.895497 0.2041 6,603,578.285892

34.898897 0.204 6,563,463.405085

34.902298 0.2041 6,516,684.545932

34.905700 0.2041 6,504,177.777124

34.909100 0.204 6,588,088.762244

34.912502 0.2041 6,660,011.460674

34.915903 0.2041 6,571,276.311695

34.919303 0.204 6,569,338.833044

34.922705 0.2041 6,509,936.976676

34.926107 0.2041 6,465,418.007546

34.929507 0.204 6,534,858.616935

34.932908 0.2041 6,527,199.178487

34.936310 0.2041 6,591,749.969474

34.939710 0.204 6,595,762.067298

34.943112 0.2041 6,471,019.426124

34.946513 0.2041 6,294,659.663376

34.949913 0.204 6,401,839.098546

34.953315 0.2041 6,669,026.252623

34.956717 0.2041 6,561,967.647301

34.960117 0.204 6,325,602.948311

34.963518 0.2041 6,232,036.997957

34.966920 0.2041 6,393,964.481768

34.970320 0.204 6,420,796.726700

34.973722 0.2041 6,368,734.827018

34.977123 0.2041 6,437,659.038983

34.980523 0.204 6,333,554.768089

34.983925 0.2041 6,396,678.884057

34.987327 0.2041 6,474,149.053513

34.990727 0.204 6,347,822.870538

34.994128 0.2041 6,396,367.949145

34.997530 0.2041 6,636,935.711770

35.000930 0.204 6,776,160.496731

35.004332 0.2041 6,769,857.831337

35.007733 0.2041 6,795,677.780670

35.011133 0.204 6,867,854.386328

35.014535 0.2041 6,958,497.299628

35.017937 0.2041 6,893,925.292895

35.021337 0.204 6,923,847.731811

35.024738 0.2041 7,117,969.975593

35.028140 0.2041 7,160,591.279750

35.031540 0.204 7,167,666.029507

35.034942 0.2041 7,100,175.120637

35.038343 0.2041 7,094,732.588871

35.041743 0.204 6,957,046.357507

35.045145 0.2041 6,710,614.002585

35.048547 0.2041 6,810,891.423315

35.051947 0.204 6,899,697.448449

35.055348 0.2041 6,747,585.801428

35.058750 0.2041 6,633,676.609920

35.062150 0.204 6,519,976.123288

35.065552 0.2041 6,516,971.621060

35.068953 0.2041 6,729,967.873906

35.072353 0.204 6,733,957.751447

35.075755 0.2041 6,704,297.499591

35.079157 0.2041 6,760,949.937048

35.082557 0.204 6,685,694.719912

35.085958 0.2041 6,626,098.107464

35.089360 0.2041 6,697,724.062542

35.092760 0.204 6,726,342.209114

35.096162 0.2041 6,657,114.280058

35.099563 0.2041 6,731,879.321772

35.102963 0.204 6,704,499.576988

35.106365 0.2041 6,722,484.974229

35.109767 0.2041 6,841,509.661236

35.113167 0.204 6,806,076.937109

35.116568 0.2041 6,811,503.378981

35.119970 0.2041 6,747,862.844325

35.123370 0.204 6,674,973.301134

35.126772 0.2041 6,585,789.999572

35.130173 0.2041 6,376,505.295839

35.133573 0.204 6,314,241.098416

35.136975 0.2041 6,427,863.302150

35.140377 0.2041 6,573,664.610269

35.143777 0.204 6,553,301.810498

35.147178 0.2041 6,442,020.847558

35.150580 0.2041 6,534,006.031565

35.153980 0.204 6,514,599.892429

35.157382 0.2041 6,315,619.002366

35.160783 0.2041 6,395,077.031501

35.164183 0.204 6,492,471.066025

35.167585 0.2041 6,380,770.641928

35.170987 0.2041 6,446,428.054217

35.174387 0.204 6,626,933.147439

35.177788 0.2041 6,693,910.755773

35.181190 0.2041 6,528,223.597513

35.184590 0.204 6,395,666.053755

35.187992 0.2041 6,599,186.297330

35.191393 0.2041 6,801,741.680949

35.194793 0.204 6,841,037.946079

35.198195 0.2041 6,578,907.201423

35.201597 0.2041 6,565,075.992935

35.204997 0.204 6,846,014.125489

35.208398 0.2041 6,673,588.534671

35.211800 0.2041 6,561,746.966002

35.215200 0.204 6,643,701.452429

35.218602 0.2041 6,824,050.491288

35.222003 0.2041 7,182,464.775571

35.225405 0.2041 8,077,841.739209

35.228805 0.204 10,282,906.670777

35.232207 0.2041 14,435,878.158052

35.235608 0.2041 22,585,723.618938

35.239008 0.204 36,995,397.368469

35.242410 0.2041 61,170,521.556732

35.245812 0.2041 98,882,186.800801

35.249212 0.204 152,153,236.264783

35.252613 0.2041 221,870,839.001895

35.256015 0.2041 304,068,704.032778

35.259415 0.204 383,565,292.696275

35.262817 0.2041 456,565,373.673347

35.266218 0.2041 535,434,164.332446

35.269618 0.204 596,672,727.458643

35.273020 0.2041 612,188,510.026367

35.276422 0.2041 596,116,896.955777

35.279822 0.204 548,422,306.656654

35.283223 0.2041 458,861,338.050112

35.286625 0.2041 355,207,872.600233

35.290025 0.204 259,362,930.255791

35.293427 0.2041 173,855,275.040788

35.296828 0.2041 110,807,937.651639

35.300228 0.204 70,934,749.326986

35.303630 0.2041 45,663,694.753586

35.307032 0.2041 30,688,686.902398

35.310432 0.204 22,116,413.028490

35.313833 0.2041 17,420,638.410532

35.317235 0.2041 14,706,798.449209

35.320635 0.204 13,073,972.483052

35.324037 0.2041 12,074,502.056480

35.327438 0.2041 11,331,830.158080

35.330838 0.204 10,789,215.349783

35.334240 0.2041 10,225,041.989460

35.337642 0.2041 9,838,039.232412

35.341042 0.204 9,604,766.732763

35.344443 0.2041 9,156,370.650686

35.347845 0.2041 9,101,793.113694

35.351245 0.204 9,111,659.348527

35.354647 0.2041 8,731,895.673578

35.358048 0.2041 8,572,061.816597

35.361448 0.204 8,475,140.153008

35.364850 0.2041 8,165,807.533647

35.368252 0.2041 8,110,962.637412

35.371652 0.204 8,256,600.054732

35.375053 0.2041 8,188,943.581696

35.378455 0.2041 8,108,919.058101

35.381855 0.204 8,026,885.749768

35.385257 0.2041 7,786,098.932207

35.388658 0.2041 7,732,060.344940

35.392058 0.204 7,855,873.363262

35.395460 0.2041 7,814,910.772102

35.398862 0.2041 7,717,827.637704

35.402262 0.204 7,621,934.501095

35.405663 0.2041 7,626,609.496142

35.409065 0.2041 7,664,136.760951

35.412465 0.204 7,612,092.748546

35.415867 0.2041 7,611,253.049352

35.419268 0.2041 7,694,415.363129

35.422668 0.204 7,800,086.763418

35.426070 0.2041 7,944,885.674977

35.429472 0.2041 8,056,839.879309

35.432872 0.204 8,141,418.639595

35.436273 0.2041 8,155,710.967043

35.439675 0.2041 8,070,602.346009

35.443075 0.204 8,173,185.216993

35.446477 0.2041 8,220,609.291506

35.449878 0.2041 8,024,930.542430

35.453278 0.204 7,741,427.660379

35.456680 0.2041 7,764,714.102276

35.460082 0.2041 7,938,857.832901

35.463482 0.204 7,818,106.058531

35.466883 0.2041 7,665,475.615741

35.470285 0.2041 7,549,531.729325

35.473685 0.204 7,588,621.163454

35.477087 0.2041 7,707,466.932663

35.480488 0.2041 7,871,974.862877

35.483888 0.204 8,053,293.654577

35.487290 0.2041 8,086,791.445039

35.490692 0.2041 8,170,626.566899

35.494092 0.204 8,416,277.256863

35.497493 0.2041 8,569,904.876157

35.500895 0.2041 8,796,725.223635

35.504295 0.204 9,236,360.378985

35.507697 0.2041 9,334,581.971597

35.511098 0.2041 9,361,422.374192

35.514498 0.204 9,576,421.517439

35.517900 0.2041 9,687,619.206473

35.521302 0.2041 9,761,824.542502

35.524702 0.204 9,415,430.935602

35.528103 0.2041 9,163,409.722145

35.531505 0.2041 9,303,898.403018

35.534905 0.204 9,098,666.799143

35.538307 0.2041 9,052,288.182798

35.541708 0.2041 9,033,401.214948

35.545108 0.204 8,944,543.573658

35.548510 0.2041 9,326,459.445123

35.551912 0.2041 9,540,613.478663

35.555312 0.204 9,715,451.954737

35.558713 0.2041 10,336,883.683459

35.562115 0.2041 10,784,487.443486

35.565515 0.204 10,940,005.623049

35.568917 0.2041 10,822,921.446748

35.572318 0.2041 10,430,156.270568

35.575718 0.204 10,170,061.753920

35.579120 0.2041 9,872,959.050952

35.582522 0.2041 9,606,584.934891

35.585922 0.204 9,670,294.258313

35.589323 0.2041 9,836,934.957325

35.592725 0.2041 10,187,878.198476

35.596125 0.204 11,035,419.486324

35.599527 0.2041 12,301,374.514273

35.602928 0.2041 13,715,692.334068

35.606328 0.204 15,368,202.797032

35.609730 0.2041 17,021,801.772853

35.613132 0.2041 18,037,653.863421

35.616533 0.2041 18,592,729.044303

35.619933 0.204 18,737,534.991403

35.623335 0.2041 18,148,841.587682

35.626737 0.2041 17,028,516.518720

35.630137 0.204 15,404,272.357537

35.633538 0.2041 13,542,886.071553

35.636940 0.2041 11,892,193.960655

35.640340 0.204 10,578,573.033042

35.643742 0.2041 9,627,584.801623

35.647143 0.2041 8,920,576.794911

35.650543 0.204 8,424,237.554075

35.653945 0.2041 8,253,953.894801

35.657347 0.2041 8,207,597.624870

35.660747 0.204 8,140,453.686936

35.664148 0.2041 8,334,820.850339

35.667550 0.2041 8,468,424.678709

35.670950 0.204 8,551,926.639088

35.674352 0.2041 8,921,949.440876

35.677753 0.2041 9,358,844.736169

35.681153 0.204 9,842,630.821110

35.684555 0.2041 10,259,978.225067

35.687957 0.2041 10,437,269.852534

35.691357 0.204 10,435,438.732603

35.694758 0.2041 10,340,434.938054

35.698160 0.2041 10,167,555.034478

35.701560 0.204 9,768,195.350996

35.704962 0.2041 9,336,982.832690

35.708363 0.2041 9,027,272.286272

35.711763 0.204 8,580,487.696653

35.715165 0.2041 8,091,143.526642

35.718567 0.2041 7,889,430.102603

35.721967 0.204 7,823,274.621504

35.725368 0.2041 7,538,186.225084

35.728770 0.2041 7,430,266.074319

35.732170 0.204 7,456,736.431266

35.735572 0.2041 7,313,556.187484

35.738973 0.2041 7,357,591.876022

35.742373 0.204 7,411,976.076009

35.745775 0.2041 7,370,030.457734

35.749177 0.2041 7,307,458.573208

35.752577 0.204 7,303,176.602267

35.755978 0.2041 7,424,675.605968

35.759380 0.2041 7,348,469.494055

35.762780 0.204 7,253,697.070520

35.766182 0.2041 7,341,371.107898

35.769583 0.2041 7,308,983.154571

35.772983 0.204 7,373,432.793502

35.776385 0.2041 7,444,483.622041

35.779787 0.2041 7,312,527.550352

35.783187 0.204 7,324,051.084925

35.786588 0.2041 7,342,618.307173

35.789990 0.2041 7,501,198.407507

35.793390 0.204 7,840,066.518167

35.796792 0.2041 7,864,658.216064

35.800193 0.2041 7,713,365.556085

35.803593 0.204 7,681,562.146554

35.806995 0.2041 7,660,963.840706

35.810397 0.2041 7,622,955.605767

35.813797 0.204 7,498,506.556542

35.817198 0.2041 7,364,696.730501

35.820600 0.2041 7,370,741.311809

35.824000 0.204 7,328,657.687717

35.827402 0.2041 7,265,711.929005

35.830803 0.2041 7,334,680.237863

35.834203 0.204 7,467,728.040418

35.837605 0.2041 7,505,839.272992

35.841007 0.2041 7,673,494.405193

35.844407 0.204 7,849,521.116298

35.847808 0.2041 8,001,643.031223

35.851210 0.2041 8,295,545.742713

35.854610 0.204 8,368,346.615565

35.858012 0.2041 8,510,577.844914

35.861413 0.2041 8,672,953.271639

35.864813 0.204 8,696,031.736515

35.868215 0.2041 8,676,455.439076

35.871617 0.2041 8,427,053.941261

35.875017 0.204 8,133,202.474296

35.878418 0.2041 7,825,730.205640

35.881820 0.2041 7,740,366.902631

35.885220 0.204 7,954,899.172143

35.888622 0.2041 8,151,083.984856

35.892023 0.2041 8,271,181.047544

35.895423 0.204 8,288,898.570031

35.898825 0.2041 8,339,282.925770

35.902227 0.2041 8,388,369.943000

35.905627 0.204 8,294,326.011493

35.909028 0.2041 8,265,104.976418

35.912430 0.2041 8,267,866.530597

35.915830 0.204 8,073,817.794835

35.919232 0.2041 7,812,929.119228

35.922633 0.2041 7,498,365.318940

35.926033 0.204 7,368,733.260092

35.929435 0.2041 7,428,247.082642

35.932837 0.2041 7,225,956.451401

35.936237 0.204 7,107,417.274600

35.939638 0.2041 7,131,744.561371

35.943040 0.2041 7,125,759.527110

35.946440 0.204 7,134,677.301567

35.949842 0.2041 7,069,018.997350

35.953243 0.2041 6,962,058.175396

35.956643 0.204 6,859,162.919284

35.960045 0.2041 6,972,969.759139

35.963447 0.2041 7,172,868.764583

35.966847 0.204 7,216,436.529421

35.970248 0.2041 7,252,343.528648

35.973650 0.2041 7,207,339.892707

35.977052 0.2041 7,176,528.162829

35.980452 0.204 7,281,650.643205

35.983853 0.2041 7,225,089.672015

35.987255 0.2041 7,150,226.481722

35.990655 0.204 7,238,683.494824

35.994057 0.2041 7,246,321.925208

35.997458 0.2041 7,184,031.201470

36.000858 0.204 7,057,800.453045

36.004260 0.2041 7,000,664.872028

36.007662 0.2041 7,070,081.066033

36.011062 0.204 7,071,378.337637

36.014463 0.2041 7,244,434.017010

36.017865 0.2041 7,289,401.459020

36.021265 0.204 7,095,634.813534

36.024667 0.2041 7,115,395.575392

36.028068 0.2041 7,107,984.030961

36.031468 0.204 7,158,324.158510

36.034870 0.2041 7,208,353.987864

36.038272 0.2041 7,262,572.659556

36.041672 0.204 7,483,585.692034

36.045073 0.2041 7,526,190.297955

36.048475 0.2041 7,546,425.511973

36.051875 0.204 7,790,119.758474

36.055277 0.2041 7,980,868.904309

36.058678 0.2041 8,064,121.398449

36.062078 0.204 8,245,646.956405

36.065480 0.2041 8,233,618.428252

36.068882 0.2041 7,976,413.225378

36.072282 0.204 7,997,256.507125

36.075683 0.2041 8,088,078.322769

36.079085 0.2041 8,113,072.052503

36.082485 0.204 8,019,633.715728

36.085887 0.2041 7,791,586.604588

36.089288 0.2041 7,880,193.339856

36.092688 0.204 7,940,622.866139

36.096090 0.2041 7,939,556.307252

36.099492 0.2041 8,185,518.239508

36.102892 0.204 8,681,912.987973

36.106293 0.2041 9,197,194.179119

36.109695 0.2041 9,692,788.790567

36.113095 0.204 10,108,100.990452

36.116497 0.2041 10,276,351.965356

36.119898 0.2041 10,515,786.637415

36.123298 0.204 10,466,364.222585

36.126700 0.2041 10,018,458.332698

36.130102 0.2041 9,580,240.311148

36.133502 0.204 9,190,197.168836

36.136903 0.2041 8,634,462.394287

36.140305 0.2041 8,086,236.586618

36.143705 0.204 7,821,669.963972

36.147107 0.2041 7,705,098.023106

36.150508 0.2041 7,545,214.693957

36.153908 0.204 7,280,882.376483

36.157310 0.2041 7,282,805.354787

36.160712 0.2041 7,410,263.265757

36.164112 0.204 7,436,090.303721

36.167513 0.2041 7,398,847.394997

36.170915 0.2041 7,357,815.122329

36.174315 0.204 7,370,528.736853

36.177717 0.2041 7,531,210.425380

36.181118 0.2041 7,704,235.152948

36.184518 0.204 7,553,717.729803

36.187920 0.2041 7,468,066.536608

36.191322 0.2041 7,529,271.748779

36.194722 0.204 7,423,749.224763

36.198123 0.2041 7,349,336.655166

36.201525 0.2041 7,367,328.502635

36.204925 0.204 7,300,558.367829

36.208327 0.2041 7,330,290.102122

36.211728 0.2041 7,395,291.795212

36.215128 0.204 7,155,930.312260

36.218530 0.2041 7,032,694.549270

36.221932 0.2041 7,269,064.146610

36.225332 0.204 7,200,949.078436

36.228733 0.2041 7,037,874.921519

36.232135 0.2041 7,047,413.784278

36.235535 0.204 7,097,142.208034

36.238937 0.2041 7,253,843.584431

36.242338 0.2041 7,314,866.149460

36.245738 0.204 7,096,891.354747

36.249140 0.2041 6,986,681.712855

36.252542 0.2041 7,235,663.670734

36.255942 0.204 7,245,672.323721

36.259343 0.2041 7,092,695.041882

36.262745 0.2041 7,147,890.691169

36.266145 0.204 7,287,090.058446

36.269547 0.2041 7,116,695.986766

36.272948 0.2041 6,975,899.770167

36.276348 0.204 7,161,583.665910

36.279750 0.2041 7,125,124.700517

36.283152 0.2041 7,152,193.245591

36.286552 0.204 7,272,809.754003

36.289953 0.2041 7,153,559.098332

36.293355 0.2041 7,091,949.593731

36.296755 0.204 7,138,679.242734

36.300157 0.2041 7,261,099.588362

36.303558 0.2041 7,302,105.885813

36.306958 0.204 7,209,729.227360

36.310360 0.2041 7,257,685.115055

36.313762 0.2041 7,229,029.245029

36.317162 0.204 7,023,998.654010

36.320563 0.2041 7,064,035.945796

36.323965 0.2041 7,162,574.211909

36.327367 0.2041 7,083,847.259552

36.330767 0.204 7,141,285.016570

36.334168 0.2041 7,140,491.144778

36.337570 0.2041 7,118,112.637117

36.340970 0.204 7,333,578.888030

36.344372 0.2041 7,398,007.495089

36.347773 0.2041 7,282,959.065940

36.351173 0.204 7,321,694.781982

36.354575 0.2041 7,358,509.121683

36.357977 0.2041 7,300,684.560249

36.361377 0.204 7,361,459.396490

36.364778 0.2041 7,364,673.647591

36.368180 0.2041 7,433,599.978259

36.371580 0.204 7,410,683.348560

36.374982 0.2041 7,270,863.471112

36.378383 0.2041 7,465,399.835701

36.381783 0.204 7,638,372.256307

36.385185 0.2041 7,622,509.403600

36.388587 0.2041 7,549,257.983638

36.391987 0.204 7,589,480.842348

36.395388 0.2041 7,596,123.495913

36.398790 0.2041 7,687,279.711859

36.402190 0.204 7,818,559.342007

36.405592 0.2041 7,673,731.214053

36.408993 0.2041 7,582,752.699925

36.412393 0.204 7,484,720.642509

36.415795 0.2041 7,427,017.189170

36.419197 0.2041 7,527,670.988326

36.422597 0.204 7,545,186.181960

36.425998 0.2041 7,462,376.195155

36.429400 0.2041 7,375,979.162181

36.432800 0.204 7,285,301.563016

36.436202 0.2041 7,258,220.007410

36.439603 0.2041 7,225,848.649989

36.443003 0.204 7,254,526.968033

36.446405 0.2041 7,273,676.121325

36.449807 0.2041 7,210,155.655246

36.453207 0.204 7,267,244.783240

36.456608 0.2041 7,250,786.537129

36.460010 0.2041 7,337,520.181563

36.463410 0.204 7,294,595.463692

36.466812 0.2041 7,139,979.537578

36.470213 0.2041 7,357,134.347850

36.473613 0.204 7,362,714.285692

36.477015 0.2041 7,275,821.576237

36.480417 0.2041 7,257,966.392078

36.483817 0.204 7,154,267.528205

36.487218 0.2041 7,187,836.162026

36.490620 0.2041 7,178,298.664571

36.494020 0.204 7,222,529.743903

36.497422 0.2041 7,332,154.143030

36.500823 0.2041 7,202,325.105035

36.504223 0.204 7,138,762.045729

36.507625 0.2041 7,228,551.045973

36.511027 0.2041 7,108,807.636348

36.514427 0.204 7,081,656.616658

36.517828 0.2041 7,228,380.968439

36.521230 0.2041 7,251,453.607244

36.524630 0.204 7,152,965.655026

36.528032 0.2041 7,111,238.965944

36.531433 0.2041 7,117,949.133588

36.534833 0.204 7,105,275.483504

36.538235 0.2041 7,115,873.123238

36.541637 0.2041 7,053,923.592504

36.545037 0.204 7,093,863.739324

36.548438 0.2041 7,272,161.098181

36.551840 0.2041 7,283,533.273550

36.555240 0.204 7,151,476.486246

36.558642 0.2041 7,125,075.177988

36.562043 0.2041 7,185,968.490317

36.565443 0.204 7,139,304.012775

36.568845 0.2041 7,178,234.645861

36.572247 0.2041 7,310,141.012685

36.575647 0.204 7,338,529.832364

36.579048 0.2041 7,534,796.197349

36.582450 0.2041 7,887,972.998724

36.585850 0.204 8,068,868.866475

36.589252 0.2041 8,227,866.585042

36.592653 0.2041 8,466,200.472548

36.596053 0.204 8,570,634.444079

36.599455 0.2041 8,752,866.074224

36.602857 0.2041 8,867,651.589903

36.606257 0.204 8,887,345.404646

36.609658 0.2041 8,998,980.057635

36.613060 0.2041 8,949,876.085900

36.616460 0.204 8,764,163.717155

36.619862 0.2041 8,594,594.587272

36.623263 0.2041 8,315,333.434834

36.626663 0.204 8,003,932.793635

36.630065 0.2041 7,896,086.162046

36.633467 0.2041 7,860,356.007685

36.636867 0.204 7,786,688.239667

36.640268 0.2041 7,643,994.601160

36.643670 0.2041 7,507,672.743365

36.647070 0.204 7,382,896.572206

36.650472 0.2041 7,361,526.348676

36.653873 0.2041 7,327,546.158447

36.657273 0.204 7,171,835.391717

36.660675 0.2041 7,323,714.364735

36.664077 0.2041 7,509,948.946239

36.667477 0.204 7,545,796.705119

36.670878 0.2041 7,564,962.158807

36.674280 0.2041 7,410,374.231228

36.677682 0.2041 7,342,977.716999

36.681082 0.204 7,392,458.215471

36.684483 0.2041 7,450,606.166465

36.687885 0.2041 7,488,751.078462

36.691285 0.204 7,353,545.608998

36.694687 0.2041 7,360,428.258617

36.698088 0.2041 7,373,003.148561

36.701488 0.204 7,198,731.338457

36.704890 0.2041 7,307,605.294522

36.708292 0.2041 7,377,089.068911

36.711692 0.204 7,264,839.794816

36.715093 0.2041 7,210,005.921031

36.718495 0.2041 7,217,209.051111

36.721895 0.204 7,271,598.674704

36.725297 0.2041 7,274,019.227889

36.728698 0.2041 7,345,416.791210

36.732098 0.204 7,521,258.229600

36.735500 0.2041 7,381,226.296324

36.738902 0.2041 7,227,561.606809

36.742302 0.204 7,289,202.756152

36.745703 0.2041 7,247,450.148324

36.749105 0.2041 7,317,673.891314

36.752505 0.204 7,355,985.576411

36.755907 0.2041 7,350,877.755568

36.759308 0.2041 7,373,190.370287

36.762708 0.204 7,427,270.116415

36.766110 0.2041 7,465,158.769913

36.769512 0.2041 7,411,752.543603

36.772912 0.204 7,349,045.543387

36.776313 0.2041 7,253,347.540672

36.779715 0.2041 7,326,537.537499

36.783115 0.204 7,428,263.912831

36.786517 0.2041 7,406,773.884761

36.789918 0.2041 7,330,955.863088

36.793318 0.204 7,161,494.622421

36.796720 0.2041 7,193,270.445753

36.800122 0.2041 7,354,494.521767

36.803522 0.204 7,370,527.060416

36.806923 0.2041 7,476,428.880136

36.810325 0.2041 7,619,714.653541

36.813725 0.204 7,531,056.091035

36.817127 0.2041 7,247,996.684210

36.820528 0.2041 7,342,358.544111

36.823928 0.204 7,636,870.717062

36.827330 0.2041 7,546,107.956531

36.830732 0.2041 7,565,877.981741

36.834132 0.204 7,493,851.639154

36.837533 0.2041 7,288,822.079989

36.840935 0.2041 7,369,018.915776

36.844335 0.204 7,484,720.108492

36.847737 0.2041 7,467,424.046763

36.851138 0.2041 7,435,450.596930

36.854538 0.204 7,571,079.068390

36.857940 0.2041 7,613,176.794735

36.861342 0.2041 7,640,608.175433

36.864742 0.204 8,052,478.538822

36.868143 0.2041 8,380,263.730335

36.871545 0.2041 8,496,783.319912

36.874945 0.204 8,815,193.621751

36.878347 0.2041 9,214,294.434140

36.881748 0.2041 9,596,268.936429

36.885148 0.204 9,818,600.855622

36.888550 0.2041 9,737,491.818238

36.891952 0.2041 9,708,847.436323

36.895352 0.204 9,755,639.139603

36.898753 0.2041 9,449,314.567413

36.902155 0.2041 9,085,551.697413

36.905555 0.204 8,896,637.067047

36.908957 0.2041 8,600,286.876205

36.912358 0.2041 8,260,477.570197

36.915758 0.204 8,081,928.311556

36.919160 0.2041 8,022,138.643926

36.922562 0.2041 8,118,512.425373

36.925962 0.204 8,124,789.035652

36.929363 0.2041 7,931,833.965013

36.932765 0.2041 7,877,051.847460

36.936165 0.204 7,881,516.870807

36.939567 0.2041 7,716,696.604279

36.942968 0.2041 7,653,728.837693

36.946368 0.204 7,755,069.761358

36.949770 0.2041 7,690,131.076276

36.953172 0.2041 7,635,223.987008

36.956572 0.204 7,563,624.652929

36.959973 0.2041 7,511,143.617598

36.963375 0.2041 7,564,961.964512

36.966775 0.204 7,705,395.177195

36.970177 0.2041 7,671,836.474367

36.973578 0.2041 7,391,522.182661

36.976978 0.204 7,482,519.631593

36.980380 0.2041 7,595,847.996978

36.983782 0.2041 7,545,792.012415

36.987182 0.204 7,708,934.104766

36.990583 0.2041 7,781,823.023978

36.993985 0.2041 7,812,029.922027

36.997385 0.204 7,691,820.226268

37.000787 0.2041 7,575,798.493287

37.004188 0.2041 7,707,900.980049

37.007588 0.204 7,723,468.221351

37.010990 0.2041 7,604,199.340050

37.014392 0.2041 7,531,557.041031

37.017793 0.2041 7,626,620.755108

37.021193 0.204 7,689,621.890442

37.024595 0.2041 7,666,248.641966

37.027997 0.2041 7,605,251.340447

37.031397 0.204 7,624,095.221874

37.034798 0.2041 7,602,609.469413

37.038200 0.2041 7,444,876.687851

37.041600 0.204 7,535,417.691102

37.045002 0.2041 7,677,968.720587

37.048403 0.2041 7,530,491.727037

37.051803 0.204 7,337,778.575499

37.055205 0.2041 7,443,789.625389

37.058607 0.2041 7,545,277.407297

37.062007 0.204 7,488,186.903967

37.065408 0.2041 7,538,189.380294

37.068810 0.2041 7,442,339.327728

37.072210 0.204 7,455,100.938951

37.075612 0.2041 7,570,785.945795

37.079013 0.2041 7,395,335.578614

37.082413 0.204 7,494,165.265743

37.085815 0.2041 7,690,514.937827

37.089217 0.2041 7,660,568.566277

37.092617 0.204 7,708,084.564837

37.096018 0.2041 7,657,634.018170

37.099420 0.2041 7,642,697.144429

37.102820 0.204 7,647,909.615093

37.106222 0.2041 7,505,389.051386

37.109623 0.2041 7,549,970.995893

37.113023 0.204 7,492,799.337639

37.116425 0.2041 7,527,497.812474

37.119827 0.2041 7,586,241.657976

37.123227 0.204 7,513,383.285942

37.126628 0.2041 7,650,359.204860

37.130030 0.2041 7,606,934.796685

37.133430 0.204 7,523,687.921046

37.136832 0.2041 7,409,109.410045

37.140233 0.2041 7,375,371.748849

37.143633 0.204 7,515,201.520988

37.147035 0.2041 7,493,403.817129

37.150437 0.2041 7,414,593.273133

37.153837 0.204 7,477,995.446723

37.157238 0.2041 7,579,324.195675

37.160640 0.2041 7,577,571.995774

37.164040 0.204 7,520,325.666973

37.167442 0.2041 7,465,186.397383

37.170843 0.2041 7,486,275.790125

37.174243 0.204 7,476,230.210800

37.177645 0.2041 7,440,210.724071

37.181047 0.2041 7,517,675.333874

37.184447 0.204 7,630,834.082376

37.187848 0.2041 7,637,757.986076

37.191250 0.2041 7,542,292.374993

37.194650 0.204 7,579,659.915465

37.198052 0.2041 7,657,287.114297

37.201453 0.2041 7,608,520.871790

37.204853 0.204 7,594,383.139941

37.208255 0.2041 7,514,090.536797

37.211657 0.2041 7,504,814.553420

37.215057 0.204 7,645,457.050624

37.218458 0.2041 7,688,603.523350

37.221860 0.2041 7,507,528.722440

37.225260 0.204 7,495,132.735315

37.228662 0.2041 7,602,453.707759

37.232063 0.2041 7,506,821.873433

37.235463 0.204 7,561,289.567437

37.238865 0.2041 7,601,360.484260

37.242267 0.2041 7,460,790.254518

37.245667 0.204 7,315,190.824304

37.249068 0.2041 7,416,183.126177

37.252470 0.2041 7,648,352.689535

37.255870 0.204 7,605,111.895300

37.259272 0.2041 7,475,512.678295

37.262673 0.2041 7,376,218.581386

37.266073 0.204 7,441,205.816992

37.269475 0.2041 7,703,587.213712

37.272877 0.2041 7,621,423.631499

37.276277 0.204 7,493,595.177979

37.279678 0.2041 7,435,523.112472

37.283080 0.2041 7,471,855.312741

37.286480 0.204 7,581,000.607128

37.289882 0.2041 7,332,557.189688

37.293283 0.2041 7,371,937.803907

37.296683 0.204 7,522,320.881308

37.300085 0.2041 7,579,052.064959

37.303487 0.2041 7,612,895.397417

37.306887 0.204 7,506,136.241841

37.310288 0.2041 7,431,439.784677

37.313690 0.2041 7,410,193.487574

37.317090 0.204 7,516,026.677765

37.320492 0.2041 7,477,717.453768

37.323893 0.2041 7,405,880.549639

37.327293 0.204 7,428,435.570534

37.330695 0.2041 7,465,949.085826

37.334097 0.2041 7,560,522.687771

37.337497 0.204 7,669,433.348393

37.340898 0.2041 7,595,981.763057

37.344300 0.2041 7,489,424.980630

37.347700 0.204 7,591,456.360667

37.351102 0.2041 7,473,670.836147

37.354503 0.2041 7,521,742.808783

37.357903 0.204 7,739,422.518273

37.361305 0.2041 7,633,779.211033

37.364707 0.2041 7,645,121.151409

37.368107 0.204 7,667,569.428246

37.371508 0.2041 7,725,477.590712

37.374910 0.2041 7,642,650.364402

37.378312 0.2041 7,500,315.472796

37.381712 0.204 7,651,076.549570

37.385113 0.2041 7,583,295.034528

37.388515 0.2041 7,453,232.923992

37.391915 0.204 7,494,221.087236

37.395317 0.2041 7,454,987.518703

37.398718 0.2041 7,423,011.380718

37.402118 0.204 7,596,702.939791

37.405520 0.2041 7,590,810.698173

37.408922 0.2041 7,475,317.282098

37.412322 0.204 7,582,786.708423

37.415723 0.2041 7,621,362.724971

37.419125 0.2041 7,637,340.666710

37.422525 0.204 7,695,099.866159

37.425927 0.2041 7,614,154.616220

37.429328 0.2041 7,645,831.353827

37.432728 0.204 7,727,706.610664

37.436130 0.2041 7,652,901.760113

37.439532 0.2041 7,664,716.276401

37.442932 0.204 7,601,991.685415

37.446333 0.2041 7,660,040.060832

37.449735 0.2041 7,823,709.691059

37.453135 0.204 7,629,724.311433

37.456537 0.2041 7,562,037.010753

37.459938 0.2041 7,693,154.882598

37.463338 0.204 7,667,047.544784

37.466740 0.2041 7,698,933.093159

37.470142 0.2041 7,658,046.061053

37.473542 0.204 7,499,144.755251

37.476943 0.2041 7,587,623.665326

37.480345 0.2041 7,671,320.642771

37.483745 0.204 7,698,522.678343

37.487147 0.2041 7,707,395.324844

37.490548 0.2041 7,687,826.173546

37.493948 0.204 7,816,322.932195

37.497350 0.2041 7,842,150.389082

37.500752 0.2041 7,780,489.766934

37.504152 0.204 7,723,987.122003

37.507553 0.2041 7,758,070.495374

37.510955 0.2041 7,840,507.465760

37.514355 0.204 7,809,089.549424

37.517757 0.2041 7,831,468.635643

37.521158 0.2041 7,804,106.421720

37.524558 0.204 7,817,698.734012

37.527960 0.2041 7,833,409.576442

37.531362 0.2041 7,688,912.346416

37.534762 0.204 7,654,267.116414

37.538163 0.2041 7,633,269.354727

37.541565 0.2041 7,576,650.539097

37.544965 0.204 7,600,263.551421

37.548367 0.2041 7,557,016.987613

37.551768 0.2041 7,457,992.099104

37.555168 0.204 7,445,916.100573

37.558570 0.2041 7,412,732.481854

37.561972 0.2041 7,506,062.127329

37.565372 0.204 7,740,858.297789

37.568773 0.2041 7,773,234.369535

37.572175 0.2041 7,754,083.099652

37.575575 0.204 7,787,207.334763

37.578977 0.2041 7,889,907.585719

37.582378 0.2041 8,023,012.953739

37.585778 0.204 8,122,061.746756

37.589180 0.2041 8,306,257.986451

37.592582 0.2041 8,221,255.256190

37.595982 0.204 8,070,870.271967

37.599383 0.2041 8,188,817.418106

37.602785 0.2041 8,036,480.797612

37.606185 0.204 7,827,533.442983

37.609587 0.2041 8,011,332.652761

37.612988 0.2041 8,061,439.173223

37.616388 0.204 7,720,198.062675

37.619790 0.2041 7,575,886.783070

37.623192 0.2041 7,671,851.281430

37.626592 0.204 7,739,855.973228

37.629993 0.2041 7,766,854.357787

37.633395 0.2041 7,705,870.157734

37.636795 0.204 7,600,746.676328

37.640197 0.2041 7,680,013.266360

37.643598 0.2041 7,882,605.165145

37.646998 0.204 7,863,401.695162

37.650400 0.2041 7,823,448.699116

37.653802 0.2041 7,896,088.570844

37.657202 0.204 7,973,244.392492

37.660603 0.2041 7,961,460.693463

37.664005 0.2041 7,995,149.744566

37.667405 0.204 8,121,915.703942

37.670807 0.2041 8,221,421.886413

37.674208 0.2041 8,455,282.081933

37.677608 0.204 8,548,975.876548

37.681010 0.2041 8,555,247.238186

37.684412 0.2041 8,369,558.440321

37.687812 0.204 8,129,689.559896

37.691213 0.2041 8,159,824.529837

37.694615 0.2041 8,104,938.903556

37.698015 0.204 8,221,834.962054

37.701417 0.2041 8,158,467.458567

37.704818 0.2041 7,992,174.658639

37.708218 0.204 8,028,622.234339

37.711620 0.2041 7,799,656.628169

37.715022 0.2041 7,827,184.389534

37.718423 0.2041 7,982,157.425352

37.721823 0.204 7,875,869.888022

37.725225 0.2041 7,838,821.677445

37.728627 0.2041 7,861,537.834246

37.732027 0.204 7,834,374.064371

37.735428 0.2041 7,862,853.169904

37.738830 0.2041 7,947,024.179359

37.742230 0.204 7,892,983.332212

37.745632 0.2041 7,841,689.800862

37.749033 0.2041 7,639,811.800748

37.752433 0.204 7,519,753.259095

37.755835 0.2041 7,707,473.710847

37.759237 0.2041 7,722,907.871665

37.762637 0.204 7,781,554.424931

37.766038 0.2041 7,848,020.187293

37.769440 0.2041 7,831,168.067204

37.772840 0.204 7,857,553.391929

37.776242 0.2041 7,795,146.409464

37.779643 0.2041 7,802,798.562543

37.783043 0.204 7,853,301.588125

37.786445 0.2041 7,787,915.089757

37.789847 0.2041 7,809,385.681841

37.793247 0.204 7,678,509.772730

37.796648 0.2041 7,544,503.038146

37.800050 0.2041 7,678,861.257906

37.803450 0.204 7,564,275.533165

37.806852 0.2041 7,485,670.215897

37.810253 0.2041 7,673,386.573454

37.813653 0.204 7,746,124.322881

37.817055 0.2041 7,674,230.217331

37.820457 0.2041 7,726,566.307073

37.823857 0.204 7,720,018.400565

37.827258 0.2041 7,768,029.702714

37.830660 0.2041 7,920,125.015303

37.834060 0.204 7,839,013.478997

37.837462 0.2041 7,896,746.325698

37.840863 0.2041 7,901,027.772820

37.844263 0.204 7,867,393.917993

37.847665 0.2041 8,029,772.807382

37.851067 0.2041 8,002,399.372910

37.854467 0.204 7,931,037.815964

37.857868 0.2041 7,850,473.356591

37.861270 0.2041 7,818,747.073905

37.864670 0.204 7,944,434.423938

37.868072 0.2041 7,906,149.090535

37.871473 0.2041 7,864,917.369785

37.874873 0.204 7,908,786.340389

37.878275 0.2041 7,841,650.069221

37.881677 0.2041 7,816,595.710750

37.885077 0.204 7,845,542.861378

37.888478 0.2041 7,812,024.837433

37.891880 0.2041 7,708,107.871177

37.895280 0.204 7,676,454.069937

37.898682 0.2041 7,798,141.123560

37.902083 0.2041 7,886,765.293882

37.905483 0.204 7,832,831.741835

37.908885 0.2041 7,695,563.393179

37.912287 0.2041 7,886,979.606672

37.915687 0.204 8,246,975.859469

37.919088 0.2041 8,367,595.730714

37.922490 0.2041 8,615,772.017794

37.925890 0.204 9,227,537.343208

37.929292 0.2041 10,049,606.944638

37.932693 0.2041 10,899,265.609018

37.936093 0.204 11,601,527.341694

37.939495 0.2041 12,197,579.959025

37.942897 0.2041 12,840,041.441451

37.946297 0.204 13,386,031.173594

37.949698 0.2041 13,631,898.613120

37.953100 0.2041 13,315,973.633039

37.956500 0.204 12,787,059.901165

37.959902 0.2041 12,353,991.762342

37.963303 0.2041 11,705,713.147726

37.966703 0.204 10,768,952.551146

37.970105 0.2041 9,932,181.050253

37.973507 0.2041 9,556,616.702964

37.976907 0.204 9,050,159.990421

37.980308 0.2041 8,595,478.986446

37.983710 0.2041 8,432,547.019016

37.987110 0.204 8,184,607.160666

37.990512 0.2041 8,061,367.318676

37.993913 0.2041 8,076,013.384269

37.997313 0.204 8,045,069.070146

38.000715 0.2041 7,923,044.520422

38.004117 0.2041 7,894,255.484440

38.007517 0.204 7,892,006.907551

38.010918 0.2041 7,934,484.712860

38.014320 0.2041 7,925,486.365276

38.017720 0.204 7,717,668.398147

38.021122 0.2041 7,707,652.131020

38.024523 0.2041 7,796,035.937332

38.027923 0.204 7,854,909.752664

38.031325 0.2041 7,874,187.399898

38.034727 0.2041 7,769,685.422257

38.038127 0.204 7,888,061.050240

38.041528 0.2041 7,982,410.445064

38.044930 0.2041 7,939,072.968464

38.048330 0.204 7,966,551.903339

38.051732 0.2041 8,013,252.960971

38.055133 0.2041 8,069,617.828072

38.058533 0.204 8,008,549.124060

38.061935 0.2041 8,014,099.804233

38.065337 0.2041 7,920,748.496477

38.068738 0.2041 7,778,742.515029

38.072138 0.204 7,881,155.715093

38.075540 0.2041 8,034,625.269543

38.078942 0.2041 7,975,439.947280

38.082342 0.204 7,887,008.041973

38.085743 0.2041 7,942,359.530395

38.089145 0.2041 7,860,727.850588

38.092545 0.204 7,801,174.832067

38.095947 0.2041 7,753,143.177482

38.099348 0.2041 7,775,974.998681

38.102748 0.204 7,999,715.778686

38.106150 0.2041 8,026,315.828958

38.109552 0.2041 7,938,722.271377

38.112952 0.204 7,994,632.204240

38.116353 0.2041 8,078,550.350629

38.119755 0.2041 8,060,608.434371

38.123155 0.204 8,000,577.583910

38.126557 0.2041 7,966,190.414564

38.129958 0.2041 7,922,739.114195

38.133358 0.204 7,911,797.659696

38.136760 0.2041 7,902,395.113892

38.140162 0.2041 7,843,041.181682

38.143562 0.204 7,818,243.506548

38.146963 0.2041 7,839,000.235295

38.150365 0.2041 7,813,988.184923

38.153765 0.204 7,830,080.377400

38.157167 0.2041 7,882,407.930130

38.160568 0.2041 7,965,819.836353

38.163968 0.204 8,090,124.321398

38.167370 0.2041 8,122,447.597099

38.170772 0.2041 8,183,882.697389

38.174172 0.204 8,198,417.529346

38.177573 0.2041 8,381,113.438163

38.180975 0.2041 8,909,493.526415

38.184375 0.204 9,398,840.759717

38.187777 0.2041 10,168,992.184954

38.191178 0.2041 11,617,959.114863

38.194578 0.204 13,973,354.591092

38.197980 0.2041 17,401,448.566575

38.201382 0.2041 22,290,609.380492

38.204782 0.204 29,343,447.894327

38.208183 0.2041 39,242,514.600549

38.211585 0.2041 51,853,337.821877

38.214985 0.204 66,097,942.988826

38.218387 0.2041 81,706,734.264613

38.221788 0.2041 97,268,536.137907

38.225188 0.204 110,864,491.734270

38.228590 0.2041 120,568,811.512626

38.231992 0.2041 125,148,679.107661

38.235392 0.204 125,292,582.251021

38.238793 0.2041 120,653,606.782415

38.242195 0.2041 110,887,160.092968

38.245595 0.204 98,169,315.983188

38.248997 0.2041 84,722,668.905323

38.252398 0.2041 70,638,751.894431

38.255798 0.204 58,062,410.755295

38.259200 0.2041 47,560,352.570986

38.262602 0.2041 38,644,151.625753

38.266002 0.204 31,933,275.830164

38.269403 0.2041 26,694,347.111578

38.272805 0.2041 23,061,157.818074

38.276205 0.204 20,394,564.556221

38.279607 0.2041 18,158,764.173769

38.283008 0.2041 16,325,812.097679

38.286408 0.204 15,120,087.424738

38.289810 0.2041 14,335,878.319114

38.293212 0.2041 13,450,658.284524

38.296612 0.204 12,915,769.391540

38.300013 0.2041 12,560,757.331458

38.303415 0.2041 12,011,638.357611

38.306815 0.204 11,822,489.428206

38.310217 0.2041 11,628,990.105390

38.313618 0.2041 11,287,181.953465

38.317018 0.204 11,481,915.069640

38.320420 0.2041 11,764,424.587715

38.323822 0.2041 11,951,447.066079

38.327222 0.204 12,438,436.673715

38.330623 0.2041 13,104,532.343313

38.334025 0.2041 13,799,065.772789

38.337425 0.204 14,604,370.517470

38.340827 0.2041 15,091,248.871739

38.344228 0.2041 15,411,932.550763

38.347628 0.204 15,629,023.005412

38.351030 0.2041 15,584,530.428298

38.354432 0.2041 15,437,177.406990

38.357832 0.204 14,837,563.708050

38.361233 0.2041 14,036,765.813559

38.364635 0.2041 13,334,081.454578

38.368035 0.204 12,723,233.435243

38.371437 0.2041 12,141,683.851698

38.374838 0.2041 11,391,208.628132

38.378238 0.204 10,709,526.450181

38.381640 0.2041 10,127,236.719237

38.385042 0.2041 9,706,272.493011

38.388442 0.204 9,549,988.350523

38.391843 0.2041 9,442,055.144167

38.395245 0.2041 9,312,674.062877

38.398645 0.204 9,135,553.707219

38.402047 0.2041 8,925,347.832999

38.405448 0.2041 9,023,814.901933

38.408850 0.2041 9,089,443.356957

38.412250 0.204 8,802,100.049970

38.415652 0.2041 8,727,962.825069

38.419053 0.2041 8,702,001.875372

38.422453 0.204 8,552,316.002646

38.425855 0.2041 8,612,778.471740

38.429257 0.2041 8,788,350.592220

38.432657 0.204 8,819,794.065398

38.436058 0.2041 8,753,848.542295

38.439460 0.2041 8,713,365.402517

38.442860 0.204 8,813,452.265882

38.446262 0.2041 8,816,610.501984

38.449663 0.2041 8,608,176.021188

38.453063 0.204 8,635,701.655823

38.456465 0.2041 8,671,716.901836

38.459867 0.2041 8,566,032.676799

38.463267 0.204 8,754,642.008475

38.466668 0.2041 8,789,571.770106

38.470070 0.2041 8,677,659.249593

38.473470 0.204 8,867,513.378608

38.476872 0.2041 9,007,582.321527

38.480273 0.2041 9,137,283.477526

38.483673 0.204 9,245,327.294899

38.487075 0.2041 9,298,377.230256

38.490477 0.2041 9,623,886.352690

38.493877 0.204 10,012,147.446740

38.497278 0.2041 10,123,870.874198

38.500680 0.2041 10,162,851.626952

38.504080 0.204 10,312,853.031881

38.507482 0.2041 10,244,670.746172

38.510883 0.2041 10,112,088.643305

38.514283 0.204 10,136,788.589581

38.517685 0.2041 9,802,471.804767

38.521087 0.2041 9,606,244.435189

38.524487 0.204 9,535,737.854478

38.527888 0.2041 9,122,329.381152

38.531290 0.2041 8,829,544.212727

38.534690 0.204 8,750,873.964483

38.538092 0.2041 8,760,695.121608

38.541493 0.2041 8,702,975.698913

38.544893 0.204 8,641,582.484791

38.548295 0.2041 8,674,623.868571

38.551697 0.2041 8,573,252.613550

38.555097 0.204 8,393,903.028822

38.558498 0.2041 8,307,970.089906

38.561900 0.2041 8,284,969.558000

38.565300 0.204 8,315,229.394679

38.568702 0.2041 8,228,258.392733

38.572103 0.2041 8,151,187.125434

38.575503 0.204 8,329,407.970339

38.578905 0.2041 8,454,421.025682

38.582307 0.2041 8,329,131.767293

38.585707 0.204 8,207,265.407817

38.589108 0.2041 8,156,703.753881

38.592510 0.2041 8,172,236.679420

38.595910 0.204 8,225,171.695288

38.599312 0.2041 8,377,443.175069

38.602713 0.2041 8,514,922.456346

38.606113 0.204 8,452,612.863456

38.609515 0.2041 8,357,948.653653

38.612917 0.2041 8,219,700.476735

38.616317 0.204 8,159,273.650097

38.619718 0.2041 8,193,843.417709

38.623120 0.2041 8,224,232.888492

38.626520 0.204 8,360,084.975961

38.629922 0.2041 8,376,469.754744

38.633323 0.2041 8,317,122.237469

38.636723 0.204 8,324,149.733958

38.640125 0.2041 8,203,118.770936

38.643527 0.2041 8,096,571.392002

38.646927 0.204 8,104,001.139442

38.650328 0.2041 8,133,576.688391

38.653730 0.2041 8,341,864.329200

38.657130 0.204 8,396,750.317914

38.660532 0.2041 8,297,411.110354

38.663933 0.2041 8,288,198.973432

38.667333 0.204 8,233,053.666504

38.670735 0.2041 8,208,315.647485

38.674137 0.2041 8,260,915.037425

38.677537 0.204 8,423,069.707530

38.680938 0.2041 8,422,022.647324

38.684340 0.2041 8,261,262.741056

38.687740 0.204 8,144,832.722681

38.691142 0.2041 8,240,986.848565

38.694543 0.2041 8,292,556.324876

38.697943 0.204 8,231,813.400049

38.701345 0.2041 8,125,146.435575

38.704747 0.2041 8,114,791.996155

38.708147 0.204 8,195,177.248053

38.711548 0.2041 8,077,002.004660

38.714950 0.2041 8,128,186.650317

38.718350 0.204 8,234,308.932520

38.721752 0.2041 8,198,527.703544

38.725153 0.2041 8,094,917.065088

38.728553 0.204 8,108,422.698557

38.731955 0.2041 8,051,706.477922

38.735357 0.2041 8,182,334.716026

38.738757 0.204 8,373,576.532383

38.742158 0.2041 8,064,055.457512

38.745560 0.2041 8,137,104.116001

38.748962 0.2041 8,373,998.035561

38.752362 0.204 8,272,313.324196

38.755763 0.2041 8,301,670.246918

38.759165 0.2041 8,236,525.885164

38.762565 0.204 8,122,576.718537

38.765967 0.2041 8,260,588.388226

38.769368 0.2041 8,289,028.700627

38.772768 0.204 8,166,428.394495

38.776170 0.2041 8,197,311.875013

38.779572 0.2041 8,193,662.563277

38.782972 0.204 8,030,096.937888

38.786373 0.2041 8,010,871.733394

38.789775 0.2041 8,201,174.822574

38.793175 0.204 8,301,520.643567

38.796577 0.2041 8,239,953.766560

38.799978 0.2041 8,278,428.508734

38.803378 0.204 8,143,161.796129

38.806780 0.2041 8,096,742.587754

38.810182 0.2041 8,278,298.803594

38.813582 0.204 8,293,771.325194

38.816983 0.2041 8,299,809.740417

38.820385 0.2041 8,185,640.632859

38.823785 0.204 8,353,680.658411

38.827187 0.2041 8,546,256.522999

38.830588 0.2041 8,484,450.141172

38.833988 0.204 8,455,834.382854

38.837390 0.2041 8,285,280.952534

38.840792 0.2041 8,392,415.253753

38.844192 0.204 8,503,548.328041

38.847593 0.2041 8,368,754.637254

38.850995 0.2041 8,318,778.300211

38.854395 0.204 8,193,293.345144

38.857797 0.2041 8,126,250.326540

38.861198 0.2041 8,077,428.039325

38.864598 0.204 8,121,759.096493

38.868000 0.2041 8,234,488.769514

38.871402 0.2041 8,130,620.534867

38.874802 0.204 8,116,923.577139

38.878203 0.2041 8,268,538.028407

38.881605 0.2041 8,300,450.343869

38.885005 0.204 8,153,602.120699

38.888407 0.2041 8,139,605.396467

38.891808 0.2041 8,292,327.953570

38.895208 0.204 8,285,808.376963

38.898610 0.2041 8,254,467.650729

38.902012 0.2041 8,280,455.327923

38.905412 0.204 8,341,220.081783

38.908813 0.2041 8,528,717.281581

38.912215 0.2041 8,962,783.806688

38.915615 0.204 9,910,176.926933

38.919017 0.2041 11,366,629.455077

38.922418 0.2041 13,455,704.365324

38.925818 0.204 16,338,347.070798

38.929220 0.2041 20,873,717.471673

38.932622 0.2041 27,541,021.776450

38.936022 0.204 36,237,908.602476

38.939423 0.2041 47,253,026.792213

38.942825 0.2041 60,305,802.904769

38.946225 0.204 75,356,966.130272

38.949627 0.2041 92,779,103.041232

38.953028 0.2041 111,035,991.673419

38.956428 0.204 127,251,232.695615

38.959830 0.2041 140,443,805.434717

38.963232 0.2041 146,992,249.823489

38.966632 0.204 147,231,083.426787

38.970033 0.2041 147,573,621.141168

38.973435 0.2041 143,684,754.192842

38.976835 0.204 132,311,241.623636

38.980237 0.2041 117,121,889.395887

38.983638 0.2041 99,883,135.800569

38.987038 0.204 83,735,097.926019

38.990440 0.2041 68,861,058.578071

38.993842 0.2041 54,788,548.417412

38.997242 0.204 42,563,300.874489

39.000643 0.2041 33,313,151.092593

39.004045 0.2041 26,929,667.274615

39.007445 0.204 21,976,069.270677

39.010847 0.2041 18,421,965.527382

39.014248 0.2041 16,047,611.289360

39.017648 0.204 14,454,029.314714

39.021050 0.2041 13,280,977.511092

39.024452 0.2041 12,409,313.299297

39.027852 0.204 12,159,841.684029

39.031253 0.2041 11,994,477.679826

39.034655 0.2041 11,569,281.610695

39.038055 0.204 11,456,130.201296

39.041457 0.2041 11,472,933.063327

39.044858 0.2041 11,184,851.468267

39.048258 0.204 10,889,027.842332

39.051660 0.2041 10,827,585.605077

39.055062 0.2041 10,752,725.829883

39.058462 0.204 10,385,594.619567

39.061863 0.2041 10,185,738.209934

39.065265 0.2041 10,205,472.200668

39.068665 0.204 9,917,865.894140

39.072067 0.2041 9,760,543.546400

39.075468 0.2041 9,699,473.114265

39.078868 0.204 9,499,111.609025

39.082270 0.2041 9,499,578.267218

39.085672 0.2041 9,418,299.403963

39.089072 0.204 9,346,306.197485

39.092473 0.2041 9,288,631.303990

39.095875 0.2041 9,065,612.986205

39.099275 0.204 9,003,029.636682

39.102677 0.2041 9,033,420.991365

39.106078 0.2041 8,977,371.320752

39.109478 0.204 8,910,518.085770

39.112880 0.2041 9,008,169.652258

39.116282 0.2041 9,054,569.952873

39.119683 0.2041 8,828,143.545165

39.123083 0.204 8,778,615.921940

39.126485 0.2041 8,787,122.895661

39.129887 0.2041 8,743,747.279082

39.133287 0.204 8,832,115.251032

39.136688 0.2041 8,633,468.117077

39.140090 0.2041 8,502,525.599417

39.143490 0.204 8,681,892.636816

39.146892 0.2041 8,789,007.087312

39.150293 0.2041 8,789,067.646623

39.153693 0.204 8,747,918.225142

39.157095 0.2041 8,725,424.487087

39.160497 0.2041 8,952,612.097715

39.163897 0.204 9,206,742.970373

39.167298 0.2041 9,136,335.167689

39.170700 0.2041 9,269,641.837759

39.174100 0.204 9,475,932.204678

39.177502 0.2041 9,455,033.370010

39.180903 0.2041 9,507,021.044229

39.184303 0.204 9,513,683.605714

39.187705 0.2041 9,720,770.666523

39.191107 0.2041 10,035,479.654739

39.194507 0.204 10,143,500.066476

39.197908 0.2041 10,411,322.620959

39.201310 0.2041 10,796,086.287586

39.204710 0.204 11,456,895.389168

39.208112 0.2041 12,459,512.279019

39.211513 0.2041 13,424,450.232748

39.214913 0.204 14,493,226.627661

39.218315 0.2041 15,961,280.911154

39.221717 0.2041 17,852,259.479079

39.225117 0.204 19,489,397.585959

39.228518 0.2041 20,834,309.848061

39.231920 0.2041 22,064,414.548092

39.235320 0.204 22,793,854.820669

39.238722 0.2041 23,135,649.764474

39.242123 0.2041 23,021,563.083202

39.245523 0.204 22,136,121.112244

39.248925 0.2041 20,973,010.111629

39.252327 0.2041 19,847,164.673728

39.255727 0.204 18,207,738.203853

39.259128 0.2041 16,471,395.715125

39.262530 0.2041 14,835,390.309048

39.265930 0.204 13,273,322.590941

39.269332 0.2041 12,117,768.281812

39.272733 0.2041 11,201,593.281999

39.276133 0.204 10,506,026.993034

39.279535 0.2041 9,867,879.470021

39.282937 0.2041 9,305,917.070645

39.286337 0.204 9,132,162.136835

39.289738 0.2041 9,013,952.661533

39.293140 0.2041 8,717,529.547643

39.296540 0.204 8,638,131.894774

39.299942 0.2041 8,585,089.283384

39.303343 0.2041 8,544,384.900869

39.306743 0.204 8,646,732.994990

39.310145 0.2041 8,604,365.945646

39.313547 0.2041 8,454,705.921426

39.316947 0.204 8,460,788.152654

39.320348 0.2041 8,640,860.417642

39.323750 0.2041 8,654,728.654707
[truncated: 47,823 more chars]
